# Exploring the Anti-Cancer Mechanism of Novel 3,4′-Substituted Diaryl Guanidinium Derivatives

**DOI:** 10.3390/ph13120485

**Published:** 2020-12-21

**Authors:** Viola Previtali, Helene B. Mihigo, Rebecca Amet, Anthony M. McElligott, Daniela M. Zisterer, Isabel Rozas

**Affiliations:** 1 School of Chemistry, Trinity Biomedical Sciences Institute (TBSI), Trinity College Dublin (TCD), 152-160 Pearse Street, D02R590 Dublin 2, Ireland; Viola.Previtali@iit.it (V.P.); mihigoh@tcd.ie (H.B.M.); 2 School of Biochemistry and Immunology, Trinity Biomedical Sciences Institute (TBSI), Trinity College Dublin (TCD), 152-160 Pearse Street, D02R590 Dublin 2, Ireland; ametr@tcd.ie (R.A.); dzistrer@tcd.ie (D.M.Z.); 3 Trinity Translational Medicine Institute, Trinity College and St James’s Hospital, D02R590 Dublin 8, Ireland; Tony.McElligott@tcd.ie

**Keywords:** 3,4′-bis-guanidino, 3-amino-4′-guanidino, diphenyl ether, phenyl pyridyl ether, intramolecular hydrogen bond, cancer cell viability, HL-60, BRAF, apoptosis

## Abstract

We previously identified a guanidinium-based lead compound that inhibited BRAF through a hypothetic type-III allosteric mechanism. Considering the pharmacophore identified in this lead compound (i.e., “lipophilic group”, “di-substituted guanidine”, “phenylguanidine polar end”), several modifications were investigated to improve its cytotoxicity in different cancer cell lines. Thus, several *lipophilic groups* were explored, the *di-substituted guanidine* was replaced by a secondary amine and the phenyl ring in the *polar end* was substituted by a pyridine. In a structure-based design approach, four representative derivatives were docked into an in-house model of an active triphosphate-containing BRAF protein, and the interactions established were analysed. Based on these computational studies, a variety of derivatives was synthesized, and their predicted drug-like properties calculated. Next, the effect on cell viability of these compounds was assessed in cell line models of promyelocytic leukaemia and breast, cervical and colorectal carcinomas. The potential of a selection of these compounds as apoptotic agents was assessed by screening in the promyelocytic leukaemia cell line HL-60. The toxicity against non-tumorigenic epithelial MCF10A cells was also investigated. These studies allowed for several structure-activity relationships to be derived. Investigations on the mechanism of action of representative compounds suggest a divergent effect on inhibition of the MAPK/ERK signalling pathway.

## 1. Introduction

Various interlinked signalling pathways are involved in cell proliferation, apoptosis or survival, and these processes are even more relevant in the case of tumour formation [1]. Oncogenic mutations in one of these signalling pathways, the Ras/RAF/MEK/ERK (MAPK/ERK) pathway, are observed frequently in many cancers [2,3]. Additionally, since mutations in RAF kinases are common events, these kinases (i.e., ARAF, BRAF and CRAF) have become very interesting therapeutic targets [4,5].

Most of the protein kinase inhibitors developed so far are ATP competitive and, based on the conformation of the protein kinase they bind to, have been broadly classified as type I (bind to the αC-helix-IN/DFG-IN conformation), type II (bind to the αC-helix-IN/DFG-OUT conformation), or type I/II (bind to the αC-helix-OUT/DFG-IN conformation) [6]. In addition, there are allosteric inhibitors, known as type III protein kinase inhibitors, which do not compete with ATP. These inhibitors tend to exhibit the highest degree of kinase selectivity because they exploit binding sites and regulatory mechanisms unique to particular kinases [7].

**Figure 1:**
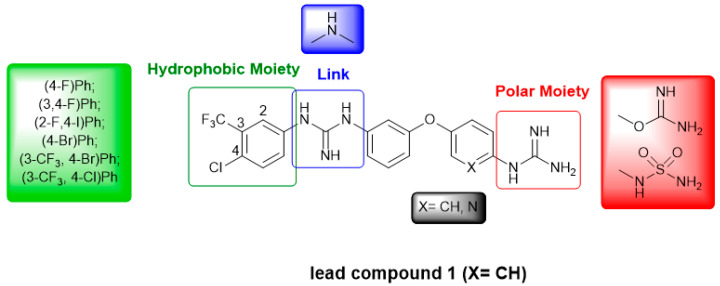
Structural modifications proposed for the optimization of the compound **1**.

Previously, we had identified a 3,4′-bis-guanidinium diphenyl derivative (**1**, Figure 1) that demonstrated strong cytotoxicity, mediated through induction of apoptosis, in colorectal cancer cells containing wild type(wt)-BRAF and mutated ^V600E^BRAF [8,9]. Compound **1** also inhibited ERK1/2 signalling, EGFR activation, as well as Src, STAT3 and Akt phosphorylation. We also showed that **1** did not inhibit ATP binding to BRAF, but a radiometric assay of BRAF activity indicated that this was inhibited in vitro. From these studies, we hypothesised that **1** could inhibit BRAF as a type III inhibitor. We propose that the positively charged guanidines present in compound **1** could interact with the negatively charged phosphates of the ATP present in the active state of all kinases [10].

Considering that only very recently a crystal structure of a BRAF protein kinase in complex with an ATP analogue (AMP-PCP) and MEK has been resolved (PDB 6U2G) [11], we had previously constructed a model that reproduced an active form of this kinase including ATP to model potential type III inhibitors [12].This in-house model allowed us to explore the potential allosteric inhibition through ATP binding of compound **1** and it was considered a good target model for structure-based design when no crystallographic data was available.

Accordingly, in this article, we first present a computational study of a series of guanidine-based di-aromatic systems representative of all the compounds proposed with a simplified version of our in-house active BRAF model to explore their potential as type-III allosteric inhibitors. Additionally, we describe the preparation of the compounds proposed and the study of the anti-proliferative and pro-apoptotic activity on cancerous cell lines as well as their effect on the MAPK/ERK signalling pathway.

## 2. Results

Considering the structure of compound **1** (“link” -blue box-, “polar moiety” -red box- and “hydrophobic moiety” -green box-; see Figure 1), we explored a number of modifications in the different moieties, while maintaining the diaryl ether core due to its versatility and suitability for synthesis. First, several “hydrophobic” substituents have been considered (green box in Figure 1), since in our previously reported computational model [12], this section of the molecule seems to interact with a hydrophobic pocket. Next, based on previous computational studies [9], the effect of the length of the molecule on its activity has been studied by replacing the disubstituted guanidinium in the “link” by a secondary amine (blue box in Figure 1). Additionally, we explored the effect of substituting one of the phenyl rings by a pyridine to lock the orientation of the guanidinium by means of intramolecular hydrogen bonds (IMHBs) [13]. Finally, in the “polar” region, different guanidine surrogates (i.e., isourea and sulfamide) have been tested (red box in Figure 1).

### 2.1. Molecular Modelling Studies

**Figure 2:**
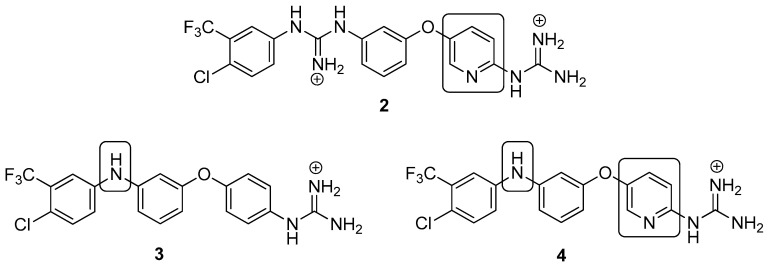
Structures of the proposed new derivatives of **1** (compounds **2**, **3** and **4**), which docking to the in-house triphosphate (TP)-containing BRAF simplified model was studied.

We had previously reported the docking of compound **1** into our in-house ATP-containing BRAF model [12]; here, we are using a simplified model containing triphosphate (TP) instead of ATP since the interactions of interest only occur with the phosphates and adjacent hydrophobic pocket. Thus, in order to validate our TP-containing BRAF model, we first docked compound **1** (Figure 1) and considering that similar outcomes were observed, we used the TP-containing structure and similar conditions for the rest of the computational studies. Accordingly, a set of representative structures of all the compounds proposed were selected; compound **2** contains a pyridine instead of a phenyl ring as it was in compound **1**, in compound **3** the di-substituted guanidine of **1** has been changed to a secondary amine and compound **4** shows both modifications (Figure 2).

As in our previous modelling studies [12], we found that compound **1** interacts with BRAF through an allosteric region found near the ATP binding site and with the TP system. The main interactions established involved bifurcated hydrogen bonds (HBs) between the mono-substituted guanidinium and two negatively charged O atoms of a phosphate (NH^…^O distances: 1.6, 2.9 and 2.7 Å, Figure S1) and a parallel HB interaction between the di-substituted guanidinium and the carboxylate of Glu648 (NH^…^O distances: of 2.0 and 2.6 Å, Figure S1). Both sets of HBs are reinforced by ionic interactions due to the charged nature of guanidinium, phosphate and carboxylate groups. Additionally, interactions are observed in the lipophilic pocket with the (4-Cl,3-CF_3_)Ph moiety; thus, the CF_3_ group interacts with Met620 and the Cl with Trp619 (Figure S1).

**Figure 3:**
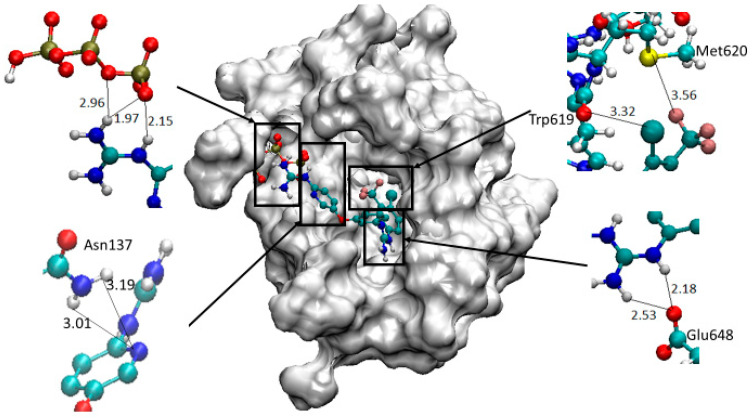
Docking of derivative **2** in the TP-containing BRAF simplified model indicating the bifurcated (up left, bottom left and right) and single (up right) hydrogen bond (HB) interactions observed. Distances are expressed in Å.

Likewise, when derivative **2** is docked into the TP-BRAF simplified model, it forms ionically reinforced bifurcated HBs between the mono-substituted guanidinium and two negatively charged O atoms of TP (Figure 3). Likewise, a bifurcated HB interaction between the di-substituted guanidinium and Glu648 and van der Waals contacts at the lipophilic pocket with the (4-Cl,3-CF_3_)Ph moiety were also found for compound **2** (Figure 3). The newly introduced pyridine seems to form a HB with Asn137 as well as an IMHB that locks the mono-substituted guanidinium (Figure 3). This conformational restriction could result in increased affinity.

Replacement of the di-substituted guanidine by an -NH- leads to a significantly shorter molecule as in system **3** (Figure 2). Upon docking to the TP-containing simplified BRAF, we observed that the mono-substituted guanidinium still forms the expected bifurcated HBs. Compound **3** also fits within the hydrophobic pocket of the target establishing weaker contacts (longer interaction distances) with Met620 and Trp619. Additionally, the newly introduced -NH- group forms a HB with one of the O atoms of Glu648 (Figure S2).

Finally, the docking study of compound **4** (Figure 2) into the aforementioned target reproduce the results observed for the mono-substituted guanidinium pyridine system in analogue **2** by establishing bifurcated HBs with the TP, IMHB between guanidinium and pyridine as well as contacts between the pyridine N and Asn137. Additionally, as the -NH- shortens the structure, similar interactions to those seen for compound **3** are observed, i.e., the -NH- group forms a HB with Glu648 and contacts within the hydrophobic pocket are found (Figure S3).

All this work was carried out prior to the recent publication of the crystal structure of BRAF containing an analogue of ATP (AMP-PCP) [11], and in order to validate our in-house BRAF-ATP model [12] we now superimposed both the reported crystal structure with our model finding an RMSD of 2.53 Å between them and with both ATP-like systems occupying the same pocket and with very similar orientations (Figures S4 and S5). This level of similarity gives us confidence on the docking studies performed with our BRAF-TP model.

The G-scores obtained for the best-poses obtained were very similar for the four compounds studied (around −7.7 kcal/mol); hence, the interaction with the target was favoured in all the cases but did not help to discriminate among the four compounds. In summary, according to the docking studies all the compounds proposed seem to establish favourable interactions with BRAF, suggesting a possible type III allosteric binding.

### 2.2. Synthesis

**Scheme 1:**
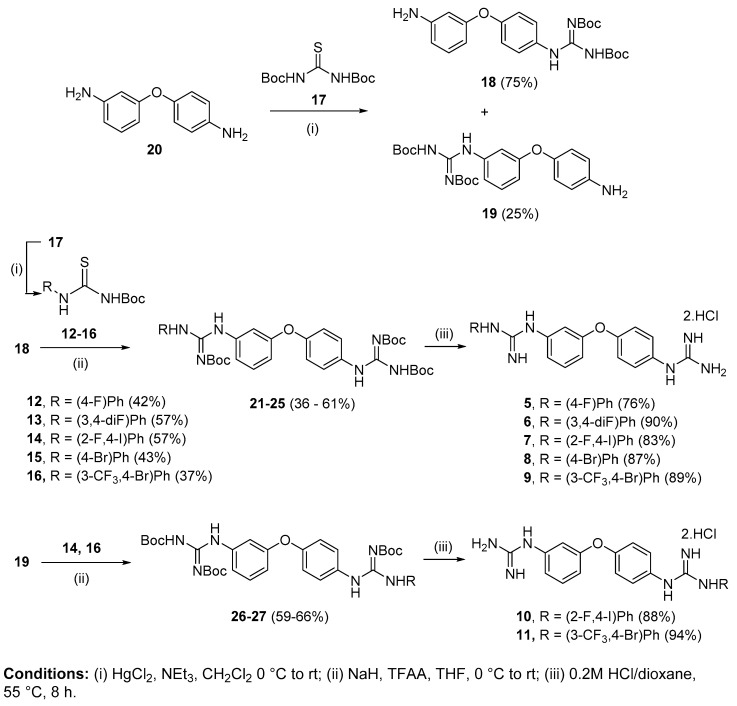
Preparation of mono-substituted 3,4’-bis-guanidinium diphenyl ether derivatives.

The synthesis of the analogues of compound **1**, 3,4′-bis-guanidino diphenyl ethers **5**–**11**, required the preparation of the corresponding *N*-aryl-*N’*-Boc-protected thioureas (**12**–**16**) following a procedure previously developed in the group starting from *N*,*N’*-bis*-*(*tert*-butoxycarbonyl)thiourea **17** (Scheme 1) [8]. Given the diverse electron-withdrawing effect of differently substituted commercially available anilines, different yields of the corresponding thioureas **12**–**16** were obtained (37–57%, see details in ESI). The corresponding Boc-protected mono-guanidines **18** and **19** were prepared by reaction of commercially available 3,4′-dianiline ether **20** and *N*,*N’*-bis*-*(*tert*-butoxycarbonyl)thiourea **17** in the presence of HgCl_2_ and NEt_3_ yielding **18** and **19** as a mixture that was separated by column chromatography in good and moderate yields (Scheme 1) [14].

Next, the *N*-aryl-*N’*-Boc-protected thioureas **12**–**16** were reacted with **18** or **19** under our standard conditions (HgCl_2_ and NEt_3_) to yield the corresponding Boc-protected 3-arylguanidino-4′-guanidino (**21**–**25**) and 3-guanidino-4′-arylguanidino (**26**–**27**) diphenyl ether derivatives (Scheme 1).

**Scheme 2:**
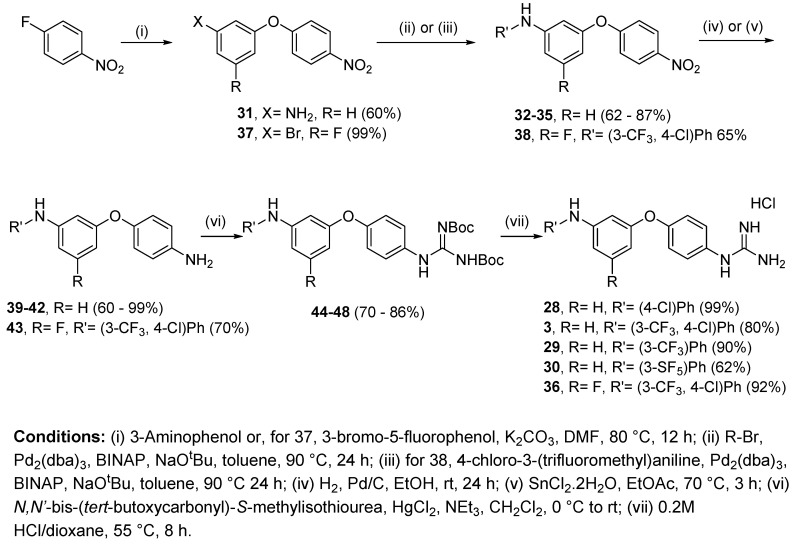
Preparation of 3-amino,4’-guanidine diphenyl ether derivatives.

In order to prepare compound **3** and its analogues **28**–**30**, the nitrophenyl precursor **31** was synthesised by a S_N_Ar reaction between commercially available 3-aminophenol and 1-fluoro-4-nitrobenzene [15]. Compounds **32**–**35** were synthesized using a Buchwald–Hartwig cross-coupling; the success of this reaction depends on variables such as ligand, Pd source, base and solvent [16]. The conditions chosen (Pd_2_(dba)_3_ 3 mol%, BINAP 3 mol%, NaOtBu 1.4 eq. in dry toluene (2 mL mmol^−1^) at 90 °C) afforded the proposed compounds **32**–**35** in high yields (62–87%, Scheme 2).

With the intention of probing the effect of branching in the diphenyl ether core, we prepared the fluoro derivative **36** for which we synthesised precursor **37** by using commercially available 3-bromo-5-fluorophenol that in the presence of K_2_CO_3_ and DMF quantitatively reacted with 4-fluoronitrobenzene. Then, **37** was used for the Buchwald–Hartwig coupling with 4-chloro-3-(trifluoromethyl)aniline to afford compound **38** in good yield (Scheme 2).

Precursors **32**–**35** and **38** were then subjected to selective reduction of the nitro group to the amine that will serve as handle for the introduction of the guanidine moiety. Nitro reduction of compounds **34** and **35** was achieved using catalytic hydrogenation (H_2_, Pd/C 10 mol%) yielding aniline derivatives **41** and **42**; however, in the case of chloro-derivatives **32**, **33,** and **38**, selective reduction was achieved with the use of tin(II) chloride dihydrate (SnCl_2_·2H_2_O) to produce anilines **39**, **40,** and **43** [17]. Utilising our standard conditions, a guanidine moiety was introduced affording Boc-protected mono-guanidines **44**–**48** (Scheme 2).

**Scheme 3:**
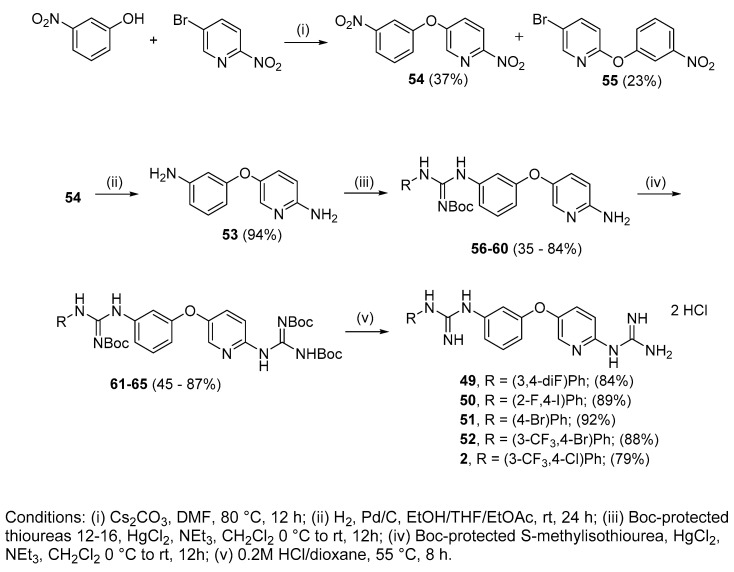
Preparation of 3,4’-bis-guanidine pehylpyridyl ether derivatives.

In order to prepare the 3,4′-bis-guanidino phenyloxypyridines **2**, **4,** and **49**–**52**, the starting 5-(3-aminophenoxy)pyridin-2-amine (**53**) was synthesized. Thus, S_N_Ar between 5-bromo-2-nitropyridine and 3-nitrophenol yielded the previously reported mixture of isomers (**54** and **55**) [18], which were separated by chromatography. Further hydrogenation of **54** gave the desired product **53** in good yield (Scheme 3) [15,19].

Compound **53** was then reacted with Boc-protected thioureas **12**–**16**, under our standard conditions to yield Boc-protected mono-guanidines **56**–**60** [8]. Subsequent guanidylation at position 2 of the pyridine ring was carried out with commercial *N,N’*-bis-(*tert*-butoxycarbonyl)-*S*-methylisothiourea to obtain Boc-protected compounds **61**–**65** (Scheme 3).

**Scheme 4:**
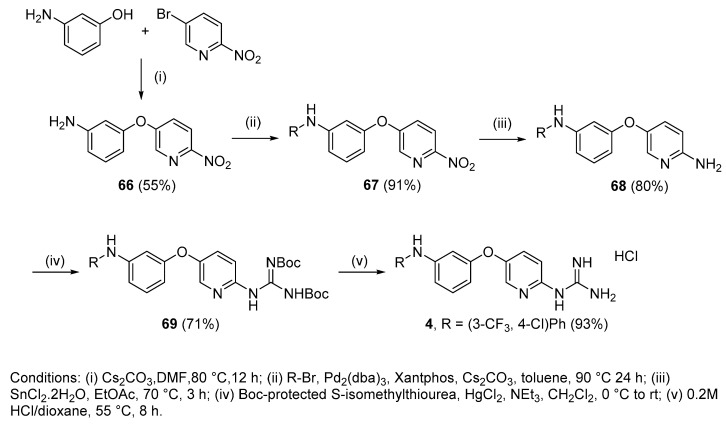
Preparation of 3-amino-4’-guanidine phenylpiridyl ether derivatives.

Preparation of the corresponding precursors for compound **4** required the synthesis of 3-((6-nitropyridin-3-yl)oxy)aniline **66** through a S_N_Ar between 5-bromo-2-nitropyridine and 3-aminophenol (Scheme 4).

Preparation of the arylanilino nitropyridine **67** was next attempted following the previous conditions described for the synthesis of **32**–**35** and **38** with poor results. However, Pd-catalysed reaction using of Xantphos and Cs_2_CO_3_ yielded compound **67** in good yield. Next, SnCl_2_·2H_2_O reduction conditions afforded compound **68** that was then subjected to guanidylation using *N,N’*-bis-(*tert*-butoxycarbonyl)-*S*-methylisothiourea to obtain Boc-protected compound **69** (Scheme 4).

**Scheme 5:**
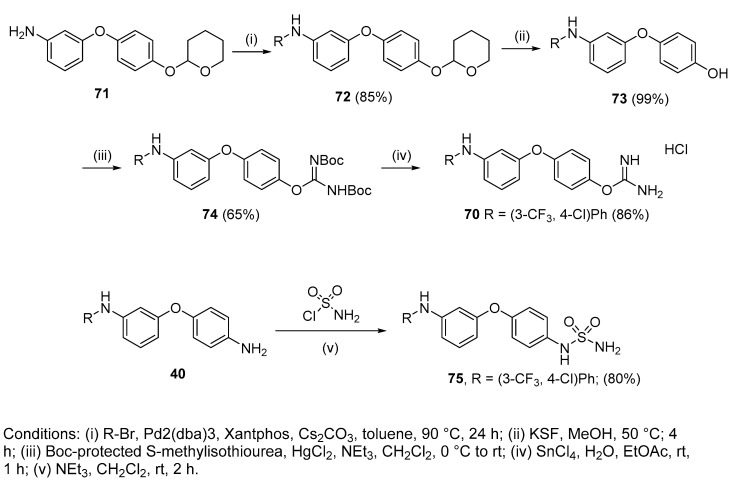
Preparation of 3-amino-4’-isourea and 3-amino-4’-sulfamido diphenyl ether derivatives.

With the aim of introducing diversity to the “polar” moiety of compound **3**, we also explored the substitution of the guanidinium system by an isouronium cation or a sulfamide (Scheme 5). Thus, preparation of the 3-anilino-4’-*O*-isourea phenylozybenzene derivative **70** involved the use of the previously synthesised compound **71** [20] as starting material for the synthesis of the intermediate **72** through the mentioned conditions for a Buchwald–Hartwig coupling (Pd_2_(dba)_3_, Xantphos, Cs_2_CO_3_, toluene, 90 °C, 24 h).

Compound **72** was then deprotected with montmorillonite KSF to obtain the corresponding phenol **73**, which OH was then amidylated using standard conditions previously described by us [20] to yield the corresponding Boc-protected isouronium **74** (Scheme 5). Sulfamide **75** was synthesised by treating amine **40** with sulfamoyl chloride (previously prepared from chlorosulfonyl isocyanate and formic acid [21,22]) to afford compound **75** in good yield (Scheme 5).

All Boc-protected precursors were deprotected using HCl 4M/dioxane to yield compounds **2**–**11**, **28**–**30**, **36**, **49**–**52** (Scheme 1, Scheme 2, Scheme 3 and Scheme 4) and SnCl_4_ to obtain compound **70**. The purity of all the final hydrochloride salts was determined by HPLC, where a minimum purity of 95% was required before proceeding to biological testing (ESI).

### 2.3. Predicted Physicochemical and In Vitro ADME Properties

Attention to physicochemical and pharmacokinetic properties of active molecules should be given at the early stages of their design in order to shorten their development to a drug. However, these properties are not always easy to be experimentally evaluated and, hence, computational approaches represent a good solution to get a general idea of the potential of compounds as drugs. Thus, to assess the drug-likeness of the compounds studied, we utilized SwissADME [23] and ChemAxon’s Marvin [24] to computationally evaluate the mentioned properties and the calculated values are reported in Tables S1–S3 (ESI). All proposed compounds have a molecular weight (MW) <500 Da, except for 3,4′-bis-guanidine phenyloxybenzenes **7**, **9**, **10** and **11** or phenyloxypyridines **50** and **52**, in which the MW exceeds by 7–10 units this threshold; such small exceptions are considered acceptable [25]. The consensus logP_o/w_ [23] for all synthesised compounds is reported in Table S2 (ESI) indicating that, overall, all the compounds have a logP < 5. Topological Polar Surface Area (TPSA), which is an indicator of HB formation and a commonly used metric for a drug’s ability to permeate cells, was calculated for all the synthesised molecules and all of them have values <140 Å^2^, which is the limit suggested in the literature for poor cell membrane permeation [25,26,27]. Specifically, shorter 3-amino-4′-guanidines have much lower TPSA (80–100 Å^2^) than 3,4′-bis-guanidinium analogues (120–135 Å^2^).

The BOILED egg graph presents a correlation between calculated logP and calculated TPSA and is an intuitive simultaneous prediction of two key ADME parameters, i.e., the passive gastrointestinal (GI) absorption and brain access (blood brain barrier, BBB) [23,28]. Compounds that fall into the white part of the graph are likely to undergo GI absorption and those that fall into the yellow part of the graph are likely to be brain permeant. Accordingly, all of our derivatives could undertake passive GI absorption (except pentafluorosulfanyl derivative **30** and sulfamide **75**), but none of them can cross the BBB (Figure S6). In addition, SwissADME enables the estimation for a chemical to be a substrate of the permeability glycoprotein (P-gp), which is the most important ATP-binding cassette transporter responsible for an active efflux through biological membranes, e.g., from the GI wall to the lumen or exiting the brain) [28,29]. Thus, as reported in the legend of Figure S6, red dots indicate that all of our compounds, except compounds **49**–**51** (blue dots), are non-substrates of P-gp (Table S3).

The theoretical pK_aH_ values of all the synthesised compounds calculated with Marvin are reported in Table S1. The results indicate that exchanging the *para* mono-substituted guanidinium (compound **3**) by an isouronium moiety (compound **70**) results in a pK_aH_ decrease (less basic molecule). Interestingly, the pyridine derivatives (**49**–**52**, **2** and **4**) show lower pK_aH_ values than their diphenyl counterparts (**6**–**9**, **1** and **3**, respectively), probably due to the IMHB formed between the *para* guanidinium and the pyridine ring.

Solubility is not an easy parameter to model in silico, but SwissADME gives an estimation of the solubility class based on three predictors, two topological and one fragmental method [23]. According to this program, all our molecules are poorly soluble in aqueous environments (Table S2). For the preclinical evaluation of our molecules, their solubility in EtOH/DMSO is still acceptable, but future work will have to be carried out to achieve full water solubility.

Other numerical descriptors are used to assess drug-likeness including the number of HB acceptors and donors (HBAs and HBDs) or the number of rotatable bonds (RotB) and the results obtained for this set of compounds are shown in Table S1 [27]. According to these descriptors, in general, all studied compounds fulfil drug-like conditions.

### 2.4. Biochemical Studies

#### 2.4.1. Cell Viability in Cancerous and Non-Cancerous Cell Lines

Cell viability and proliferation assays were used to evaluate the in vitro cytotoxicity of all synthesised compounds in a variety of different cancer cell lines using the alamarBlue^®^ viability assay and the results are presented in Table 1 and Table 2. The sensitivity of cancer cells to drugs can often be compromised by PIK3CA, Ras and BRAF mutations. To determine whether such mutations are critical to the efficacy of our new compounds we tested them in a range of cancerous cell lines expressing different mutations. Firstly, we used the HL-60 (human promyelocytic leukaemia, NRas mutated) cell line for general cytotoxicity screening of all compounds. Next, the most active derivatives were studied in MCF-7 (breast adenocarcinoma, Ras/RAF wild type, and PIK3CA mutant), HeLa (cervical carcinoma, Ras/RAF and PIK3CA wild type), as well as HCT-116 and HKH-2 (colorectal carcinoma, KRas mutant and mutated KRas disrupted, respectively) cell lines [8]. Lastly, toxicity against MCF-10A, which is a non-tumorigenic epithelial cell line, was also assessed for one of the most promising compounds (**4**). The graphs representing the viability results with the HL-60, MCF-7, HeLa, HCT-116, and HKH-2 cancer cell lines for compound **1** and derivatives **2**, **3,** and **4** are shown in Figures S7–S10 (ESI). Sorafenib (a known inhibitor of protein kinases including VEGFR, PDGFR and RAF [30,31]) was used as a positive control in all viability assays.

The results obtained with the HL-60 cell line (Table 1) for the 3,4′-bis-guanidine derivatives **5**–**11** show, in general, more cytotoxicity than the previously tested compound **1** [8]. However, compounds **5** and **6**, which carry 3-F and 3,4-diF phenyl groups, respectively, give IC_50_ values above 100 μM; this drop in activity is a clear indication of the importance of the size and nature of the substituents on the phenyl ring [32]. The presence of 2-F and 4-I substituents in the hydrophobic moiety of derivative **7** resulted in an IC_50_ value (8.63 μM) similar to **1**. Substitution of the 4-Cl in compound **1** by a 4-Br (i.e., compound **9**), gave a four-fold increase in activity, indicating that the halogen in this hydrophobic moiety could establish a halogen-bond with a Lewis base in the protein binding site. Additionally, compound **8** had decreased activity because of the absence of the 3-CF_3_ substituent; this is an indication that such a big and polarized halogen atom can only result in a beneficial increment in activity when a bulky lipophilic substituent as CF_3_ is present at position 3.

Cytotoxicity results of compounds **10** and **11** (lipophilic moiety in 4′-position of the phenyloxyphenyl core instead of the 3-position) with HL-60 cells show that compound **10** maintains a similar IC_50_ value as **7**; however, **11** has a reduction of activity compared to **9**.

Compound **3**, which is a shorter version of **1** (-NH- link instead of a di-substituted guanidinium) shows increased cytotoxicity in HL-60 cells (IC_50_ = 3.08 μM). Similar to what was observed for the 3,4′-bis-guanidine derivatives, removal of the 3-CF_3_ group in the lipophilic section caused decreased activity in the shorter analogue **28** (7.50 μM). Interestingly, removal of the 4-halogen in this lipophilic section did not affect the IC_50_ value of compounds **29** and **30** compared to their analogue **3**. This could be explained by the compensation of bulky and lipophilic effects when going from trifluoromethyl (-CF_3_) to bulkier pentafluorosulfanyl (-SF_5_) substitution.

The 3,4′-bis-guanidines phenyloxypyridines **49**–**52**, **2** and **4** show, overall, increased HL-60 cytotoxicity than the previously discussed derivatives. Compound **2**, the pyridine analogue of **1**, has an IC_50_ value of 2.36 μM, a four-fold increased activity compared to **1**. Likewise, compound **52** with a 3-CF_3_-4-Br phenyl system, shows a low IC_50_ of 1.53 μM. Surprisingly, the introduction of a pyridinoguanidinium system as in compound **49** (11.61 μM) instead of a phenylguanidinium moiety as in **3** (>100 μM) results in increased HL-60 cytotoxicity. Even though this is not the most active compound of the series, it is a clear indication of the importance of the pyridinoguanidinium system in improving cytotoxicity in HL-60 cells.

Interestingly, compound **4**, which includes both a shorter -NH- link and the pyridinoguanidinium moiety has a relatively low IC_50_ value of 3.48 μM. Compound **70**, with a -NH- link and an isouronium instead of the para guanidinium, shows similar cell viability as the guanidinium analogue **3**. From these results we can deduce that the isouronium cation has a similar behaviour to the guanidinium cation, as we had previously observed in the 3,4′-bis-guanidinium series [20]. Finally, compound **75**, where the *para* guanidinium is replaced by a sulfamide, shows a decreased cytotoxicity in HL-60 cells to 9.14 μM, indicating the importance of the guanidinium or isouronium cations.

The IC_50_ values obtained for selected compounds in the MCF-7 cell line (Table 2, Figure S8) were similar to the results obtained for the HL-60 cell line with most values in the low μM range. Compounds **3** and **4** (IC_50_ = 2.02 and 3.73 μM, respectively) are still the most cytotoxic agents compared to compound **1** (IC_50_ of 9.30 μM). The pyridine ring present in compounds **4** and **2** still appears responsible for the improved activity, even though less accentuated than in the HL-60 cell line. Instead, the isouronium version of compound **3**, compound **70**, has an increased IC_50_ in MCF-7 cells compared with HL-60 cells indicating a certain degree of cell selectivity.

We have also evaluated the effect of compounds **2**, **3**, **4,** and **1** on the viability of the HeLa cell line and the results are reported in Table 2 (Figure S9). In this cell line, compound **4** showed again the lowest IC_50_ value (1.33 μM). The rest of the compounds maintained similar cytotoxic activity in HeLa cells compared to HL-60 and MCF-7 cells.

It is known that sorafenib was originally developed as an inhibitor of the Ras effector RAF, and there are studies showing that sorafenib enhances the therapeutic efficacy of rapamycin in certain colorectal cancers [33]. The IC_50_ results in Table 2 (Figure S10) show that compound **1** has more potency in the HCT116 *KRAS* mutated cancer cell line (9.96 μM) than in the HKH-2 *KRAS* wt isogenic form (19.18 μM). Remarkably, its cytotoxic effect is like that of sorafenib in HCT116 (6.79 μM), but not in HKH-2, where sorafenib has a much lower IC_50_. Similar activity in both cell lines is reached with compound **2**, while **4** is revealed to be the most active of the series with an IC_50_ of 4.59 μM in HCT116 and 2.88 μM in HKH-2. Interestingly, compound **3**, which has a phenyl group instead of the 2-pyridinyl of compounds **2** and **4**, shows poor cytotoxic activity in both cell lines.

Searching for a relationship between physicochemical properties and cytotoxicity, we observed a trend between the calculated logP and the HL-60 IC_50_ values obtained for all synthesised compounds (except the inactive **5** and **6**, IC_50_ > 100 μM) (Figure S11). This supports our hypothesis that the hydrophobic moiety of our molecules interacts with a specific allosteric hydrophobic pocket in protein kinases, justifying the use of bulkier and more lipophilic substituents (larger logP) in order to obtain improved anti-cancer activity (lower IC_50_ values).

**Figure 4:**
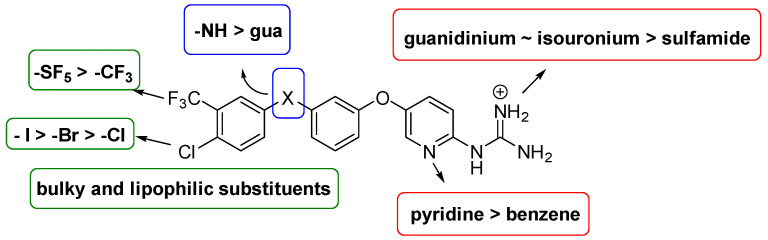
Structure Activity Relationship (SAR) deduced from the analysis of the HL-60 cytotoxicity results.

Therefore, some structure–activity relationships (SARs) can be drawn from the cell viability assays (Figure 4): (i) the hydrophobic moiety is necessary for the activity; particularly, bulky and lipophilic substituents improve the cytotoxicity of these compounds (I- or Br- substitution at the 4-position better than Cl-), and a substituent at the 3-position is required to maintain efficacy (CF_3_ or SF_5_); (ii) replacement of the di-substituted guanidinium in compound **1** (position 3 of the phenyloxyaryl core) by a shorter -NH- link results in compounds with better cytotoxicity; (iii) a mono-substituted guanidinium group as in compound **1** (position 4′ of the phenyloxyaryl core) gives the greatest cytotoxic activity; additionally, incorporating a 2-pyridinyl instead of a phenyl group, attached to this guanidinium facilitates forming an IMHB that seems to affect positively the cytotoxic activity.

Considering the promising cytotoxicity results obtained with the cancer cell lines, we also evaluated the potential toxicity of the most efficient derivative (compound **4**) in the non-cancerous cell line MCF10A (human mammary epithelial cell line). We observed that this compound **4** only shows toxicity towards MCF10A at a high concentration of 100 μM while at lower concentrations of 10 and 1 μM the compound does not appear to affect in any way cell viability (Figure S12).

#### 2.4.2. Apoptosis Assay in HL-60

**Figure 5:**
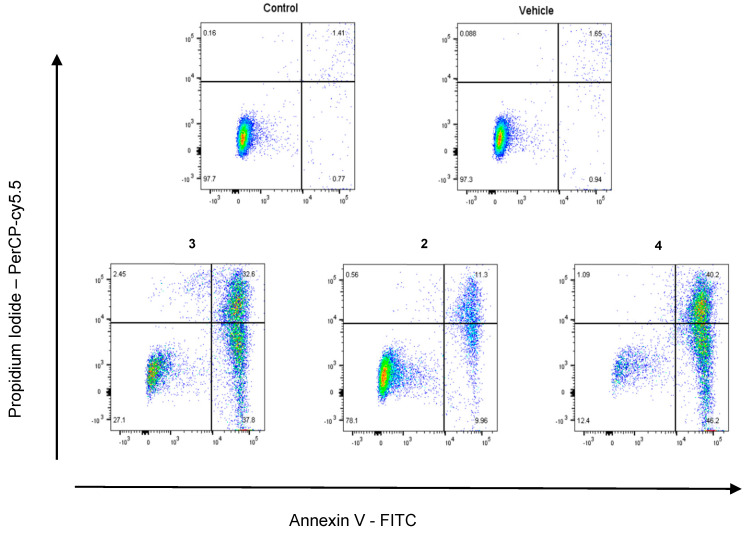
Annexin V-FITC vs. PI flow cytometry analysis of HL-60 cancer cells treated with compounds **3** (5 μM), **2** (5 μM) and **4** (4 μM) for 48 h. These figures are representative of three independent experiments. The viable cells, early apoptotic, necrotic and late apoptotic cells are represented by the lower left, lower right, upper left and upper right quadrants, respectively.

We have previously reported that compound **1** induces 68.9 ± 0.1% apoptosis in HL-60 cells at a 10 μM concentration after 48 h [8]. Accordingly, in order to evaluate the apoptotic effect of the most relevant derivatives (**2**, **3**, and **4**), HL-60 cells were treated with these compounds (at 5 µM, 5 µM, and 4 µM concentration, respectively) for 48 h, stained with annexin V-FITC/PI and analysed using flow cytometry (Figure 5).

The results presented in Figure 5 and Figure S13 show that, under these conditions, a more potent induction of apoptosis was observed with **3** (82.5 ± 11.3%) and **4** (92.7 ± 5.5%) compared to compound **2** (30.7 ± 11.6%). As the alamarBlue assay indirectly assesses cytotoxicity by quantifying cell viability and proliferation, these data suggest that compound **2** may have a predominantly cytostatic mechanism of action.

#### 2.4.3. Effect on the MAPK/ERK Pathway

**Figure 6:**
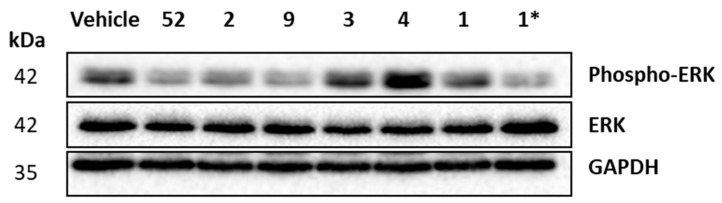
Western immunoblot of HL-60 cell extracts following incubation with compounds **2**, **3**, **4**, **9**, **52** and **1** (as a control). HL60 cells were seeded at 2 × 10^5^ cells/mL and were treated with either vehicle [0.5% EtOH (*v/v*)], compounds **52**, **2**, **9**, **3** and **4** (5 µM) or compound **1** (5 and (*) 10 µM, as in reference [9]) for 16 h. Cells were lysed and equal amounts of protein were loaded and separated on 15% SDS-PAGE gels, transferred to PVDF membrane and probed with antibodies against total and phosphorylated ERK. Anti-GAPDH was used as a loading control. Results are representative of 2 independent experiments.

Taking into account the positive binding results obtained in the docking studies to the BRAF-ATP model and the promising cytotoxicity shown in several cancer cells, we next explored the effect of compounds with IC_50_ near to that of sorafenib (**1**, **2**, **3**, **4**, **9,** and **52**) on the MAPK/ERK pathway. Thus, using Western immunoblot analysis of HL-60 extracts, we investigated the expression and phosphorylation levels of ERK (as an indication of ERK activation), which is the downstream effector of the Ras/BRAF signalling pathway (Figure 6).

We observed that compounds **3** and **4** do not appear to interfere with the Ras/BRAF signalling pathway; however, compounds **2**, **9,** and **52** have a similar effect as lead compound **1** (both at 5 and 10 μM) in inhibiting ERK phosphorylation and therefore inhibiting the Ras/BRAF pathway. These results may suggest that the potent cytotoxicity observed with these derivatives may be due to different mechanisms of action. Therefore, compounds **2**, **9,** and **52**, which are all 3,4′-bis-guanidino phenyloxy(phenyl or pyridyl) analogues of **1** with similar lipophilic moieties (3-CF_3_,4-(Br/Cl)-Ph), may exert their cytotoxicity by interfering with the Ras/BRAF pathway. On the contrary, compounds **3** and **4** (shorter 3-amino-4′-guanidino phenyloxy(phenyl or pyridyl) derivatives) may act in a different signalling pathway or in the same pathway but through a different mechanism from compound **1** [6]. Pulikakos and co-workers have reported that the biochemical effect of a RAF inhibitor on ERK signalling would be the combined outcome of different mechanisms [34], clearly indicating the complexity of the biological target and opening the door to future studies to understand the mechanism of action of these compounds.

## 3. Discussion

Considering the promising results previously obtained for lead compound **1** we explored several modifications to improve its cytotoxicity in different cancer cell lines. Accordingly, we designed a variety of compounds where different changes have been introduced: several lipophilic groups were considered; the di-substituted guanidine was replaced by a secondary amine; the phenyl ring was exchanged with a pyridine; and the mono-substituted guanidine in the polar side was switched to an isourea or a sulfanylamide group.

Molecular docking was utilised to understand the interactions between the proposed biological target and model compounds **1**–**4**. Thus, considering the putative activity of **1** as a type-III kinase inhibitor, compounds **1**–**4** were docked into an in-house model of an active TP-containing BRAF kinase. All final poses exhibit similar interactions between the lipophilic moiety and the lipophilic pocket, as well as between the mono-substituted guanidinium and one of the phosphate groups of TP.

Based on this computational study, 3,4′-bis-guanidino diphenyl ether derivatives **5**–**11** were prepared by reacting the corresponding 3,4′-diamino diphenyl ether with conveniently substituted Boc-protected thioureas. Additionally, synthesis of the 3-amino-4′-guanidino diphenyl ethers **3**, **28**–**30** and **36** required the preparation of the corresponding starting diamines. Furthermore, preparation of 3,4′-bis-guanidino and 3-amino-4′-guanidino phenylpyridyl ether derivatives **2**, **49**–**52,** and **4**, required different synthetic approaches involving Buchwald–Hartwig coupling. Finally, the 3-amino-4′-isouronium **70** and 3-amino-4′-sulfonamido **75** derivatives were prepared following specific synthetic routes. All compounds were obtained as hydrochloride salts and their purity was determined to be >95% by HPLC before proceeding to biological testing.

A screening of the cytotoxicity of all compounds was performed in the HL-60 cell line and the more potent compounds were selected for further biological evaluation in MCF-7, HeLa, HCT116, and HKH-2 cell lines. These cell viability studies revealed that these compounds can inhibit cell proliferation in the low μM range, showing up to a nine-fold increase in cytotoxicity compared to lead compound **1**. All modifications explored generated SAR information which helped to understand the structural requirements for a more potent cytotoxicity. Cytotoxicity of compound **4** was also evaluated against non-tumorigenic MCF10A and the results show that, at active concentration in cancer cell lines, compound **4** has no toxic effects to non-tumorigenic cells.

Flow cytometry in HL-60 cell lines was also performed to assess the apoptotic effect of compounds **2**, **3**, and **4**. The results are in agreement with the corresponding structures; thus, mono-guanidinium compounds (**3** and **4**) induce a stronger apoptotic effect in HL-60 cells (82% and 92%, respectively) compared to the bis-guanidinium compounds (**1** and **2**, around 60%^7^ and 30%, respectively).

With the aim of determining whether compounds **1**–**4**, **9,** and **52** exert their anticancer activity by interfering with the Ras/BRAF pathway (as previously suggested by us for lead compound **1**), Western immunoblot analysis was performed with HL-60 cell extracts measuring the activation of the downstream effector ERK. Thus, we observed that compounds **1**, **2**, **9,** and **52** (3,4′-bis-guanidine phenyloxy(phenyl or pyridyl) derivatives) abrogates ERK activation, suggesting potential inhibition of the Ras/BRAF pathway; however, compounds **3** and **4** (shorter derivatives) do not act in the same way.

## 4. Materials and Methods

### 4.1. Computational Details

#### 4.1.1. Ligand Optimization

All ligands were fully optimized at DFT level using the M06-2X functional with the 6-311+G* basis set as implemented in the Gaussian16 package [35]. Frequency calculations were performed at the same computational level to confirm that the resulting optimized structures were energetic minima. The effect of water solvation was accounted for by using the SCRF-PCM approach implemented in the Gaussian16 package including dispersion, repulsion and cavitation energy terms of the solvent in the optimization.

#### 4.1.2. Docking Experiments

The program AutoDock Vina 4.2 was used to carry out docking studies [36]. The ligands were flexibly docked into the rigid in-house TP-containing BRAF model (see details in ESI). The corresponding docking scores (G-scores) were measured in kcal/mol and are only indicative of the quality of the interaction with the target; they do not provide a quantitative measure of binding. Poses obtained from the docking were visualised with VMD [37].

### 4.2. Chemistry

#### 4.2.1. 1-(4-Fluorophenyl)-2-(3-(4-guanidinophenoxy)phenyl)guanidine dihydrochloride (5)

Following Method A (see ESI), **21** (80 mg, 0.12 mmol) was dissolved in 4 M HCl in dioxane (0.54 mL, 2.16 mmol) and in additional dioxane (0.07 mL) until a final concentration of 0.2 M was reached. After 8 h stirring at 55 °C, the reaction was adjudged complete (TLC), solvents were evaporated and the residue was purified by silica gel (CH_3_Cl:MeOH) chromatography to afford the pure hydrochloride salt as a white-yellow solid (35 mg, 76%). Mp: decomp. > 180 °C. δ_H_ (400 MHz, CD_3_OD): 7.00 (dd, *J* = 8.3, 2.4, 1H, H-4), 7.04 (t, *J* = 2.1 Hz, 1H, H-2), 7.13–7.17 (m, 3H, H-8 and H-8′ and H-6), 7.19–7.24 (m, 2H, H-13 and H-13′), 7.31 (d, *J* = 8.9 Hz, 2H, H-9 and H-9′), 7.36–7.39 (m, 2H, H-12 and H-12′), 7.47 (t, *J* = 8.1 Hz, 1H, H-5). δ_C_ (100 MHz, CD_3_OD): 116.5 (CH Ar, C-2), 117.8 (d, *J* = 23.3 Hz, 2 CH Ar, C-13 and C-13′), 118.6 (CH Ar, C-4), 121.1 (CH Ar, C-6), 121.5 (2 CH Ar, C-8 and C-8′), 129.95 (d, *J* = 8.8 Hz, 2 CH Ar, C-12 and C-12′), 128.96 (2 CH Ar, C-9 and C-9′), 131.6 (qC), 132.3 (d, *J* = 3.1 Hz, qC, C-11), 132.4 (CH Ar, C-5), 138.0 (qC), 156.6 (qC), 157.4 (qC), 158.4 (qC), 159.5 (qC), 163.2 (d, *J* = 246.1 Hz, qC, C-14). δ_F_ (376 MHz, CD_3_OD): −115.93 (m). ν_max_(ATR)/cm^−1^: 3110 (NH), 3052 (NH), 2922, 2330, 2134, 1655 (C=N), 1582 (C=N), 1505 (C-N), 1486, 1404, 1212 (C-O), 1066 (C-F), 834, 792, 552. HRMS (*m/z* ESI^+^): found: 379.1687 (M^+^ + H), C_20_H_20_N_6_OF requires: 379.1683. HPLC: 99.7% (*t*R: 22.9 min).

#### 4.2.2. 1-(3:4-Di-fluorophenyl)-2-(3-(4-guanidinophenoxy)phenyl)guanidine dihydrochloride (6)

Following Method A (see ESI), **22** (53 mg, 0.08 mmol) was dissolved in 4 M HCl in dioxane (0.36 mL, 1.37 mmol) and in additional dioxane (0.06 mL) until a final concentration of 0.2 M was reached. After 8 h stirring at 55 °C, the reaction was adjudged complete (TLC), solvents were evaporated and the residue was purified by silica gel chromatography (CHCl_3_:MeOH) to afford the pure hydrochloride salt as a white solid (34 mg, 90%). Mp: 158–160 °C. δ_H_ (400 MHz, CD_3_OD): 7.00 (dd, *J* = 8.3, 2.4 Hz, 1H, H-4), 7.05 (t, *J* = 2.2 Hz, 1H, H-2), 7.13–7.20 (m, 4H, H-8 and H-8′, H-6 and H-16), 7.32 (d, *J* = 8.9 Hz, 2H, H-9 and H-9′), 7.34–7.41 (m, 2H, H-12 and H-15), 7.47 (t, *J* = 8.1 Hz, 1H, H-5). δ_C_ (100 MHz, CD_3_OD): 116.3 (d, *J* = 19.7 Hz, C-12 or C-15), 116.5 (CH Ar, C-2), 118.6 (CH Ar, C-4), 119.5 (d, *J* = 18.8 Hz, C-12 or C-15), 121.1 (CH Ar, C-6), 121.5 (2 CH Ar, C-8 and C-8′), 123.4 (dd, *J* = 6.7, 3.7 Hz, C-16), 129.0 (2 CH Ar, C-9 and C-9′), 131.6 (qC), 132.4 (CH Ar, C-5), 133.0 (dd, *J* = 8.3, 3.6 Hz, qC, C-11), 137.9 (qC), 150.8 (dd, *J* = 247.8, 12.6 Hz, qC, C-13 or C-14), 151.8 (dd, *J* = 248.7, 13.7 Hz, qC, C-13 or C-14), 156.5 (qC), 157.4 (qC), 158.3 (qC), 159.5 (qC). δ_F_ (376 MHz, CD_3_OD): −137.23 (m), −141.12 (m). ν_max_(ATR)/cm^−1^: 3228 (NH), 3040 (NH), 2923, 2853, 1655 (C=N), 1579 (C=N), 1505, 1485, 1401, 1259, 1211 (C-F), 1149, 972, 825, 771, 694, 649, 609, 587. HRMS (*m/z* ESI^+^): found: 397.1598 (M^+^ + H), C_20_H_19_N_6_OF_2_ requires: 397.1588. HPLC: 96.8% (*t*R: 23.2 min).

#### 4.2.3. 1-(2-Fluoro-4-iodophenyl)-2-(3-(4-guanidinophenoxy)phenyl)guanidine dihydrochloride (7)

Following Method A (see ESI), **23** (357 mg, 0.44 mmol) was dissolved in 4 M HCl in dioxane (2 mL, 7.92 mmol) and in additional dioxane (0.2 mL) until a final concentration of 0.2 M was reached. After 8 h stirring at 55 °C, the reaction was adjudged complete (TLC), solvents were evaporated and the residue was purified by silica gel chromatography (CH_3_Cl:MeOH) to afford the pure hydrochloride salt as a white solid (210 mg, 83%). Mp: decomp. > 180 °C. δ_H_ (400 MHz, CD_3_OD): 6.99–7.01 (m, 2H, H-2 and H-4), 7.11–7.14 (m, 1H, H-6), 7.15 (d, *J* = 8.9 Hz, 2H, H-8 and H-8′), 7.20 (t, *J* = 8.2 Hz, 1H, H-5), 7.33 (d, *J* = 8.9 Hz, 2H, H-9 and H-9′), 7.44–7.49 (m, 1H, H-15), 7.64–7.66 (m, 1H, H-16), 7.69 (dd, *J* = 9.6, 1.8 Hz, 1H, H-13). δ_C_ (100 MHz, CD_3_OD): 93.4 (d, *J* = 7.5 Hz, qC, C-14), 116.4 (CH Ar, C-2), 118.6 (CH Ar, C-4), 121.0 (CH Ar, C-6), 121.5 (2 CH Ar, C-8 and C-8′), 124.1 (d, *J* = 12.4 Hz, qC, C-11), 127.3 (d, *J* = 22.2 Hz, CH Ar, C-13), 128.9 (2 CH Ar, C-9 and C-9′), 130.9 (CH Ar, C-5), 131.5 (qC), 132.4 (CH Ar, C-15), 136.0 (d, *J* = 3.9 Hz, CH Ar, C-16), 137.9 (qC), 156.4 (qC), 158.0 (d, *J* = 254.4 Hz, qC, C-12), 157.3 (qC), 158.3 (qC), 159.5 (qC). δ_F_ (376, CD_3_OD):—121.10 (t, *J* = 8.9 Hz). ν_max_(ATR)/cm^−1^: 3318 (NH), 3098 (NH), 2958, 1661 (C=N), 1620, 1579, 1485, 1214 (C-F), 1149, 625, 609, 576 (C-I), 566. HRMS (*m/z* ESI^+^): found 505.0646 (M^+^ + H), C_20_H_19_N_6_OFI requires: 505.0649. HPLC: 99.2% (*t*R: 25.3 min).

#### 4.2.4. 1-(4-Bromophenyl)-2-(3-(4-guanidinophenoxy)phenyl)guanidine dihydrochloride (8)

Following Method A (see ESI), **24** (212 mg, 0.29 mmol) was dissolved in 4 M HCl in dioxane (1.30 mL, 5.22 mmol) and in additional dioxane (0.12 mL) until a final concentration of 0.2 M was reached. After 8 h stirring at 55 °C, the reaction was adjudged complete (TLC), solvents were evaporated and the residue was purified by flash chromatography to afford the pure hydrochloride salt as a light-yellow solid (128 mg, 87%). Mp: decomp. > 110 °C. δ_H_ (400 MHz, CD_3_OD): 6.99 (dd, *J* = 8.2, 2.3 Hz, 1H, H-4), 7.03 (t, *J* = 2.2 Hz, 1H, H-2), 7.12–7.16 (m, 3H, H-6 and H-8 and H-8′), 7.27 (d, *J* = 8.7 Hz, 2H, H-12 and H-12′ or H-13 and H-13′), 7.31 (d, *J* = 8.8 Hz, 2H, H-9 and H-9′), 7.46 (t, *J* = 8.1 Hz, 1H, H-5), 7.61 (d, *J* = 8.7 Hz, 2H, H-12 and H-12′ or H-13 and H-13′). δ_C_ (100 MHz, CD_3_OD): 116.3 (CH Ar, C-2), 118.5 (CH Ar, C-4), 120.9 (CH Ar, C-6), 121.5 (2 CH Ar, C-8 and C-8′), 121.7 (qC, C-14), 127.9 (2 CH Ar, C-12 and C-12′ or C-13 and C-13′), 128.9 (2 CH Ar, C-9 and C-9′), 131.5 (qC), 132.4 (CH Ar, C-5), 134.1 (CH Ar, C-12 and C-12′ or C-13 and C-13′), 135.8 (qC), 138.0 (qC), 156.2 (qC), 157.4 (qC), 158.3 (qC), 159.5 (qC). ν_max_(ATR)/cm^−1^: 3124 (NH), 3044 (NH), 1655, 1571 (C=N), 1504 (C=N), 1484, 1405, 1213 (C-O), 1070 (C-Br), 1010, 617-567. HRMS (*m/z* APCI^+^): found: 439.0857 (M^+^ + H), C_20_H_20_BrN_6_O requires: 439.0876. HPLC: 99.8% (*t*R: 24.8 min).

#### 4.2.5. 1-(4-Bromo-3-(trifluoromethyl)phenyl)-2-(3-(4-guanidinophenoxy)phenyl)guanidine dihydrochloride (9)

Following Method A (see ESI), **25** (566 mg, 0.70 mmol) was dissolved in 4 M HCl in dioxane (3.15 mL, 12.6 mmol) and in additional dioxane (0.35 mL) until a final concentration of 0.2 M was reached. After 8 h stirring at 55 °C, the reaction was adjudged complete (TLC), solvents were evaporated and the residue was purified by silica gel chromatography (CHCl_3_:MeOH) to afford the pure hydrochloride salt as a white solid (361 mg, 89%). Mp: decomp. >136 °C. δ_H_ (400 MHz, CD_3_OD): 6.99 (dd, *J* = 8.0, 1.9 Hz, 1H, H-4), 7.06 (t, *J* = 2.1 Hz, 1H, H-2), 7.14–7.16 (m, 3H, H-8 and H-8′ and H-6), 7.32 (d, *J* = 8.9 Hz, 2H, H-9 and H-9′), 7.46 (t, *J* = 8.1 Hz, 1H, H-5), 7.51 (dd, *J* = 8.6, 2.4 Hz, 1H, H-16), 7.74 (d, *J* = 2.4 Hz, 1H, H-12), 7.88 (d, *J* = 8.6 Hz, 1H, H-15). δ_C_ (100 MHz, CD_3_OD): 116.2 (CH Ar, C-2), 118.3 (qC, C-14), 118.5 (CH Ar, C-4), 120.8 (CH Ar, C-6), 121.5 (2 CH Ar, C-8 and C-8′), 123.9 (d, *J* = 260.3 Hz, qCF_3_), 125.3 (m, CH Ar, C-12), 128.9 (2 CH Ar, C-9 and C-9′), 130.5 (CH Ar, C-16), 131.6 (qC), 132.3 (d, *J* = 31.7 Hz, qC, C-13), 132.4 (CH Ar, C-5), 136.9 (qC), 137.8 (CH Ar, C-15), 138.0 (qC), 156.1 (qC), 157.3 (qC), 158.3 (qC), 159.6 (qC). δ_F_ (376 MHz, CD_3_OD):—64.73 (s). ν_max_(ATR)/cm^−1^: 3119 (NH), 3053 (NH), 1663 (C=O), 1584 (C=N), 1478, 1412, 1320 (C-F), 1238, 1214, 1174, 1129 (CF_3_), 1099 (C-Br), 1023, 828, 581—558. HRMS (*m/z* ESI^+^): found 507.0766 (M^+^ + H), C_21_H_19_N_6_OF_3_Br requires: 507.0756. HPLC: 99.9% (*t*R: 26.3 min).

#### 4.2.6. 1-(2-Fluoro-4-iodophenyl)-2-(4-(3-guanidinophenoxy)phenyl)guanidine dihydrochloride (10)

Following Method A (see ESI), **26** (310 mg, 0.39 mmol) was dissolved in 4 M HCl in dioxane (1.73 mL, 6.93 mmol) and in additional dioxane (0.17 mL) until a final concentration of 0.2 M was reached. After 8 h stirring at 55 °C, the reaction was adjudged complete (TLC), solvents were evaporated and the residue was purified by flash chromatography to afford the pure hydrochloride salt as a white solid (198 mg, 88%). Mp: decomp. >150 °C. δ_H_ (400 MHz, CD_3_OD): 6.97 (t, *J* = 2.1 Hz, 1H, H-2), 7.00 (dd, *J* = 8.2, 2.3 Hz, 1H, H-4), 7.08 (dd, *J* = 7.6, 1.5 Hz, 1H, H-6), 7.16 (d, *J* = 8.8 Hz, 2H, H-8 and H-8′), 7.22 (t, *J* = 8.2 Hz, 1H, H-5 or H-15), 7.36 (d, *J* = 8.8 Hz, 2H, H-9 and H-9′), 7.47 (t, *J* = 8.1 Hz, 1H, H-5 or H-15), 7.66 (d, *J* = 9.1 Hz, 1H, H-16), 7.70 (dd, *J* = 9.6, 1.7 Hz, 1H, H-13). δ_C_ (100 MHz, CD_3_OD): 93.5 (d, *J* = 7.5 Hz, qC, C-14), 116.6 (CH Ar, C-2), 118.6 (CH Ar, C-4), 121.3 (CH Ar, C-6), 121.6 (2 CH Ar, C-8 and C-8′), 124.0 (d, *J* = 12.5 Hz, qC, C-11), 127.3 (d, *J* = 22.2 Hz, C-13), 128.7 (2 CH Ar, C-9 and C-9′), 131.1 (CH Ar, C-5 or C-15), 131.6 (qC), 132.4 (CH Ar, C-5 or C-15), 136.0 (d, *J* = 3.9 Hz, C-16), 137.7 (qC), 156.8 (qC), 157.3 (qC), 157.9 (qC), 158.2 (d, *J* = 254.5 Hz, qC, C-12), 159.5 (qC). δ_F_ (376 MHz, CD_3_OD): −121.04 (t, *J* = 8.8 Hz). ν_max_(ATR)/cm^−1^: 3335 (NH), 3265 (NH), 3180 (NH), 3052, 2868, 2325, 1616 (C=N), 1560 (C=N), 1504, 1400, 1226 (C-F), 1162, 1109, 971, 875, 789, 684—573 (C-I). HRMS (*m/z* ESI^+^): found: 505.0645 (M^+^ + H), C_20_H_19_N_6_OFI requires: 505.0649. HPLC: 99.9% (*t*R: 25.9 min).

#### 4.2.7. 1-(4-Bromo-3-(trifluoromethyl)phenyl)-2-(4-(3-guanidinophenoxy)phenyl)guanidine dihydrochloride (11)

Following Method A (see ESI), 27 (148 mg, 0.18 mmol) was dissolved in 4 M HCl in dioxane (0.81 mL, 3.24 mmol) and in additional dioxane (0.10 mL) until a final concentration of 0.2 M was reached. After 8 h stirring at 55 °C, the reaction was adjudged complete (TLC), solvents were evaporated and the residue was purified by flash chromatography to afford the pure hydrochloride salt as a white solid (100 mg, 94%). Mp: decomp. > 95 °C. δ_H_ (400 MHz, CD_3_OD): 6.98–7.02 (m, 2H, H-2 and H-4), 7.09 (ddd, 1H, *J* = 8.0, 1.9, 0.9 Hz, H-6), 7.17 (d, *J* = 8.9 Hz, 2H, H-8 and H-8′), 7.41 (d, *J* = 9.0 Hz, 2H, H-9 and H-9′), 7.49 (t, *J* = 8.0 Hz, 1H, H-5), 7.55 (dd, *J* = 8.5, 2.6 Hz, 1H, H-16), 7.77 (d, *J* = 2.6 Hz, 1H, H-12), 7.91 (d, *J* = 8.6 Hz, 1H, H-15). δ_C_ (100 MHz, CD_3_OD): 116.6 (CH Ar, C-2 or C-4), 118.5 (qC, C-14), 118.6 (CH Ar, C-2 or C-4), 121.3 (CH Ar, C-6), 121.6 (2 CH Ar, C-8 and C-8′), 123.9 (d, *J* = 272.8 Hz, qCF_3_), 125.5 (q, *J* = 5.6 Hz, CH Ar, C-12), 128.5 (2 CH Ar, C-9 and C-9′), 130.8 (CH Ar, C-16), 131.7 (qC), 132.3 (q, *J* = 31.6 Hz, qC, C-13), 132.4 (CH Ar, C-5), 136.7 (qC), 137.7 (qC), 137.8 (CH Ar, C-15), 156.5 (qC), 157.3 (qC), 157.9 (qC), 159.6 (qC). δ_F_ (376 MHz, CD_3_OD):—64.30 (s). ν_max_(ATR)/cm^−1^: 3309 (NH), 3116 (NH), 3053 (NH), 2837, 2280, 1663 (C=N), 1577 (C=N), 1505, 1486, 1405, 1320, 1258 (C-O), 1214 (CF_3_), 1129, 1023 (C-Br), 829, 595—575. HRMS (*m/z* ESI^+^): found: 507.0750 (M^+^ + H), C_21_H_19_N_6_OBrF_3_ requires: 507.0756. HPLC: 99.9% (*t*R: 26.6 min).

#### 4.2.8. 1-(4-(3-((4-Chlorophenyl)amino)phenoxy)phenyl)guanidine hydrochloride (28)

Following Method A (see ESI), **44** (97 mg, 0.18 mmol) was dissolved in 4 M HCl in dioxane (0.53 mL, 2.10 mmol) and in additional dioxane (0.9 mL) until a final concentration of 0.2 M was reached. After 8 h stirring at 55 °C, the reaction was adjudged complete (TLC), solvents were evaporated and the residue was purified by silica gel chromatography (CHCl_3_:MeOH) to afford the pure hydrochloride salt as a purple solid (69 mg, 99%). Mp: 50–52 °C. δ_H_ (400 MHz, CD_3_OD): 6.51 (dd, *J* = 8.3, 1.9 Hz, 1H, H-4), 6.73 (t, *J* = 2.2 Hz, 1H, H-2), 6.85 (dd, *J* = 7.8, 1.8 Hz, 1H, H-6), 7.05 (d, *J* = 8.9 Hz, 2H, H-12 and H-12′), 7.08 (d, *J* = 8.9 Hz, 2H, H-8 and H-8′), 7.18 (d, *J* = 8.9 Hz, 2H, H-13 and H-13′), 7.22 (t, *J* = 8.2 Hz, 1H, H-5), 7.27 (d, *J* = 8.9 Hz, 2H, H-9 and H-9′). δ_C_ (100 MHz, CD_3_OD): 108.6 (CH Ar, C-2), 111.8 (CH Ar, C-4), 113.7 (CH Ar, C-6), 120.0 (2 CH Ar, C-12 and C-12′), 120.7 (2 CH Ar, C-8 and C-8′), 126.1 (qC, C-14), 128.8 (2 CH Ar, C-9 and C-9′), 130.1 (2 CH Ar, C-13 and C-13′), 130.6 (qC), 131.5 (CH Ar, C-5), 143.4 (qC), 146.7 (qC), 158.4 (qC), 158.5 (qC), 158.9 (qC). ν_max_(ATR)/cm^−1^: 3297 (N-H), 3126 (N-H), 1688, 1586 (C=N), 1502, 1485 (C-N), 1325, 1216 (C-O), 1142 (C-Cl), 997, 972, 823, 770, 689, 604, 588, 570. HRMS (*m/z* ESI^+^): found 353.1177 (M^+^ + H. C_19_H_18_N_4_OCl requires: 353.1169). HPLC: 98.0% (*t*R: 32.3 min).

#### 4.2.9. 1-(4-(3-((4-Chloro-3-(trifluoromethyl)phenyl)amino)phenoxy)phenyl)guanidine hydrochloride (3)

Following Method A (see ESI), **45** (112 mg, 0.18 mmol) was dissolved in 4 M HCl in dioxane (0.54 mL, 2.16 mmol) and in additional dioxane (0.36 mL) until a final concentration of 0.2 M was reached. After 8 h stirring at 55 °C, the reaction was adjudged complete (TLC), solvents were evaporated and the residue was purified by flash chromatography to afford the pure hydrochloride salt as a light brown solid (59 mg, 80%). Mp: 58–60 °C. δ_H_ (400 MHz, CD_3_OD): 6.62 (dd, *J* = 8.7, 2.3 Hz, 1H, H-4), 6.78 (t, *J* = 2.2 Hz, 1H, H-2), 6.90 (dd, *J* = 8.1, 2.1 Hz, 1H, H-6), 7.11 (d, *J* = 8.9 Hz, 2H, H-8 and H-8’), 7.24 (dd, *J* = 8.8, 2.8 Hz, 1H, H-16), 7.27–7.31 (m, 3H, H-9, H-9’ and H-5) 7.36–7.39 (m, 2H, H-12, H-15). δ_C_ (100 MHz, CD_3_OD): 109.8 (CH Ar, C-2), 113.1 (CH Ar, C-4), 114.8 (CH Ar, C-6), 116.1 (q, *J* = 5.6 Hz, CH Ar, C-12), 121.0 (2 CH Ar, C-8 and C-8’), 121.5 (CH Ar, C-16), 122.1 (qC, C-14), 124.4 (d, *J* = 272.5 Hz, qCF_3_), 129.0 (2 CH Ar, C-9 and C-9’), 129.6 (q, *J* = 31.0, qC, C-13), 131.0 (qC), 131.8 (CH Ar, C-5), 133.4 (CH Ar, C-15), 144.4 (qC), 145.3 (qC), 158.2 (qC), 158.4 (qC), 159.2 (qC). δ_F_ (376 MHz, CD_3_OD):—64.18 (s). ν_max_(ATR)/cm^−1^: 3295 (NH), 3163, 2923, 2853, 2400, 1664 (C=O), 1595 (C=N), 1504, 1482, 1441, 1333, 1258, 1217 (CF_3_), 1127, 1112 (C-Cl), 1027, 999, 977, 825. HRMS (*m/z* ESI^+^): found 421.1044 (M^+^ + H. C_20_H_17_ClF_3_N_4_O requires: 421.1043). HPLC: 97.8% (*t*R: 32.9 min).

#### 4.2.10. 1-(4-(3-((3-(Trifluoromethyl)phenyl)amino)phenoxy)phenyl)guanidine hydrochloride (29)

Following Method A (see ESI), **46** (371 mg, 0.63 mmol) was dissolved in 4 M HCl in dioxane (1.90 mL, 7.56 mmol) and in additional dioxane (1.25 mL) until a final concentration of 0.2 M was reached. After 6 h stirring at 55 °C, the reaction was adjudged complete (TLC), solvents were evaporated and the residue was purified by flash chromatography to afford the pure hydrochloride salt as a white solid (242 mg, 90%). Mp: 93–95 °C. δ_H_ (400 MHz, CD_3_OD): 6.59 (dd, *J* = 8.1, 2.3 Hz, 1H, H-4), 6.78 (t, *J* = 2.2 Hz, 1H, H-2), 6.90 (dd, *J* = 7.9, 1.9 Hz, 1H, H-6), 7.07–7.13 (m, 3H, H-8 and 8′ and H-12 or H-14), 7.26–7.30 (m, 5H, H-9 and 9′, H-12 or H-14, H-16 and H-5 or H-15), 7.37 (t, *J* = 8.3 Hz, 1H, H-5 or H-15). δ_C_ (100 MHz, CD_3_OD): 109.4 (CH Ar, C-2), 112.6 (CH Ar, C-4), 113.9 (q, *J* = 4.0 Hz, CH Ar, C-12 or C-14), 114.6 (CH Ar, C-6), 117.3 (q, *J* = 4.0 Hz, CH Ar, C-12 or C-14), 119.9 (d, *J* = 280.7 Hz, qCF_3_), 120.9 (2 CH Ar, C-8 and C-8′), 121.2 (CH Ar, C-16), 128.9 (2 CH Ar, C-9 and C-9′), 130.8 (qC), 131.1 (CH Ar, C-5 or C-15), 131.7 (CH Ar, C-5 or C-15), 132.6 (d, *J* = 31.9 Hz, qC, C-13), 145.7 (qC), 145.9 (qC), 158.3 (qC), 158.4 (qC), 159.1 (qC). δ_F_ (376 MHz, CD_3_OD): −64.42 (s). ν_max_(ATR)/cm^−1^: 3301 (NH), 3135 (NH), 1665, 1587 (C=N), 1490, 1486, 1335 (C-N), 1216 (C-O), 1161, 1116 (CF_3_), 1067 (C-Cl), 976, 836, 785, 689. HRMS (*m/z* ESI^+^): found 387.1438 (M^+^ + H. C_20_H_18_N_4_OF_3_ requires: 387.1433). HPLC: 97.5% (*t*R: 31.7 min).

#### 4.2.11. 1-(4-(3-((3-(Pentafluorosulfanyl)phenyl)amino)phenoxy)phenyl)guanidine hydrochloride (30)

Following Method A (see ESI), **47** (272 mg, 0.42 mmol) was dissolved in 4 M HCl in dioxane (1.27 mL, 5.06 mmol) and in additional dioxane (0.83 mL) until a final concentration of 0.2 M was reached. After 6 h stirring at 55 °C, the reaction was adjudged complete (TLC), solvents were evaporated and the residue was purified by silica gel chromatography (CHCl_3_:MeOH) to afford the pure hydrochloride salt as an orange solid (127 mg, 62%). Mp: 104–106 °C. δ_H_ (400 MHz, CD_3_OD): 6.61 (dd, *J* = 8.2, 2.3 Hz, 1H, H-4), 6.78 (t, *J* = 2.2. Hz, 1H, H-2), 6.90 (dd, *J* = 8.1, 2.1 Hz, 1H, H-6), 7.11 (d, *J* = 8.9 Hz, H-8 and H-8′), 7.22–7.31 (m, 5H, H-9 and H-9′, H-5 or H-15, H-14 and H-16), 7.34–7.38 (m, 1H, H-5 or H-15), 7.44 (t, *J* = 2.2 Hz, 1H, H-12). δ_C_ (100 MHz, CD_3_OD): 109.6 (CH Ar, C-2), 112.9 (CH Ar, C-4), 114.6 (CH Ar, C-6), 114.9 (p, *J* = 4.6 Hz, CH Ar, C-12), 118.0 (p, *J* = 4.7 Hz, CH Ar, C-14), 120.8 (CH Ar, C-16), 120.9 (2 CH Ar, C-8 and C-8′), 128.8 (2 CH Ar, C-9 and C-9′), 130.6 (CH Ar, C-5 or C-15), 130.8 (qC), 131.8 (CH Ar, C-5 or C-15), 145.6 (qC), 145.7 (qC), 155.9 (p, *J* = 16.4 Hz, qC, C-13), 158.2 (qC), 158.3 (qC), 159.2 (qC). δ_F_ (376 MHz, CD_3_OD): −64.34 (s). ν_max_(ATR)/cm^−1^: 3273 (NH), 3150 (NH), 1669, 1593 (C=N), 1487 (C-N), 1218 (C-O), 834 (SF_5_), 567. HRMS (*m/z* ESI^+^): found 445.1124 (M^+^ + H. C_19_H_18_N_4_OSF_5_ requires: 445.1121). HPLC: 95.4% (*t*R: 32.3 min).

#### 4.2.12. 1-(4-(3-((4-Chloro-3-(trifluormethyl)phenyl)amino)-5-fluorophenoxy)phenyl)guanidine hydrochloride (36)

Following Method A (see ESI), **48** (166 mg, 0.26 mmol) was dissolved in 4 M HCl in dioxane (0.78 mL, 3.12 mmol) and in additional dioxane (0.51 mL) until a final concentration of 0.2 M was reached. After 8 h stirring at 55 °C, the reaction was adjudged complete (TLC), solvents were evaporated and the residue was purified by silica gel chromatography (CHCl_3_:MeOH) to afford the pure hydrochloride salt as a light brown solid (114 mg, 92%). Mp: 92–94 °C. δ_H_ (600 MHz, CD_3_OD): 6.31 (dt, *J* = 9.9, 2.1 Hz, 1H, H-4), 6.55 (s, 1H, H-2), 6.58 (dt, *J* = 10.7, 2.0 Hz, 1H, H-6), 7.16 (d, *J* = 8.8 Hz, 2H, H-8 and H-8′), 7.29 (dd, *J* = 8.8, 2.7 Hz, 1H, H-16), 7.32 (d, *J* = 8.8 Hz, 2H, H-9 and H-9′), 7.40 (d, *J* = 2.6 Hz, 1H, H-12), 7.43 (d, *J* = 8.7 Hz, 1H, H-15). δ_C_ (150 MHz, CD_3_OD): 99.4 (d, *J* = 25.7 Hz, CH Ar, C-4), 100.3 (d, *J* = 25.5 Hz, CH Ar, C-6), 103.8 (d, *J* = 2.6 Hz, CH Ar, C-2), 117.4 (q, *J* = 5.5 Hz, CH Ar, C-12), 121.8 (2 CH Ar, C-8 and C-8′), 122.7 (CH Ar, C-16), 123.4 (qC, C-14), 124.3 (d, *J* = 272.2 Hz, qCF_3_), 128.9 (2 CH Ar, C-9 and C-9′), 129.8 (q, *J* = 31.0, qC, C-13), 131.7 (qC), 133.5 (CH Ar, C-15), 143.4 (qC), 146.7 (d, *J* = 13.2 Hz, qC, C-1 or C-3), 157.1 (qC), 158.4 (qC), 160.7 (d, *J* = 13.7 Hz, qC, C-1 or C-3), 165.8 (d, *J* = 243.4 Hz, qC, C-5). δ_F_ (376 MHz, CD_3_OD): −64.67 (s), −112.53 (s). ν_max_(ATR)/cm^−1^: 3285 (NH), 3139 (NH), 1666, 1601 (C=N), 1504, 1476, 1323 (CF_3_), 1216 (C-O), 1112 (C-F), 1020 (C-Cl), 994, 823, 660. HRMS (*m/z* ESI^+^): found 439.0945 (M^+^ + H. C_20_H_16_N_4_OF_4_Cl requires: 439.0943). HPLC: 95.7% (*t*R: 33.1 min).

#### 4.2.13. 1-(3,4-Di-fluorophenyl)-3-(3-((6-guanidinopyridin-3-yl)oxy)phenyl)guanidine dihydrochloride (49)

Following Method A (see ESI), **61** (176 mg, 0.25 mmol) was dissolved in 4 M HCl in dioxane (1.14 mL, 4.54 mmol) and in additional dioxane (0.11 mL) until a final concentration of 0.2 M was reached. After 8 h stirring at 55 °C, the reaction was adjudged complete (TLC), solvents were evaporated and the residue was purified by silica gel chromatography (CHCl_3_:MeOH) to afford the pure hydrochloride salt as a white solid (101 mg, 84%). Mp: decomp. above 110 °C. δ_H_ (400 MHz, CD_3_OD): 6.62 (dd, *J* = 7.5, 2.4 Hz, 1H, H-4), 6.77–6.81 (m, 1H, H-17), 6.93 (dd, *J* = 8.0, 1.8 Hz, 1H, H-6), 6.99 (d, *J* = 8.9 Hz, 1H, H-9), 7.00–7.13 (m, 3H, H-2, H-13 and H-16), 7.25 (t, *J* = 8.1 Hz, 1H, H-5), 7.49 (dd, *J* = 8.9, 2.9 Hz, 1H, H-8), 8.05 (d, *J* = 2.9 Hz, 1H, H-11). δ_C_ (100 MHz, CD_3_OD): 112.0 (d, *J* = 18.8 Hz, CH Ar, C-13 or C-16), 112.4 (CH Ar, C-2), 113.0 (CH Ar, C-4), 116.4 (CH Ar, C-9), 118.0 (CH Ar, C-6), 118.1 (d, *J* = 17.9 Hz, CH Ar, C-13 or C-16), 119.1 (dd, *J* = 5.6, 3.1 Hz, CH Ar, C-17), 131.3 (CH Ar, C-5), 131.4 (CH Ar, C-8), 138.5 (CH Ar, C-11), 143.9 (dd, *J* = 7.4, 2.2 Hz, qC, C-12), 146.6 (qC), 147.2 (dd, *J* = 240.4, 12.9 Hz, qC, C-14 or C-15), 150.1 (qC), 151.1 (qC), 151.4 (dd, *J* = 245.1, 13.4 Hz, qC, C-14 or C-15), 152.5 (qC), 157.4 (qC), 159.0 (qC). δ_F_ (376 MHz, CD_3_OD): −139.72 (m), −149.19 (m). ν_max_(ATR)/cm^−1^: 3313 (NH), 3154 (NH), 2922, 2861, 1682, 1625 (C=N), 1507, 1473 (C-F), 1375, 1228 (C-F), 1166, 1146 (C-O), 866, 830, 770, 570, 557. HRMS (*m/z* ESI^+^): found 398.1548 (M^+^ + H. C_19_H_18_N_7_OF_2_ requires: 398.1541). HPLC: 99.8% (*t*R: 23.6 min).

#### 4.2.14. 1-(2-Fluoro-4-iodophenyl)-3-(3-((6-guanidinopyridin-3-yl)oxy)phenyl)guanidine dihydrochloride (50)

Following Method A (see ESI), **62** (104 mg, 0.13 mmol) was dissolved in 4 M HCl in dioxane (0.58 mL, 2.32 mmol) and in additional dioxane (0.10 mL) until a final concentration of 0.2 M was reached. After 8 h stirring at 55 °C, the reaction was adjudged complete (TLC), solvents were evaporated and the residue was purified by silica gel chromatography (CHCl_3_:MeOH) to afford the pure hydrochloride salt as a white solid (67 mg, 89%). Mp: decomp. above 120 °C. δ_H_ (400 MHz, CD_3_OD): 6.98 (ddd, *J* = 8.3, 2.4, 0.7 Hz, 1H, H-4), 7.02 (t, *J* = 2.2 Hz, 1H, H-2), 7.11–7.18 (m, 3H, H-5 or H-16, H-6 and H-9), 7.46 (t, *J* = 8.1 Hz, 1H, H-5 or H-16), 7.61–7.64 (m, 2H, H-17 and H-8), 7.67 (dd, *J* = 9.7, 1.8 Hz, 1H, H-14), 8.16 (d, *J* = 2.9 Hz, 1H, H-11). δ_C_ (100 MHz, CD_3_OD): 92.7 (qC, C-15), 115.5 (CH Ar, C-2), 115.6 (CH Ar, C-9), 117.6 (CH Ar, C-4), 120.9 (CH Ar, C-6), 127.2 (d, *J* = 22.2 Hz, CH Ar, C-14), 127.3 (d, *J* = 14.1 Hz, qC, C-12), 130.6 (CH Ar, C-5 or C-16), 132.1 (CH Ar, C-8), 132.5 (CH Ar, C-5 or C-16), 136.0 (CH Ar, *J* = 3.9 Hz, C-17), 138.7 (qC), 139.1 (CH Ar, C-11), 149.2 (qC), 151.2 (qC), 156.2 (qC), 156.9 (qC), 157.9 (qAr, *J* = 253.9 Hz, C-13), 159.4 (qC). δ_F_ (376 MHz CD_3_OD):—121.55 (t, *J* = 8.3 Hz). ν_max_(ATR)/cm^−1^: 3277 (NH), 3122 (NH), 2923, 2849, 1680, 1660, 1623–1570 (C=N), 1474 (C-F), 1227 (C-O), 1160, 1026, 945, 600 (C-I). HRMS (*m/z* ESI^+^): found 506.0609 (M^+^ + H. C_19_H_18_N_7_OFI requires: 506.0602). HPLC: 98.1% (*t*R: 25.7 min).

#### 4.2.15. 1-(4-Bromophenyl)-3-(3-((6-guanidinopyridin-3-yl)oxy)phenyl)guanidine dihydrochloride (51)

Following Method A (see ESI), **63** (184 mg, 0.24 mmol) was dissolved in 4 M HCl in dioxane (1.08 mL, 4.4 mmol) and in additional dioxane (0.12 mL) until a final concentration of 0.2 M was reached. After 8 h stirring at 55 °C, the reaction was adjudged complete (TLC), solvents were evaporated and the residue was purified by silica gel chromatography (CHCl_3_:MeOH) to afford the pure hydrochloride salt as a white solid (113 mg, 92%). Mp: decomp. above 124 °C. δ_H_ (400 MHz, CD_3_OD): 6.99–7.01 (m, 1H, H-4), 7.04 (bs, 1H, H-2), 7.14–7.16 (m, 2H, H-6 and H-9), 7.28 (d, *J* = 8.5 Hz, 2H, H-13 and H-13′ or H-14 and H-14′), 7.48 (t, *J* = 8.1 Hz, 1H, H-5), 7.60–7.63 (m, 3H, H-13 and H-13′ or H-14 and H-14′and H-8), 8.16 (d, *J* = 2.6 Hz, 1H, H-11). δ_C_ (100 MHz, CD_3_OD): 115.6 (CH Ar, C-6 or C-9), 115.8 (CH Ar, C-2), 118.0 (CH Ar, C-4), 121.1 (CH Ar, C-6 or C-9), 121.7 (qC, C-15), 127.9 (CH Ar, C-13 and C-13′ or C-14 and C-14′), 132.1 (CH Ar, C-8), 132.5 (CH Ar, C-5), 134.1 (CH Ar, C-13 and C-13′ or C-14 and C-14′), 135.7 (qC), 138.2 (qC), 139.0 (CH Ar, C-11), 149.2 (qC), 151.2 (qC), 156.1 (qC), 156.8 (qC), 159.4 (qC). ν_max_(ATR)/cm^−1^: 3256 (NH), 3114 (NH), 2971, 1680, 1660, 1619 (C=N), 1566 (C=N), 1474, 1376 (C-N), 1226 (C-O), 1069 (C-Br), 1010, 832, 637—584. HRMS (*m/z* ESI^+^): found 440.0837 (M^+^ + H. C_19_H_19_N_7_OBr requires: 440.0834). HPLC: 97.5% (*t*R: 25.2 min).

#### 4.2.16. 1-(4-Bromo-3-(trifluoromethyl)phenyl)-3-(3-((6-guanidinopyridin-3-yl)oxy)phenyl) guanidine dihydrochloride (52)

Following Method A (see ESI), **64** (358 mg, 0.44 mmol) was dissolved in 4 M HCl in dioxane (2 mL, 7.97 mmol) and in additional dioxane (0.2 mL) until a final concentration of 0.2 M was reached. After 8 h stirring at 55 °C, the reaction was adjudged complete (TLC), solvents were evaporated and the residue was purified by silica gel chromatography (CHCl_3_:MeOH) to afford the pure hydrochloride salt as a white solid (225 mg, 88%). Mp: decomp. above 170 °C. δ_H_ (400 MHz, CD_3_OD): 6.66 (dd, *J* = 8.1, 2.4 Hz, 1H, H-4), 6.96 (dd, *J* = 8.0, 1.9 Hz, 1H, H-6), 7.04 (d, *J* = 8.9 Hz, 1H, H-9), 7.08 (t, *J* = 2.1 Hz, 1H, H-2), 7.20 (dd, *J* = 8.6, 2.5 Hz, 1H, H-17), 7.28 (t, *J* = 8.1 Hz, 1H, H-5), 7.48 (d, *J* = 2.5, 1H, H-13), 7.56 (dd, *J* = 8.9, 2.9 Hz, 1H, H-8), 7.60 (d, *J* = 8.6 Hz, 1H, H-16), 8.10 (d, *J* = 2.7 Hz, 1H, H-11). δ_C_ (100 MHz, CD_3_OD): 111.8 (d, *J* = 1.8 Hz, qC, C-15), 112.4 (CH Ar, C-2), 113.3 (CH Ar, C-4), 115.5 (CH Ar, C-9), 117.8 (CH Ar, C-6), 122.4 (q, *J* = 5.5 Hz, CH Ar, C-13), 124.4 (d, *J* = 272.6 Hz, qCF_3_), 127.7 (CH Ar, C-17), 130.9 (d, *J* = 30.8 Hz, qC, C-14), 131.4 (CH Ar, C-5), 131.6 (CH Ar, C-8), 136.6 (CH Ar, C-16), 138.6 (CH Ar, C-11), 146.1 (qC), 147.2 (qC), 148.9 (qC), 151.8 (qC), 152.4 (qC), 156.9 (qC), 159.0 (qC). δ_F_ (376 MHz, CD_3_OD):—63.96 (s). ν_max_(ATR)/cm^−1^: 3281 (NH), 3142 (NH), 2922, 2849, 1680, 1625 (C=N), 1566 (C=N), 1473, 1319, 1227 (CF_3_), 1129 (C-Br), 1023, 832, 592-583. HRMS (*m/z* ESI^+^): found 508.0710 (M^+^ + H. C_20_H_18_N_7_OF_3_Br requires: 508.0708). HPLC: 99.8% (*t*R: 27.5 min).

#### 4.2.17. 1-(4-Chloro-3-(trifluoromethyl)phenyl)-3-(3-((6-guanidinopyridin-3-yl)oxy)phenyl) guanidine dihydrochloride (2)

Following Method A (see ESI), **65** (113 mg, 0.15 mmol) was dissolved in 4M HCl in dioxane (0.67 mL, 2.70 mmol) and in additional dioxane (0.10 mL) until a final concentration of 0.2M was reached. After 6 h stirring at 55 °C, the reaction was adjudged complete (TLC), solvents were evaporated and the residue was purified by flash chromatography to afford the pure hydrochloride salt as a white solid (63 mg, 79%). Mp: 169–171 °C. δ_H_ (400 MHz, CD_3_OD): 6.93 (dd, *J* = 8.3, 2.3 Hz, 1H, H-4), 7.06 (t, *J* = 2.1 Hz, 1H, H-2), 7.11–7.13 (m, 2H, H-6 and H-9), 7.44 (t, *J* = 8.2 Hz, 1H, H-5), 7.53 (dd, *J* = 8.6, 2.5 Hz, 1H, H-17), 7.60–7.65 (m, 2H, H-16 and H-8), 7.69 (d, *J* = 2.4 Hz, 1H, H-13), 8.15 (d, *J* = 2.9 Hz, 1H, H-11). δ_C_ (100 MHz, CD_3_OD): 115.1 (CH Ar, C-2), 115.6 (CH Ar, C-9), 117.1 (CH Ar, C-4), 120.5 (CH Ar, C-6), 123.9 (d, *J* = 272.5 Hz, qCF_3_) 124.5 (q, *J* = 5.5 Hz, CH Ar, C-13), 129.9 (qC, C-15), 130.1 (CH Ar, C-17), 130.2 (d, *J* = 34.7 Hz, qC, C-14), 132.0 (CH Ar, C-8 or C-16), 132.3 (CH Ar, C-5), 134.0 (CH Ar, C-8 or C-16), 138.3 (qC), 139.0 (CH Ar, C-11), 139.7 (qC), 149.1 (qC), 151.3 (qC), 155.5 (qC), 156.9 (qC), 159.3 (qC). δ_F_ (376 MHz, CD_3_OD):—64.24 (s). ν_max_(ATR)/cm^−1^: 3297 (NH), 3121 (NH), 2923, 2854, 1625 (C=N), 1581 (C=N), 1474 (CF_3_), 1320, 1227 (C-O), 1130 (C-Cl), 1032, 832, 589, 557. HRMS (*m/z* ESI^+^): found: 464.1222 (M^+^ + H. C_20_H_18_N_7_OF_3_Cl requires: 464.1213). HPLC: 96.9% (*t*R: 26.8 min).

#### 4.2.18. 1-(5-(3-((4-Chloro-3-(trifluoromethyl)phenyl)amino)phenoxy)pyridin-2-yl)guanidine hydrochloride (4)

Following Method A (see ESI), **69** (200 mg, 0.32 mmol) was dissolved in 4 M HCl in dioxane (0.96 mL, 3.86 mmol) and in additional dioxane (0.65 mL) until a final concentration of 0.2 M was reached. After 8 h stirring at 55 °C, the reaction was adjudged complete (TLC), solvents were evaporated and the residue was purified by silica gel chromatography (CHCl_3_:MeOH) to afford the pure hydrochloride salt as a white solid (136 mg, 93%). Mp: 89–91 °C. δ_H_ (400 MHz, CD_3_OD): 6.60 (dd, *J* = 8.2, 2.3 Hz, 1H, H-4), 6.75 (t, *J* = 2.2 Hz,1H, H-2), 6.90 (dd, *J* = 8.1, 2.0 Hz, 1H, H-6), 7.08 (d, *J* = 8.9 Hz, 1H, H-9), 7.24 (dd, *J* = 8.7, 2.7 Hz, 1H, H-17), 7.29 (t, *J* = 8.1 Hz, 1H, H-5), 7.37–7.39 (m, 2H, H-13 and H-16), 7.59 (dd, *J* = 8.9, 2.9 Hz, 1H, H-8), 8.13 (d, *J* = 2.9 Hz, 1H, H-11). δ_C_ (100 MHz, CD_3_OD): 108.7 (CH Ar, C-2), 112.2 (CH Ar, C-4), 114.7 (CH Ar, C-6), 115.5 (CH Ar, C-9), 116.2 (q, *J* = 5.5 Hz, CH Ar, C-13), 121.8 (CH Ar, C-17), 122.4 (qC, C-15), 124.3 (d, *J* = 272.4 Hz, qCF_3_), 129.6 (d, *J* = 31.0 Hz, qC, C-14), 131.8 (CH Ar, C-8), 132.0 (CH Ar, C-5), 133.4 (CH Ar, C-16), 138.7 (CH Ar, C-11), 144.2 (qC), 145.6 (qC), 148.8 (qC), 151.8 (qC), 156.9 (qC), 159.4 (qC). δ_F_ (376 MHz, CD_3_OD):—64.15 (s). ν_max_(ATR)/cm^−1^: 3264 (NH), 2923, 2863, 1684, 1629, 1595, 1474 (C=N), 1400, 1332, 1229 (C-O), 1129 (CF_3_), 1115 (C-Cl), 977, 998. HRMS (*m/z* ESI^+^): found 422.0987 (M^+^ + H. C_19_H_16_N_5_OClF_3_ requires: 422.0995). HPLC: 98.6% (*t*R: 33.3 min).

#### 4.2.19. 4-(3-((4-Chloro-3-(trifluoromethyl)phenyl)amino)phenoxy)phenyl carbamimidate hydrochloride (70)

To a stirred solution of **74** (205 mg, 0.33 mmol, 1eq) in EtOAc was added SnCl_4_ (0.15 mL, 1.32 mmol, 4 eq). After 2 h of stirring at room temperature, the solvent and the excess of SnCl_4_ were evaporated in vacuo. The remaining liquid was purified by silica gel chromatography(CHCl_3_:Acetone) to afford the pure hydrochloride salt (130 mg, 86%) as a colourless gum. δ_H_ (400 MHz, DMSO-*d_6_*): 6.61 (dd, *J* = 7.6, 2.3 Hz, 1H, H-4), 6.76 (t, *J* = 2.2 Hz, 1H, H-2), 6.92 (dd, *J* = 7.8, 1.7 Hz, 1H, H-6), 7.16 (d, *J* = 9.1 Hz, 2H, H-8 and H-8′ or H-9 and H-9′), 7.30–7.34 (m, 2H, H-5 and H-16), 7.37 (d, *J* = 9.0 Hz, 2H, H-8 and H-8′ or H-9 and H-9′), 7.40 (d, *J* = 2.7 Hz, 1H, H-12), 7.50 (d, *J* = 8.8 Hz, 1H, H-15), 8.61 (bs, 4H, NH), 8.88 (bs, NH). δ_C_ (100 MHz, DMSO-*d_6_*): 107.8 (CH Ar, C-2), 111.2 (CH Ar, C-4), 113.1 (CH Ar, C-6), 114.7 (q, *J* = 5.5 Hz, CH Ar, C-12), 119.4 (qC, C-14), 120.4 (CH Ar, C-5 or C-16), 120.5 (2 CH Ar, C-8 and C-8′ or C-9 and C-9′), 122.0 (d, *J* = 227.5 Hz, qCF_3_), 123.1 (2 CH Ar, C-8 and C-8′ or C-9 and C-9′), 127.2 (d, *J* = 30.7 Hz, qC, C-13), 130.9 (CH Ar, C-5 or C-16), 132.7 (CH Ar, C-15), 142.8 (qC), 143.5 (qC), 145.2 (qC), 155.2 (qC), 157.6 (qC), 161.1 (qC). δ_F_ (376 MHz, CD_3_OD):—61.59 (s). ν_max_(ATR)/cm^−1^: 3285 (NH), 2924, 2854, 1693, 1655, 1593 (C=N), 1481 (C-N), 1400, 1258, 1231, 1195, 1175 (C-O), 1128 (CF_3_), 1111 (C-Cl), 1027, 824, 681, 665. HRMS (*m/z* ESI^+^): found: 422.0883 (M^+^ + H. C_20_H_16_N_3_O_2_ClF_3_ requires: 422.0883). HPLC: 96.2% (*t*R: 32.2 min).

#### 4.2.20. 4’-Sulfonamide-3-[4-chloro-3 trifluoromethylphenylamino]diphenylether (75)

Compound **40** (100 mg, 0.26 mmol, 1 eq.), sulfamoyl chloride (30 mg, 0.26 mmol, 1 eq.) and NEt_3_ (0.05 mL, 0.29 mmol, 1.1 eq.) were dissolved in CH_2_Cl_2_ (2 mL) and stirred at overnight at room temperature. The mixture was then washed with water and the organic layer extracted with EtOAc, washed with brine, dried over MgSO_4_, concentrated under vacuum and purified by silica gel chromatography (hexanes:EtOAc) to get **75** as a light brown solid (95 mg, 80%). Mp: 124–126 °C. δ_H_ (400 MHz, CD_3_OD): 6.54 (dd, *J* = 8.2, 2.3 Hz, 1H, H-4), 6.69 (t, *J* = 2.2 Hz, 1H, H-2), 6.84 (dd, *J* = 8.1, 2.1 Hz, 1H, H-6), 6.99 (d, *J* = 9.0 Hz, 2H, H-8 and H-8′), 7.21–7.26 (m, 4H, H-9 and H-9′, H-16, H-5), 7.34–7.38 (m, 2H, H-12, H-15). δ_C_ (100 MHz, CD_3_OD): 109.1 (CH Ar, C-2), 112.2 (CH Ar, C-4), 113.8 (CH Ar, C-6), 116.1 (q, *J* = 5.5 Hz, C-12, CH Ar), 121.1 (2 CH Ar, C-8 and C-8′), 121.2 (CH Ar, C-16), 121.9 (qC, C-14), 123.2 (2 CH Ar, C-9 and C-9′), 124.4 (d, *J* = 272.5 Hz, qCF_3_), 129.7 (d, *J* = 30.9 Hz, qC, C-13), 131.5 (CH Ar, C-5), 133.3 (CH Ar, C-15), 136.1 (qC), 144.6 (qC), 145.1 (qC), 154.4 (qC), 160.5 (qC). δ_F_ (376 MHz, CD_3_OD):—64.19 (s). ν_max_ (ATR)/cm^−1^: 3404 (NH), 3279 (NH), 1596 (S=O), 1489 (S=O), 1143 (C-O), 1153 (CF_3_), 1125 (C-N), 830 (C-Cl), 821. HRMS (*m/z* ESI^−^): found: 456.0397 (M^−^—H. C_19_H_14_N_3_O_3_SClF_3_ requires: 456.0397). HPLC: 99.3% (*t*R: 35.4 min).

### 4.3. Biochemistry

#### 4.3.1. Cell Viability Studies (alamarBlue)

Cells were counted and seeded in 96-well plates at a density of 2 × 10^5^ cells/mL for HL-60, 2.5 × 10^4^ cells/mL for MCF-7, MCF10A and HeLa, 1 × 10^5^ cells/mL for HCT116 and HKH-2, all of them in their respective media. The 96-well plates were then treated with a 1:100 dilution of stock concentrations of drugs or EtOH (1% *v/v*)/DMSO (0.1% *v/v*) as vehicle control in triplicate. Three blank wells containing 200 µL RPMI with no cells were also set-up as blanks. After a 72 h incubation, 20 µL of alamarBlue was added to each well. The plates were incubated in darkness at 37 °C for 4–5 h using a Molecular Devices microplate reader, the fluorescence (F) was then read at an excitation wavelength of 544 nm and an emission wavelength of 590 nm. Cell viability was then determined by subtracting the mean blank fluorescence (Fb) from the treated sample fluorescence (Fs) and expressing this as a percentage of the fluorescence of the blanked vehicle control (Fc). This is demonstrated in the equation below. The results were then plotted as a nonlinear regression, sigmoidal dose-response curves on Prism, from which the IC_50_ value for each drug was determined.

#### 4.3.2. Flow Cytometry

Apoptosis was analysed using annexin V fluorescein isothiocyanate (FITC) and propidium iodide (PI). HL-60 cells were seeded at a density of 2 × 10^5^ cells/mL in 12 well plates. Cells were then treated with either vehicle (0.5% ethanol), **2** (5 µM), **3** (5 µM), or **4** (4 µM) for 48 h. Following treatment, HL60 cells were collected and washed with annexin V binding buffer (5 mM HEPES, 70 mM NaCl, 1.25 mM CaCl2 pH 7.4) and stained with annexin V-FITC (iQ Corporation, Groningen, The Netherlands) for 20 min. Following washing with annexin V binding buffer, cells were resuspended in PI (0.5 µg/mL) in binding buffer and analysed on BD FACS Canto II flow cytometer (BD Sciences) using FloJo software (Ashland, OR, USA).

#### 4.3.3. Western Blotting

HL60s were seeded in T25 flasks at 50 × 10^4^ cells/mL and cells were treated with either vehicle [0.5% EtOH (*v/v*)], **1**–**4**, **9** or **52** (5 µM), as well as **1** (10 µM as reported [9]). After 16 h, cells were collected and washed with PBS. Cell pellets were re-suspended in cold cell lysis buffer (radio-immunoprecipitation assay buffer) supplemented with 1% phosphatase inhibitor cocktail 2 and 3 (Sigma) and 10% protease inhibitor (Roche). Cells were lysed for 30 min on ice. Protein concentration was then determined by BCA assay. Lysates were boiled with Laemmli sample buffer [Tris-HCL 50 mM (pH 6.7), glycerol 10% (*w/v*), sodium dodecyl sulphate 2% (*w/v*), bromophenol blue 0.02% (*w/v*)] containing DTT 50 µM for 10 min at 90 °C. Moreover, 20 µg of lysates were resolved by sodium dodecyl sulphate polyacrylamide gel electrophoresis (SDS-PAGE) and transferred onto PVDF transfer membrane (EMD Millipore). Membranes were blocked with 5% non-fat milk and probed with primary antibodies for ERK and phospho-ERK (Cell Signalling). Anti-GAPDH was used as a loading control (Calbiochem).

## 5. Conclusions

Taking all of these results into account, the following SARs were drawn in terms of HL-60 cytotoxicity; for example, for a good cytotoxicity bulky substituents (4-Br/Cl and 3-CF_3_) in the phenyl ring of the hydrophobic moiety are needed; replacement of the di-substituted guanidinium as in compound **1** by a shorter -NH- link is also beneficial; a mono-substituted guanidinium group at the position 4′ of the phenyloxyaryl core gives good cytotoxic activity; additionally, substituting one of the phenyl rings by a 2-pyridinyl to facilitate IMHB seems also to increase the cytotoxic activity in the 3,4′-bis-guanidinium series. On the negative side, in the bis-guanidinium diphenyl ether series, when only one substituent is kept in the phenyl ring of the hydrophobic moiety (4-Br or 4-F as in **8** or **5**) or both are very small (3,4-diF as in **6**), cytotoxicity decreases or is completely abolished. This is not the case either in the amino-guanidinium diphenyl ether or in the bis-guanidinium phenyloxypyridine series where mono-substituted or di-fluoro phenyl rings in the hydrophobic moiety still exhibit good HL-60 cytotoxicity (i.e., compounds **28**–**30** or **49** and **51**).

Future work will be required to investigate the molecular target(s) of our guanidinium derivatives, but nonetheless, while compound **2**, **9,** and **52** seem to be improved derivatives of previous *lead* molecule **1**, compounds **3** and **4** can be considered excellent *hit* molecules in the search for new anticancer therapies.

## Figures and Tables

**Table 1:** Effect on the viability of HL-60 cells (IC_50_, μM) of compounds **1**–**11**, **28**–**30**, **36**, **49**–**52**, **70**, **75** and sorafenib with alamarBlue^®^ assays.

Compounds.	HL-60 IC_50_ ± SEM	Compounds.	HL-60 IC_50_ ± SEM	Compounds.	HL-60 IC_50_ ± SEM
**Sorafenib**	2.53 ± 0.68	**8**	15.26 ±1.98	**49**	11.61 ±0.27
**1** ^7^	9.72 ± 0.9	**9**	2.07 ± 0.33	**50**	8.33 ± 0.32
**2**	2.36 ± 0.14	**10**	8.85 ± 0.55	**51**	9.37 ± 0.54
**3**	3.08 ± 0.15	**11**	10.99 ±0.64	**52**	1.53 ± 0.23
**4**	3.48 ± 0.28	**28**	7.50 ± 0.05	**70**	4.22 ± 0.04
**5**	>100	**29**	4.64 ± 0.83	**75**	9.14 ± 0.69
**6**	>100	**30**	3.23 ± 0.36		
**7**	8.63 ± 0.51	**36**	4.07 ± 0.10		

(a) Cells were seeded at a density of 2 × 10^5^ cells/mL (HL-60) in a 96-well plate and treated with different concentrations of the compounds dissolved in EtOH or DMSO (1% *v/v* and 0.1% *v/v*, respectively). Sorafenib was used as a reference and tested in the same manner. Once treated, cells were incubated for 72 h at 37 °C after which they were treated with alamarBlue^®^ and left in darkness in an incubator for 5 h. The resulting fluorescence (λ_excitation_ = 544 nm, λ_emission_ = 590 nm) was read using a plate reader from which percentage viability was calculated. IC_50_ values were calculated using Prism GraphPad Prism software from at least three independent experiments performed in triplicate. Highlighted in grey are those IC_50_ values better than or similar to the control used, Sorafenib.

**Table 2:** Effect in viability of MCF-7, HeLa, HCT116, and HKH-2 cancer cells (IC_50_, μM) of compounds **1**–**4**, **70** and sorafenib on the alamarBlue^®^ assays.

Compds.	MCF-7 IC_50_ ± SEM	HeLa IC_50_ ± SEM	HCT116 IC_50_ ± SEM	HKH-2 IC_50_ ± SEM
**Sorafenib**	3.07 ± 0.10	4.59 ± 0.45	6.79 ± 0.18	4.43 ± 0.39
**1**	9.30 ± 1.87	9.48 ± 0.16	9.96 ± 0.60	19.18 ± 0.84
**2**	4.91 ± 1.04	4.87 ± 0.27	7.29 ± 0.53	8.09 ± 0.78
**3**	2.02 ± 0.27	4.33 ± 0.54	15.88 ± 2.73	10.34 ± 0.74
**4**	3.73 ± 0.35	1.33 ± 0.08	4.59 ± 0.40	2.88 ± 0.15
**70**	7.32 ± 0.35			

(a) Cells were seeded at a density of 25 × 10^3^ cells/mL (MCF-7 and HeLa) or 1 × 10^5^ cells/mL (HCT-116 and HKH-2) in a 96-well plate and treated with different concentrations of the compounds dissolved in EtOH or DMSO (1% *v/v* and 0.1% *v/v*, respectively). Sorafenib was used as a reference and tested in the same manner. Once treated, cells were incubated for 72 h at 37 °C after which they were treated with alamarBlue^®^ and left in darkness in an incubator for 5 h. The resulting fluorescence (λ_excitation_ = 544 nm, λ_emission_ = 590 nm) was read using a plate reader from which percentage viability was calculated. IC_50_ values were calculated using Prism GraphPad Prism software from at least three independent experiments performed in triplicate. Highlighted in grey are those IC_50_ values better than or similar to the control used, Sorafenib, in each particular cell line.

## Supplementary Materials

The following are available online at https://www.mdpi.com/1424-8247/13/12/485/s1, synthetic details (general information, general procedures, characterisation of intermediates); computational details (Figure S1, Figure S2, Figure S3, Figure S4 and Figure S5); theoretical physicochemical and pharmacokinetic parameters (Table S1, Table S2, and Table S3, Figure S6); biochemical protocols (Figure S7, Figure S8, Figure S9, Figure S10, Figure S11, Figure S12, Figure S13); NMR spectra of final salts; and HPLC chromatograms of final salts.
