# Supplementary material for: Exploring the Anti-Cancer Mechanism of Novel 3,4′-Substituted Diaryl Guanidinium Derivatives
Source: Pharmaceuticals (Basel). 2020 Dec 21;13(12):485. doi: 10.3390/ph13120485 (PMC7767381; doi:10.3390/ph13120485)
Supplement: Supplementary file 1 [file pharmaceuticals-13-00485-s001.pdf]

Supporting Information

Design, Synthesis and Anti-Cancer Activity of a Novel Series of Diaromatic Guanidinium Derivatives

Viola Previtali,[a] Helene B. Mihigo,[a] Rebecca Amet,[b] Anthony M. McElligott,[c] Daniela Zisterer,[b] Isabel Rozas[a,]*

[a]*School of Chemistry, TBSI, TCD, 152-160 Pearse Street, Dublin 2, Ireland*
[b]*School of Biochemistry and Immunology, TBSI, TCD, 152-160 Pearse Street, Dublin 2, Ireland*
[c]*Trinity Translational Medicine Institute, Trinity College and St James's Hospital, Dublin 8, Ireland*

Table of contents

1. Synthetic details

    1.1. General information

    1.2. General procedures

    1.3. Characterisation of intermediates

2. Computational details

3. Theoretical physicochemical and pharmacokinetic parameters

4. Biochemical protocols

5. NMR Spectra of Final Salts

6. HPLC Chromatograms of Final Salts

7. References

1. Synthetic details

1.1. General Information

Reagents were purchased at the highest commercial quality and used without further purification, unless otherwise stated. Dry solvents were obtained from PureSolvTM solvent purification system (Innovative Technology, Inc.) or otherwise purchased from Sigma-Aldrich. Solvents for synthesis purposes were used at GPR grade. Yields refer to chromatographically and spectroscopically (^{1}H NMR) homogeneous materials, unless otherwise stated. Reactions were monitored by thin layer chromatography (TLC) using Merck Kieselgel 60 F254 silica gel or aluminium oxide plates. Visualisation was achieved by UV light (254 nm) and basic aqueous potassium permanganate ($KMnO_4$) or ninhydrin and heat as developing agents. Concentration of organic solvents was performed on a rotary evaporator under reduced pressure followed by further evacuation using a two-stage mechanical pump.

Deuterated solvents for NMR use were purchased from Apollo and Euriso-top. Chromatographic columns were run using silica gel 60 (230–400 mesh ASTM). NMR spectra were recorded on Bruker DPX-600 and Bruker DPX-400 instruments operating at 400.13 MHz and 600.1 MHz for ^{1}H NMR and at 100.6 MHz and 150.9 MHz for ^{13}C NMR and they were calibrated using residual undeuterated solvent as an internal reference ($CHCl_3$ at δ 7.26 and 77.16 ppm for ^{1}H NMR and ^{13}C NMR, respectively; CH_3OH at δ 3.31 and 49.00 ppm for ^{1}H NMR and ^{13}C NMR, respectively; DMSO at δ 2.50 and 39.52 ppm for ^{1}H NMR and ^{13}C NMR, respectively). NMR data were processed using MestreNova software. The following abbreviations (or combinations thereof) were used in NMR characterisation: s = singlet, d = doublet, t = triplet, q = quartet, p = pentaplet, m = multiplet, bs = broad singlet, H = proton, CH Ar, aromatic carbon, qC = quaternary carbon. Compounds are assigned arbitrary numbers for assignment of the NMR spectrum and these numbers do not correspond to the naming of the compound. High-resolution mass spectra (HRMS) were measured on a Micromass LCT electrospray TOF instrument with a WATERS 2690 autosampler and HPLC grade methanol as carrier solvent. Melting points were determined using a Stuart Scientific Melting Point SMP1 apparatus and are uncorrected. Infrared spectra were recorded on a Perkin Elmer Spectrum One FT-IR Spectrometer equipped with a Universal ATR sampling accessory. HPLC purity analysis was carried out using a Varian ProStar system equipped with a Varian Prostar 335 diode array detector and a manual injector (20 µL). For purity assessment, UV detection was

performed at 254 nm and peak purity was confirmed using a purity channel. The stationary phase consisted of an ACE 5 C18-AR column (150 × 4.6 mm), and the mobile phase used the following gradient system, eluting at 1 mL/min: aqueous formate buffer (30 mM, pH 3.0) for 10 min, linear ramp to 85% methanol buffered with the same system over 25 min, hold at 85% buffered methanol for 10 min. Minimum requirement for purity was set at 95.0%.

1.2. General procedures

Method A: General procedure for the deprotection of *N*-Boc protected diaryl guanidines using hydrochloric acid.[1]

To a solution of the corresponding Boc protected diaryl guanidine (1 eq.) in 1,4-dioxane, 4 M HCl in 1,4-dioxane (6 eq. per Boc group) was added to reach a guanidine final concentration of 0.2 M. The mixture was stirred at 55 °C until the reaction was complete (typically 6-8 h, adjudged by TLC). At the reaction endpoint, solvent and HCl were evaporated under vacuum and the crude salt was dissolved in the minimum volume of $CHCl_3$. It was then purified by flash chromatography (silica gel, $CHCl_3$:MeOH). The purified fractions were evaporated until dry to afford the pure hydrochloride salt.

Method B: General procedure for the synthesis of *N*-aryl-*N′*-Boc-protected thiourea derivatives.[1]

To a solution of *N*,*N′*-bis-(*tert*-butoxycarbonyl)thiourea (1 eq.) in dry tetrahydrofuran (0.12 M) under argon at 0 °C, sodium hydride as a 60% suspension in mineral oil (1.5 eq.) were added. The reaction mixture was stirred at the same temperature for 1 h and 30 min, then trifluoroacetic anhydride (1.1 eq.) was added and the stirring continued for an additional 30 min. The corresponding aniline (1.15 eq.) was added and the reaction was stirred at 0 °C for 5 h. The reaction was cooled again to 0 °C and dropwise H_2O was added to quench the reaction, followed by extraction with EtOAc. The combined organic phases were washed with 80% brine and dried using anhydrous $MgSO_4$, followed by removal of solvents under vacuum. The compounds were purified by silica gel chromatography and/or recrystallization (as specified for each compound) to afford the desired product.

Method C: General procedure for the synthesis of asymmetric Boc-protected 3,4'-bis-guanidine derivatives.[1]

Mercury(II) chloride ($HgCl_2$) (1.2 eq.) was added to a solution of the corresponding mono Boc-protected guanidine (1 eq.), the corresponding thiourea (1.2 eq.) and triethylamine (3.1 eq.) in CH_2Cl_2 (0.19 M). The resulting mixture was stirred at 0 °C for 1 h and then at room temperature for 6-8 h or overnight depending (as specified for each compound). The crude was then diluted with EtOAc and filtered through a pad of Celite® in order to remove the mercury sulfide precipitate formed and the filter cake was rinsed with EtOAc. The organic phase was extracted with water, washed with brine, dried over anhydrous $MgSO_4$, and concentrated under vacuum to give a crude residue that was purified by flash chromatography on silica gel (eluting with a gradient of hexane:EtOAc).

Method D: General procedure for *N*-Arylation.[2]

An oven-dried round bottom flask was charged with a magnetic stir bar, $Pd_2(dba)_3$ (3 mol%), organophosphorus ligand (3-5 mol%), aniline derivative (1 eq.), base (1.4 eq.) and bromine source (1 eq.). The flask was evacuated and refilled with argon (three times). In case of liquid bromine source, it was added by syringe under a counterflow of argon, followed by addition of dry toluene (2 mL/mmol). The mixture was placed in a preheated oil bath at 90 °C and stirred for 24 h. The reaction mixture was then cooled to room temperature, diluted with EtOAc, filtered through a pad of Celite®, and washed with water. The organic layer was washed with brine, dried over $MgSO_4$, concentrated under vacuum and purified by silica gel chromatography (hexanes:EtOAc) to obtain the desired product.

Method E: General procedure for nitro-reduction using Palladium on carbon (Pd/C).

To a solution of appropriate nitro derivative (1 eq.) in specified solvent, was added 10% Pd/C mixture (10 mol%), and the reaction mixture was stirred at room temperature under hydrogen atmosphere (1.0 atm) for 24 h. The reaction mixture was filtered off, and the filtrate was concentrated in vacuo. The residue was purified by silica gel chromatography

(hexanes:EtOAc), and the desired fractions were concentrated under reduced pressure to obtain the desired product.

Method F: General procedure for nitro-reduction using $SnCl_2·2H_2O$.[3]

A mixture of appropriate nitro derivative (1 eq.) dissolved in absolute ethanol and $SnCl_2·2H_2O$ (6 eq.) was stirred at 70 °C until the reaction was complete (typically 3 h, adjudged by TLC as specified for each compound). The solution was allowed to cool down and then poured into ice. The pH was made slightly basic (pH 7-8) by addition of 5% aqueous $NaHCO_3$. The solution was washed with water, the organic layer extracted with EtOAc, washed with brine, dried over $MgSO_4$, concentrated under vacuum and purified by silica gel chromatography (hexanes:EtOAc) to obtain the desired product.

Method G: General procedure for the synthesis of 1-aryl-2,3-di(*tert*-butoxycarbonyl)guanidine derivatives.[4]

Mercury(II) chloride ($HgCl_2$) (1.04 eq.) was added to a solution of the corresponding aromatic amine (1 eq.), *N,N'*-bis-(*tert*-butoxycarbonyl)-*S*-methylisothiourea (1.04 eq.) and triethylamine (NEt_3) (3.5 eq.) in CH_2Cl_2 (0.19 M). The resulting mixture was stirred at 0 °C for 1 h and then 24 h or 48 h (as specified for each compound) at room temperature (reaction progress adjudged by TLC). The crude was then diluted with with EtOAc and filtered through a pad of Celite® in order to remove the mercury sulfide precipitate formed. The filter cake was rinsed with EtOAc. The organic phase was extracted with water, washed with brine, dried over anhydrous $MgSO_4$, and concentrated under vacuum to give a residue that was purified by flash chromatography on silica gel as specified.

Method H: General procedure for the synthesis of arylamido derivatives.[5]

The appropriate benzoic acid derivative (1.2 eq.) was placed under nitrogen atmosphere and dissolved in a 0.3 M solution of dry dichloromethane (CH_2Cl_2). Oxalyl chloride (2 eq.) was added, followed by dimethylformamide (DMF) (3 drops approx.). The solution was stirred overnight. Any remaining oxalyl chloride and DMF was removed under vacuum to obtain a

yellow residue that was dissolved in CH_2Cl_2 (0.6 M) and cooled to 0 ℃. Corresponding aniline (1 eq.) and triethylamine (NEt_3) (2.4 eq.) was also dissolved in CH_2Cl_2 and added dropwise to the cooled solution. After 12 h the solution was warmed to room temperature. The organic layer was extracted using water, washed with brine, dried over $MgSO_4$ and concentrated under vacuum to give a residue that was purified by silica gel chromatography (eluting with a gradient of hexane:EtOAc) to yield final compound.

1.3. Details of precursors

1.3.1. Functionalised Thioureas

N-(*tert*-Butoxycarbonyl)-*N'*-(4-fluorobenzene)thiourea (12)

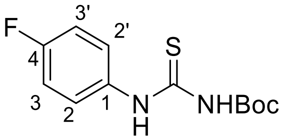

Following Method B, to a solution of *N,N'*-bis-(*tert*-butoxycarbonyl)thiourea (422 mg, 1.53 mmol) in dry THF (12 mL) under argon at 0 °C, NaH as a 60% suspension in mineral oil (91 mg, 2.30 mmol) was added. The reaction mixture was stirred at the same temperature for 1.5 h, then 1.1 eq. of trifluoroacetic anhydride (0.20 mL, 1.68 mmol) were added, and the stirring continue for an additional 30 min. 1.15 eq. of 4-fluoroaniline (196 mg, 1.76 mmol) were then added and the reaction was stirred at 0 °C for 5 h. Usual work-up followed by silica gel chromatography (hexane:Et_2O) afforded the desired product as a white solid (173 mg, 42%). **Mp**: 132-134 °C. $\boldsymbol{\delta_H}$ (400 MHz, $CDCl_3$): 1.53 (s, 9H, $(CH_3)_3$), 7.04 – 7.12 (m, 2H, H-2 and H-2'), 7.54 – 7.61 (m, 2H, H-3 and H-3'), 7.97 (bs, 1H, NH), 11.42 (s, 1H, NH). $\boldsymbol{\delta_C}$ (100 MHz, $CDCl_3$): 28.0 ($(CH_3)_3$), 84.5 (qC, $\underline{C}(CH_3)_3$), 115.7 (d, *J* = 22.9 Hz, 2 CH Ar, C-3 and C-3'), 126.5 (d, *J* = 8.2 Hz, 2 CH Ar, C-2 and C-2'), 133.7 (d, *J* = 2.9 Hz, qC, C-1), 151.9 (qC), 160.9 (d, *J* = 246.7 Hz, qC, C-4), 178.9 (qC). **HRMS** (m/z ESI^-): Found: 269.0756 (M^- - H), $C_{12}H_{14}FN_2O_2S$ Requires: 269.0766. $\boldsymbol{\nu_{max}}$ (ATR)/cm^{-1}: 3168 (NH), 2983, 2931, 1715 (C=O), 1525-1507 (C-F), 1249, 1146 (C=S), 838, 727.

N-(*tert*-Butoxycarbonyl)-*N'*-(3,4-difluoromethylphenyl)thiourea (13)

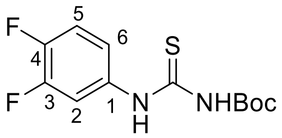

Following Method B, to a solution of *N,N'*-bis-(*tert*-butoxycarbonyl)thiourea (1000 mg, 3.6 mmol) in dry THF (27 mL) under argon at 0 °C, NaH as a 60% suspension in mineral oil (216 mg, 5.4 mmol) was added. The reaction mixture was stirred at the same temperature for 1.5 h, then 1.1 eq. of trifluoroacetic anhydride (0.55 mL, 3.96 mmol) were added, and the stirring continue for an additional 30 min. 1.15 eq. of 3,4-difluoroaniline (0.41 mg, 4.14 mmol) were then added and the reaction was stirred at 0 °C for 5 h. Usual work-up followed by silica gel chromatography (hexane:Et_2O) afforded the desired product as a white solid (592 mg, 57%). **Mp**: 116-118 °C. **δ_H** (400 MHz, $CDCl_3$): 1.53 (s, 9H, (CH3)3), 7.16 (dd, 1H, *J* = 18.1, 9.0 Hz, H-2), 7.25 - 7.27 (m, 1H, H-6), 7.71 - 7.76 (m, 1H, H-5), 7.96 (s, 1H, NH), 11.52 (s, 1H, NH). **δ_C** (150 MHz, $CDCl_3$): 28.0 (($(CH_3)_3$)), 84.7 (qC, C($(CH_3)_3$)), 114.0 (d, *J* = 20.8 Hz, CH Ar, C-5), 117.1 (d, *J* = 18.3 Hz, CH Ar, C-2), 120.3 (dd, *J* = 6.1, 3.6 Hz, CH Ar, C-6), 134.1 (dd, *J* = 8.3, 3.4 Hz, qC, C-1), 148.5 (d, *J* = 248.6, 13.0 Hz, qC, C-3 or C-4), 149.82 (d, *J* = 248.6 , 13.4 Hz , qC, C-3 or C-4) 151.9 (qC), 178.6 (qC). **HRMS** (m/z ESI^-): Found: 287.0661 (M^-- H), $C_{12}H_{13}F_2N_2O_2S$ Requires: 287.0671. **ν_{max}** (ATR)/cm^{-1}: 3183 (NH), 3081, 2978, 1713 (C=O), 1514 (C-F), 1251, 1112 (C=S), 1139, 723.

***N*-(*tert*-Butoxycarbonyl)-*N'*-(4-iodo-2-fluoromethylphenyl)thiourea (14)**

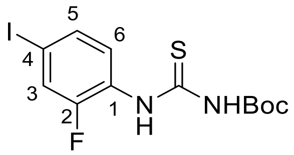

Following Method B, to a solution of *N,N'*-bis-(*tert*-butoxycarbonyl)thiourea (1350 mg, 4.88 mmol) in dry THF (40 mL) under argon at 0 °C, NaH as a 60% suspension in mineral oil (293 mg, 7.32 mmol) was added. The reaction mixture was stirred at the same temperature for 1.5 h, then 1.1 eq. of trifluoroacetic anhydride (0.75 mL, 5.37 mmol) were added, and the stirring continue for an additional 30 min. 1.15 eq. of 2-fluoro-4-iodoaniline (1531 mg, 5.61 mmol) were then added and the reaction was stirred at 0 °C for 5 h. Usual work-up followed by silica gel chromatography (hexane:Et_2O) and recrystallization from boiling CH_2Cl_2, afforded the desired product as a white solid (1109 mg, 57%). **Mp**: 156-158 °C. **δ_H** (400 MHz, $CDCl_3$): 1.54 (s, 9H, $(CH_3)_3$), 7.49 – 7.51 (m, 2H, H-3 and H-6), 8.02 (bs, 1H, NH), 8.17 – 8.21 (m, 1H, H-5),

11.63 (bs, 1H, NH). **δ_C** (150 MHz, $CDCl_3$): 28.2 ($(CH_3)_3$), 84.9 (qC, $\underline{C}(CH_3)$), 89.6(d, *J* = 7.5 Hz, qC, C-4), 124.9 (d, *J* = 21.8 Hz, CH Ar, C-3), 126.3 (d, *J* = 10.5 Hz, qC, C-1), 126.7 (CH Ar, C-5), 133.4 (d, *J* = 3.8 Hz, CH Ar, C-6), 151.9 (qC), 154.6 (d, *J* = 253.5 Hz, qC, C-2), 178.4 (qC). **HRMS** (m/z ESI$^-$): Found: 394.9703 (M^- - H), $C_{12}H_{13}FIN_2O_2S$ Requires: 394.9732. **ν_{max}** (ATR)/cm^{-1}: 3339 (NH), 2975, 1719 (C=O), 1587 (C-F), 1505, 1481, 1342, 1243, 1206, 1133 (C=S), 949, 853, 728 (C-I).

***N*-(*tert*-Butoxycarbonyl)-*N'*-(4-bromophenyl)thiourea (15)**

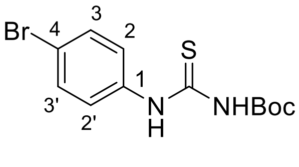

Following Method B, to a solution of *N,N'*-bis-(*tert*-butoxycarbonyl)thiourea (2000 mg, 7.24 mmol) in dry THF (59 mL) under argon at 0 °C, NaH as a 60% suspension in mineral oil (434 mg, 10.86 mmol) was added. The reaction mixture was stirred at the same temperature for 1.5 h, then 1.1 eq. of trifluoroacetic anhydride (1.11 mL, 7.96 mmol) were added, and the stirring continue for an additional 30 min. 1.15 eq. of 4-bromoaniline(1432 mg, 8.33 mmol) were then added and the reaction was stirred at 0 °C for 5 h. Usual work-up followed by silica gel chromatography (hexane:Et_2O) and recrystallization from boiling CH_2Cl_2, afforded the desired product as a white solid (1031 mg, 43%). **Mp**: 150 - 153 °C. **δ_H** (400 MHz, $CDCl_3$): 1.53 (s, 9H, CH_3), 7.50 (d, *J* = 8.8 Hz, 2H, H-2 and H-2'), 7.56 (d, *J* = 8.8 Hz, 2H, H-3 and H-3'), 7.96 (s, 1H, NH), 11.51 (s, 1H, NH). **δ_C** (150 MHz, $CDCl_3$): 28.2 ($(CH_3)_3$), 84.7 (qC, $\underline{C}(CH_3)$), 119.9 (qC, C-4), 125.9 (2 CH Ar, C-2 and C-2'), 132.0 (2 CH Ar, C-3 and C-3'), 136.9 (qC, C-1), 152.0 (q), 178.4 (qC). **HRMS** (m/z ESI$^-$): Found: 328.9967 (M^- - H), $C_{12}H_{14}BrN_2O_2S$ Requires: 328.9965. **ν_{max}** (ATR)/cm^{-1}: 3415 (NH), 3200, 2982, 1715 (C=O), 1524, 1487, 1137 (C=S), 905 (C-Br), 726.

***N*-(*tert*-Butoxycarbonyl)-*N'*-(4-bromo-3-trifluoromethylphenyl)thiourea (16)**

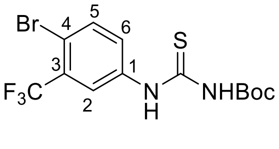

Following Method B, to a solution of *N,N'*-bis-(*tert*-butoxycarbonyl)thiourea (925 mg, 3.38 mmol) in dry THF (27 mL) under argon at 0 °C, NaH as a 60% suspension in mineral oil (202 mg, 5.07 mmol) was added. The reaction mixture was stirred at the same temperature for 1.5 h, then 1.1 eq. of trifluoroacetic anhydride (0.52 mL, 3.72 mmol) were added, and the stirring

continue for an additional 30 min. 1.15 eq. of 4-bromo-3-(trifluoromethyl)aniline(933 mg, 3.89 mmol) were then added and the reaction was stirred at 0 °C for 5 h. Usual work-up followed by silica gel chromatography (hexane:Et_2O) and recrystallization from boiling CH_2Cl_2, to afford the desired product as a white solid (500 mg, 37%). **Mp**: 132 - 134 °C. **δ_H** (600 MHz, $CDCl_3$): 1.54 (s, 9H, CH_3), 7.71 (d, 1H, *J* = 8.6 Hz, H-5), 7.84 (dd, 1H, *J* = 8.6, 1.8 Hz, H-6), 7.98 – 8.02 (m, 2H, NH and H-2), 11.68 (bs, 1H, NH). **δ_C** (150 MHz, $CDCl_3$): 28.2 ($(CH_3)_3$), 85.1 (qC, $\underline{C}(CH_3)$), 117.0 (qC, C-4), 123.5 (q, *J* = 11.1 Hz, CH Ar, C-2), 124.5 (d, *J* = 273.6 Hz, qCF_3), 128.3 (CH Ar, C-6), 130.8 (d, *J* = 31.9 Hz, qC, C-3), 135.4 (CH Ar, C-5), 137.3 (qC), 152.1 (qC), 178.6 (qC). **HRMS** (m/z ESI^-): Found: 396.9832(M^- - H), $C_{13}H_{13}N_2O_2BrF_3S$ Requires: 396.9839. **ν_{max}** (ATR)/cm^{-1}: 3159 (NH), 1706 (C=O), 1592, 1533, 1474, 1324, 1237 (C=S), 1128 (C-F), 1016 (C-Br), 818, 688.

***N*-(*tert*-Butoxycarbonyl)-*N'*-(4-chloro-3-trifluoromethylphenyl)thiourea (17)[1]**

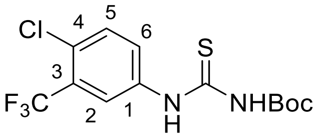

Following Method B, to a solution of *N,N'*-bis-(*tert*-butoxycarbonyl)thiourea (2000 mg, 7.25 mmol) in dry THF (58 mL) under argon at 0 °C, NaH as a 60% suspension in mineral oil (435 mg, 10.9 mmol) was added. The reaction mixture was stirred at the same temperature for 1.5 h, then 1.1 eq. of trifluoroacetic anhydride (1.11 mL, 8.0 mmol), and the stirring continue for an additional 30 min. Then, 1.15 eq. of 4-chloro-3-(trifluoromethyl)aniline (1.63 g, 8.34 mmol) were added and the reaction was stirred at 0 °C for 5 h. Usual work-up followed by silica gel chromatography (eluting with a gradient of hexane:EtOAc) and recrystallization from boiling hexane, to afford the desired product as a white solid (1414 mg, 55%). **Mp**: 138 – 140 ºC. (lit. 140-142 ºC).[1] **δ_H** (400 MHz, $CDCl_3$): 1.54 (s, 9H, CH_3), 7.51 (d, 1H, *J* = 8.7 Hz, H-5), 7.91 (d, 1H, *J* = 8.6, 2.3 Hz, H-6), 7.96 – 8.00 (m, 2H, NH, H-2), 11.67 (bs, 1H, NH).

1.3.2. Nitrophenyl derivatives

3-(4-nitrophenoxy)aniline (31)[6]

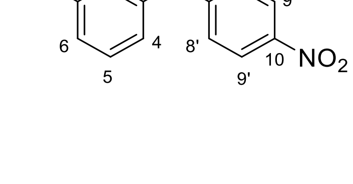

1-Fluoro-4-nitrobenzene (100 mg, 0.71 mmol, 1 eq.), 3-aminophenol (117 mg, 1.07 mmol, 1.5 eq.) and K_2CO_3 (1148 mg, 1.07 mmol, 1.5 eq.) were dissolved in DMF (1.5 mL) and stirred at 80 ˚C for 12 h. The mixture was cooled to room temperature, washed with water and the organic layer extracted with EtOAc, washed with brine, dried over $MgSO_4$, concentrated under vacuum and purified by flash chromatography (silica gel, hexanes:EtOAc) to get **31** as a yellow solid (98 mg, 60%). **Mp**: 76-79 °C (lit. 79 °C).[6] **δ_H** (400 MHz, $CDCl_3$): 3.79 (bs, 2H, NH_2), 6.39 (app. t, *J* = 2.2 Hz, 1H, H-2), 6.45 (dd, *J* = 8.0, 2.2 Hz, 1H, H-4 or H-6), 6.55 (dd, *J* = 8.1, 2.2 Hz, 1H, H-4 or H-6), 7.02 (d, *J* = 9.2, 2H, H-8 and H-8'), 7.18 (t, *J* = 8.0 Hz, 1H, H-5), 8.19 (d, *J* = 9.2, 1H, H-9 and H-9').

4-chloro-N-(3-(4-nitrophenoxy)phenyl)-3-(trifluoromethyl)aniline (32)

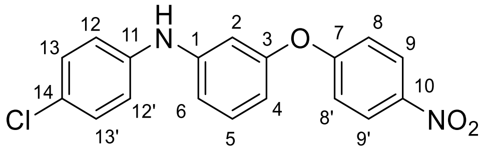

Following Method D, $Pd_2(dba)_3$ (3 mol%, 55 mg), BINAP (3 mol%, 37 mg), compound **31** (460 mg, 2 mmol), NaO*t*Bu (269 mg, 2.8 mmol) and remaining 1-bromo-4-chlorobenzene (383 mg, 2 mmol) were mixed, followed by syringe addition of dry toluene (4 mL). The mixture was stirred at 90 °C for 24 h and usual work up and flash chromatography afforded **32** as a yellow-brown oil (457 mg, 67%). **δ_H** (400 MHz, $CDCl_3$): 5.80 (s, 1H, NH), 6.60 (dd, *J* = 8.1, 1.8 Hz, 1H, H-4), 6.74 (t, J = 2.2 Hz, 1H, H-2), 6.87 (dd, *J* = 8.1, 2.1 Hz, 1H, H-6), 7.02 – 7.05 (m, 4H, H-8 and H-8', H-12 and H-12'), 7.23 – 7.30 (m, 3H, H-13, H-13' and H-5), 8.20 (d, *J* = 9.2 Hz, 2H, H-9 and H-9'). **δ_C** (100 MHz, $CDCl_3$): 108.8 (CH Ar, C-2), 112.6 (CH Ar, C-4), 114.0 (CH Ar, C-6), 117.3 (2 CH Ar, C-8 and C-8' or C-12 and C-12'), 120.3 (2 CH Ar, C-8 and C-8' or C-12 and C-12'), 126.1 (2 CH Ar, C-9 and C-9'), 127.0 (qC, C-14), 129.6 (2 CH Ar, C-13 and C-13'), 131.2 (CH Ar, C-5), 140.7 (qC), 142.8 (qC), 145.4 (qC), 156.0 (qC), 163.3 (qC). **HRMS** (m/z ESI^-): Found: 339.0544 (M^- - H. $C_{18}H_{12}ClN_2O_3$ Requires: 339.0536). **ν_{max}** (ATR)/cm^{-1}: 3386 (NH), 3078, 2924, 2853, 1724, 1578, 1511, 1482 (NO_2), 1339 (NO_2), 1234 (CF_3), 1164, 1141, 1110, 1091 (C-Cl), 998, 847, 749.

4-chloro-*N*-(3-(4-nitrophenoxy)phenyl)-3-(trifluoromethyl)aniline (33)

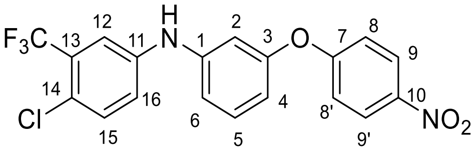

Following Method D, $Pd_2(dba)_3$ (3mol%, 55 mg), BINAP (3 mol%, 37 mg), compound **31** (460 mg, 2 mmol), NaO^tBu (269 mg, 2.8 mmol) and remaining liquid 4-bromo-1-chloro-2-(trifluoromethyl)benzene (0.28 mL, 2 mmol) were mixed, followed by syringe addition of dry toluene (4 mL). The mixture was stirred at 90 °C for 24 h and usual work up and flash chromatography afforded **33** as a yellow solid (713 mg, 87%). **Mp**: 103-105 °C. **δ_H** (600 MHz, $CDCl_3$): 5.99 (s, 1H, NH), 6.70 (dd, *J* = 8.1, 2.1 Hz, 1H, H-4), 6.77 (s, 1H, H-2), 6.92 (dd, *J* = 8.1, 1.5 Hz, 1H, H-6), 7.05 (d, *J* = 9.3 Hz, 2H, H-8 and H-8'), 7.16 (dd, *J* = 8.7, 2.6 Hz, 1H, H-16), 7.33 (t, *J* = 8.1 Hz, 1H, H-5), 7.35 – 7.37 (m, 2H, H-12 and H-15), 8.21 (d, *J* = 9.3 Hz, 2H, H-9 and H-9'). **δ_C** (150 MHz, $CDCl_3$): 109.8 (CH Ar, C-2), 113.7 (CH Ar, C-4), 114.8 (CH Ar, C-6), 116.7 (q, *J* = 5.5 Hz, CH Ar, C-12), 117.4 (2 CH Ar, C-8 and C-8'), 121.5 (CH Ar, C-16), 122.6 (d, *J* = 273.2 Hz, qCF_3), 123.6 (qC, C-14), 126.0 (2 CH Ar, C-9 and C-9'), 129.2 (d, *J* = 31.2 Hz, qC, C-13), 131.3 (CH Ar, C-5), 132.4 (CH Ar, C-15), 141.3 (qC), 142.9 (qC), 143.9 (qC), 156.1 (qC), 162.9 (qC). **HRMS** (m/z ESI^-): Found: 407.0422 (M^- - H. $C_{19}H_{11}ClF_3N_2O_3$ Requires: 407.0416). **ν_{max}** (ATR)/cm^{-1}: 3381 (NH), 1481 (NO_2), 1338, 1328, 1236 (C-O), 1131 (CF_3), 1110 (C-N), 847 (C-Cl),749.

3-(4-nitrophenoxy)-*N*-(3-(trifluoromethyl)phenyl)aniline (34)

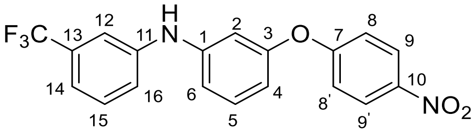

Following Method D, $Pd_2(dba)_3$ (3 mol%, 55 mg), BINAP (3 mol%, 37 mg), compound **31** (460 mg, 2 mmol), NaO^tBu (269 mg, 2.8 mmol) and remaining liquid 1-bromo-3-(trifluoromethyl)benzene (0.28 mL, 2 mmol) were mixed, followed by syringe addition of dry toluene (4 mL). The mixture was stirred at 90 °C for 24 h and usual work up and flash chromatography afforded **34** as an orange solid (464 mg, 62%). **Mp**: 111-113 °C. **δ_H** (600 MHz, $CDCl_3$): 5.93 (bs, 1H, NH), 6.69 (dd, *J* = 8.1, 2.1 Hz, 1H, H-4), 6.80 (s, 1H, H-2), 6.94 (dd, *J* = 8.1, 1.8, 1H, H-6), 7.06 (d, *J* = 9.1 Hz, 2H, H-8 and H-8'), 7.19 (d, *J* = 7.8 Hz, 1H, H-14), 7.24 (d, *J* = 8.0 Hz, 1H, H-16), 7.30 (s, 1H, H-12), 7.33 (t, *J* = 8.1 Hz, 1H, H-5 or H-15), 7.38 (t, *J* = 7.9 Hz, 1H, H-5 or H-15), 8.21 (d, *J* = 9.1 Hz, H-9 and H-9'). **δ_C** (150 MHz, $CDCl_3$): 109.8 (CH Ar, C-2), 113.4 (CH Ar, C-4), 114.6 (q, *J* = 7.6 Hz, CH Ar, C-12), 114.8 (CH Ar, C-6), 117.5 (2 CH Ar, C-8 and C-

8’), 118.3 (q, J = 7.5 Hz, CH Ar, C-14), 121.2 (CH Ar, C-16), 124.1 (d, J = 272.4 Hz, qCF_3), 126.1 (2 CH Ar, C-9 and C-9’), 130.2 (CH Ar, C-5 or C-15), 131.3 (CH Ar, C-5 or C-15), 132.1 (q, J = 32.2 Hz, qC, C-13), 142.96 (qC), 142.97 (qC), 144.4 (qC), 156.2 (qC), 163.2 (qC). **HRMS** (m/z ESI^-): Found 373.0816 (M^- - H. $C_{19}H_{12}F_3N_2O_3$ Requires: 373.0806). ν_{max} (ATR)/cm^{-1}: 3377 (NH), 1598 (NO_2), 1528, 1476 (NO_2), 1413, 1340 (CF_3), 1243 (C-O), 1165, 1122 (C-N), 978, 848, 793.

4'-nitro-3-(3-pentafluorosulfanylphenylamino]diphenylether (35)

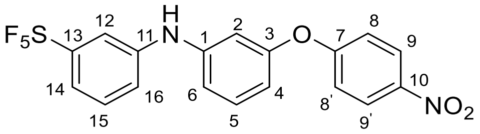

Following Method D, $Pd_2(dba)_3$ (3 mol%, 14 mg), BINAP (3 mol%, 9 mg), compound **31** (115 mg, 0.5 mmol), NaO^tBu (67 mg, 0.7 mmol) and remaining liquid (3-bromophenyl)sulfur pentafluoride (0.08 mL, 0.5 mmol) were mixed, followed by syringe addition of dry toluene (1 mL). The mixture was stirred at 90 °C for 24 h and usual work up and flash chromatography afforded **35** as a yellow solid (151 mg, 70%). **Mp**: 114-116 °C. δ_H (400 MHz, $CDCl_3$): 6.00 (s, 1H, NH), 6.70 (dd, J = 8.1, 2.3, Hz, 1H, H-4), 6.79 (t, J = 2.2 Hz, 1H, H-2), 6.93 (dd, J = 8.1, 2.1 Hz 1H, H-6), 7.06 (d, J = 9.3 Hz, 2H, H-8 and H-8’), 7.18 – 7.22 (m, 1H, H-16), 7.29 – 7.37 (m, 3H, H-5, H-15 and H-14) 7.44 (t, 1H, H-12), 8.21 (d, J = 9.3 Hz, 2H, H-9 and H-9’). δ_C (100 MHz, $CDCl_3$): 109.8 (CH Ar, C-2), 113.7 (CH Ar, C-4), 114.8 (CH Ar, C-6), 115.6 (p, J = 4.8 Hz, CH Ar, C-12), 117.5 (2 CH Ar, C-8 and C-8’), 118.9 (p, J = 4.5 Hz, CH Ar, C-14), 120.8 (CH Ar, C-16), 126.1 (2 CH Ar, C-9 and C-9’), 129.7 (CH Ar, C-15 or C-5), 131.4 (CH Ar, C-15 or C-5), 142.93 (qC), 142.95 (qC), 144.2 (qC), 155.0 (m, qC, C-13), 156.2 (qC), 163.1 (qC). **HRMS** (m/z ESI^-): Found 431.0488 (M^- - H. $C_{18}H_{12}F_5N_2O_3S$ Requires: 431.0489). ν_{max} (ATR)/cm^{-1}: 3383 (NH), 3079, 2927, 1597 (NO_2), 1580 (NO_2), 1483, 1340 (C-N), 1236 (C-O), 821 (SF_5), 833 (SF_5), 804 (SF_5).

1-Bromo-3-fluoro-5-(4-nitrophenoxy)benzene (37)

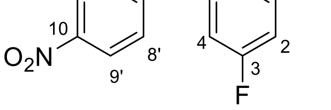

1-Fluoro-4-nitrobenzene (2.00 g, 14.17 mmol, 1 eq.), 3-bromo-5-fluorophenol (2.71 g, 14.17 mmol, 1 eq.) and K_2CO_3 (2.35 g, 17.00 mmol, 1.2 eq.) were dissolved in DMF (30 mL) and stirred at 80 ˚C for 12 h. The mixture was cooled to room temperature, washed with water

and the organic layer extracted with EtOAc, washed with brine, dried over $MgSO_4$, concentrated under vacuum and purified by flash chromatography (hexanes:EtOAc) to get **37** as a yellow solid (4.42 g, 100%). **Mp**: 65-67 °C. **δ_H** (400 MHz, $CDCl_3$): 6.77 (dt, 1H, *J* = 9.2, 2.2, H-4), 7.04 (dd, 1H, *J* = 2.9, 1.8, H-6), 7.07-7.11 (d, *J* = 9.2 Hz, 2H, H-8 and H-8'), 7.12 – 7.15 (m, 1H, H-2), 8.26 (d, *J* = 9.2 Hz, 2H, H-9' and H-9'). **δ_C** (100 MHz, $CDCl_3$): 107.3 (d, *J* = 24.4, CH Ar, C-4), 116.1 (d, *J* = 24.4, CH Ar, C-2), 118.4 (2 CH Ar, C-8 and C-8'), 119.3 (d, *J* = 3.6, CH Ar, C-6), 123.6 (d, *J* = 11.7, qC, C-1), 126.3(2 CH Ar, C-9 and C-9'), 143.9 (qC, C-7), 156.9 (d, *J* = 11.7, qC, C-5), 161.6 (qC, C-10), 163.3 (d, *J* = 252.8, qC, C-3). **HRMS** (m/z $APCI^-$): Found: 309.9526 (M^- - H. $C_{12}H_6BrFNO_3$ Requires: 309.9521). **ν_{max}** (ATR)/cm^{-1}: 3080, 2922, 1580 (NO_2), 1342 (NO_2), 1237 (C-F), 1221 (C-O), 1122 (C-Br), 838, 848.

4-chloro-*N*-(3-fluoro-5-(4-nitrophenoxy)phenyl)-3-(trifluoromethyl)aniline (38)

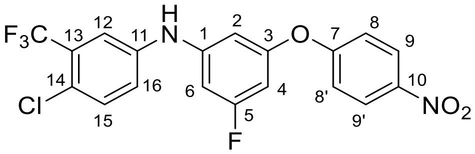

Following a modification of Method D, an oven-dried round bottom flask was charged with a magnetic stir bar, $Pd_2(dba)_3$ (3 mol%, 14 mg), BINAP (3 mol%, 9 mg), 4-chloro-3-(trifluoromethyl)aniline (98 mg, 0.5 mmol, 1 eq.) and NaO^{t}Bu (67 mg, 0.7 mmol, 1.4 eq.). The tube was evacuated and refilled with argon (three times). Under a counterflow of argon, **37** (156 g, 0.5 mmol, 1 eq.) in dry toluene (1 mL) was added dropwise to the mixture, followed by additional dry toluene (1 mL) by syringe. The tube was placed in a preheated oil bath at 90 °C and stirred overnight. The reaction mixture was then cooled to room temperature, diluted with EtOAc, filtered through a pad of Celite®, and washed with water. The organic layer was washed with brine, dried over $MgSO_4$, concentrated under vacuum and purified by flash chromatography (silica gel, hexanes:EtOAc) to obtain **38** as a yellow solid (139 mg, 65 %). **Mp**: 133-137 °C. **δ_H** (400 MHz, $CDCl_3$): 5.93 (bs, 1H NH), 6.39 (dt, *J* = 9.2, 2.1 Hz, 1H, H-4), 6.50 (s, 1H, H-2), 6.60 (dt, *J* = 10.2, 2.1 Hz, 1H, H-6), 7.10 (d, *J* = 9.2 Hz, 2H, H-8 and H-8'), 7.21 (dd, *J* = 8.6, 2.7 Hz, 1H, H-16), 7.39 (d, *J* = 2.6 Hz, 1H, H-12), 7.41 (d, *J* = 8.6 Hz, H-15), 8.24 (d, *J* = 9.2 Hz, 2H, H-9 and H-9'). **δ_C** (100 MHz, $CDCl_3$): 100.9 (d, *J* = 25.0 Hz, CH Ar, C-4), 100.91 (d, *J* = 25.0 Hz, CH Ar, C-6), 104.4 (d, *J* = 3.0 Hz, CH Ar, C-2), 118.1 (2 CH Ar, C-8 and C-8'), 118.2 (q, *J* = 5.5 Hz, CH Ar, C-12), 122.7 (d, *J* = 273.7, qCF_3), 123.0 (CH Ar, C-16), 125.1 (qC, C-14), 126.2 (2 CH Ar, C-9 and C-9'), 132.7 (CH Ar, C-15), 140.3 (qC), 143.5 (qC), 145.3 (d, *J* = 12.6 Hz, qC,

C-1 or C-3), 157.4 (d, J = 13.4 Hz, qC, C-1 or C-3), 162.2 (qC), 164.6 (d, J = 247.1 Hz, qC, C-5). **HRMS** (m/z ESI$^-$): Found: 425.0319 (M$^-$ - H. $C_{19}H_{10}N_2O_3ClF_4$ Requires: 425.0316). **ν_{max}** (ATR)/cm^{-1}: 3359 (NH), 3085, 1592 (NO_2), 1480 (NO_2), 1340 (C-F), 1238 (CF_3), 1180 (C-O), 1156, 1143, 1131 (C-N), 1128, 1020 (C-Cl), 845, 828, 661.

1.3.3. Nitropyridinyl derivatives

2-nitro-5-(3-nitrophenoxy)pyridine (54)[6,7]

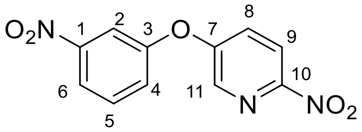

To a mixture of 5-bromo-2-nitropyridine (100 mg, 0.49 mmol, 1 eq.) and Cs_2CO_3 (241 mg, 0.74 mmol, 1.5 eq.) in DMF (10 mL), a solution of 3-nitrophenol (75 mg, 0.54 mmol, 1.1 eq.) in DMF (5 mL) was added dropwise and the reaction mixture was stirred at room temperature for 12 h. The reaction was then diluted with water and extracted with ethyl acetate. The organic layer was washed with 5% aqueous $NaHCO_3$ and brine and dried over anhydrous $MgSO_4$. The residue was purified by basic silica gel chromatography (hexanes:EtOAc), and the desired fractions were concentrated under reduced pressure to give the product **54** as a yellow solid (47 mg, 37%). **Mp**: 102-103 °C (lit. 113-114 °C).[6,7] **δ_H** (400 MHz, $CDCl_3$): 7.47 (dd, J = 8.1, 2.0 Hz, 1H, H-4), 7.54 (dd, J = 8.9, 2.7 Hz, 1H, H-8), 7.67 (t, J = 8.2 Hz, 1H, H-5), 7.98 (s, 1H, H-2), 8.16 (d, J = 8.2 Hz, 1H, H-6), 8.32 (d, J = 8.9 Hz, 1H, H-9), 8.39 (d, J = 2.7 Hz, 1H, H-11).

3-((6-nitropyridin-3-yl)oxy)aniline (66)

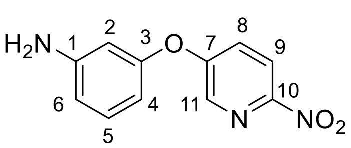

5-bromo-2-nitropyridine (500 mg, 2.45 mmol, 1eq.), 3-aminophenol (295 mg, 2.7 mmol, 1.1 eq.) and Cs_2CO_3 (1200 mg, 3.7 mmol, 1.5 eq.) were dissolved in DMF (5 mL) and stirred at 80 ˚C for 12 h. The mixture was cooled to room temperature, washed with water and the organic layer extracted with EtOAc, washed with brine, dried over $MgSO_4$, concentrated under vacuum and purified by flash chromatography (hexanes:EtOAc) to get **66** as a yellow solid (311 mg, 55 %). **Mp**: 118-120 °C. **δ_H** (400 MHz, $CDCl_3$): 6.40 (s, 1H, H-2), 6.45 (d, J = 8.0 Hz, H-

4), 6.58 (d, *J* = 8.1 Hz, H-6), 7.20 (t, *J* = 8.1 Hz, H-5), 7.42 (dd, *J* = 8.9, 2.8 Hz, H-8), 8.22 (d, *J* = 8.6 Hz, 1H, H-9), 8.32 (d, *J* = 2.3 Hz, 1H, H-11). **δ_C** (150 MHz, $CDCl_3$): 106.6 (CH Ar, C-2), 109.7 (CH Ar, C-4), 112.7 (CH Ar, C-6), 120.0 (CH Ar, C-9), 125.7 (CH Ar, C-8), 131.3 (CH Ar, C-5), 139.0 (CH Ar, C-11), 149.0 (qC), 155.4 (qC), 159.1 (qC), 171.3 (qC). **HRMS** (*m/z* – ESI^+): Found: 232.0715 (M^+ + H. C11H10N3O3 Requires: 232.0717). **ν_{max}** (ATR)/cm^{-1}: 3473 (NH), 3378 (NH), 3219, 3054, 292, 1624, 1604, 1521 (NO_2), 1488, (NO_2), 1455, 1344 (C-N), 1298, 1238 (C-O), 1142, 1114, 995, 875, 850, 686, 674.

4-chloro-*N*-(3-((6-nitropyridin-3-yl)oxy)phenyl)-3-(trifluoromethyl)aniline (67)

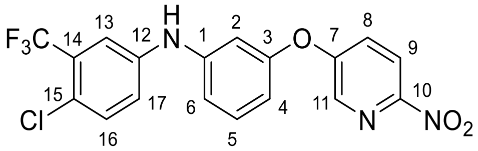

Following Method D, $Pd_2(dba)_3$ (3 mol%, 6 mg), Xantphos (3 mol%, 4 mg), compound **66** (50 mg, 0.22 mmol), Cs_2CO_3 (100 mg, 0.31 mmol) and remaining liquid 4-bromo-1-chloro-2-(trifluoromethyl)benzene (0.03 mL, 0.22 mmol) were mixed, followed by syringe addition of dry toluene (0.5 mL). The mixture was stirred at 90 °C for 24 h and usual work up and flash chromatography afforded **67** as a yellow oil (82 mg, 91%). **δ_H** (400 MHz, $CDCl_3$): 6.01 (bs, 1H, NH), 6.70 (dd, *J* = 8.1, 2.2 Hz, 1H, H-4), 6.79 (t, *J* = 2.2 Hz, 1H, H-2), 6.96 (dd, *J* = 7.9, 1.8 Hz, 1H, H-6), 7.18 (dd, *J* = 8.5, 2.8 Hz, 1H, H-17), 7.34 – 7.39 (m, 3H, H-13, H-16 and H-5), 7.46 (dd, *J* = 8.9, 2.8 Hz, 1H, H-8), 8.24 (d, *J* = 8.9 Hz, 1H, H-9), 8.33 (d, *J* = 2.8 Hz, 1H, H-11). **δ_C** (100 MHz, $CDCl_3$): 109.4 (CH Ar, C-2), 113.2 (CH Ar, C-4), 115.3 (CH Ar, C-6), 117.2 (q, *J* = 5.5 Hz, CH Ar, C-13), 119.9 (CH Ar, C-9), 122.0 (CH Ar, C-17), 122.7 (d, *J* = 273.4 Hz, qCF_3), 124.1 (qC, C-15), 126.0 (CH Ar, C-8), 129.4 (d, *J* = 31.2 Hz, qC, C-14), 131.7 (CH Ar, C-5 or C-16), 132.6 (CH Ar, C-5 or C-16), 138.8 (CH Ar, C-11), 141.1 (qC), 144.5 (qC), 151.3 (qC), 155.5 (qC), 158.8 (qC). **HRMS** (m/z ESI^+): Found: 410.0520 (M^+ + H. $C_{18}H_{12}N_3O_3ClF_3$ Requires: 410.0519). **ν_{max}** (ATR)/cm^{-1}: 3385 (NH), 3064, 2924, 1724, 1599, 1567, 1531 (NO_2), 1483 (NO_2), 1459, 1347, 1332, 1236 (CF_3), 1130 (C-O), 1111 (C-Cl), 1028, 999, 975, 868, 823, 786.

1.3.4. Aminoaryl and aminopyridinyl intermediates

2-(3-aminophenoxy)pyridin-5-amine (53)[6,7]

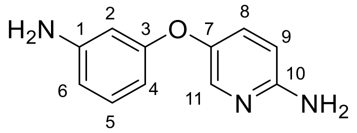

Following Method E, to a solution of nitro derivative **54** (781 mg, 2.99 mmol) in methanol/ tetrahydrofuran/ethyl acetate (5:1:1, 78 mL) was added 10% Pd/C mixture (10 mol%, 318 mg). After 24 h, usual work up followed by silica gel chromatography afforded **53** as a brown solid (566 mg, 94%). **Mp**: 80 °C.[6,7] **δ_H** (400 MHz, $CDCl_3$): 3.68 (s, 2H, NH_2), 4.37 (s, 2H, NH_2), 6.24 (s, 1H, H-2), 6.30 (d, *J* = 8.1 Hz, 1H, H-4 or H-6), 6.36 (d, *J* = 7.9 Hz, 1H, H-4 or H-6), 6.51 (d, *J* = 8.7 Hz, 1H, H-9), 7.05 (t, *J* = 8.1 Hz, 1H, H-5), 7.20 (dd, *J* = 8.8, 2.7 Hz, 1H, H-8), 7.91 (d, *J* = 2.4 Hz, 1H, H-11). **HRMS** (m/z ESI$^+$): Found: 202.0964 (M^+ + H. $C_{11}H_{12}N_3O$ Requires: 202.0975).

***N*-(3-(4-aminophenoxy)phenyl)-3-trifluoromethylaniline (41)**

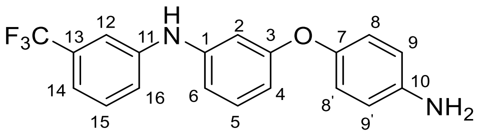

Following Method E, to a solution of nitro derivative **34** (409 mg, 1.1 mmol) in absolute ethanol (20 mL) was added 10% Pd/C mixture (10 mol%, 117 mg). After 24 h, usual work up followed by silica gel chromatography afforded **41** as brown oil (375 mg, 99 %). **δ_H** (400 MHz, $CDCl_3$): 3.61 (bs, NH_2, 2H), 5.84 (bs, 1H, NH), 6.57 (dd, *J* = 8.0, 1.9 Hz, 1H, H-4), 6.64 (t , *J* = 2.2 Hz, 1H, H-2), 6.68 (d, *J* = 8.7, 2H, H-9 and H-9'), 6.73 (dd, *J* = 8.0, 1.5 Hz, 1H, H-6), 6.89 (d, *J* = 8.7 Hz, 2H, H-8 and H-8'), 7.11 – 7.21 (m, 3H, H-5 or H-15, H-14 and H-16), 7.25 (s, 1H, H-12), 7.32 (t, *J* = 7.9 Hz, H-5 or H-15). **δ_C** (100 MHz, $CDCl_3$): 107.2 (CH Ar, C-2), 110.9 (CH Ar, C-4), 112.2 (CH Ar, C-6), 113.7 (q, *J* = 3.9 Hz, CH Ar, C-12), 116.4 (2 CH Ar, C-9 and C-9'), 117.3 (q, *J* = 3.9 Hz, CH Ar, C-14), 120.4 (CH Ar, C-16), 121.5 (2 CH Ar, C-8 and C-8'), 124.2 (d, *J* = 272.6 Hz, qCF_3), 130.0 (CH Ar, C-5 or C-15), 130.5 (CH Ar, C-5 or C-15), 131.8 (d, *J* = 32 Hz, qC, C-13), 143.1 (qC), 143.3 (qC), 143.7 (qC), 148.2 (qC), 160.4 (qC). **HRMS** (m/z ESI$^+$): Found 345.1211 (M^+ + H. $C_{19}H_{16}F_3N_2O$ Requires: 345.1209). **ν_{max}** (ATR)/cm^{-1}: 3316 (NH), 1594 (C-C), 1505, 1333 (C-O), 1217, 1173 (C-N), 1124 (C-Cl), 702.

***N*-(3-(4-aminophenoxy)phenyl)-3-pentafluorosulfanylaniline (42)**

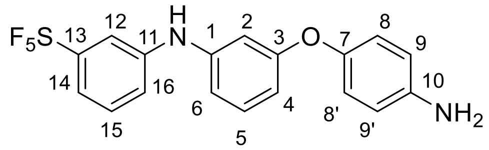

Following Method E, to a solution of nitro derivative **35** (75 mg, 0.17 mmol) in absolute ethanol (6 mL) was added 10% Pd/C mixture (10 mol%, 18 mg). After 24 h, usual work up followed by silica gel chromatography afforded **42** as a brown oil (41 mg, 60%). **δ_H** (400 MHz, $CDCl_3$): 3.60 (s, 2H, NH_2), 5.86 (s, 1H, NH), 6.58 (dd, *J* = 8.2, 2.2 Hz, 1H, H-4), 6.62 (t, *J* = 2.2 Hz, 1H, H-2), 6.68 (d, *J* = 8.7 Hz, 1H, H-9 and H-9'), 6.72 (dd, *J* = 8.0, 1.9 Hz, 1H, H-6), 6.89 (d, *J* = 8.7 Hz, 2H, H-8 and H-8'), 7-12 – 7.15 (m, 1H, H-16), 7.19 (t, *J* = 8.1 Hz, 1H, H-5 or H-15), 7.23 – 7.31 (m, 2H, H-15 or H-5, and H-14), 7.39 (t, *J* = 2.0 Hz, 1H, H-12). **δ_C** (100 MHz, $CDCl_3$): 107.3 (CH Ar, C-2), 111.2 (CH Ar, C-4), 112.2 (CH Ar, C-6), 114.7 (m, CH Ar, C-12), 116.4 (2 CH Ar, C-9 and C-9'), 118.0 (m, CH Ar, C-14), 120.0 (CH Ar, C-16), 121.5 (2 CH Ar, C-8 and C-8'), 129.5 (CH Ar, C-15 or C-5), 130.6 (CH Ar, C-15 or C-5), 143.1 (qC), 143.2 (qC), 143.7 (qC), 148.2 (qC), 155.1 (m, qC, C-13), 160.5 (qC). **HRMS** (m/z ESI^+): Found 403.0909 (M^+ + H. $C_{18}H_{16}F_5N_2OS$ Requires: 403.0904). **ν_{max}** (ATR)/cm^{-1}: 3491 (NH_2), 3404 (NH_2), 2924, 1595, 1488 (C-N), 1212 (C-O), 829 (SF_5), 806 (SF_5), 769, 681, 667.

***N*-(3-(4-aminophenoxy)phenyl)-4-chloro-3-(trifluoromethyl)aniline (40)**

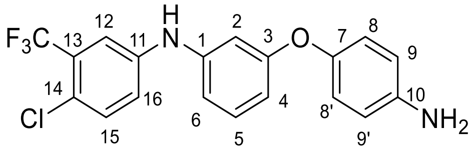

Following Method F, a mixture of **33** (570 mg, 1.32mmol) and $SnCl_2{\cdot}2H_2O$ (1890 mg, 8.37 mmol) in 3 mL of absolute ethanol and few drops of EtOAc to increase solubility, were heated at 70°C for 3 h. Usual work up and flash chromatography afforded **40** as a brown solid (340 mg, 68%). **Mp**: 102-104 °C. **δ_H** (600 MHz, $CDCl_3$): 3.58 (s, 2H, NH_2), 5.80 (s, 1H, NH), 6.58 – 6.61 (m, 2H, H-4 and H-2), 6.67 – 6.71 (m, 3H, H-9, H-9' and H-6), 6.89 (d, *J* = 8.7 Hz, 2H, H-8 and H-8'), 7.09 (dd, *J* = 8.6, 2.6 Hz, 1H, H-16), 7.20 (t, *J* = 8.1 Hz, 1H, H-5), 7.30 – 7.32 (m, 2H, H-12 and H-15). **δ_C** (150 MHz, $CDCl_3$): 107.4 (CH Ar, C-2), 111.3 (CH Ar, C-4), 112.4 (CH Ar, C-6), 116.0 (q, *J* = 5.4 Hz, CH Ar, C-12), 116.4 (2 CH Ar, C-9 and C-9'), 120.9 (CH Ar, C-16), 122.0 (2 CH Ar, C-8 and C-8'), 122.7 (qC C-14), 122.9 (q, *J* = 273.4 Hz, qCF_3), 129.1 (q, *J* = 31.2 Hz, qC, C-13), 130.7 (CH Ar, C-5), 132.4 (CH Ar, C-15), 142.2 (qC, C-11), 142.9 (qC, C-1), 143.2 (qC, C-10),

148.1 (qC, C-7), 160.5 (qC, C-3). **HRMS** (m/z ESI$^+$): Found: 379.0824 (M^+ + H. $C_{19}H_{15}ClF_3N_2O$ Requires: 379.0820). **ν$_{max}$** (ATR)/cm^{-1}: 3383 (NH), 2926, 1725, 1594, 1504 (C-N), 1482 (C-N), 1329, 1250, 1172 (C-Cl), 1130 (C-O), 1112 (CF_3), 825, 681, 666.

N-(3-(4-aminophenoxy)phenyl)-4-chloroaniline (39)

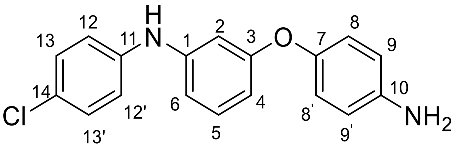

Following Method F, a mixture of **32** (208 mg, 0.61 mmol) and $SnCl_2·2H_2O$ (826 mg, 3.7 mmol) in 2 mL of absolute ethanol were heated at 70 °C for 3 h. Usual work up and flash chromatography afforded **39** as a brown solid (139 mg, 73%). **Mp**: 119-121 °C. **δ$_H$** (400 MHz, $CDCl_3$): 3.58 (bs, 2H, NH_2), 5.65 (bs, 1H, NH), 6.48 (dd, *J* = 8.2, 2.3 Hz, 1H, H-4), 6.61 (t, *J* = 2.2 Hz, 1H, H-2), 6.66 – 6.70 (m, 3H, H-6, H-9 and H-9’), 6.88 (d, *J* = 8.8 Hz, 2H, H-8 and H-8’), 6.98 (d, *J* = 8.8 Hz, 2H, H-12 and H-12’), 7.15 (t, *J* = 8.1 Hz, 1H, H-5), 7.20 (d, *J* = 8.8 Hz, 2H, H-13 and H-13’). **δ$_C$** (100 MHz, $CDCl_3$): 106.6 (CH Ar, C-2), 110.0 (CH Ar, C-4), 111.4 (CH Ar, C-6), 116.3 (2 CH Ar, C-9 and C-9’), 119.4 (2 CH Ar, C-12 and C-12’), 121.4 (2 CH Ar, C-8 and C-8’), 126.0 (qC, C-14), 129.4 (2 CH Ar, C-13 and C-13’), 130.4 (CH Ar, C-5), 141.5 (qC), 143.0 (qC), 144.3 (qC), 148.4 (qC), 160.3 (qC). **HRMS** (m/z ESI$^+$): Found 311.0954 (M^+ + H. $C_{18}H_{16}ClN_2O$ Requires: 311.0946). **ν$_{max}$** (ATR)/cm^{-1}: 3409 (NH), 3286 (NH), 3377 (NH), 1588, 1527, 1484 (C-O), 1324 (C-N), 1250, 1206, 1139 (C-Cl), 994, 972, 760.

N-(3-(4-aminophenoxy)-5-fluorophenyl)-4-chloro-3-(trifluoromethyl)aniline (43)

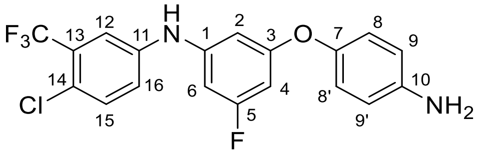

Following Method F, a mixture of **38** (139 mg, 0.33 mmol) and $SnCl_2·2H_2O$ (441 mg, 1.95 mmol) in 3 mL of absolute ethanol, were heated at 70 °C for 3 h. Usual work up and flash chromatography afforded **43** as a brown solid (92 mg, 70%). **Mp**: 113-115 °C. **δ$_H$** (400 MHz, $CDCl_3$): 3.63 (bs, 1H, NH_2), 5.83 (bs, 1H, NH), 6.25 (dt, *J* = 10.2, 2.1 Hz, 1H, H-4), 6.34 (s, 1H, H-2), 6.39 (dt, *J* = 10.2, 2.0 Hz, 1H, H-6), 6.69 (d, *J* = 8.7 Hz, 2H, H-9 and H-9’), 6.88 (d, *J* = 8.7 Hz, 2H, H-8 and H-8’), 7.14 (dd, *J* = 8.6, 2.7 Hz, 1H, H-16), 7.33 (d, *J* = 2.6 Hz, 1H, H-12), 7.35 (d, J

= 8.7 Hz, H-15). **δ$_C$** (100 MHz, $CDCl_3$): 98.2 (d, *J* = 25.6 Hz, CH Ar, C-4), 98.5 (d, *J* = 25.4 Hz, CH Ar, C-6), 101.8 (d, *J* = 2.1 Hz, CH Ar, C-2), 116.4 (2 CH Ar, C-9 and C-9'), 117.2 (q, *J* = 5.4 Hz, CH Ar, C-12), 121.8 (2 CH Ar, C-8 and C-8'), 122.0 (CH Ar, C-16), 122.8 (d, *J* = 273.3 Hz, qCF_3), 123.9 (qC, C-14), 129.3 (q, *J* = 31.2 Hz, qC, C-13), 132.5 (CH Ar, C-15), 141.2 (qC), 143.7 (qC), 144.1 (d, *J* = 12.7 Hz, qC, C-1 or C-3), 147.4 (qC), 161.8 (d, *J* = 13.3 Hz, qC, C-1 or C-3), 164.5 (d, *J* = 244.3 Hz, qC, C-5). **HRMS** (m/z ESI^-): Found: 395.0573 (M^-- H. $C_{19}H_{12}N_2OClF_4$ Requires: 395.0574). **ν$_{max}$** (ATR)/cm^{-1}: 3361 (NH), 3256 (NH), 1629, 1601, 1578, 1505, 1474, 1322 (CF_3), 1259, 1110 (C-F), 1004 (C-Cl), 822, 748, 670.

4'-Amino-3-[2-(*tert*-butoxycarbonyl)-3-(2,4-difluorophenyl)guanidino]phenoxypyridine (56)

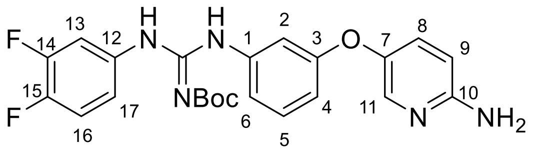

Following a modification of Method C, $HgCl_2$ (404 mg, 1.49 mmol) was added over a solution of **53** (250 mg, 1.24 mmol), thioureaderivative **13** (430 mg, 1.49 mmol) and NEt_3 (0.5 mL, 3.84 mmol) in CH_2Cl_2 (8 mL). After 6 h workup and silica gel chromatography (hexanes:EtOAc) afforded compound **56** as a yellow amorphous solid (254 mg, 45%). **Mp**: 119 - 121 °C. **δ$_H$** (600 MHz, $CDCl_3$): 1.48 (s, 9H, $(CH_3)_3$), 4.38 (s, 2H, NH_2), 6.47 – 6.67 (m, 4H, CH Ar), 7.09 (m, 2H, CH Ar), 7.05 – 7.13 (m, 1H, CH Ar), 7.22 (bs, 1H, CH Ar), 7.43 (bs, 1H, NH), 7.83 (bs, 1H, CH Ar), 7.92 (bs, 1H, CH Ar), 9.62 (s, 1H, NH). **δ$_C$** (150 MHz, $CDCl_3$): 28.2($(CH_3)_3$), 83.9 (qC, ($CH(CH_3)_3$), 109.4 (CH Ar), 111.1 (CH Ar), 111.4 (CH, Ar), 111.9 (CH, Ar), 115.5 (CH, Ar), 116.6 (CH, Ar), 117.0 (CH, Ar), 118.0 (CH, Ar), 129.9 (CH, Ar), 140.4 (CH Ar), 143.5 (qC), 145.3 (qC), 148.2 (qC), 149.2 (qC), 151.0 (qC), 153.1 (qC), 155.3 (qC), 159.1 (qC), 160.1 (qC). **HRMS** (m/z ESI^+): Found: 456.1855 (M^+ + H. $C_{23}H_{24}F_2N_5O_3$ Requires: 456.1842). **ν$_{max}$** (ATR)/cm^{-1}: 3422 (NH), 3304 (NH), 2978, 2932, 1723 (C=O), 1662, 1560, 1594 C=N), 1482, 1226 (C-F), 1144 (C-F), 1085.

4'-Amino-3-[2-(*tert*-butoxycarbonyl)-3-(2-fluoro-4-iodophenyl)guanidino]phenoxypyridine (57)

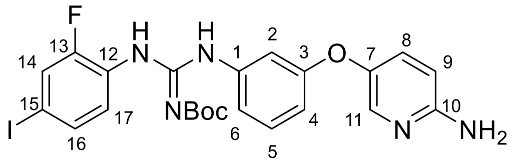

Following a modification of Method C, $HgCl_2$ (307 mg, 1.13 mmol) was added over a solution of **53** (190 mg, 0.94 mmol), thioureaderivative **14** (448 mg, 1.13 mmol) and NEt_3 (0.40 mL, 2.9 mmol) in CH_2Cl_2 (5 mL). After 6 h workup and silica gel chromatography (hexanes:EtOAc) afforded compound **57** as a yellow oil (185 mg, 35%). **δ_H** (400 MHz, DMSO-*d*$_6$): 1.27 (s, 9H, $(CH_3)_3$), 5.84 (s, 2H, NH_2), 6.46 – 6.49 (m, 2H, CH Ar and H-9), 6.66 (t, *J* = 8.5 Hz, 1H, H-5), 7.16 – 7.19 (m, 2H, CH Ar), 7.25 – 7.27 (m, 2H, CH Ar), 7.36 – 7.38 (m, 1H, CH Ar), 7.46 – 7.49 (m, 1H, H-14), 7.72 (d, *J* = 2.4 Hz, 1H, H-11), 9.08 (bs, 1H, NH), 9.31 (bs, 1H, NH). **δ_C** (150 MHz, DMSO-*d*$_6$): 27.7 ($(CH_3)_3$), 59.7 (qC, $\underline{C}(CH_3)_3$), 80.1 (qAr, C-15),107.3 (CH Ar), 108.6 (CH Ar, C-9), 110.0 (CH Ar), 113.0 (CH Ar), 124.0 (d, *J* = 21.5 Hz, CH Ar, C-14), 126.1 (CH Ar, C-5), 129.5 (CH Ar), 130.4 (CH Ar), 132.9 (CH Ar), 139.6 (CH Ar, C-11), 141.3 (qC), 142.7 (qC), 142.9 (qC), 151.9 (qC), 152.7 (d, *J* = 239.8 Hz, qAr, C-13), 156.9 (qC), 158.9 (qC), 170.3 (qC). **HRMS** (m/z ESI^+): Found: 564.0908 (M^+ + H. $C_{23}H_{24}FIN_5O_3$ Requires: 564.0902). **ν_{max}**(ATR)/cm^{-1}:3489 (NH), 3413 (NH), 3299, 3203 (NH), 2970, 2931, 1720 (C=O), 1661 (C=N), 1482 (C-F), 1462, 1224 (C-N), 1147 (C-O), 687 (C-I).

4'-Amino-3-[2-(*tert*-butoxycarbonyl)-3-(4-bromophenyl)guanidino]phenoxypyridine (58)

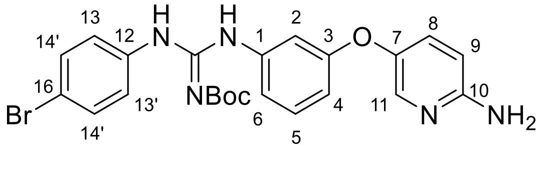

Following a modification of Method C, $HgCl_2$ (406 mg, 1.49 mmol) was added over a solution of **53** (250 mg, 1.24 mmol), thioureaderivative **15** (493 mg, 1.49 mmol) and NEt_3 (0.5 mL, 3.84 mmol) in CH_2Cl_2 (8 mL). After 6 h workup and silica gel chromatography (hexanes:EtOAc) afforded compound **58** as a yellow-brown amorphous solid (517 mg, 84%). **Mp**: 141 - 143 °C. **δ_H** (400 MHz, $CDCl_3$): 1.48 (s, 9H, $(CH_3)_3$), 6.53 – 6.57 (m, 2H, CH Ar and H-9), 6.61 – 6.68 (m, 2H, CH Ar), 7.20 - 7.24 (m, 3H, CH Ar), 7.37 - 7.42 (m, 3H, CH Ar), 7.87 (d, *J* = 2.7 Hz, 1H, H-11).

δC (150 MHz, $CDCl_3$): 28.2 ($(CH_3)_3$), 83.7 (qC, $\underline{C}(CH_3)_3$), 109.4 (CH Ar), 111.1 (CH Ar), 111.8 (CH Ar), 114.4 (CH Ar), 115.3 (CH Ar), 116.6 (q), 121.7 (CH Ar), 124.5 (CH Ar), 131.0 (CH Ar), 131.8 (CH Ar), 132.7 (CH Ar), 138.1(CH Ar), 140.5 (qC), 145.3 (qC), 148.4 (qC), 153.1 (qC), 155.3 (qC), 159.1 (qC), 160.1 (qC). **HRMS** (m/z ESI$^+$): Found: 498.1141 (M^+ + H. $C_{23}H_{25}BrN_5O_3$ Requires: 498.1135). **νmax** (ATR)/cm^{-1}: 3477 (NH), 3415 (NH), 3296 (NH), 3166, 2965, 1729 (C=O), 1655 (C=N), 1592, 1580, 1549, 1477, 1398 (C-N), 1145 (C-Br), 1067 (C-O), 823, 770.

4'-Amino-3-[2-(*tert*-butoxycarbonyl)-3-(4-bromo-3-trifluoromethylphenyl)guanidino] phenoxypyridine (59)

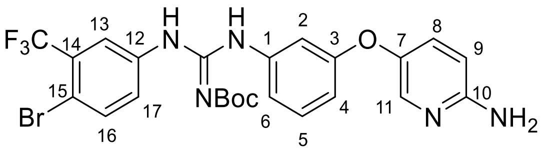

Following a modification of Method C, $HgCl_2$ (988 mg, 3.64 mmol) was added over a solution of **53** (610 mg, 3.03 mmol), thiourea derivative **16** (1455 mg, 3.64 mmol) and NEt_3 (1.30 mL, 9.39 mmol) in CH_2Cl_2 (16 mL). After 6 h workup and silica gel chromatography (hexanes:EtOAc) afforded compound **59** as a yellow oil (789 mg, 46%). **δH** (600 MHz, DMSO-*d6*): 1.25 (s, 9H, $(CH_3)_3$), 5.84 (s, 2H, NH_2), 6.47 – 6.49 (m, 2H, CH Ar), 6.94 (bs, 1H, CH Ar), 7.17 – 7.18 (m, 3H, CH Ar), 7.28 (bs, 1H, CH Ar), 7.68 – 7.83 (m, 3H, CH Ar), 9.12 (bs, 1H, NH), 9.36 (bs, 1H, NH). **δC** (100 MHz, DMSO-*d6*): 27.7 ($(CH_3)_3$), 79.0 (qC, $\underline{C}(CH_3)_3$), 80.3 (qC, C-15), 107.4 (CH Ar), 108.8 (CH Ar), 110.0 (CH Ar), 113.1 (CH Ar), 124.2 (CH Ar), 126.2 (CH Ar), 129.7 (CH Ar), 130.5 (CH Ar), 133.0 (CH Ar), 139.6 (CH Ar), 141.31 (qC), 141.33 (qC), 142.8 (qC), 143.0 (qC), 152.0 (qC), 157.0 (qC), 159.0 (qC), 170.5 (qC). **HRMS** (m/z ESI$^+$): Found: 566.1014 (M^+ + H. $C_{24}H_{24}BrF_3N_5O_3$ Requires: 566.1015). **νmax** (ATR)/cm^{-1}: 3476 (NH), 3413 (NH), 3297, 2981, 2935, 1720 (C=O), 1659 (C=N), 1588 (C=N), 1553 (C=N), 1476, 1230 (C-O), 1134 (CF_3), 1053 (C-Br), 826, 771.

4'-Amino-3-[2-(*tert*-butoxycarbonyl)-3-(4-chloro-3-(trifluoromethyl)phenyl)guanidino] phenoxypyridine (60)

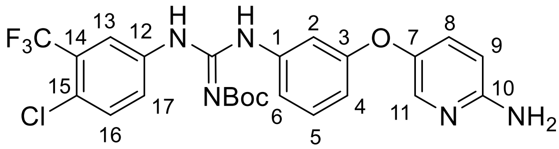

Following a modification of Method C, $HgCl_2$ (548 mg, 2.02 mmol) was added over a solution of **53** (338 mg, 1.68 mmol), thioureaderivative **17** (715 mg, 2.02 mmol) and NEt_3 (0.72 mL, 5.21 mmol) in CH_2Cl_2 (9 mL). After 6 h workup and silica gel chromatography (hexanes:EtOAc) afforded compound **60** as a yellow oil (368 mg, 42%). **δ_H** (400 MHz, $CDCl_3$): 1.49 (s, 9H, $(CH_3)_3$), 4.38 (bs, 2H, NH_2), 6.47 – 6.72 (m, 4H), 7.20 – 7.22 (m, 2H), 7.38 – 7.52 (m, 1H), 7.92 (bs, 2H), 9.66 (bs, 1H, NH), 9.80 (bs, 1H, NH). **δ_H** (400 MHz, $CDCl_3$): 28.2 ($(CH_3)_3$), 84.1 (qC, $\underline{C}(CH_3)_3$), 109.4, 111.0, 111.9, 116.4, 121.5, 123.9, 124.0, 130.9, 131.0, 132.6, 140.1, 140.4, 140.5, 155.3, 155.4. **HRMS** (m/z APCI$^-$): Found: 520.1391 (M^- - H. $C_{24}H_{22}N_5O_3ClF_3$ Requires: 520.1369). **ν_{max}** (ATR)/cm^{-1}: 3406 (NH), 3299 (NH), 3191 (NH), 2981, 2936, 1720 (C=O), 1659 (C=N), 1590, 1478, 1320, 1231 (C-O), 1135 (CF_3), 1088 (C-Cl), 1044, 826, 771, 666.

5-(3-((4-chloro-3-(trifluoromethyl)phenyl)amino)phenoxy)pyridin-2-amine (68)

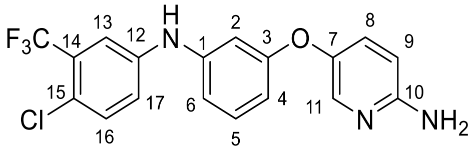

Following Method F, a mixture of **67** (66 mg, 0.16 mmol) and $SnCl_2\cdot 2H_2O$ (218 mg, 0.97 mmol) in 1 mL of absolute ethanol were heated at 70°C for 3 h. Usual work up and flash chromatography afforded **68** as a brown oil (48 mg, 80%). **δ_H** (400 MHz, $CDCl_3$): 4.41 (s, 2H, NH_2), 5.88 (s, 1H, NH), 6.53 (d, *J* = 8.8 Hz, 1H, H-9), 6.56 (dd, *J* = 8.3, 2.2 Hz, 1H, H-4), 6.61 (t, *J* = 2.0 Hz, 1H, H-2), 6.73 (dd, *J* = 8.0, 1.7 Hz, 1H, H-6), 7.11 (dd, *J* = 8.6, 2.7 Hz, 1H, H-17), 7.19 – 7.23 (m, 2H, H-5 and H-8), 7.32 – 7.34 (m, 2H, H-13 and H-16), 7.91 (d, *J* = 2.1 Hz, 1H, H-11). **δ_C** (100 MHz, $CDCl_3$): 107.0 (CH Ar, C-2), 109.4 (CH Ar, C-9), 110.8 (CH Ar, C-4), 112.6 (CH Ar, C-6), 116.2 (q, *J* = 5.4 Hz, CH Ar, C-13), 121.1 (CH Ar, C-17), 122.8 (d, *J* = 273.3 Hz, qCF_3), 123.0 (qC, C-15), 129.2 (d, *J* = 31.1 Hz, CH Ar, C-14), 130.8 (CH Ar, C-5 or C-8),131.1 (CH Ar, C-5 or C-8), 132.4 (CH Ar, C-16), 140.5 (CH Ar, C-11), 142.0 (qC), 143.2 (qC), 145.1 (qC), 155.4 (qC), 160.1 (qC). **HRMS** (*m/z* ESI$^-$): Found: 378.0629 (M^- - H. $C_{18}H_{12}N_3OF_3Cl$ Requires: 378.0621). **ν_{max}** (ATR)/cm^{-1}: 3332 (NH), 2923 (C-H), 2847 (C-H), 1595, 1480, 1327, 1258, 1225, 1172, 1125 (CF_3), 1111 (C-Cl), 820, 711, 665.

1.3.5. Boc-protected mono-guanidino phenyloxyaryl derivatives

3-Amino-[4'-(2,3-di-*tert*-butoxycarbonyl)guanidino]diphenylether (18)[1]

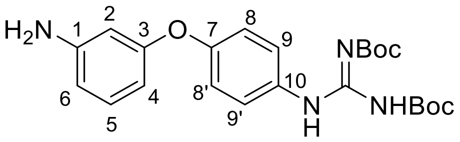

$HgCl_2$ (1200 mg, 4.42 mmol, 1.2 eq.) was added over a solution of **20** (2170 mg, 10.86 mmol, 3 eq.), *N,N'*-bis-(*tert*-butoxycarbonyl)thiourea (1000 mg, 3.62 mmol, 1 eq.) and triethylamine (1.56 mL, 11.22 mmol, 3.1 eq.) in CH_2Cl_2 (18 mL) at 0 °C. The resulting mixture was stirred at 0 °C for 1 h and then overnight at room temperature. When the reaction was adjudged complete by TLC, the organic phase was washed with brine, dried over $MgSO_4$, filtered, and concentrated under vacuum. The crude product was purified by silica gel chromatography (hexane:EtOAc) to afford the title product as a white solid (1201 mg, 75%). **Mp**: 78-79 °C (lit. 78-79 °C).[1] **δ_H** (400 MHz, $CDCl_3$): 1.50 (s, 9H, $(CH_3)_3$), 1.55 (s, 9H, $(CH_3)_3$), 3.68 (bs, 2H, NH_2), 6.30 (s, 1H, H-2), 6.38 – 6.40 (m, 2H, H-4 and H-6), 6.99 (d, 2H, *J*= 8.9, H-8 and H-8'), 7.08 (t, 1H, *J* = 8.0 Hz, H-5), 7.55 (d, 2H, *J* = 8.9 Hz, H-9 and H-9'), 10.28 (bs, 1H, NH), 11.64 (bs, 1H, NH).

4'-Amino-[3-(2,3-di-*tert*-butoxycarbonyl)guanidino]diphenylether (19)

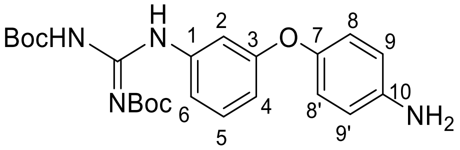

$HgCl_2$ (1200 mg, 4.42 mmol, 1.2 eq.) was added over a solution of **20** (2170 mg, 10.86 mmol, 3 eq.), *N,N'*-bis-(*tert*-butoxycarbonyl)thiourea (1000 mg, 3.62 mmol, 1 eq.) and triethylamine (1.56 mL, 11.22 mmol, 3.1 eq.) in CH_2Cl_2 (18 mL) at 0 °C. The resulting mixture was stirred at 0 °C for 1 h and then overnight at room temperature. When the reaction was adjudged complete by TLC, the organic phase was washed with brine, dried over $MgSO_4$, filtered, and concentrated under vacuum. The crude product was purified by silica gel chromatography (hexane:EtOAc) to afford the title product as a yellow oil (400 mg, 25%). **δ_H** (400 MHz, $CDCl_3$): 1.49 (s, 9H, $(CH_3)_3$), 1.52 (s, 9H, $(CH_3)_3$), 3.58 (bs, 2H, NH_2), 6.65 - 6.69 (m, 3H, H-9 and H-9' and H-4), 6.88 (d, 2H, *J*= 8.6, H-8 and H-8'), 7.17 - 7.24 (m, 3H,H-5, H-6 and H-2), 10.25 (bs, 1H, NH), 11.59 (bs, 1H, NH). **δ_C** (100 MHz, $CDCl_3$): 28.2 ($(CH_3)_3$), 28.3 ($(CH_3)_3$), 79.7 (qC, $\underline{C}(CH_3)_3$), 83.9 (qC, $\underline{C}(CH_3)_3$), 111.4 (CH Ar, C-2), 113.5 (CH Ar, C-4), 116.1 (CH Ar, C-6), 116.4 (2 CH Ar, C-9 and C-9'), 121.5 (2 CH Ar, C-8 and C-8'), 129.7 (CH Ar, C-5), 138.1 (qC), 142.9 (qC), 148.4 (qC), 153.4 (qC), 153.6 (qC), 159.4 (qC), 163.6 (qC). **HRMS** (*m/z* - ESI$^+$): Found: 443.2295 (M^+ + H.

$C_{23}H_{31}N_4O_5$ Requires: 443.2289. $\mathbf{v_{max}}$ (ATR)/cm^{-1}: 3474 (NH), 2976, 1719 (C=O), 1632 (C=N), 1507, 1410, 1213, 1145 (C-O).

1.3.6. Boc-protected precursor of final salts

4'-[2,3-Di-(*tert*-butoxycarbonyl)guanidino]-3-[2-(*tert*-butoxycarbonyl)-3-(4-fluorophenyl) guanidino]diphenylether (21)

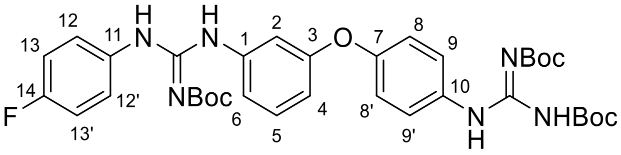

Following Method C, $HgCl_2$ (92 mg, 0.34 mmol) was added over a solution of **18** (124 mg, 0.28 mmol), thiourea derivative **12** (93 mg, 0.34 mmol) and NEt_3 (0.12 mL, 0.87 mmol) in CH_2Cl_2 (1.5 mL). After 12 h workup and silica gel chromatography (hexanes:EtOAc) afforded compound **21** as a yellow oil (108 mg, 57%). $\mathbf{\delta_H}$ (400 MHz, $CDCl_3$): 1.48 (s, 9H, $(CH_3)_3$), 1.49 ((s, 9H, $(CH_3)_3$), 1.53 (s, 9H, $(CH_3)_3$), 6.55-6.70 (m, 3H, CH Ar), 6.84 (bs, 1H, CH Ar), 6.98-6.70 (m, 4H, CH Ar), 7.24 (bs, 1H, CH Ar), 7.32-7.47 (bs, 1H, CH Ar), 7.56 (m, 2H, CH Ar), 9.50-9.55 (m, NH), 10.29 (bs, NH), 11.64 (bs, NH). $\mathbf{\delta_C}$ (100 MHz, $CDCl_3$): 28.2 ($2(CH_3)_3$), 28.3 ($(CH_3)_3$), 79.8 (qC, $\underline{C}(CH_3)_3$), 83.5 (qC, $\underline{C}(CH_3)_3$), 83.9 (qC, $\underline{C}(CH_3)_3$), 112.7 (CH Ar), 113.0 (CH Ar), 115.6 (CH Ar), 116.3 (CH Ar), 119.9 (CH Ar), 123.9 (CH Ar), 129.8 (CH Ar), 130.8 (CH Ar), 132.6 (qC), 140.5 (qC), 153.0 (qC), 153.5 (qC), 153.7 (qC), 157.9 (qC), 158.9 (qC), 163.7 (qC). **HRMS** (m/z ESI$^+$): Found: 679.3253 (M^+ + H. $C_{35}H_{44}N_6O_7F$ Requires: 679.3256). $\mathbf{v_{max}}$ (ATR)/cm^{-1}: 3412 (NH), 3308 (NH), 3263, 2979, 2931, 1719 (C=O), 1628, 1596 (C=N), 1234 (C-N), 1210, 1121 (C-O), 1145 (C-F), 1095, 832, 804, 771, 731, 687.

4'-[2,3-Di-(*tert*-butoxycarbonyl)guanidino]-3-[2-(*tert*-butoxycarbonyl)-3-(3,4-difluoro phenyl)guanidino]diphenylether (22)

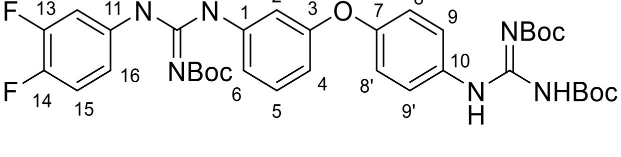

Following Method C, $HgCl_2$ (92 mg, 0.34 mmol) was added over a solution of **18** (124 mg, 0.28 mmol), thiourea derivative **13** (98 mg, 0.34 mmol) and NEt_3 (0.12 mL, 0.87 mmol) in CH_2Cl_2

(1.5 mL). After 12 h workup and silica gel chromatography (hexanes:EtOAc) afforded compound **22** as a white-yellow amorphous solid (120 mg, 61%). **Mp**: 125 – 127 °C. δ_H (400 MHz, $CDCl_3$): 1.48 (s, 9H, $(CH_3)_3$), 1.49 (s, 9H, $(CH_3)_3$), 1.54 (s, 9H, $(CH_3)_3$), 6.54 - 6.71 (m, 4H, CH Ar and NH), 7.00 (bs, 2H,CH Ar), 7.07 - 7.18 (m, 2H, CH Ar), 7.28 – 7.44 (bs, 1H, CH Ar), 7.56 - 7.58 (bs, 2H, CH Ar), 7.82 (bs, 1H, CH Ar), 9.62 (bs, NH), 10.30 (bs, NH), 11.64 (bs, NH). δ_C (100 MHz, $CDCl_3$): 28.2 ($2(CH_3)_3$), 28.3 ($(CH_3)_3$), 79.8 (qC, $\underline{C}(CH_3)_3$), 83.8 (qC, $\underline{C}(CH_3)_3$), 84.0 (qC, $\underline{C}(CH_3)_3$), 109.7 (CH Ar), 112.5 (CH Ar), 113.3 (CH Ar), 115.6 (CH Ar), 117.1 (CH Ar), 118.2 (CH Ar), 119.9 (2 CH Ar), 123.9 (2 CH Ar), 130.9 (CH Ar), 132.7 (qC), 140.1 (qC), 148.1 (qC), 153.0 (qC), 153.5 (qC), 153.7 (qC), 159.1 (qC), 163.7 (qC). **HRMS** (m/z ESI^+): Found: 697.3157 (M^+ + H. $C_{35}H_{43}F_2N_6O_7$ Requires: 697.3161). ν_{max}(ATR)/cm^{-1}: 3413 (NH), 3304, 3264 (NH), 2979, 2928, 2856, 1719 (C=O), 1627, 1596 (C=N), 1565, 1505, 1480, 1461, 1233 (C-N), 1210 (C-O), 1145 (C-F), 803, 773, 731.

4'-[2,3-Di-(*tert*-butoxycarbonyl)guanidino]-3-[2-(*tert*-butoxycarbonyl)-3-(2-fluoro-4-iodo phenyl)guanidino]diphenylether (23)

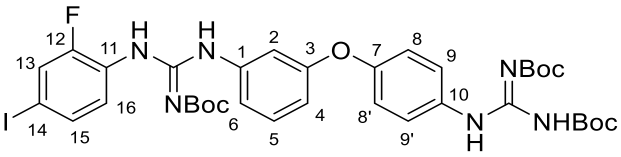

Following Method C, $HgCl_2$ (147 mg, 0.54 mmol) was added over a solution of **18** (200 mg, 0.45 mmol), thioureaderivative **14** (214 mg, 0.54 mmol) and NEt_3 (0.19 mL, 1.40 mmol) in CH_2Cl_2 (2.5 mL). After 12 h workup and silica gel chromatography (hexanes:EtOAc) afforded compound **23** as a white-pale yellow amorphous solid (220 mg, 61%). **Mp**: 79-81 °C. δ_H (400 MHz, $CDCl_3$): 1.49 (s, 18H, $(CH_3)_3$), 1.54 (s, 9H, $(CH_3)_3$), 6.54 - 6.71 (m, 3H, CH Ar), 6.99 (bs, 2H, CH Ar), 7.22 - 7.28 (bs, 2H, CH Ar), 7.38 - 7.42 (m, 1H, CH Ar), 7.56 - 7.58 (m, 2H, CH Ar), 8.32 (bs, 1H, CH Ar), 9.69 (m, NH), 9.90 (m, NH), 10.30 (bs, NH), 11.64 (bs, NH). δ_C (100 MHz, $CDCl_3$): 28.2 ($(CH_3)_3$), 28.3 ($(CH_3)_3$), 79.8 (qC, $\underline{C}(CH_3)_3$), 83.9 (qC, $\underline{C}(CH_3)_3$), 112.4 (CH Ar), 113.3 (CH Ar), 116.9 (CH Ar), 120.0 (2 CH Ar), 123.1 (CH Ar), 123.9 (2 CH Ar), 130.0 (CH Ar), 131.0 (CH Ar), 133.5 (CH Ar), 134.1 (qC), 152.9 (qC), 153.5 (qC), 153.7 (qC), 159.2 (qC), 163.7 (qC). **HRMS** (m/z ESI^+): Found: 805.2213 (M^+ + H. $C_{35}H_{43}FIN_6O_7$ Requires: 805.2216). ν_{max} (ATR)/cm^{-1}: 3450 (NH), 2977, 2935, 1718 (C=O), 1591 (C=N), 1407, 1367, 1234 (C-N), 1144 (C-F), 1112 (C-O), 1056, 772 (C-I).

4'-[2,3-Di-(*tert*-butoxycarbonyl)guanidino]-3-[2-(*tert*-butoxycarbonyl)-3-(4-bromophenyl) guanidino]diphenylether (24)

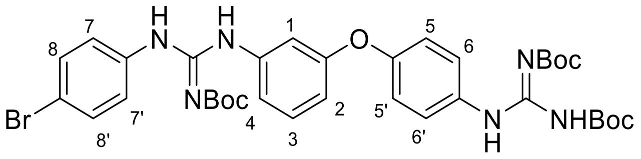

Following Method C, $HgCl_2$ (209 mg, 0.77 mmol) was added over a solution of **18** (283 mg, 0.64 mmol), thiourea derivative **15** (255 mg, 0.77 mmol) and NEt_3 (0.27 mL, 1.98 mmol) in CH_2Cl_2 (3.5 mL). After 12 h workup and silica gel chromatography (hexanes:EtOAc) afforded compound **24** as a white amorphous solid (170 mg, 36%). **Mp**: 116 - 119 °C. **δ_H** (600 MHz, $CDCl_3$): 1.48 (bs, 9H, $(CH_3)_3$), 1.49 (bs, 9H, $(CH_3)_3$), 1.54 (bs, 9H, $(CH_3)_3$), 6.55 - 6.78 (m, 4H, CH Ar), 6.99 – 7.01 (m, 2H, CH Ar), 7.39 – 7.43 (m, 3H, CH Ar), 7.57 (bs, 3H, CH Ar), 9.60 (bs, 1H, NH), 10.29 (bs, 1H, NH), 11.64 (bs, 1H, NH). **δ_C** (150 MHz, $CDCl_3$): 28.2 ($(CH_3)_3$), 28.3 ($(CH_3)_3$), 79.8 (qC, $\underline{C}(CH_3)_3$), 83.9 (qC, $\underline{C}(CH_3)_3$), 112.6 (CH Ar), 113.2 (CH Ar), 117.2 (CH Ar), 119.6 (CH Ar), 119.9 (CH Ar), 121.7 (CH Ar), 121.8 (CH Ar), 124.0 (CH Ar), 124.5 (CH Ar), 131.8 (CH Ar), 132.1 (CH Ar), 132.8 (CH Ar), 140.2 (qC), 145.7 (qC), 153.2 (qC), 153.5 (qC), 158.8 (qC), 163.4 (qC). **ν_{max}** (ATR)/cm^{-1}: 3412, 3260 (NH), 2980, 2933, 1719 (C=O), 1625, 1599 (C=N), 1551, 1481, 1459, 1481, 1408, 1368, 136, 1304, 1231, 1211, 1145 (C-O), 1113 (C-Br), 1097, 1057, 909, 731. **HRMS** (m/z ESI^+): Found: 739.2458 (M^+ + H. $C_{35}H_{44}N_6O_7Br$ Requires: 739.2455).

4'-[2,3-Di-(*tert*-butoxycarbonyl)guanidino]-3-[2-(*tert*-butoxycarbonyl)-3-(4-bromophenyl-3-trifluoromethyl) guanidino]diphenylether (25)

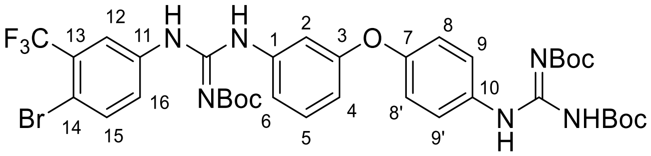

Following Method C, $HgCl_2$ (147 mg, 0.54 mmol) was added over a solution of **18** (200 mg, 0.45 mmol), thioureaderivative **16** (217 mg, 0.54 mmol) and NEt_3 (0.19 mL, 1.40 mmol) in CH_2Cl_2 (3 mL). After 12 h workup and silica gel chromatography (hexanes:EtOAc) afforded compound **25** as a white-pale yellow amorphous solid (207 mg, 57 %). **Mp**: 92-94 °C. **δ_H** (400 MHz, $CDCl_3$): 1.49 (s, 18H, $(CH_3)_3$), 1.54 (s, 9H, $(CH_3)_3$), 6.54-6-72 (m, 3H, CH Ar and NH), 7.01 (bs, 3H, CH Ar), 7.26 – 7.29 (m, 2H, CH Ar), 7.57 - 7-59 (m, 3H, CH Ar), 7.87-7.92 (m, 1H, CH Ar), 9.67-9.83 (bs, NH), 10.31 (bs, NH), 11.64 (bs, NH). **δ_C** (100 MHz, $CDCl_3$): 28.3 (3 $(CH_3)_3$),

79.8 (qC, C(CH$_3$)$_3$), 83.9 (qC, C(CH$_3$)$_3$), 84.1 (qC, C(CH$_3$)$_3$), 112.3 (CH Ar), 113.3 (CH Ar), 117.0 (CH Ar), 119.7 (CH Ar), 120.0 (CH Ar),123.9 (2 CH Ar), 124.1 (CH Ar), 124.3 (qC), 127.0 (CH Ar), 130.0 (CH Ar), 130.1 (CH Ar), 132.8 (qC), 135.2 (qC), 136.1 (qC), 138.5 (qC), 140.0 (qC), 147.9 (qC), 153.1 (qC), 153.5 (qC), 153.7 (qC), 158.1 (qC), 159.2 (qC), 163.7 (qC). **HRMS** (m/z ESI$^+$): Found: 807.2333 (M$^+$ + H. $C_{36}H_{43}BrF_3N_6O_7$ Requires: 807.2323). **ν_{max}** (ATR)/cm^{-1}: 3410 (NH), 3305 (NH), 2980, 2940, 1720 (C=O), 1589 (C=N), 1554 (C=N), 1477, 1407, 1236 (C-N), 1211 (C-O), 1144 (CF$_3$), 1112, 1094 (C-Br), 771, 832.

3-[2,3-Di(*tert*-butoxycarbonyl)guanidino]-4'-[2-(*tert*-butoxycarbonyl)-3-(2-fluoro-4-iodo phenyl)guanidino]diphenylether (26)

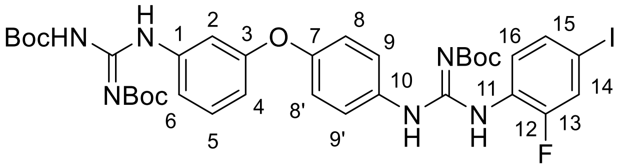

Following Method C, HgCl$_2$ (206 mg, 0.76 mmol) was added over a solution of **19** (278 mg, 0.63 mmol), thioureaderivative **14** (300 mg, 0.76 mmol) and NEt$_3$ (0.27 mL, 1.95 mmol) in CH$_2$Cl$_2$ (4 mL). After 12 h workup and silica gel chromatography (hexanes:EtOAc) afforded compound **26** as a yellow amorphous solid (299 mg, 59%). **Mp**: 87 – 88 °C. **δ_H** (400 MHz, CDCl$_3$): 1.50 (bs, 18H, (CH$_3$)$_3$), 1.53 (bs, 9H, (CH$_3$)$_3$), 6.71 – 6.75 (m, 1H, CH Ar), 6.88 – 6.90 (m, 1H, CH Ar), 7.00 – 7.07 (m, 2H, CH Ar), 7.22 – 7.24 (m, 1H, CH Ar), 7.28 – 7.32 (m, 1H, CH Ar), 7.37 – 7.46 (m, 2H, CH Ar), 7.52 – 7.65 (m, 1H, CH Ar), 7.97 – 8.01 (m, 1H, CH Ar), 8.36 – 8.40 (m, 1H, CH Ar), 9.64 (bs, 1H, NH), 9.91 (bs, 1H, NH), 10.31 (bs, 1H, NH), 11.60 (bs, 1H, NH). **δ_C** (100 MHz, CDCl$_3$): 28.2 ((CH$_3$)$_3$), 28.3 ((CH$_3$)$_3$), 79.8 (qC, C(CH$_3$)$_3$), 83.9 (qC, C(CH$_3$)$_3$), 84.1 (qC, C(CH$_3$)$_3$), 112.3, 114.4, 116.8, 120.1, 121.2, 123.0, 123.6, 124.1, 129.9, 133.8, 134.1, 138.3, 150.1, 153.4, 153.6, 158.4, 163.6. **HRMS** (*m/z* – ESI$^+$): Found: 805.2226 (M$^+$ + H. $C_{35}H_{43}N_6O_7FI$ Requires: 805.2222). **ν_{max}** (ATR)/cm^{-1}: 3411 (NH), 2976, 1719 (C=O), 1638, 1599 (C=N), 1535 (C=N), 1499, 1466, 1411, 1367, 1240 (C-O), 1153 (C-F), 1140, 1110, 1058, 816, 762 (C-I).

3-[2,3-Di(t*ert*-butoxycarbonyl)guanidino]-4'-[2-(*tert*-butoxycarbonyl)-3-(4-bromo-3-tri fluoromethylphenyl)guanidino]diphenylether (27)

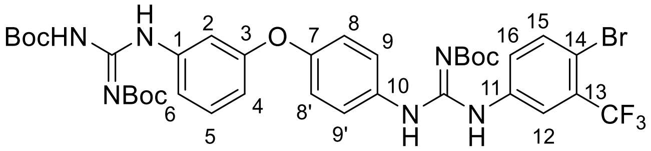

Following Method C, $HgCl_2$ (152 mg, 0.56 mmol) was added over a solution of **19** (207 mg, 0.47 mmol), thioureaderivative **16** (224 mg, 0.56 mmol) and NEt_3 (0.20 mL, 1.46 mmol) in CH_2Cl_2 (3 mL). After 12 h workup and silica gel chromatography (hexanes:EtOAc) afforded compound **27** as a yellow oil (250 mg, 66%). **δ_H** (400 MHz, $CDCl_3$): 1.49 (bs, 9H, $(CH_3)_3$), 1.53 (bs, 9H, $(CH_3)_3$), 6.71 – 6.76 (m, 1H, CH Ar), 6.88 – 6.90 (m, 1H, CH Ar), 7.00 – 7.08 (m, 2H, CH Ar), 7.22 – 7.38 (m, 3H, CH Ar), 7.57 – 7.63 (m, 2H, CH Ar), 7.88 – 7-90 (m, 1H, CH Ar), 8.00 (bs, 1H, CH Ar), 9.65 (bs, 1H NH), 9.83 (bs, 1H NH), 10.30 (bs, 1H NH), 11.60 (bs, 1H NH). **δ_C** (100 MHz, $CDCl_3$): 28.2 ($(CH_3)_3$), 28.3 ($(CH_3)_3$), 86.2 (qC, $\underline{C}(CH_3)_3$), 112.4 (CH Ar), 114.0 (CH Ar), 114.4 (CH Ar), 116.8 (CH Ar), 118.0 (CH Ar), 120.1 (CH Ar), 121.1 (CH Ar), 123.9 (CH Ar), 130.0 (CH Ar), 134.5 (qC), 135.2 (qC), 135.0 (qC), 136.5 (qC), 137.9 (qC), 138.0 (qC), 138.3 (qC), 152.7 (qC), 152.8 (qC), 153.6 (qC). **HRMS** (*m/z* – ESI$^+$): Found: 807.2329 (M^+ + H. $C_{36}H_{43}BrF_3N_6O_7$ Requires: 807.2323). **ν_{max}** (ATR)/cm^{-1}: 3260 (NH), 2980, 2929, 1718 (C=O), 1630, 1595 (C=N), 1555, 1238, 1140 (CF_3), 1105, 1055 (C-Br), 835, 773.

4'-[2,3-Di-(*tert*-butoxycarbonyl)guanidino]-3-[3-trifluoromethylphenylamino] diphenylether (46)

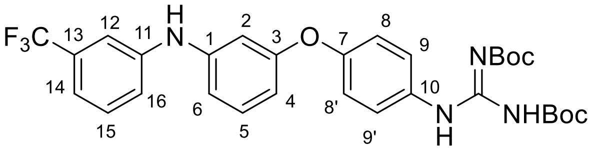

Following Method G, $HgCl_2$ (22 mg, 0.08 mmol) was added over a solution of **41** (25 mg, 0.07 mmol), *N,N'-bis*-(*tert*-butoxycarbonyl)-S-methylisothiourea) (23 mg, 0.08 mmol), and NEt_3 (0.03 mL, 0.25 mmol) in CH_2Cl_2 (1 mL). The reaction was stirred at room temperature for 24 h, then work up and silica gel chromatography (hexanes:EtOAc) afforded **46** as a yellow solid (30 mg, 73%). **Mp**: 60-63 °C. **δ_H** (400 MHz, $CDCl_3$): 1.48 (s, 9H, $(CH_3)_3$), 1.54 (s, 9H, $(CH_3)_3$), 5.99 (bs, 1H, NH), 6.58 (dd, *J* = 8.1, 2.3 Hz, 1H, H-4), 6.67 (t, *J* = 2.2 Hz, 1H, H-2), 6.80 (dd, *J* = 8.0, 1.4 Hz, 1H, H-6), 6.98 (d, *J* = 8.9 Hz, 2H, H-8 and H-8'), 7.12 (d, *J* = 7.6 Hz, 1H, H-14), 7.18 – 7.22 (m, 2H, H-5 or H-15 and H-16), 7.25 (s, 1H, H-12), 7.33 (t, *J* = 7.9 Hz, 1H, H-5 or H-15), 7.54 (d,

J = 8.9 Hz, 2H, H-9 and H-9'), 10.28 (bs, NH), 11.64 (bs, NH). **δC** (100 MHz, $CDCl_3$): 28.2 ($(CH_3)_3$), 28.3 ($(CH_3)_3$), 79.8 (qC, <u>C</u>$(CH_3)_3$), 83.9 (qC, <u>C</u>$(CH_3)_3$), 108.4 (CH Ar, C-2), 111.8 (CH Ar, C-4), 112.8 (CH Ar, C-6), 114.0 (q, J = 3.9 Hz, CH Ar, C-12), 117.4 (q, J = 3.8 Hz, CH Ar, C-14), 119.8 (2 CH Ar, C-8 and C-8'), 120.4 (CH Ar, C-16), 124.1 (2 CH Ar, C-9 and C-9'), 124.2 (d, J = 272.4 Hz, qCF_3), 129.9 (CH Ar, C-5 or C-15), 130.6 (CH Ar, C-5 or C-15), 131.9 (q, J = 32.1 Hz, qC, C-13), 132.6 (qC), 143.6 (qC), 143.7 (qC), 153.5 (qC), 153.6 (qC), 153.8 (qC), 159.0 (qC), 163.6 (qC). **HRMS** (m/z ESI^+): Found 587.2485 (M^+ + H. $C_{30}H_{34}N_4O_5F_3$ Requires: 587.2481). **ν_{max}**(ATR)/cm^{-1}:3295 (NH), 3261 (NH), 2979, 1719 (C=O), 1594 (C=N), 1407, 1325, 1304, 1231, 1145 (CF3), 1110 (C-O), 1056 (C-Cl), 1028, 998, 978, 774, 656.

4'-[2,3-Di-(*tert*-butoxycarbonyl)guanidino]-3-[3-pentafluorosulphanylphenylamino]di phenylether (47)

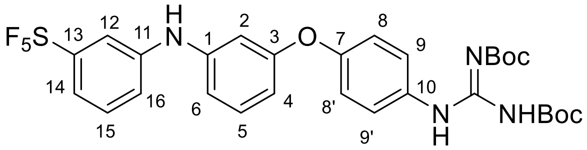

Following Method G, $HgCl_2$ (28 mg, 0.11 mmol) was added over a solution of **42** (40 mg, 0.1 mmol), *N,N'-bis*-(*tert*-butoxycarbonyl)-S-methylisothiourea (30 mg, 0.11 mmol), and NEt_3 (0.05 mL, 0.35 mmol) in CH_2Cl_2 (1 mL). The reaction was stirred at room temperature for 24 h, then work up and silica gel chromatography (hexanes:EtOAc) afforded **47** as a yellow solid (48 mg, 75%). **Mp**: 70-73 °C. **δH** (600 MHz, $CDCl_3$): 1.48 (s, 9H, $(CH_3)_3$), 1.54 (s, 9H, $(CH_3)_3$), 6.03 (bs, 1H, NH), 6.60 (dd, J = 8.1, 2.1 Hz, 1H, H-4), 6.66 (t, J = 1.9 Hz, 1H, 1H, H-2), 6.79 (dd, J = 7.9, 1.7 Hz, 1H, H-6), 6.97 (d, J = 8.8 Hz, 2H, H-8 and H-8'), 7.16 (d, J = 7.6 Hz, 1H, H-16), 7.21 (t, J = 8.1 Hz, 1H, H-5 or H-15), 7.24 – 7.26 (m, 1H, H-14), 7.30 (t, J = 8.1 Hz, H-5 or H-15), 7.39 (s, 1H, H-12), 7.54 (d, J = 8.8 Hz, 2H, H-9 and H-9'), 10.28 (bs, NH), 11.63 (bs, NH). **δC** (150 MHz, $CDCl_3$): 28.0 ($(CH_3)_3$), 28.3 ($(CH_3)_3$), 80.0 (q, <u>C</u>$(CH_3)_3$), 83.9 (q, <u>C</u>$(CH_3)_3$), 108.5 (CH Ar, C-2), 112.1 (CH Ar, C-4), 112.8 (CH Ar, C-6), 115.1 (m, CH Ar, C-12), 118.1 (m, CH Ar, C-14), 119.9 (2 CH Ar, C-8 and C-8'), 120.0 (CH Ar, C-16), 124.2 (2 CH Ar, C-9 and C-9'), 129.5 (CH Ar, C-5 or C-15), 130.7 (CH Ar, C-5 or C-15), 132.6 (qC), 143.5 (qC), 143.6 (qC), 153.5 (qC), 153.7 (qC), 153.9 (qC), 155.1 (m, qC, C-13), 159.0 (qC), 163.6 (qC). **HRMS** (m/z ESI^+): Found 645.2171 (M^+ + H. $C_{29}H_{34}F_5N_4O_5S$ Requires: 645.2165). **ν_{max}**(ATR)/cm^{-1}: 3303 (NH), 2979, 1719 (C=O), 1594 (C=N), 1486, 1486, 1407, 1303, 1293, 1213, 1146 (C-N), 1110 (C-O), 839 (SF_5), 806 (SF_5), 772 (SF_5).

4'-[2,3-Di-(t*ert*-butoxycarbonyl)guanidino]-3-[4-chloro-3-trifluoromethylphenylamino]di phenylether (45)

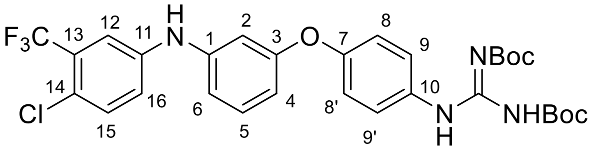

Following Method G, $HgCl_2$ (206 mg, 0.76 mmol) was added over a solution of **40** (277 mg, 0.73 mmol), *N,N'-bis*-(*tert*-butoxycarbonyl)-*S*-methylisothiourea (221 mg, 0.76 mmol) and NEt_3 (0.35 mL, 2.56 mmol) in CH_2Cl_2(4 mL). The reaction was stirred at room temperature for 24 h, then work up and silica gel chromatography (hexanes:EtOAc) afforded **54** (363 mg, 80%) as a white solid. **Mp**: 92-94 °C. **δ_H** (600 MHz, $CDCl_3$): 1.48 (s, 9H, $(CH_3)_3$), 1.54 (s, 9H, $(CH_3)_3$), 6.01 (s, 1H, NH), 6.61 (dd, *J* = 8.2, 1.5 Hz , 1H, H-4), 6.65 (s, 1H, H-2), 6.78 (dd, *J* = 8.0, 1.2 Hz, 1H, H-6), 6.98 (d, *J* = 8.8 Hz, 2H, H-8 and H-8'), 7.12 (dd, *J* = 8.6, 2.5 Hz, 1H, H-16), 7.22 (t, *J* = 8.1 Hz, 1H, H-5), 7.31-7.33 (m, 2H, H-12 and H-15), 7.54 (d, *J* = 8.7 Hz, 2H, H-9 and H-9'), 10.29 (s, 1H, NH), 11.65 (s, 1H, NH). **δ_C** (150 MHz, $CDCl_3$): 28.2 ($(CH_3)_3$), 28.3 ((($CH_3)_3$), 78.0 (qC, $\underline{C}(CH_3)_3$), 84.0 (qC, $\underline{C}(CH_3)_3$), 108.6 (CH Ar, C-2), 112.3 (CH Ar, C-4), 113.0 (CH Ar, C-6), 116.3 (d, J = 5.1 Hz, CH Ar, C-12), 120.0 (2 CH Ar, C-8 and C-8'), 120.9 (CH Ar, C-16), 122.8 (CH Ar, C-14), 122.9 (q, *J* = 273.6 Hz, qCF_3), 124.1 (2 CH Ar, C-9 and C-9'), 129.2 (q, *J* = 31.2, qC, C-13), 130.7 (CH Ar, C-5), 132.4 (CH Ar, C-15), 132.6 (qC), 142.1 (qC), 143.3 (qC), 153.5 (qC), 153.6 (qC), 153.8 (qC), 159.1 (qC), 163.4 (qC). **HRMS** (m/z ESI^+): Found: 621.2102 (M^+ + H. $C_{30}H_{33}ClF_3N_4O_5$ Requires: 621.2086). **ν_{max}** (ATR)/cm^{-1}: 3260 (NH), 2979, 2930, 1719 (C=O), 1596 (C=N), 1482, 1406, 1304, 1233 (C-O), 1213, 1141 (CF_3), 1109 (C-Cl), 1027, 818, 772.

4'-[2,3-Di-(*tert*-butoxycarbonyl)guanidino]-3-[4-chloro-phenylamino]diphenylether (44)

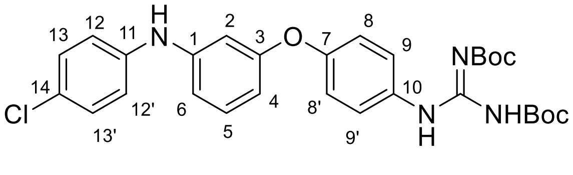

Following Method G, $HgCl_2$ (41 mg, 0.15 mmol) was added over a solution of **39** (45 mg, 0.14 mmol), *N,N'-bis*-(*tert*-butoxycarbonyl)-S-methylisothiourea (44 mg, 0.15 mmol), and NEt_3 (0.07 mL, 0.49 mmol) in CH_2Cl_2 (2 mL). The reaction was stirred at room temperature for 24 h, then work up and silica gel chromatography (hexanes:EtOAc) afforded **44** as an orange solid

(95 mg, 86 %). **Mp**: 120 °C. **δ_H** (400 MHz, $CDCl_3$): 1.49 (s, 9H, $(CH_3)_3$), 1.54 (s, 9H, $(CH_3)_3$), 5.73 (bs, 1H, NH), 6.54 (dd, *J* = 8.1, 2.3 Hz, 1H, H-4), 6.65 (t, *J* = 2.2 Hz, 1H, H-2), 6.75 (dd, *J* = 8.1, 2.1 Hz, 1H, H-6), 6.98 – 7.01 (m, 4H, H-12, H-12’, H-8 and H-8’), 7.16 – 7.22 (m, 3H, H-13, H-13’ and H-5), 7.56 (d, *J* = 9.0 Hz, 2H, H-9and H-9’), 10.29 (bs, 1H, NH), 11.64 (bs, 1H, NH). **δ_C** (100 MHz, $CDCl_3$): 28.2 ($(CH_3)_3$), 28.3 ($(CH_3)_3$), 79.8 (qC, $\underline{C}(CH_3)_3$), 83.9 (qC, $\underline{C}(CH_3)_3$), 107.6 (CH Ar, C-2), 111.1 (CH Ar, C-4), 112.0 (CH Ar, C-6), 119.6 (2 CH Ar, C-12 and C-12’ or C-8 and C-8’), 119.8 (2 CH Ar, C-12 and C-12’ or C-8 and C-8’), 123.9 (2 CH Ar, C-9 and C-9’), 126.2 (qC, C-14), 129.5 (2 CH Ar, C-13 and C-13’), 130.5 (CH Ar, C-5), 132.5 (qC), 141.3 (qC), 144.6 (qC), 153.5 (qC), 153.73 (qC), 153.74 (qC), 158.9 (qC), 163.7 (qC). **HRMS** (m/z ESI$^+$): Found 553.2190 (M^+ +H. $C_{29}H_{34}ClN_4O_5$ Requires: 553.2212). **ν_{max}** (ATR)/cm^{-1}: 3296 (NH), 2925, 1718 (C=O), 1649, 1628 (C=O), 1589 (C=N), 1485, 1390, 1367, 1330, 1310, 1258, 1148 (C-O), 1113 (C-N), 1096 (C-Cl), 829, 765.

4'-[2,3-Di-(*tert*-butoxycarbonyl)guanidino]-3-[4-chloro-3-trifluoromethylphenylamino]-4-(aminophenoxy)-5-fluorophenyl (48)

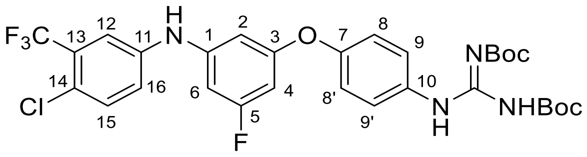

Following Method G, $HgCl_2$ (429 mg, 1.58 mmol) was added over a solution of **43** (604 mg, 1.52 mmol), *N,N’-bis*-(*tert*-butoxycarbonyl)-S-methylisothiourea (460 mg, 1.58 mmol), and NEt_3 (0.74 mL, 5.32 mmol) in CH_2Cl_2 (8 mL). The reaction was stirred at room temperature for 24 h, then work up and silica gel chromatography (hexanes:EtOAc), afforded **48** as a yellow solid (680 mg, 70 %). **Mp**: 75 - 77 °C. **δ_H** (400 MHz, $CDCl_3$): 1.47 (s, 9H, $(CH_3)_3$), 1.54 (s, 9H, $(CH_3)_3$), 6.09 (s, 1H, NH), 6.27 (dt, *J* = 9.9, 2.1 Hz, 1H, H-4), 6.36 (s, 1H, H-2), 6.44 (dt, *J* = 10.3, 2.1 Hz, 1H, H-6), 6.92 (d, *J* = 8.9 Hz, 2H, H-8 and H-8’), 7.14 (dd, *J* = 8.7, 2.7 Hz, 1H, H-16), 7.31 (d, *J* = 2.7 Hz, 1H, H-12), 7.35 (d, *J* = 8.7 Hz, 1H, H-15), 7.56 (d, *J* = 8.9 Hz, 2H, H-9 and H-9’), 10.32 (s, 1H, NH), 11.65 (s, 1H, NH). **δ_C** (100 MHz, $CDCl_3$): 28.2 ($(CH_3)_3$), 28.3 ($(CH_3)_3$), 80.0 (qC, $\underline{C}(CH_3)_3$), 84.0 (qC, $\underline{C}(CH_3)_3$), 98.9 (CH Ar, d, *J* = 25.5 Hz, C-4), 99.0 (CH Ar, d, *J* = 25.5 Hz, C-6),

102.8 (CH Ar, d, *J* = 2.7 Hz, C-2), 117.3 (q, *J* = 5.5 Hz, CH Ar, C-12), 120.4 (2 CH Ar, C-8 and C-8'), 122.0 (CH Ar, C-16), 122.8 (d, *J* = 273.6 Hz, qCF_3), 123.9 (qC, C-14), 124.4 (2 CH Ar, C-9 and C-9'), 129.3 (q, *J* = 31.2 Hz, qC, C-13), 132.5 (CH Ar, C-15), 133.2 (qC), 141.0 (qC), 144.4 (d, *J* = 12.9 Hz, qC, C-1 or C-3), 152.7 (qC), 153.5 (qC), 153.9 (qC), 160.3 (d, *J* = 13.5 Hz, qC, C-1 or C-3), 164.4 (d, *J* = 244.8 Hz, qC, C-5), 163.6 (qC). **HRMS** (m/z ESI$^+$): Found: 639.1996 (M^++ H. $C_{30}H_{32}N_4O_5ClF_4$ Requires: 639.1997). **ν_{max}** (ATR)/cm^{-1}: 3257 (NH), 2978, 1719 (C=O), 1598 (C=O), 1476, 1401, 1368, 1320 (CF_3), 1145 (C-F), 1109 (C-Cl), 808, 768.

3-{3-[2-(tert-butoxycarbonyl)-3-(3,4-difluorophenyl)guanidino]}phenoxy-[2-[(2,3-di-tert butoxycarbonyl)guanidino]pyridine (61)

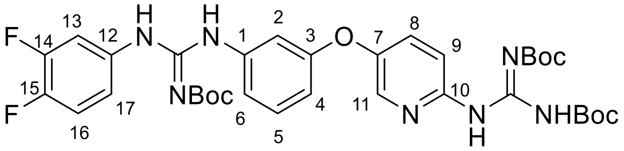

Following Method G, $HgCl_2$ (130 mg, 0.48 mmol, 1.04 eq.) was added over a solution of **56** (217 mg, 0.46 mmol), *N,N'-bis*-(*tert*-butoxycarbonyl)-*S*-methylisothiourea (139 mg, 0.48 mmol), and NEt_3 (0.22 mL, 1.61 mmol) in CH_2Cl_2 (2.5 mL). The reaction was stirred at room temperature for 48 h, then work up and silica gel chromatography (hexanes:EtOAc) afforded **61** (280 mg, 87%) as a white amorphous solid. **Mp**: 135-137 °C. **δ_H** (400 MHz, $CDCl_3$): 1.48 (s, 9H, ($(CH_3)_3$), 1.51 (9H, (s, $(CH_3)_3$), 1.53 (9H, ($(CH_3)_3$), 6.52 - 6.72 (m, 3H, CH Ar), 7.04 - 7.14 (m, 2H, CH Ar), 7.29 (bs, 1H, CH Ar), 7.40 – 7.49 (bs, 1H, CH Ar), 7.80 (bs, 1H, CH Ar), 8.11 (bs, 1H, CH Ar), 8.38 (bs, 1H, CH Ar), 9.36 (bs, NH), 9.92 - 10.14 (m, NH), 10.87 (bs, NH), 11.52 (bs, NH). **δ_C** (100 MHz, $CDCl_3$): 28.16 ($(CH_3)_3$),28.2 ($(CH_3)_3$), 28.3 ($(CH_3)_3$), 80.1 (qC, $\underline{C}(CH_3)_3$), 83.9 (qC, $\underline{C}(CH_3)_3$), 84.1 (qC, $\underline{C}(CH_3)_3$), 109.8 (CH Ar), 110.4 (CH Ar), 112.1 (CH Ar), 112.8 (CH Ar), 116.9 (CH Ar), 117.6 (CH Ar), 118.2 (CH Ar), 128.8 (CH Ar), 129.2 (CH Ar), 130.0 (CH Ar), 131.0 (CH Ar), 140.0 (CH Ar), 145 – 160, 146.3 (qC), 146.6 (qC), 149.8 (qC), 152.9 (qC), 153.0 (qC), 157.7 (qC), 158.8 (qC), 163.3 (qC). **HRMS** (*m/z* ESI$^+$): Found: 698.3110 (M^+ + H. $C_{34}H_{42}F_2N_7O_7$ Requires: 698.3114). **ν_{max}** (ATR)/cm^{-1}: 3412 (NH), 3258 (NH), 2979, 2930, 1720 (C=O), 1624 (C=N), 1596 (C=N), 1560, 1514, 1463, 1368, 1292, 1224, 1123-1104 (C-F), 771.

3-{3-[2-(tert-butoxycarbonyl)-3-(2-fluoro-4-iodophenyl)guanidino]}phenoxy-[2-[(2,3-di-tert butoxycarbonyl)guanidino]pyridine (62)

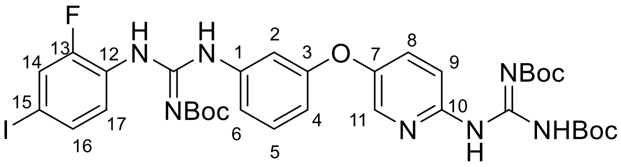

Following Method G, $HgCl_2$ (261 mg, 0.96 mmol) was added over a solution of **57** (520 mg, 0.92 mmol), *N,N'-bis*-(*tert*-butoxycarbonyl)-*S*-methylisothiourea (279 mg, 0.96 mmol), and triethylamine (0.45 mL, 3.22 mmol) in CH_2Cl_2 (5 mL). The reaction was stirred at room temperature for 48 h, then work up and silica gel chromatography (hexanes:EtOAc) afforded **82** as a white amorphous solid (500 mg, 67%). **Mp**: decomp. above 116 °C. $\boldsymbol{\delta_H}$ (400 MHz, $CDCl_3$): 1.35 (s, 9H, $(CH_3)_3$), 1.39 (s, 18H, $(CH_3)_3$), 6.39 (bs, 1H, CH Ar), 6.52 - 6.58 (m, 3H, CH Ar), 7.06 - 7.20 (m, 2H, CH Ar), 7.24 - 7.28 (m, 2H, CH Ar), 7.97 (bs, 1H, CH Ar), 8.16 (bs, NH), 8.25 (bs, 1H CH Ar), 9.00 - 9.78 (m, NH), 10.74 (bs, NH), 11.39 (bs, NH). $\boldsymbol{\delta_C}$ (100 MHz, $CDCl_3$): 28.2 ($(CH_3)_3$), 28.3 ($(CH_3)_3$), 80.0 (qC, $\underline{C}(CH_3)_3$), 83.9 (qC, $\underline{C}(CH_3)_3$), 84.1 (qC, $\underline{C}(CH_3)_3$), 112.1 (CH Ar), 112.9 (CH Ar), 116.9 (CH Ar), 117.4 (CH Ar), 123.1 (CH Ar), 128.9 (CH Ar), 130.0 (CH Ar), 131.1 (CH Ar), 133.5 (CH Ar), 139.9 (CH Ar), 145 – 160, 146.5 (qC), 148.3 (qC), 149.8 (qC), 152.8 (qC), 153.0 (qC), 157.7 (qC), 158.8 (qC), 163.3 (q). **HRMS** (m/z ESI$^+$): Found: 806.2176 (M^+ + H. $C_{34}H_{42}FIN_7O_7$ Requires: 806.2169). $\boldsymbol{\nu_{max}}$ (ATR)/cm^{-1}: 3257 (NH), 2979, 2930, 1721 (C=O), 1626 (C=N), 1591 (C=N), 1465, 1368, 1223 (C-O), 1146 – 1104 (C-F), 1056, 852, 770 (C-I).

3-{3-[2-(tert-butoxycarbonyl)-3-(4-bromophenyl)guanidino]}phenoxy-[2-[(2,3-di-tert butoxycarbonyl)guanidino]pyridine (63)

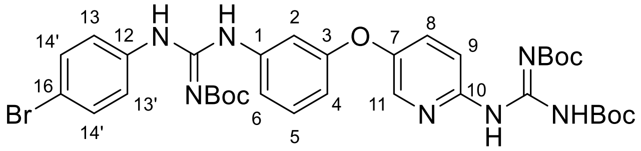

Following Method G, $HgCl_2$ (185 mg, 0.68 mmol) was added over a solution of **58** (327 mg, 0.66 mmol), *N,N'-bis*-(*tert*-butoxycarbonyl)-*S*-methylisothiourea (197 mg, 0.68 mmol), and triethylamine (0.32 mL, 2.31 mmol) in CH_2Cl_2 (4 mL). The reaction was stirred at room temperature for 48 h, then work up and silica gel chromatography (hexanes:EtOAc) afforded **63** (244, 50%) as a white amorphous solid. **Mp**: 104-107 °C. $\boldsymbol{\delta_H}$ (600 MHz, $CDCl_3$): 1.48 (s, 9H, ($(CH_3)_3$), 1.52 (s, 9H, ($(CH_3)_3$), 1.54 (9H, ($(CH_3)_3$), 6.53 – 6.78 (m, 4H, CH Ar and NH), 7.34 – 7.42 (m, 4H, CH Ar), 7.51 – 7.55 (m, 1H, CH Ar), 8.12 (bs, 1H, CH Ar), 8.39 (bs, 1H, CH Ar), 9.62 (bs, NH), 10.87 (bs, NH), 11.51 (bs, NH). $\boldsymbol{\delta_C}$ (100 MHz, $CDCl_3$): 28.0 ($(CH_3)_3$), 28.1 ($(CH_3)_3$), 28.2

((CH_3)$_3$), 79.9 (qC, C(CH_3)$_3$), 83.6 (qC, C(CH_3)$_3$), 83.9 (qC, C(CH_3)$_3$), 112.4 (CH Ar), 116.8 (CH Ar), 117.5 (CH Ar), 121.5 (CH Ar), 124.3 (CH Ar), 129.0 (CH Ar), 129.8 (CH Ar), 130.8 (CH Ar), 131.6 (CH Ar), 132.6 (CH Ar), 139.8 (CH Ar), 140 – 152, 152.8 (qC), 163.2 (qC). **HRMS** (*m/z* ESI$^+$): Found: 740.2402 (M^+ + H. $C_{34}H_{43}BrN_7O_7$ Requires: 740.2407). **ν_{max}** (ATR)/cm^{-1}: 3253 (NH), 2979, 2932, 1719 (C=O), 1625, 1586 (C=N), 1551 (C=N), 1387, 1323, 1223 (C-N), 1145 (C-O), 1123 (C-Br), 1103, 806 – 687.

3-{3-[2-(tert-butoxycarbonyl)-3-(4-bromo-3trifluoromethylphenyl)guanidino]}phenoxy-[2-[(2,3-di-tert butoxycarbonyl)guanidino]pyridine (64)

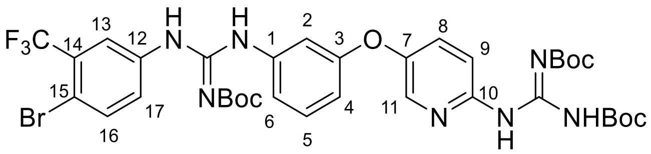

Following Method G, $HgCl_2$ (255 mg, 0.94 mmol) was added over a solution of **59** (507 mg, 0.90 mmol), *N,N'-bis*-(*tert*-butoxycarbonyl)-*S*-methylisothiourea (273 mg, 0.94 mmol), and NEt_3 (0.44 mL, 3.15 mmol) in CH_2Cl_2 (5 mL). The reaction was stirred at room temperature for 48 h, then work up and silica gel chromatography (hexanes:EtOAc) afforded **64** as a white amorphous solid (324 mg, 45%). **Mp**: 142-144 °C. **δ_H** (400 MHz, $CDCl_3$): 1.50 (s, 9H, (CH_3)$_3$), 1.53 (s, 18H, (CH_3)$_3$), 6.52 (bs, 1H, CH Ar), 6.66 – 6.69 (bs, 2H, CH Ar), 7.22 (bs, 1H, CH Ar), 7.37 (bs, 2H, CH Ar), 7.58 – 7.60 (bs, 1H, CH Ar), 7.85 – 7.92 (bs, 1H, CH Ar), 8.10 (bs, 1H, CH Ar), 8.39 (bs, 1H, CH Ar), 9.82 (bs, NH), 10.88 (bs, NH), 11.52 (bs, NH). **δ_C** (100 MHz, $CDCl_3$): 28.19 ((CH_3)$_3$), 28.21 ((CH_3)$_3$), 80.1 (qC, C(CH_3)$_3$), 84.1 (qC, C(CH_3)$_3$), 84.2 (q, C(CH_3)$_3$), 112.0 (CH Ar), 112.9 (CH Ar), 112.94 (CH Ar), 117.0 (CH Ar), 119.3 (CH Ar), 128.9 (CH Ar), 129.0 (CH Ar), 131.2 (CH Ar), 135.2 (CH Ar), 140.0 (CH Ar), 146.6 (qC), 148.1 (qC), 150.1 (qC), 152.9 (qC), 153.1 (qC), 163.3 (qC). **HRMS** (m/z ESI$^+$): Found: 808.2288 (M^+ + H. $C_{35}H_{42}BrF_3N_7O_7$ Requires: 808.2276). **ν_{max}** (ATR)/cm^{-1}: 3410 (NH), 3251 (NH), 2985 (CH), 2921, 1720 (C=O), 1645, 1631, 1583 (C=N), 1561 (C=N), 1386, 1326, 1233 (C-N), 1144 (C-O), 1128 (CF_3), 1108 (C-Br), 1081, 1059, 1020, 842, 772, 753, 550.

3-{3-[2-(tert-butoxycarbonyl)-3-(4-chloro-3trifluoromethylphenyl)guanidino]}phenoxy-[2-[(2,3-di-tert butoxycarbonyl)guanidino]pyridine (65)

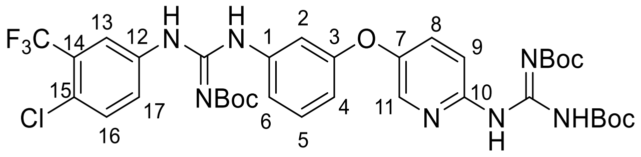

Following Method G, $HgCl_2$ (214 mg, 0.79 mmol) was added over a solution of **60** (398 mg, 0.76 mmol), *N,N'-bis*-(*tert*-butoxycarbonyl)-*S*-methylisothiourea (230 mg, 0.79 mmol), and NEt_3 (0.37 mL, 2.66 mmol) in CH_2Cl_2 (4 mL). The reaction was stirred at room temperature for 48 h, then work up and silica gel chromatography (hexanes:EtOAc) afforded **85** (354 mg, 61%) as a white-yellow amorphous solid. **Mp**: 84 - 86 °C. **δ_H** (400 MHz, $CDCl_3$): 1.49 (s, 9H, $(CH_3)_3$), 1.51 (s, 9H, $(CH_3)_3$), 1.53 (s, 9H, $(CH_3)_3$), 6.51 – 6.71 (m, 2H, CH Ar), 6.99 (bs, 1H, CH Ar), 7.23 – 7.30 (m, 1H, CH Ar), 7.40 – 7.63 (m, 3H, CH Ar), 7.83 – 7.92 (m, 1H, CH Ar), 8.11 (bs, 1H, CH Ar), 8.38 – 8.60 (m, 1H, CH Ar), 9.72 (bs, 1H, NH), 9.81 (bs, 1H, NH), 10.87 (bs, 1H, NH), 11.52 (bs, 1H, NH). **δ_C** (100 MHz, $CDCl_3$): 28.1 ($(CH_3)_3$), 28.2 ($(CH_3)_3$), 80.0 (qC, $\underline{C}(CH_3)_3$), 84.0 (qC, $\underline{C}(CH_3)_3$), 112.9, 116.9, 119.5, 121.4, 124.1, 129.1, 130.0, 131.0, 132.5, 139.9, 146.7, 148.0, 149.7, 150.1, 152.8, 152.9, 157.7, 158.8, 163.2. **HRMS** (m/z ESI$^+$): Found: 764.2784 (M^+ + H. $C_{35}H_{42}ClF_3N_7O_7$ Requires: 764.2786). **ν_{max}** (ATR)/cm^{-1}: 3256 (NH), 2925, 2855, 1720 (C=O), 1628 (C=N), 1592 (C=N), 1557, 1465, 1412, 1387, 1229 (C-N), 1143 (CF_3), 1125, (C-O), 1105 (C-Cl), 1057, 1105, 848, 804, 772, 688.

4'-[2,3-Di-(*tert*-butoxycarbonyl)guanidino]-3-[4-chloro-3-trifluoromethylphenylamino] phenoxypyridine (69)

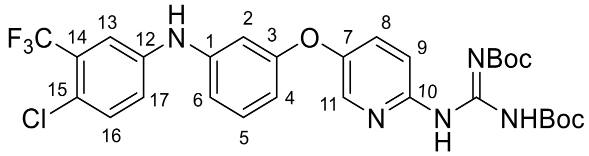

Following Method G, $HgCl_2$ (337 mg, 1.24 mmol) was added over a solution of **68** (451 mg, 1.19 mmol), *N,N'-bis*-(*tert*-butoxycarbonyl)-*S*-methylisothiourea (359 mg, 1.24 mmol) and NEt_3 (0.69 mL, 4.17 mmol) in CH_2Cl_2 (6 mL). The reaction was stired at room temperature for 24 h, then work up and silica gel chromatography (hexanes:EtOAc) afforded **69** (525 mg, 71%) as a white solid. **Mp:** 68-70 °C. **δ_H** (400 MHz, $CDCl_3$): 1.52 (s, 9H, $(CH_3)_3$), 1.54 (s, 9H, $(CH_3)_3$), 5.88 (s, 1H, NH), 6.59 (dd, *J* = 8.4, 2.1 Hz, 1H, H-4), 6.66 (t, *J* = 2.1 Hz, 1H, H-2), 6.80 (dd, *J* = 7.9, 1.8 Hz, H-6), 7.14 (dd, *J* = 8.7, 2.7 Hz, 1H, H-17), 7.24 (t, *J* = 8.1 Hz, 1H, H-5), 7.32 – 7.36 (m, 2H, H-13 and H-16), 7.40 (dd, *J* = 9.0, 2.8 Hz, 1H, H-8), 8.11 (d, *J* = 2.7 Hz, 1H, H-11), 8.39

(d, J = 8.6 Hz, 1H, H-9), 10.88 (s, 1H, NH), 11.52 (s, 1H, NH). **δ_C** (100 MHz, $CDCl_3$): 28.2 ($(CH_3)_3$), 28.3 ($(CH_3)_3$), 80.1 (qC, $\underline{C}(CH_3)_3$), 84.2 (qC, $\underline{C}(CH_3)_3$), 108.1 (CH Ar, C-2), 111.7 (CH Ar, C-4), 113.3 (CH Ar, C-6), 116.6 (q, J = 5.7 Hz, CH Ar, C-13), 117.1 (CH Ar, C-9), 121.2 (CH Ar, C-17), 122.8 (d, J = 273.4 Hz, qCF_3), 123.3 (qC, C-15), 129.1 (CH Ar, C-8), 129.3 (d, J = 27.0 Hz, qC, C-14), 131.0 (CH Ar, C-5), 132.5 (CH Ar, C-16), 140.0 (CH Ar, C-11), 141.8 (qC), 143.5 (qC), 146.6 (qC), 143.8 (qC), 152.3 (qC), 153.1 (qC), 158.8 (qC), 163.3 (qC). **HRMS** (m/z ESI$^+$): Found: 622.2054 (M^+ + H. $C_{29}H_{32}N_5O_5ClF_3$ Requires: 622.2044). **ν_{max}**(ATR)/cm^{-1}:3253 (N-H), 2980 (C-H), 1721 (C=O), 1597 (C=N), 1561, 1477, 1386, 1368, 1290 (C-O), 1122 (CF_3), 1140 (C-N), 1110, 1056 (C-Cl), 1027, 998, 978, 764, 664.

1.3.7. Isourea derivative precursors

2-(4-Iodophenoxy)tetrahydro-2*H*-pyrane[8]

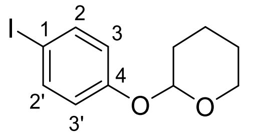

To a solution of 4-iodophenol (1.10 g, 5 mmol, 1 eq.) and pyridinium p-toluenesulfonate (PPTS) (126 mg, 0.5 mmol, 10 mol%) in dry CH_2Cl_2 (25 mL), 3,4- dihydro-2H-pyran (0.7 mL, 7.5 mmol, 1.5 eq.) was added. The resultant mixture was stirred for 2.5 h at room temperature. The reaction mixture was diluted with diethyl ether and washed once with saturated brine. The organic layer was dried over anhydrous Na_2SO_4, filtered, and concentrated under reduced pressure. Purification by flash column chromatography over silica gel (hexane-EtOAc) gave the product as a white solid (1.2 g, 82%). **Mp**: 63-64°C (lit. 64-65 °C).[8] **δ_H** (400 MHz, $CDCl_3$): 1.56 – 1.73 (m, 3H, CH_2), 1.82 – 1.86 (m, 2H, CH_2), 1.92 – 2.03 (m, 1H, CH_2), 3.55 – 3.61 (m, 1H, CH_2), 3.82 – 3.88 (m, 1H, CH_2), 5.36 (t, J = 3.2 Hz, 1H, CH), 6.82 (dd, J = 8.4, 3.7 Hz, 2H, H-3 and H-3'), 7.54 (dd, J = 8.4, 3.7 Hz, 2H, H-2 and H-2').

3-{4-[(Tetrahydro-2*H*-pyran-2-yl)oxy]phenoxy}aniline (71)[9]

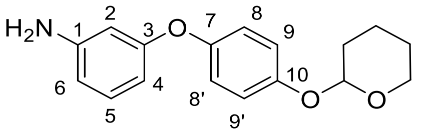

An oven-dried screw cap test tube was charged with a magnetic stirbar, copper(I) iodide (20.8 mg, 0.11 mmol, 5 mol%), 2-picolinic acid (26.8 mg, 0.22 mmol, 10 mol%), 2-(4-Iodophenoxy)tetrahydro-2*H*-pyrane (661.8 mg, 2.18 mmol), 3-aminophenol (285.9 mg, 2.62 mmol) and K_3PO_4 (926 mg, 4.36 mmol). The tube was then evacuated and back-filled with argon (three times). Under a counterflow of argon, DMSO (4.0 mL) was added by syringe. The tube was placed in a preheated oil bath at 80 ℃ and the reaction mixture was stirred vigorously for 24 h. The reaction mixture was cooled to room temperature and diluted with EtOAc and H_2O. The organic layer was separated and the aqueous layer was extracted twice more with EtOAc. Combined organic layers were dried over Na_2SO_4 and the filtrate was concentrated and the resulting residue was purified via silica gel chromatography (hexane-EtOAc) to give **71** (534 mg, 86%) as a yellow liquid. **δ_H** (400 MHz, $CDCl_3$): 1.58 – 1.73 (m, 3H, CH_2), 1.84 – 1.88 (m, 2H, CH_2), 1.96 – 2.04 (m, 1H, CH_2), 3.59 – 3.64 (m, 1H, CH_2),3.90 – 3.98 (m, 1H, CH_2), 5.35 (t, *J* = 3.4 Hz, 1H, CH), 6.33 (t, *J* = 2.2 Hz, 1H, H-2), 6.38 (dd, *J* = 8.2, 2.3 Hz, 1H, H-4), 6.42 (dd, *J* = 7.3, 2.1 Hz, 1H, H-6), 6.96 (d, *J* = 9.2 Hz, 2H, H-8 and H-8’ or H-9 and H-9’), 7.03 (d, *J* = 9.1 Hz, 2H, H-9 and H-9’ or H-8 and H-8’), 7.07 (t, *J* = 8.1 Hz, 1H, H-5). **δ_C** (150 MHz, $CDCl_3$): 19.0 (CH_2), 25.4 (CH_2), 30.6 (CH_2), 62.3 (CH_2), 97.4 (CH), 105.1 (CH Ar, C-2), 108.6 (CH Ar, C-4), 110.0 (CH Ar, C-6), 117.8 (2 CH Ar, C-8 and C-8’ or C-9 and C-9’), 121.0 (2 CH Ar, C-8 and C-8’ or C-9 and C-9’), 130.4 (CH Ar, C-5), 147.0 (qC), 150.9 (qC), 153.4 (qC), 159.7 (qC). **HRMS** (m/z ESI^+): Found 286.1454 (M^+ + H. $C_{17}H_{20}NO_3$, Requires: 286.1443). **ν_{max}** (ATR)/cm^{-1}: 3331 (NH), 2944 (CH), 2872 (CH), 1654, 1601, 1499, 1486, 1242, 1197 (C-O), 1180, 1034, 961, 835, 776, 660, 685.

4-chloro-*N*-(3-(4-((tetrahydro-2*H*-pyran-2-yl)oxy)phenoxy)phenyl)-3-(trifluoromethyl) aniline (72)

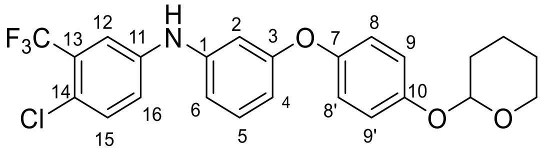

Following Method D, $Pd_2(dba)_3$ (3 mol%, 37 mg), Xantphos (5 mol%, 39 mg), Cs_2CO_3 (615.8 mg, 1.89 mmol), and remaining liquid compound **71** (385 mg, 1.35 mmol) and 4-bromo-1-chloro-2-(trifluoromethyl)benzene (0.20 mL, 1.35 mmol) were mixed, followed syringe addition of dry toluene (2.8 mL). The mixture was stirred at 90 °C for 24 h and usual work up and flash chromatography afforded **72** as a yellow oil (532 mg, 85%). **δ_H** (400 MHz, $CDCl_3$): 1.51 – 1.88 (m, 4H, CH_2), 1.96 – 2.05 (m, 2H, CH_2), 3.50 – 3.64 (m, 1H, CH_2), 3.86 – 4.04 (m, 1H, CH_2), 5.36 (t, *J* = 3.3 Hz, 1H, CH), 5.83 (s, 1H, NH), 6.59 (dd, J = 8.1, 1.8 Hz, 1H, H-4), 6.62 (t, J = 2.1 Hz, 1H, H-2), 6.72 (dd, J = 7.9, 1.8 Hz, 1H, H-6), 6.82 (d, *J* = 8.9, 2H, H-8 and H-8' or H-9 and H-9'), 6.94 (d, *J* = 8.9 Hz, 2H, H-8 and H-8' or H-9 and H-9'), 7.09 (dd, *J* = 8.7, 2.6 Hz, 1H, H-16), 7.21 (t, *J* = 8.1, H-5), 7.30 – 7.33 (m, 2H, H-12 and H-15). **δ_C** (100 MHz,$CDCl_3$): 25.3 (CH_2), 30.6 (CH_2), 62.2 (CH_2), 62.4 (CH_2), 97.2 (CH), 107.6 (CH Ar, C-2), 111.5 (CH Ar, C-4), 112.6 (CH Ar, C-6), 116.0 (q, *J* = 5.3 Hz, qC, C-12), 116.5 (2 CH Ar, C-8 and C-8' or C-9 and C-9'), 121.0 (CH Ar, C-16), 121.5 (2 CH Ar, C-8 and C-8' or C-9 and C-9'), 122.9 (d, *J* = 273.3 Hz, qCF_3), 129.1 (d, *J* = 31.2 Hz, qC, C-13), 130.7 (CH Ar, C-5), 132.4 (CH Ar, C-15), 142.1 (qC), 143.0 (qC), 149.6 (qC), 152.4 (qC), 160.1 (qC). **HRMS** (m/z ESI^-): Found 462.1085 (M^- - H. $C_{24}H_{20}NO_3ClF_3$ Requires: 462.1084). **ν_{max}** (ATR)/cm^{-1}: 3333 (NH), 3059, 3028, 2928, 1649, 1617, 1593, 1484, 1448, 1336 (C-N), 1177 (C-O), 1133 (CF_3), 1108 (C-Cl), 1133, 977, 764, 694.

4-(3-((4-chloro-3-(trifluoromethyl)phenyl)amino)phenoxy)phenol (73)

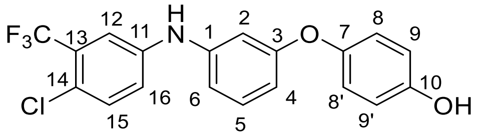

A mixture of **72** (684 mg, 1.47 mmol), methanol (12mL) and montmorillonite KSF (700 mg) was stirred at 50 °C for 4 h. After completion of the reaction, the catalyst was removed by filtration and washed with EtOAc. Evaporation of the solvent gave the corresponding crude product that was further purified by silica gel chromatography (hexane:EtOAc) to afford the title product as ayellow liquid (558 mg, 99%). **δ_H** (400 MHz, $CDCl_3$): 6.59 (dd, *J* = 7.9, 2.0 Hz, 1H, H-4), 6.62 (app. t, *J* = 2.2 Hz, 1H, H-2), 6.72 (dd, *J* = 8.0, 1.5 Hz, 1H, H-6), 6.83 (d, *J* = 8.9 Hz, 2H, H-9 and H-9'), 6.95 (d, *J* = 8.9 Hz, 2H, H-8 and H-8'), 7.09 (dd, *J* = 8.7, 2.7 Hz, 1H, H-16), 7.21 (t, *J* = 8.1 Hz, 1H, H-5), 7.31 – 7.33 (m, 2H, H-12 and H-15). **δ_C** (100 MHz, $CDCl_3$): 107.6 (CH Ar, C-2), 111.5 (CH Ar, C-4), 112.6 (CH Ar, C-6), 116.1 (q, *J* = 5.5 Hz, CH Ar, C-12), 116.5 (2

CH Ar, C-9 and C-9'), 120.9 (CH Ar, C-16), 121.5 (2 CH Ar, C-8 and C-8'), 122.8 (qC, C-14), 122.9 (d, J = 273.5 Hz, qCF_3), 129.1 (d, *J* = 31.2 Hz, qC, C-13), 130.7 (CH Ar, C-5), 132.4 (CH Ar, C-15), 140.1 (qC), 143.0 (qC), 149.6 (qC), 152.4 (qC), 160.1 (qC). **HRMS** (m/z ESI^+): Found 380.0661 (M^+ + H. $C_{19}H_{14}NO_2ClF_3$ Requires: 380.0665). ν_{max} (ATR)/cm^{-1}: 3368 (OH), 2984 (C-H), 1704, 1596, 1503, 1484, 1442, 1331 (C-N), 1258, 1232, 1205 (C-O), 1138 (CF_3), 1114 (C-Cl), 1043, 1029, 837, 780, 683.

4'-[2,3-Di-(*tert*-butoxycarbonyl)isourea]-3-[4-chloro-3-trifluoromethylphenylamino] diphenylether (74)

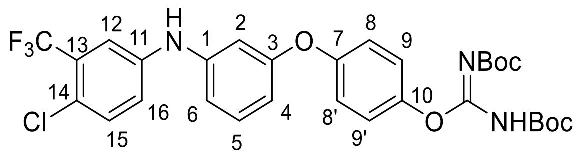

Following a modification of Method G, $HgCl_2$ (251 mg, 0.93 mmol) was added over a solution of **73** (338 mg, 0.89 mmol), *N*,*N'*-*bis*-(*tert*-butoxycarbonyl)-S-methylisothiourea (269 mg, 0.93 mmol), and NEt_3 (0.43 mL, 3.11 mmol) in CH_2Cl_2 (5 mL). The reaction was stirred at room temperature for 24 h, then work up and silica gel chromatography (hexanes:EtOAc) afforded **74** as a yellow oil (364 mg, 65%). δ_H (400 MHz, $CDCl_3$): 1.43 (s, 9H, $(CH_3)_3$), 1.54 (s, 9H, $(CH_3)_3$), 5.92 (bs, 1H, NH), 6.62 (dd, *J* = 8.4, 2.0 Hz, 1H, H-4), 6.67 (t, *J* = 2.2 Hz, 1H, H-2), 6.78 (dd, *J* = 7.9, 1.8 Hz, 1H, H-6), 6.99 (d, *J* = 9.0 Hz, 1H, H-8 and H-8' or H-9 and H-9'), 7.11-7.16 (m, 3H, H-8 and H-8' or H-9 and H-9'and H-16), 7.23 (t, *J* = 8.1 Hz, 1H, H-5), 7.30 (d, *J* = 2.8 Hz, 1H, H-12), 7.33 (d, *J* = 8.7 Hz, 1H, H-15), 10.66 (bs, 1H, NH). δ_C (100 MHz, $CDCl_3$): 28.1 ($(CH_3)_3$), 28.2 ($(CH_3)_3$), 81.4 (qC, $\underline{C}(CH_3)_3$), 83.2 (qC, $\underline{C}(CH_3)_3$), 108.9 (CH Ar, C-2), 112.4 (CH Ar, C-4), 113.3 (CH Ar, C-6), 116.3 (q, *J* = 5.6 Hz, CH Ar, C-12), 120.0 (2 CH Ar, C-8 and C-8' or C-9 and C-9'), 120.8 (qC, C-14), 120.9 (CH Ar, C-16), 122.9 (d, *J* = 273.5 Hz, qCF_3), 123.1 (2 CH Ar, C-8 and C-8' or C-9 and C-9'), 129.2 (d, *J* = 31.2 Hz, qC, C-13), 130.8 (CH Ar, C-5), 132.4 (CH Ar, C-15), 142.0 (qC), 143.3 (qC), 146.9 (qC), 148.4 (qC), 154.5 (qC), 158.68 (qC), 158.74 (qC), 162.1 (qC). **HRMS** (m/z ESI^+): Found 644.1747 (M^+ + Na. $C_{30}H_{31}N_3O_6ClF_3$ Requires: 644.1751). ν_{max} (ATR)/cm^{-1}: 3359(NH), 2934, 1739 (C=O), 1770 (C=O), 1739 (C=O), 1625 (C=N), 1593 (C=N), 1483 (C-N), 1131 (CF_3), 1114, (C-Cl), 1090 (C-O), 876, 853, 818, 768.

2. Computational details

All ligands were fully optimized at DFT level (M06-2X functional) with the 6-311+G* basis set using the Gaussian16 program.[10] Frequency calculations were performed at the same computational level to confirm that the resulting optimized structures were energetic minima. The effect of water solvation was accounted using the SCRF-PCM approach implemented in the Gaussian16 package including dispersing, repulsing and cavitation energy terms of the solvent in the optimization.

In order to perform molecular docking studies, the model of an active ATP-containing phosphorylated BRAF, previously developed by us,[11] was modified eliminating the Adenosine nucleoside for the sake of simplification. All molecular docking experiments were performed using identically parameterized receptor structures and Autodock Vina[12] using VMD[13] as visualisation software. A relaxed protein model (protein limited/truncated by the docking box) but with the Met620 refined (manually using directly MOPAC) to display the sulfur atom towards the ligand pocket (in order to maximize vdW interactions) was used.

Individual runs were performed for each ligand, ranking the resulting docked poses using the AutoDock Vina built-in scoring function. In all cases, the optimised box size was set at 15, 15, and 15 for x, y and z, respectively, and the grid centre was set to 0.003, 10.398 and 3.027 for x, y and z, respectively.

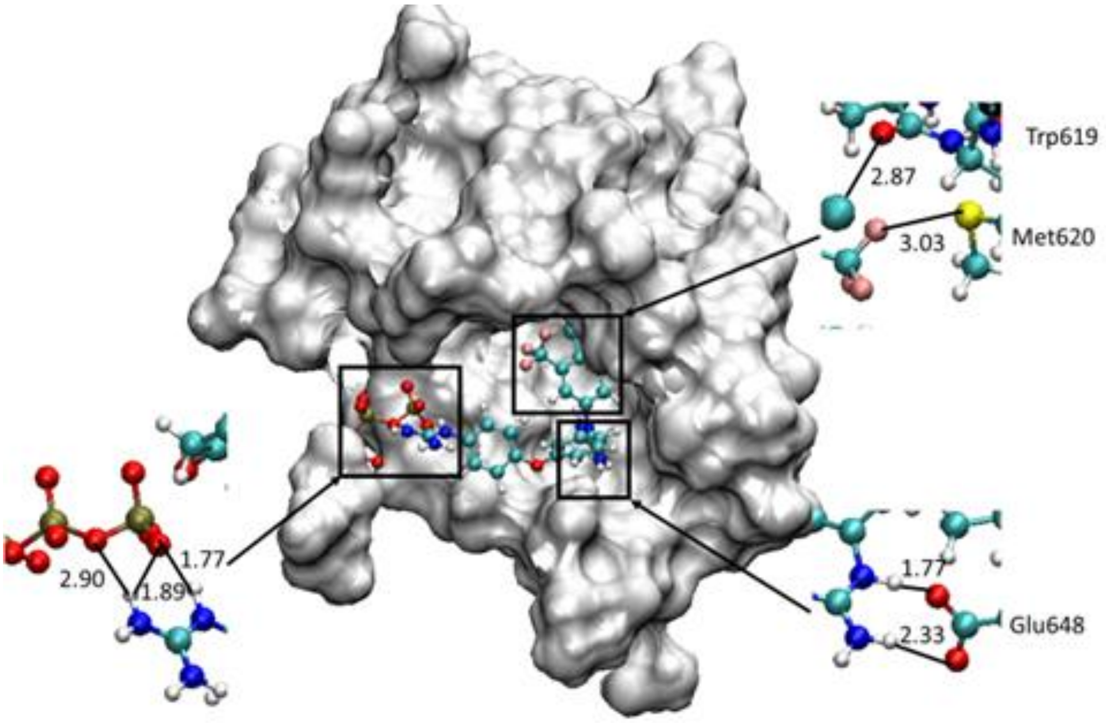

Figure S1. Docking of lead compound **1** in the in-house TP-containing BRAF model indicating the bifurcated (bottom left) and parallel (bottom right) HB interactions observed as well as some contacts within the hydrophobic pocket (up right). Distances are expressed in Å.

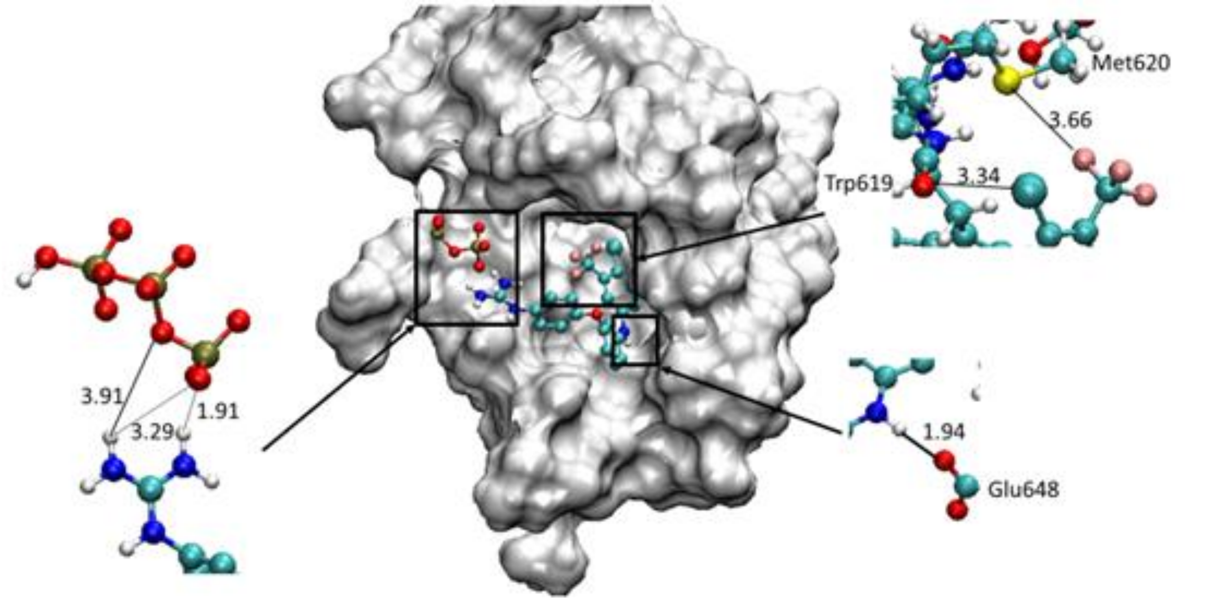

Figure S2. Docking of derivative **3** in the in-house TP-containing BRAF model indicating the bifurcated (bottom left) and single (bottom right) HB interactions observed as well as some contacts within the hydrophobic pocket (up right). Distances are expressed in Å.

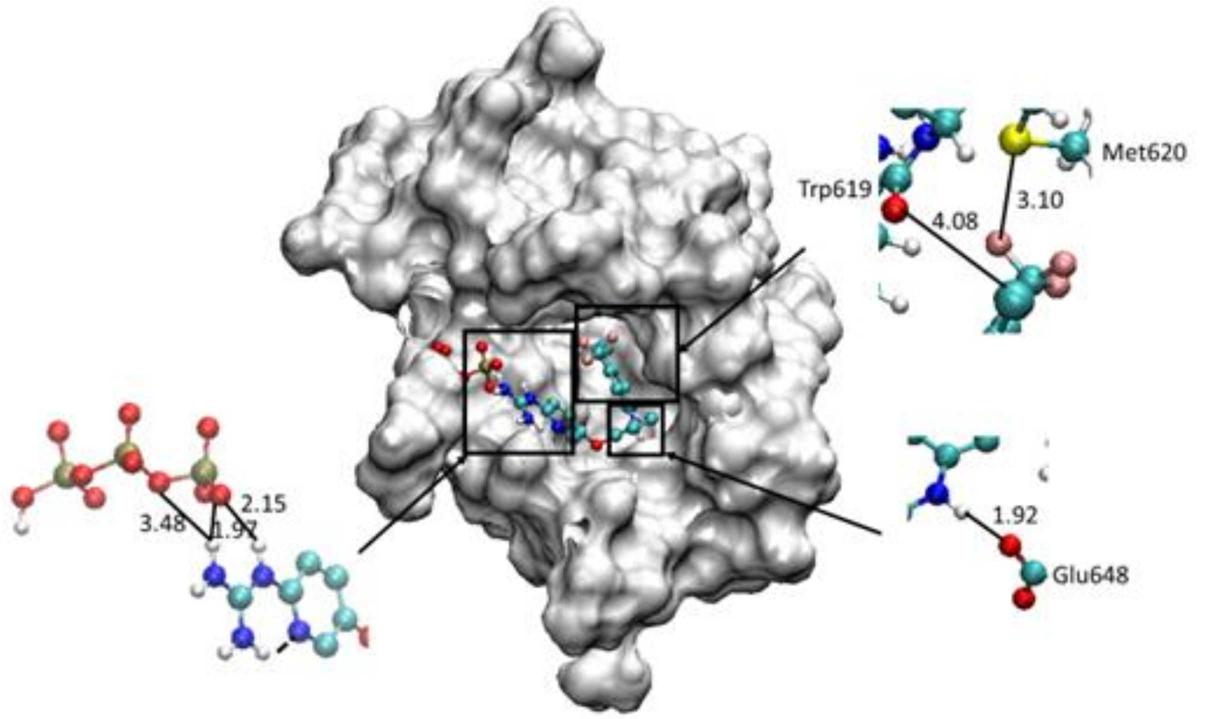

Figure S3. Docking of derivative **4** in the in-house TP-containing BRAF model indicating the bifurcated (bottom left), single (bottom right) HB interactions observed as well as some contacts within the hydrophobic pocket (up right). Distances are expressed in Å.

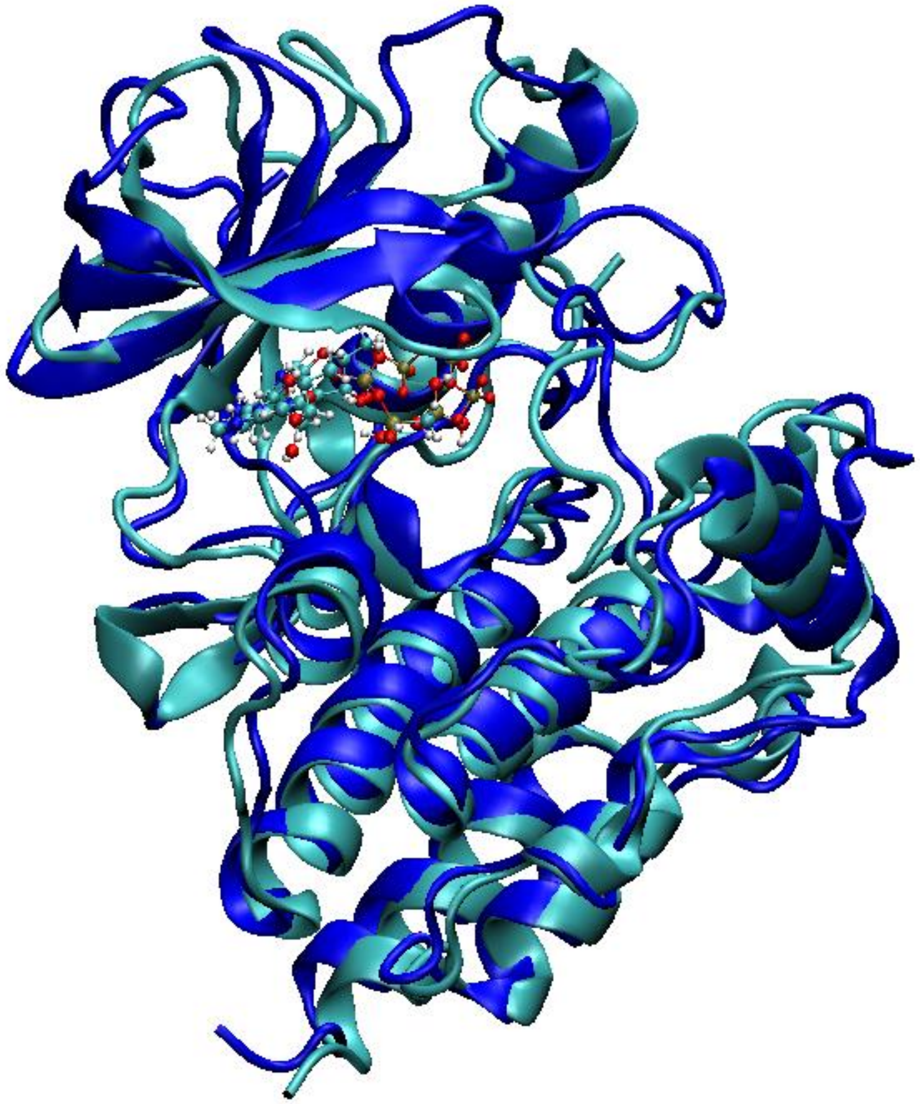

Figure S4. Alignment of the crystal structure of BRAF bound to AMP PCP (PDB 6U2G, ref. 10) in cyan and our in-house BRAF model bound to ATP (ref. 11) in blue (RMSD = 2.53 Å) using multiSeq extension in VMD.

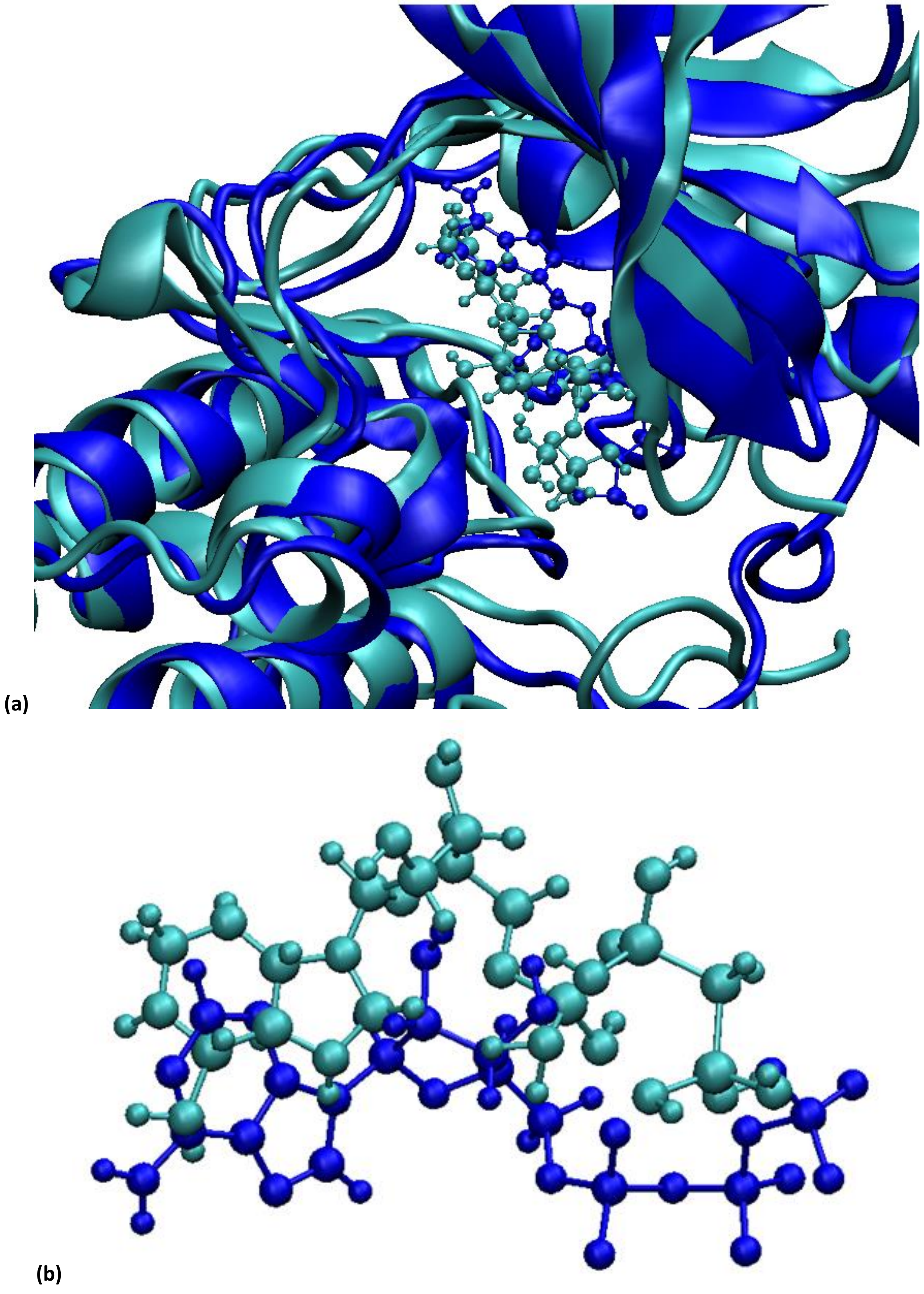

Figure S5. **(a)** Close-up of the ATP occupying pocket of the aligned BRAF structures (crystal structure in cyan; in-house model in blue) and **(b)** orientation of the two ATP-like molecules as bound into the ATP binding site (AMP-PCP, cyan; ATP, blue).

3. Theoretical physicochemical and pharmacokinetic parameters

Table S1. Calculated physicochemical properties for all final hydrochloride salts synthesised. All properties were calculated with SwissADME,[14] except for the pK_{aH} values which were calculated using ChemAxon's Marvin.[15]

Code	MW (g/mol)	pK_{aH}	#Heavy atoms	Fraction Csp^3	#Rotatable bonds	#HBA	#HBD
**Sorafenib**	464.82	3.03/11.55	32	0.1	9	7	3
**1**	464.87	8.71/ 7.02	32	0.05	9	4	6
**5**	380.42	8.72/7.40	28	0	8	2	6
**6**	398.41	8.70/6.93	29	0	8	3	6
**7**	506.32	8.56/6.29	29	0	8	2	6
**8**	441.32	8.72/7.36	28	0	8	1	6
**9**	509.32	8.71/7.10	32	0.05	9	4	6
**10**	506.32	6.47/8.40	29	0	8	2	6
**11**	509.32	7.27/8.55	32	0.05	9	4	6
**28**	353.83	8.86	25	0	6	1	4
**3**	421.82	8.86	29	0.05	7	4	4
**29**	387.38	9	28	0.05	7	4	4
**30**	445.43	8.17	30	0	7	6	4
**36**	439.81	8.62	30	0.05	7	5	4
**49**	399.4	7.56/6.79	29	0	8	4	6
**50**	507.3	7.35/6.22	29	0	8	3	6
**51**	442.31	7.71/7.09	28	0	8	2	6
**52**	510.31	7.61/6.92	32	0.05	9	5	6
**2**	465.86	7.59/6.86	32	0.05	9	5	6
**4**	422.81	7.62	29	0.05	7	5	4
**70**	422.81	8.32	29	0.05	7	5	3
**75**	457.85	10.66	30	0.05	7	7	3

Table S2. Calculated physicochemical properties for all synthesised final hydrochloride salts. All properties were calculated with SwissADME.[1]

Code	TPSA (Å²)	XLOGP3	Consensus Log P (average of all methods)	Ali Log S	Ali Sol. (mg/mL)	Ali Sol. (µmol/L)	Ali Class
**Sorafenib**	92.35	4.07	4.11	-5.71	8.98E-04	1.93	Mod. sol.
**1**	122.52	4.31	3.31	-6.6	1.18E-04	2.53	Poorly sol.
**5**	122.52	2.9	2.13	-5.13	2.80E-03	7.35	Mod. sol.
**6**	122.52	3	2.37	-5.24	2.31E-03	5.79	Mod. sol.
**7**	122.52	3.55	2.77	-5.81	7.88E-04	1.56	Mod. sol.
**8**	122.52	3.49	2.43	-5.75	7.93E-04	1.80	Mod. sol.
**9**	122.52	4.37	3.38	-6.66	1.12E-04	2.19	Poorly sol.
**10**	122.52	3.55	2.83	-5.81	7.88E-04	1.56	Mod. sol.
**11**	122.52	4.37	3.39	-6.66	1.12E-04	2.19	Poorly sol.
**28**	84.9	4.31	3.35	-5.81	5.52E-04	1.56	Mod. sol.
**3**	84.9	5.2	4.44	-6.73	7.85E-05	1.86	Poorly sol.
**29**	84.9	4.57	3.84	-6.08	3.25E-04	8.39	Poorly sol.
**30**	110.2	6.96	4.14	-9.09	3.64E-07	8.17	Poorly sol.
**36**	84.9	5.3	4.56	-6.83	6.45E-05	1.47	Poorly sol.
**49**	135.41	2.26	1.87	-4.74	7.27E-03	1.82	Mod. sol.
**50**	135.41	2.81	2.24	-5.31	2.48E-03	4.89	Mod. sol.
**51**	135.41	2.75	1.89	-5.25	2.50E-03	5.64	Mod. sol.
**52**	135.41	3.63	2.8	-6.16	3.52E-04	6.89	Poorly sol.
**2**	135.41	3.57	2.72	-6.1	3.71E-04	7.95	Poorly sol.
**4**	97.79	4.46	3.71	-6.23	2.47E-04	5.85	Poorly sol.
**70**	82.1	5.77	4.65	-7.26	2.31E-05	5.46	Poorly sol.
**75**	101.83	4.8	4.43	-6.67	9.78E-05	2.14	Poorly sol.

Table S3. Calculated pharmacokinetic properties for all synthesised final hydrochloride salts. All properties were calculated with SwissADME.[1]

Code	HIA	BBB perm.	PGp sub.	CYP1A2 inh.	CYP2C19 inh.	CYP2C9 inh.	CYP2D6 inh.	CYP3A4 inh.
**Sorafenib**	Low	No	No	Yes	Yes	Yes	Yes	Yes
**1**	High	No	No	No	No	No	No	No
**5**	High	No	No	No	No	No	No	No
**6**	High	No	No	No	No	No	No	No
**7**	High	No	No	No	No	No	No	No
**8**	High	No	No	No	No	No	No	No
**9**	High	No	No	No	No	No	No	No
**10**	High	No	No	No	No	No	No	No
**11**	High	No	No	No	No	No	No	No
**28**	High	No	No	Yes	Yes	Yes	Yes	Yes
**3**	High	No	No	No	Yes	Yes	Yes	No
**29**	High	No	No	No	No	Yes	Yes	No
**30**	Low	No	No	No	Yes	Yes	No	No
**36**	High	No	No	No	Yes	Yes	Yes	No
**49**	High	No	Yes	Yes	No	No	No	No
**50**	High	No	Yes	Yes	No	No	No	Yes
**51**	High	No	Yes	Yes	No	No	No	Yes
**52**	High	No	No	Yes	No	No	No	Yes
**2**	High	No	No	Yes	No	No	No	Yes
**4**	High	No	No	Yes	No	No	Yes	Yes
**70**	High	No	No	No	Yes	Yes	Yes	No
**75**	Low	No	No	No	Yes	Yes	No	No

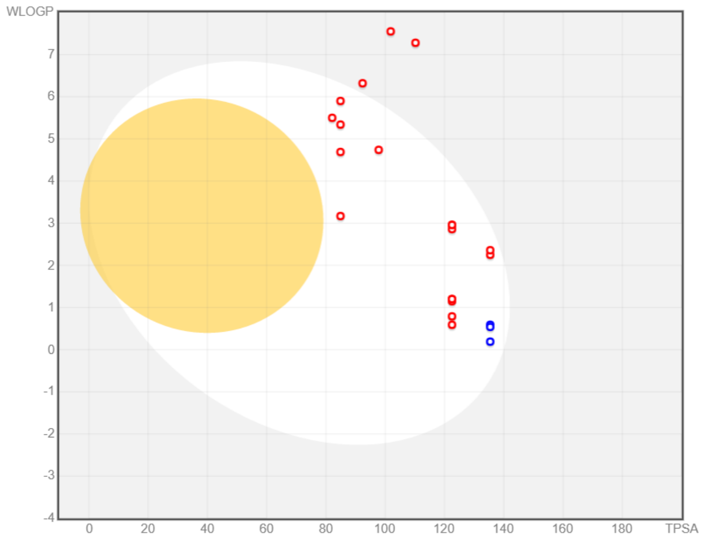

Figure S6. BOILED egg representation of lipophilicity vs. polarity. The white region is for high probability of passive absorption by the GI tract, and the yellow region (yolk) is for high probability of brain penetration; the points are coloured in blue if predicted as actively effluxed by P-gp (PGP+) and in red if predicted as non-substrate of P-gp (PGP−).

4. Biochemical protocols

4.1. Materials

All reagents were purchased from Sigma Aldrich or Gibco (Life Technologies) and sterile when required. Sorafenib was purchased from LC laboratories. Buffers where prepared when needed according to the following recipes: 20X Annexin Binding Buffer (pH 7.4 – 50 mL: 2.60 g HEPES 10.9 mM, 8.18 g NaCl 140 mM, 0.28 g $CaCl_2$ 2.5 mM); 1X Annexin Binding Buffer (40 mL: 2 mL 20X Binding Buffer, 38 mL PBS); Annexin V (600 µL: 18 µL Annexin V, 582 µL 1X Annexin Binding Buffer); Propidium Iodide (3 µL Propidium Iodide, 6 mL 1X Annexin Binding Buffer); Laemmli buffer [Tris-HCl 50 mM (pH 6.7), Glycerol 10% (w/v), sodium dodecyl sulphate 2% (w/v), Bromophenol blue 0.02% (w/v)]; Radioimmuno precipitation assay buffer (RIPA buffer) was purchased from SigmaAldrich and protease inhibitors freshly added before use; running buffer 10 X for wester blotting (250 mM Tris base, 2 M glycine, 5% v/v SDS); trasnfer buffer 10 X for wester blotting (250 mM Tris base, 2 M glycine).

4.2. General Procedures

4.2.1. Cell Storage/Cryopreservation

When an aliquot of cells was required, the cells were quickly removed from the liquid nitrogen and thawed at 37 °C for 2 min. Just before the samples had fully thawed, their contents were gently pipetted into a sterile 20 mL tube containing growing medium. The cells were then centrifuged at 300 x *g* for 5 min. The supernatant was discarded and the pellet was resuspended in 5 mL of medium. This solution was then added to a T25 flask and the cells monitored closely over the next few days.

The growth medium was stored in the fridge at 4 °C and heated to 37 °C prior to culture work. Cells were grown at 37 °C in a humidified environment maintained at 95% O_2 and 5% CO_2 and passaged at least twice weekly depending on their levels of confluency. When required for sub-culturing, if in suspension, cells were transferred to a sterile tube and centrifuged at 300 x *g* for 5 min. Otherwise, if adherent cells, cells were trypsinised and transferred to a sterile tube and centrifuged at 300 x *g* for 5 min. The supernatant was discarded and the cell pellet was resuspended in 15 mL of fresh medium.

4.2.2. Preparation of the Drugs

Stock solutions (10 mM) of the compounds were prepared in sterile EtOH or DMSO and were then sterile filtered (0.2 µM filters). Required concentration ranges (10-0.01 mM) of each drug were prepared in sterile EtOH or DMSO/ddH_2O and stored at -20 °C until required. Sorafenib was purchased from LC laboratories.

4.3. Growth and Maintenance of the Cell Lines

4.3.1. HL-60 Cell Line

The HL-60 (human caucasian promyelocytic leukemia) cell line was obtained from European Collection of Cell Cultures (Porton Down, Wiltshire, U.K.). It was maintained between 200,000 and 2,000,000 cells/mL in Roswell Park Memorial Institute (RPMI) 1640 medium with stable glutamate (GlutaMax I) supplemented with 10% (v/v) fetal bovine serum (FBS) and 1% (v/v) penicillin/ streptomycin (pen-strep).

4.3.2. MCF-7 Cell Line

The MCF-7 (human breast tumour) cell line was obtained from Prof. Mary Meegan, School of Pharmacy, TCD. It was maintained in Roswell Park Memorial Institute (RPMI) 1640 medium with stable glutamate (GlutaMax I) supplemented with 10% (v/v) fetal bovine serum (FBS), 1% (v/v) penicillin/ streptomycin (pen-strep) and 2 mM L-glutamine.

4.3.3. HeLa Cell Line

The HeLa (human epitheloid cervix carcinoma) cell line was obtained from Prof. Thorfinnur Gunnlaugsson, School of Chemistry TCD. It was maintainedin Dulbecco's Modified Eagle's Medium (DMEM) supplemented with 10% (v/v) fetal bovine serum (FBS) and 1%(v/v) penicillin/ streptomycin (pen-strep).

4.3.4. HCT116 and HKH-2 Cell Lines

The HCT116and HKH-2 (human colon cancer cells) cell lines were obtained from Prof. James Murray, School of Biochemistry and Immunology TCD. They were maintainedin Dulbecco's Modified Eagle's Medium (DMEM) supplemented with 10% (v/v) fetal bovine serum (FBS), 1% (v/v) penicillin/ streptomycin (pen-strep), 1% (v/v) sodium pyruvate (NaPyr), 1% (v/v) L-glutamine (L-Glu) and, only for HKH-2, 0.6 mg/mL geneticine (G418).

4.3.5. MCF-10A

MCF-10A (human breast epithelial) cell line was obtained from Prof. Mary Meegan, School of Pharmacy, TCD. It was cultured in Dulbecco’s Modified Eagle Medium/Nutrient Mixture F-12 (DMEM/F12; Gibco) supplemented with 5% horse serum (Invitrogen), 20 ng/mL epidermal growth factor (Merck Millipore), 0.5 µg/mL hydrocortisone (Sigma), 100 ng/mL cholera toxin (Sigma), 10 µg/mL insulin (Sigma), and penicillin/streptomycin (1% v/v) (Gibco).

4.4. AlamarBlue® Cell Viability Assay

Cells were counted and seeded in 96 well plates at a density of 2×10^5 cells/mL for HL-60, 2.5×10^4 cells/mL for MCF-7, MCF10A and HeLa, 1×10^5 cells/mL for HCT116 and HKH-2, all of them in their respective media. The 96 well plates were then treated with a 1:100 dilution of stock concentrations of drugs or EtOH (1% v/v)/DMSO (0.1% v/v) as vehicle control in triplicate.

Three blank wells containing 200 µL media with no cells were also set-up as blanks. After a 72 h incubation, 20 µL of AlamarBlue was added to each well. The plates were incubated in darkness at 37 °C for 4-5 h. Using a Molecular Devices microplate reader, the fluorescence (F) was then read at an excitation wavelength of 544 nm and an emission wavelength of 590 nm. Cell viability was then determined by subtracting the mean blank fluorescence (Fb) from the treated sample fluorescence (Fs) and expressing this as a percentage of the fluorescence of the blanked vehicle control (Fc). This is demonstrated in the equation below. The results were then plotted as a nonlinear regression, sigmoidal dose-response curves on Prism, from which the IC_{50} value for each drug was determined.

$$\frac{\mathrm{Fs} - \mathrm{Fb}}{\mathrm{Fc} - \mathrm{Fb}} \text{ x } 100 = \%\ \text{Cell Viability}$$

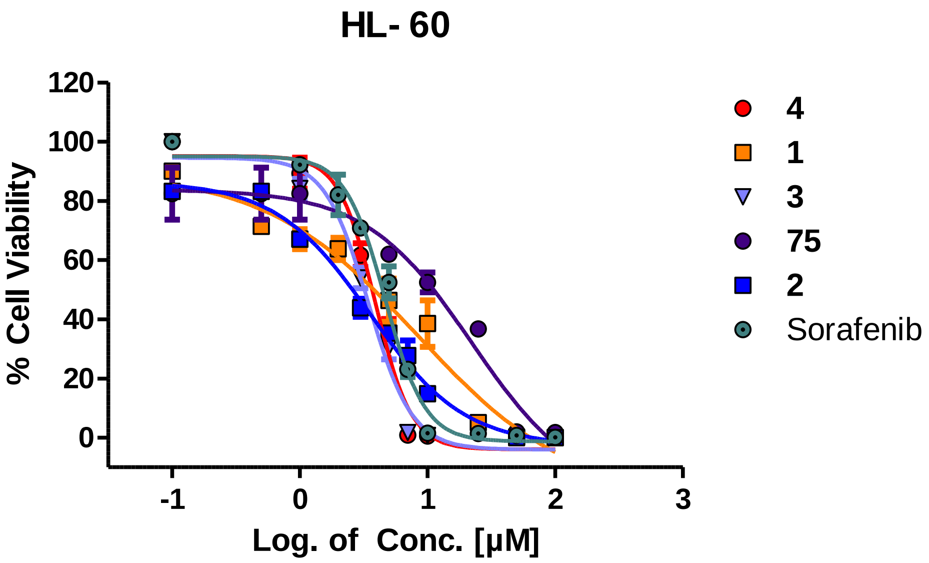

Figure S7. Compounds **4**, **3**, **75**, **2**, lead compound **1** and sorafenib reduce the viability of HL-60 cell line in a dose-dependent manner.

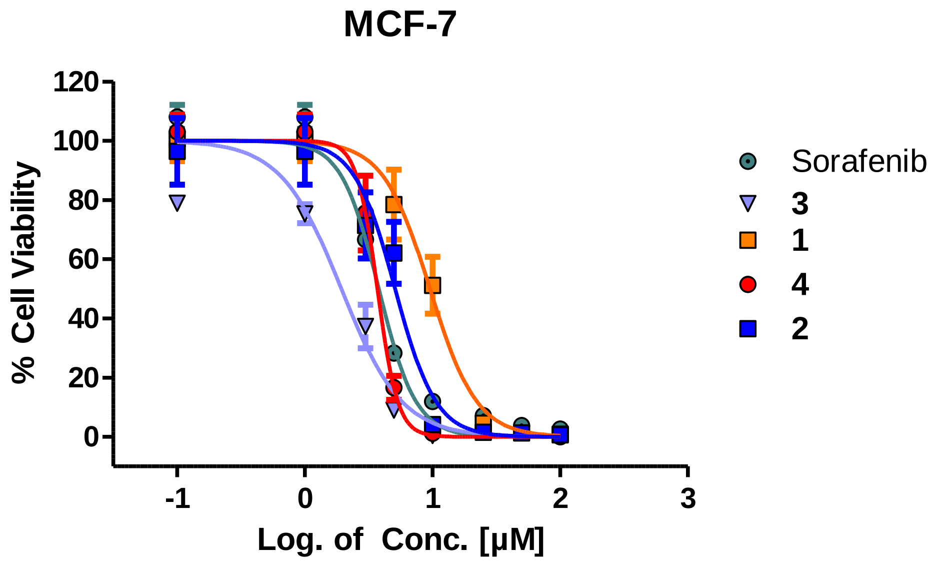

Figure S8. Compounds **3**, **4**, **2**, lead compound **1** and sorafenib reduce the viability of MCF-7 cell line in a dose-dependent manner.

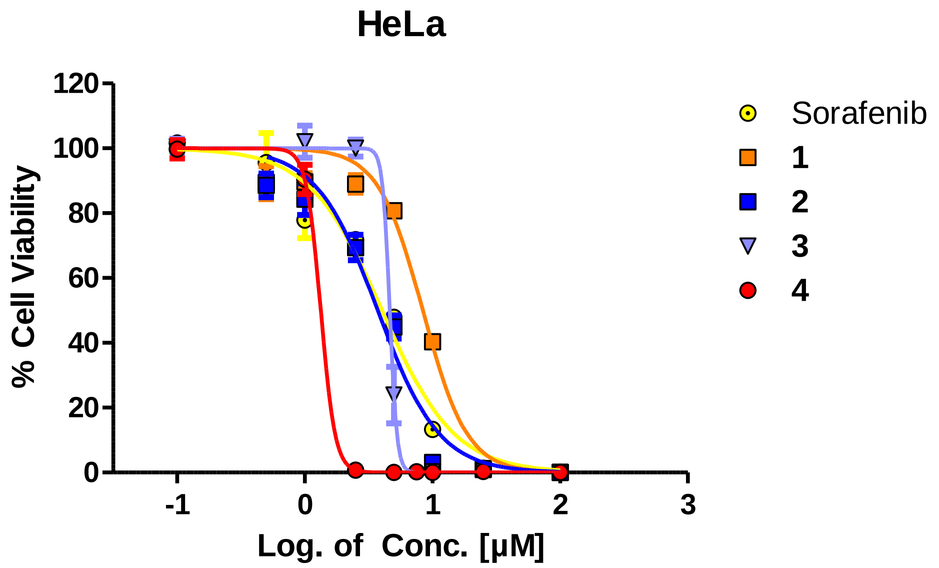

Figure S9. Compounds **2**, **3**, **4**, lead compound **1** and sorafenib reduce the viability of HeLa cell line in a dose-dependent manner.

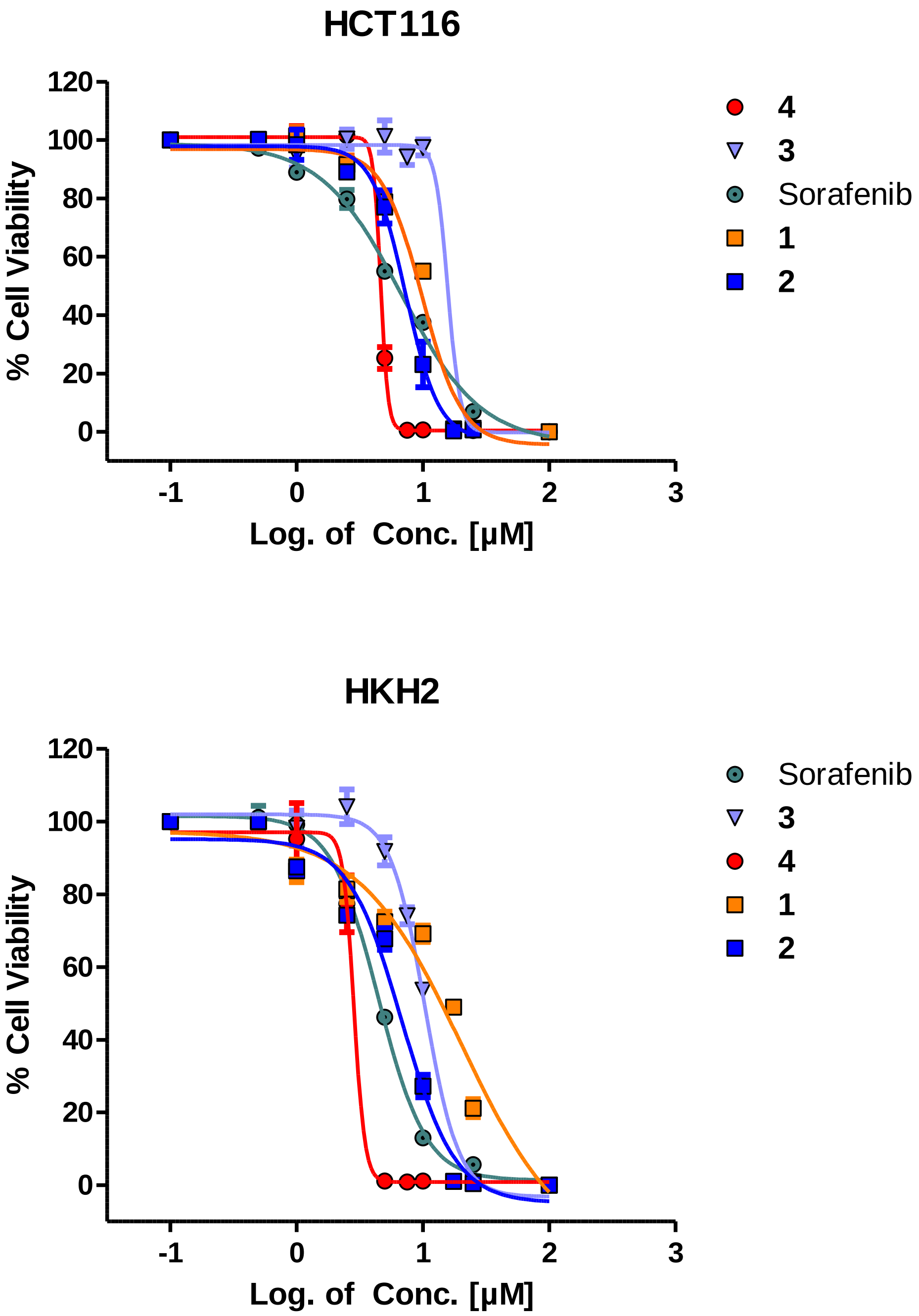

Figure S10. Compounds **3**, **2**, **4**, lead compound **1** and sorafenib reduce the viability of HCT116 (*above*) and HKH2 (*below*) cell lines in a dose-dependent manner.

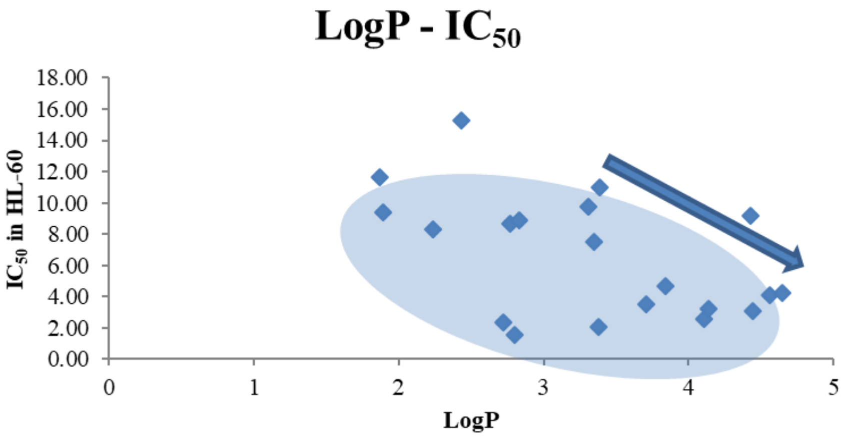

Figure S11. Scatter correlation found between the calculated logP and the HL-60 cytotoxic activity (IC_{50} values) for all of the synthesised compounds with the exception of the inactive ones (**5** and **6**, IC_{50} > 100 μM).

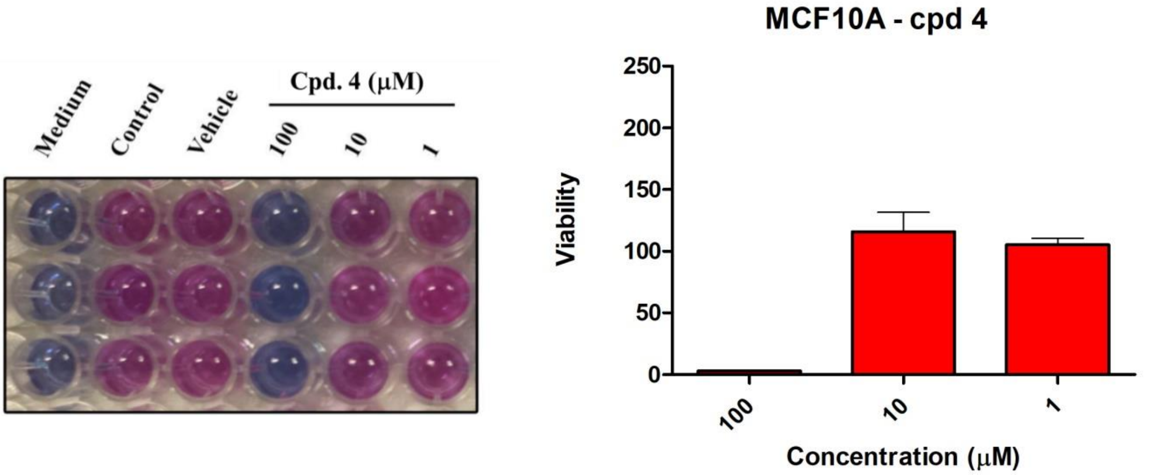

Figure S12. A 96 well plate, where an increase of compound concentration/cytotoxicity is observed due to the colour change of the alamarBlue® dye (blue-pink). MCF-10A cells were seeded at a density of 25 × 103 cells/mL in a 96-well plate and treated with different concentrations of compound **4** dissolved in EtOH (1% v/v). Once treated, cells were incubated for 24 hours at 37 °C after which they were treated with alamarBlue® and left in darkness in an incubator for 5 h.

4.5. Flow cytometry

Apoptosis was analysed using annexin V fluorescein isothiocyanate (FITC) and propidium iodide (PI). HL-60 cells were seeded at a density of $2x10^5$ cells/mL in 12 well plates. Cells were then treated with either vehicle (0.5% ethanol), **2** (5 µM), **3** (5 µM) or **4** (4 µM) for 48 hours. Following treatment, HL60 cells were collected and washed with annexin V binding buffer (5 mM HEPES, 70 mM NaCl, 1.25 mM CaCl2 pH 7.4) and stained with annexin V-FITC (iQ Corporation, Groningen, The Netherlands) for 20 minutes. Following washing with annexin V binding buffer, cells were resuspended in PI (0.5 µg/mL) in binding buffer and analysed on BD FACS Canto II flow cytometer (BD Sciences) using FloJo software (Ashland, Oregon, United States).

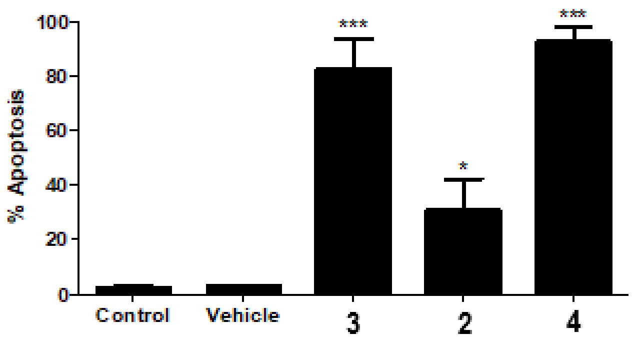

Figure S13. Compounds **3** and **4** promote strong apoptosis when inhibiting HL-60 cell viability. For flow cytometry, HL60 cells were seeded at a density of $2x10^5$ cells/mL in 12 well plates. Cells were then treated with either vehicle (0.5% ethanol), **2** (5 µM), **3** (5 µM) or **4** (4 µM) for 48 hours. After 48 hours, cells were collected and stained with annexin V – FITC and propidium iodide. Samples were analysed by flow cytometry using the BD FACs Canto II cytometer. Columns and bars represent the mean ± S.D. of 3 independent experiments. Statistical analysis was performed using one-way ANOVA with Dunnett's multiple comparisons test comparing vehicle to drug treated samples. *$p<0.05$ and ***$p<0.001$.

4.6. Western blots

HL-60 cells were seeded in T25 flasks at 50 x10^4 cells/mL and treated with either vehicle [0.5% EtOH (v/v)], sorafenib (5 µM), **2** (5 µM), **3** (5 µM) or **4** (5 µM). After 16 hours, cells were collected and washed with PBS. Cell pellets were re-suspended in cold cell lysis buffer (radio-immunoprecipitation assay buffer) supplemented with 1% phosphatase inhibitor cocktail 2 and 3 (Sigma) and 10% protease inhibitor (Roche). Cells were lysed for 30 minutes on ice. Protein concentration was then determined by BCA assay. Lysates were boiled with laemmli sample buffer [Tris-HCL 50 mM (pH 6.7), glycerol 10% (w/v), sodium dodecyl sulphate 2% (w/v), bromophenol blue 0.02% (w/v)] containing DTT 50 µM for 10 min. at 90 °C. Also 20 µg of lysates were resolved by sodium dodecyl sulphate polyacrylamide gel electrophoresis (SDS-PAGE) and transferred onto PVDF transfer membrane (EMD Millipore). Membranes were blocked with 5% non-fat milk and probed with primary antibodies for ERK and phospho-ERK (Cell Signaling). Anti-GAPDH was used as a loading control (Calbiochem).

5. NMR Spectra of Final Salts

^{1}H-NMR, ^{13}C-NMR, and ^{19}F-NMR for compound **2**

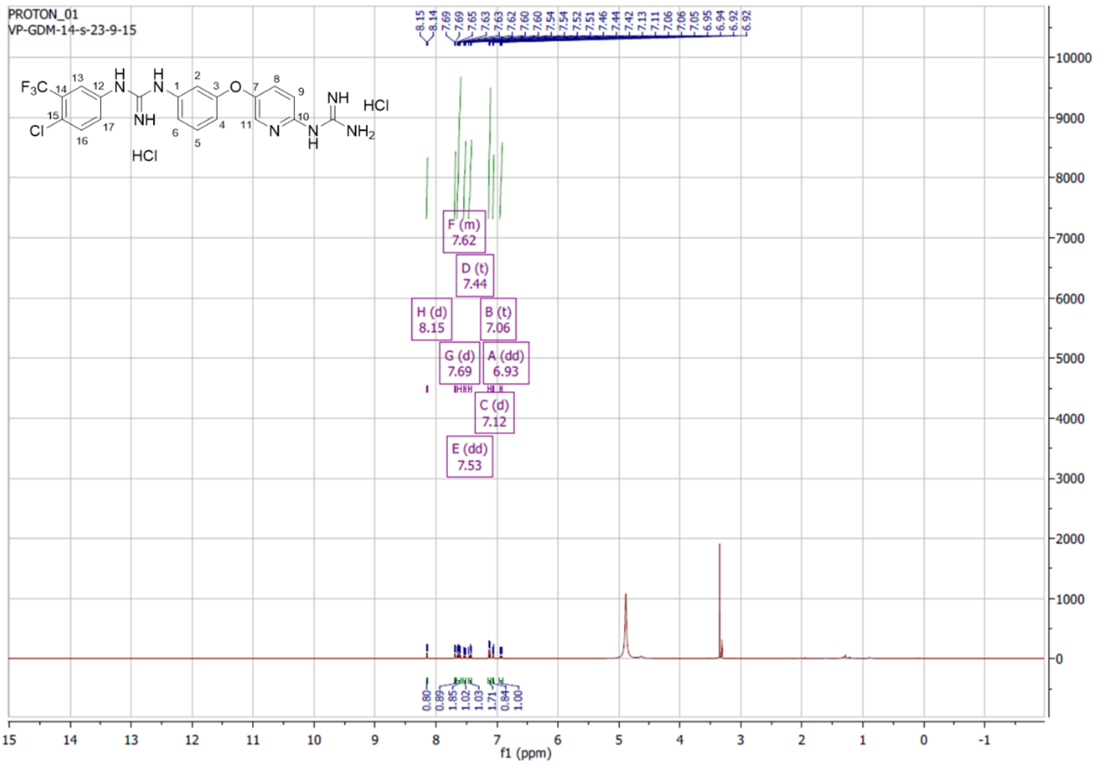

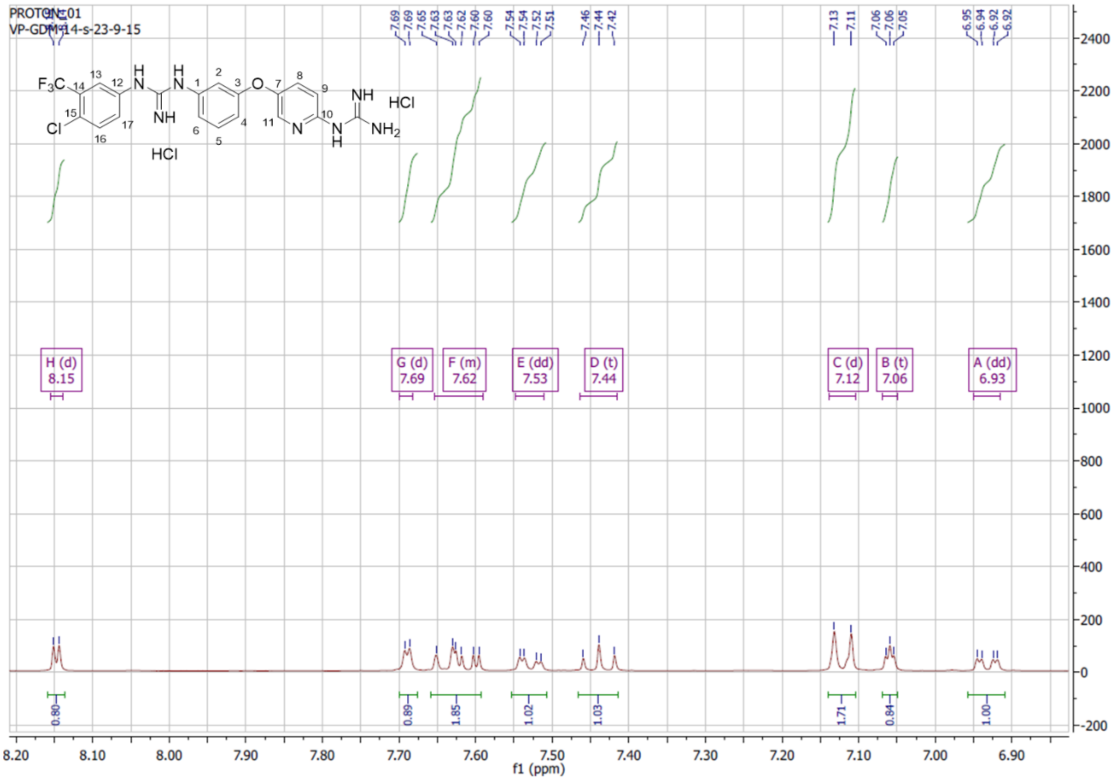

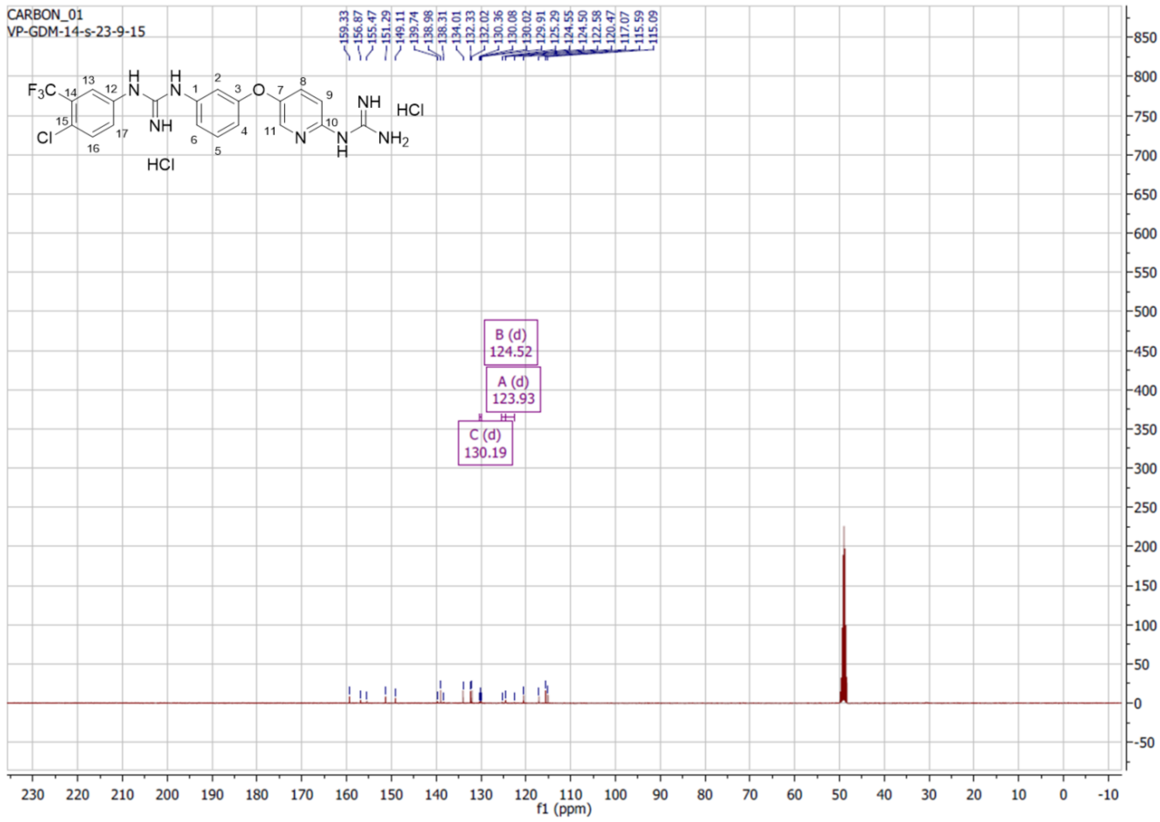
CARBON_01
VP-GDM-14-s-23-9-15
HCl
HCl
B (d)
124.52
A (d)
123.93
C (d)
130.19
f1 (ppm)

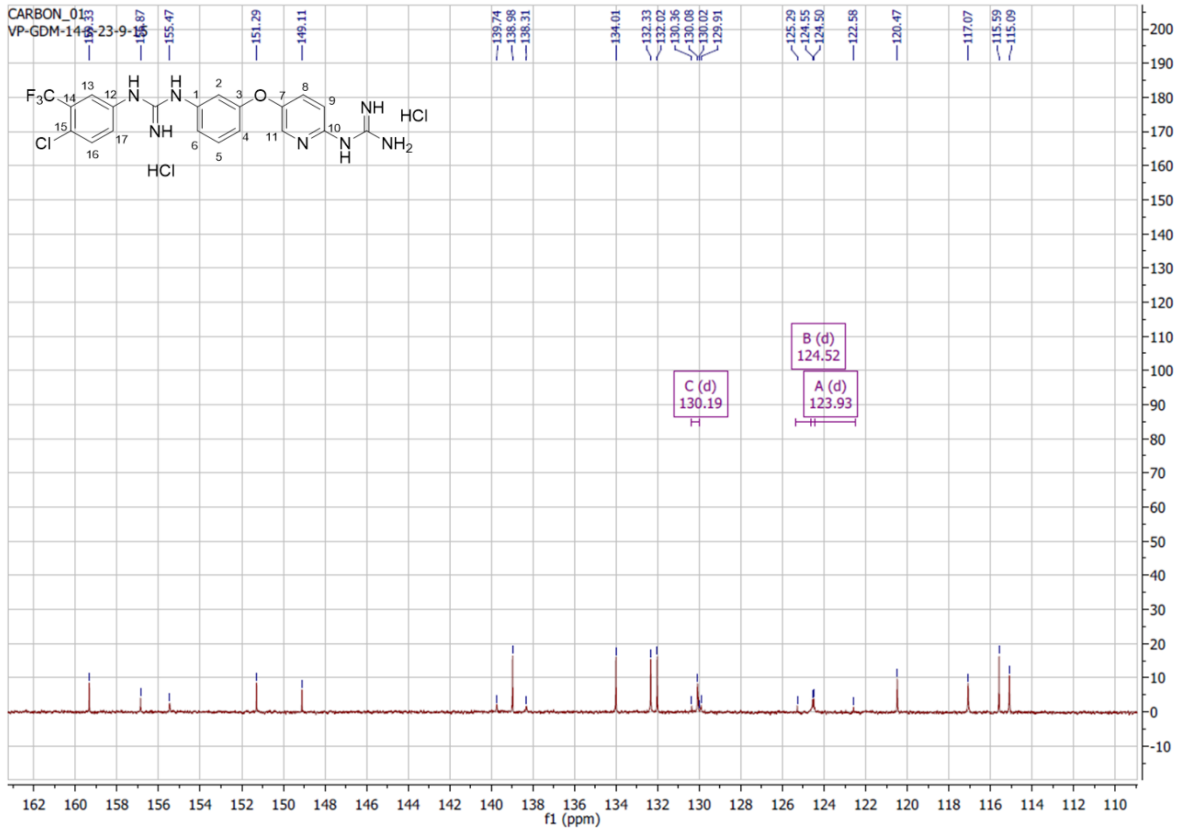
CARBON_01
VP-GDM-14-s-23-9-15
159.33
156.87
155.47
151.29
149.11
139.74
138.98
138.31
134.01
132.33
132.02
130.36
130.08
130.02
129.91
125.29
124.55
124.50
122.58
120.47
117.07
115.59
115.09
HCl
HCl
B (d)
124.52
A (d)
123.93
C (d)
130.19
f1 (ppm)

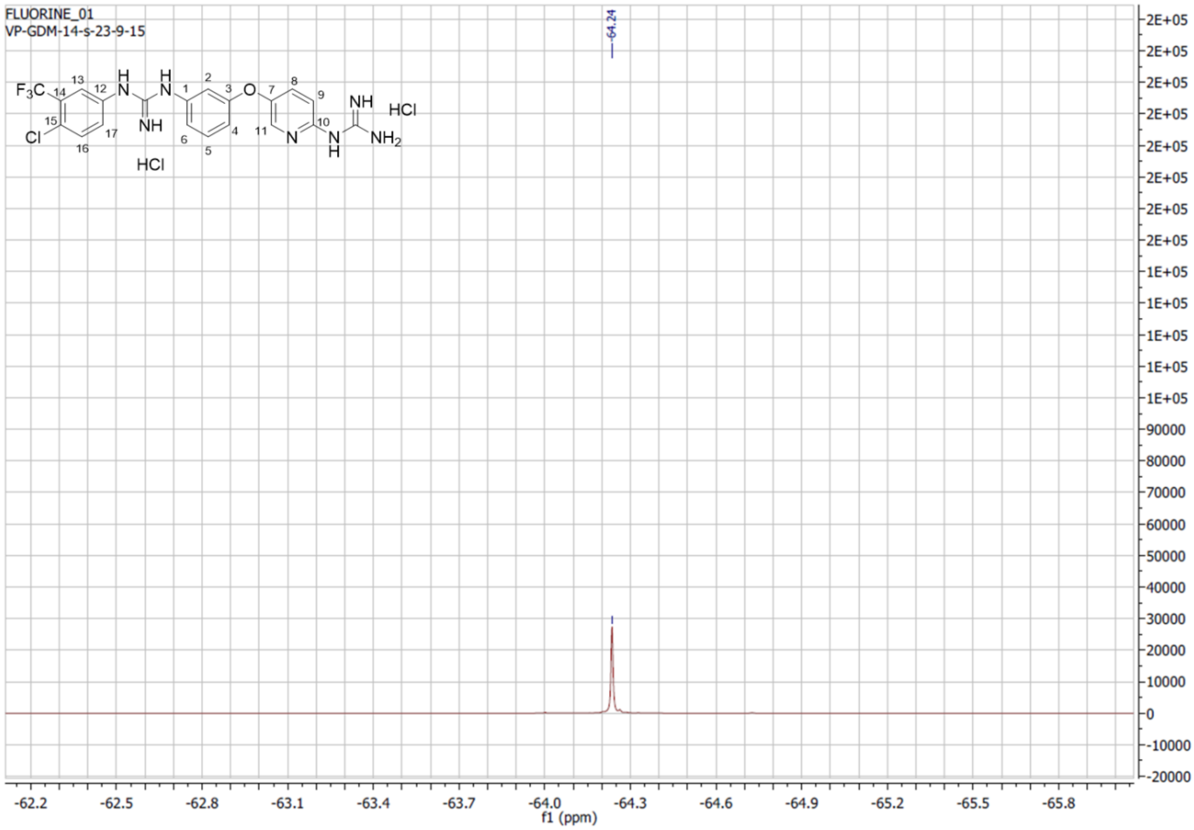
FLUORINE_01
VP-GDM-14-s-23-9-15
-64.24
HCl
HCl
f1 (ppm)

^{1}H-NMR, ^{13}C-NMR, and ^{19}F-NMR for compound **3**

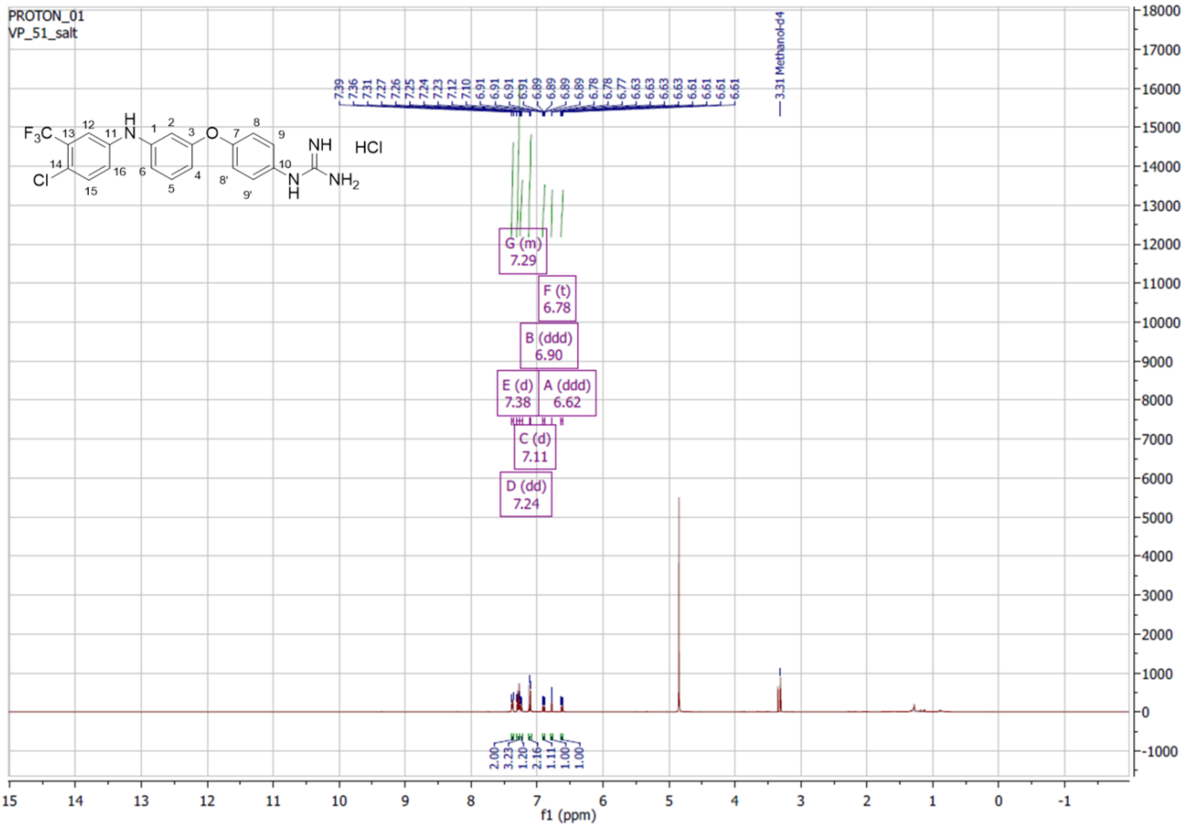

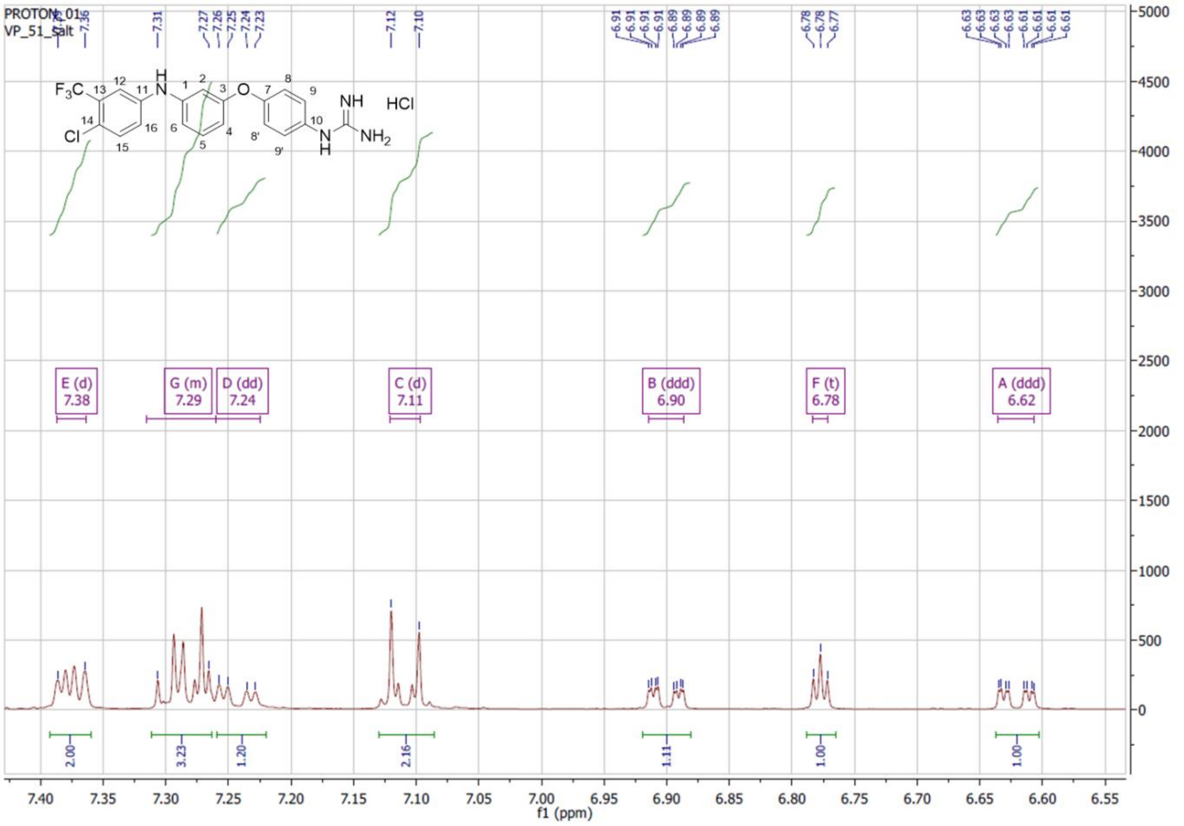

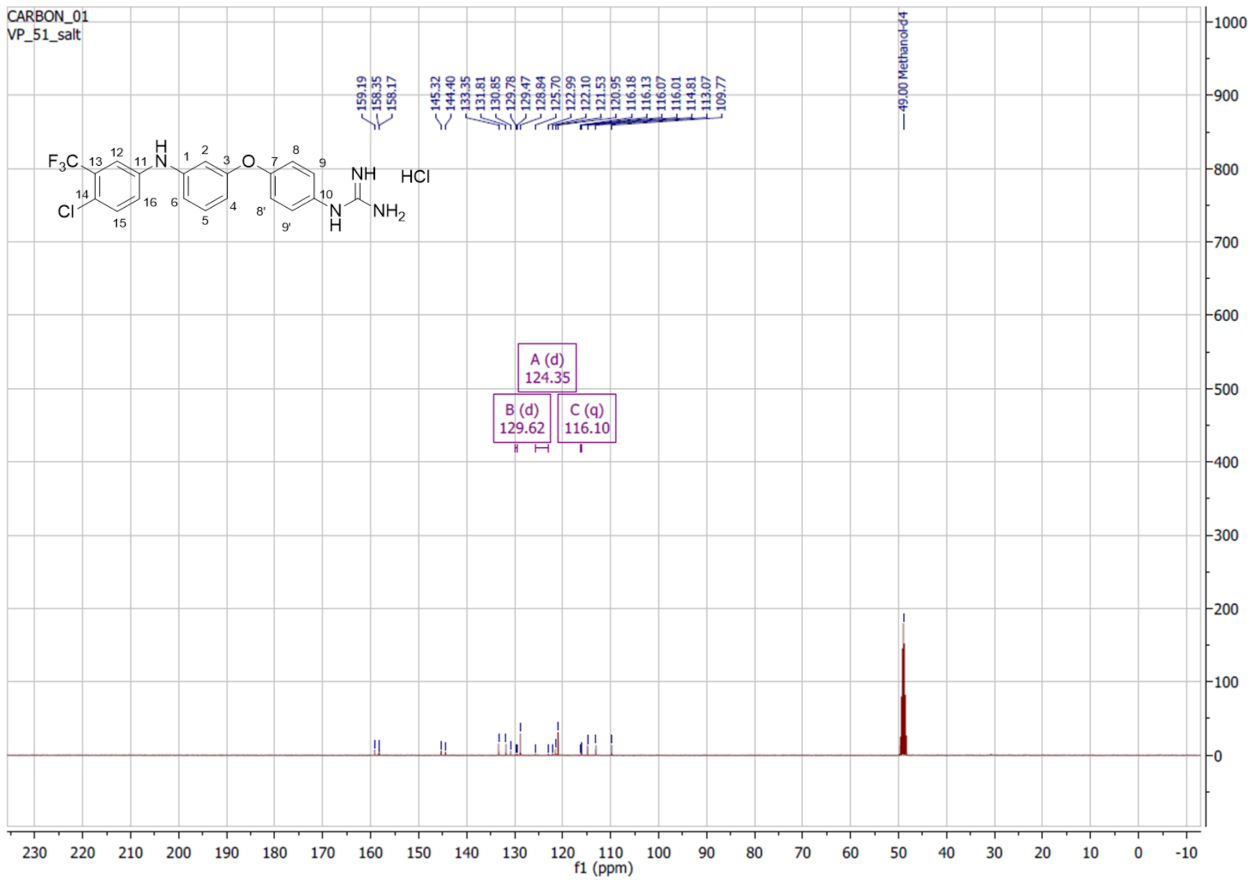
CARBON_01
VP_51_salt
159.19 158.35 158.17 145.32 144.40 133.35 131.81 130.85 129.78 129.47 128.84 125.70 122.99 122.10 121.53 120.95 116.18 116.13 116.07 116.01 114.81 113.07 109.77
49.00 Methanol-d4
HCl
A (d) 124.35
B (d) 129.62
C (q) 116.10
f1 (ppm)

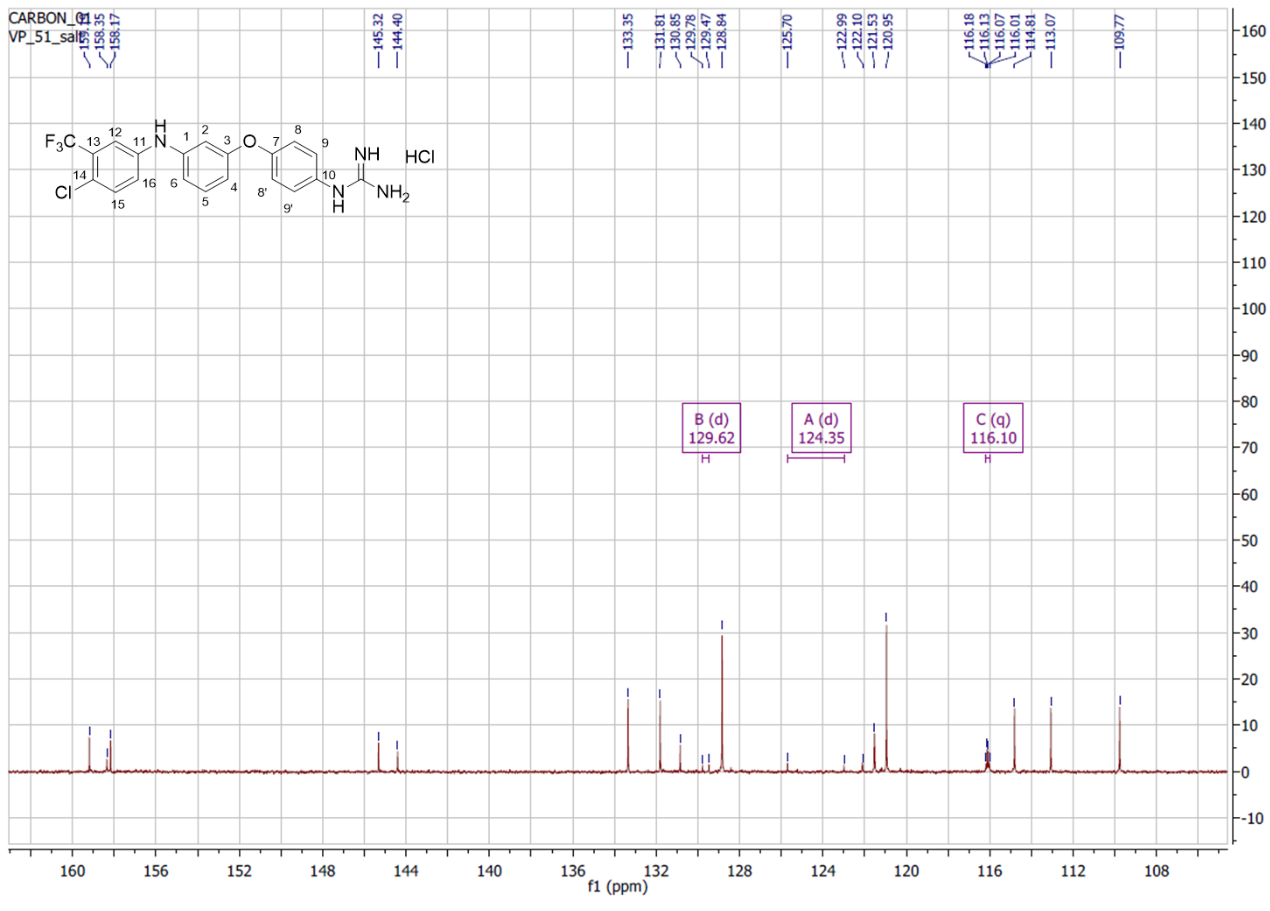
CARBON_01
VP_51_salt
159.19 158.35 158.17 145.32 144.40 133.35 131.81 130.85 129.78 129.47 128.84 125.70 122.99 122.10 121.53 120.95 116.18 116.13 116.07 116.01 114.81 113.07 109.77
HCl
B (d) 129.62
A (d) 124.35
C (q) 116.10
f1 (ppm)

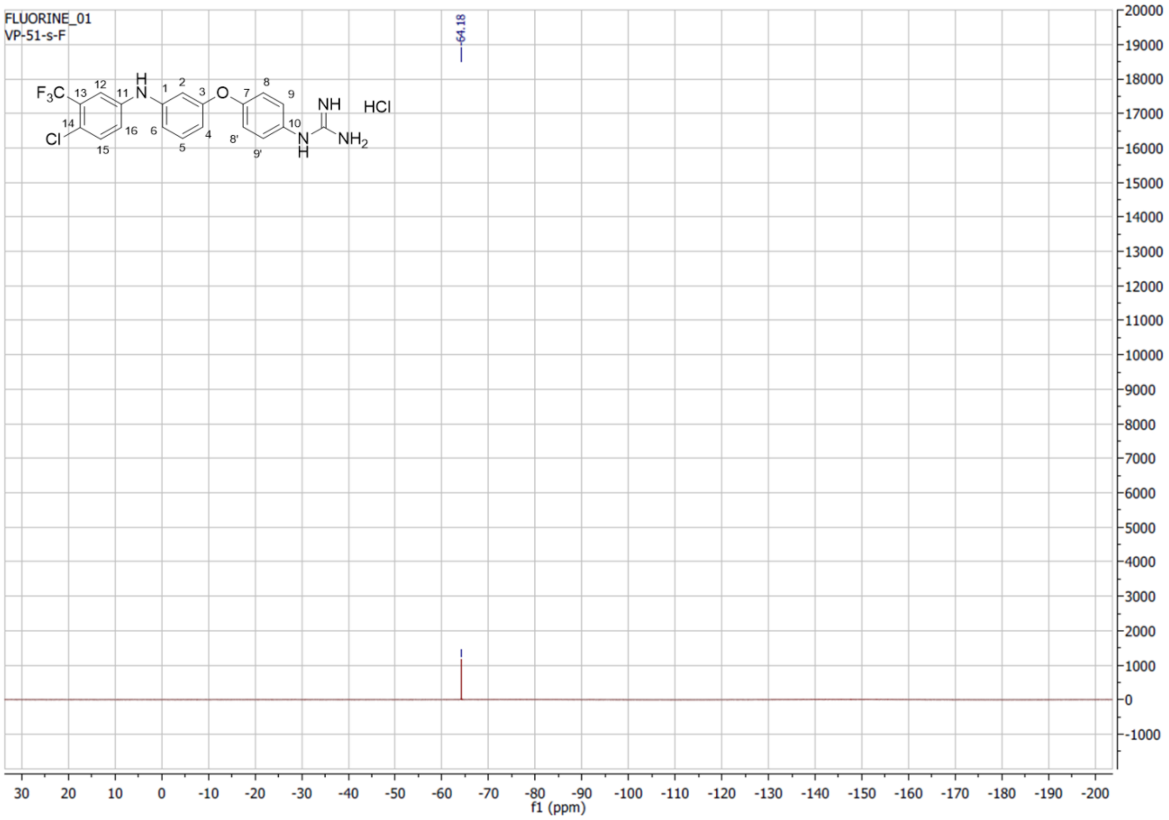
FLUORINE_01
VP-51-s-F
-64.18
HCl
f1 (ppm)

^{1}H-NMR, ^{13}C-NMR, and ^{19}F-NMR for compound **4**

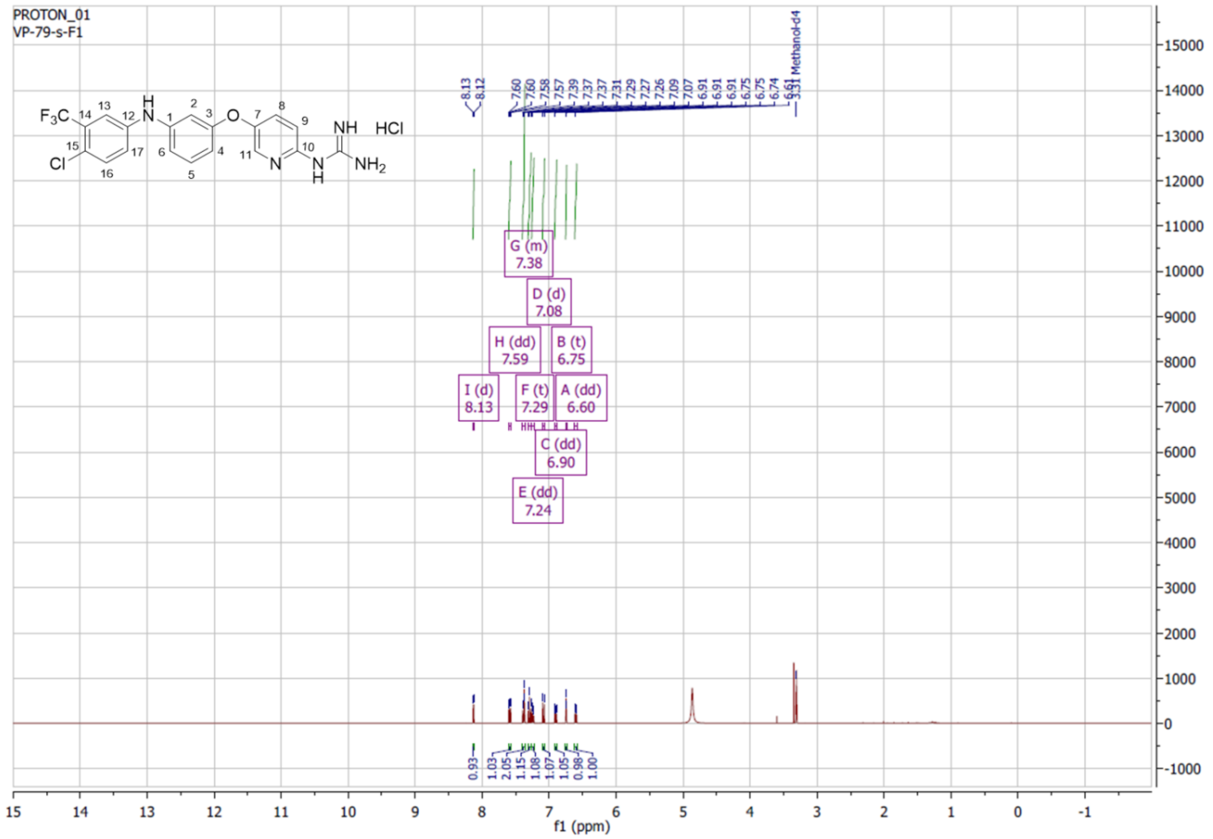

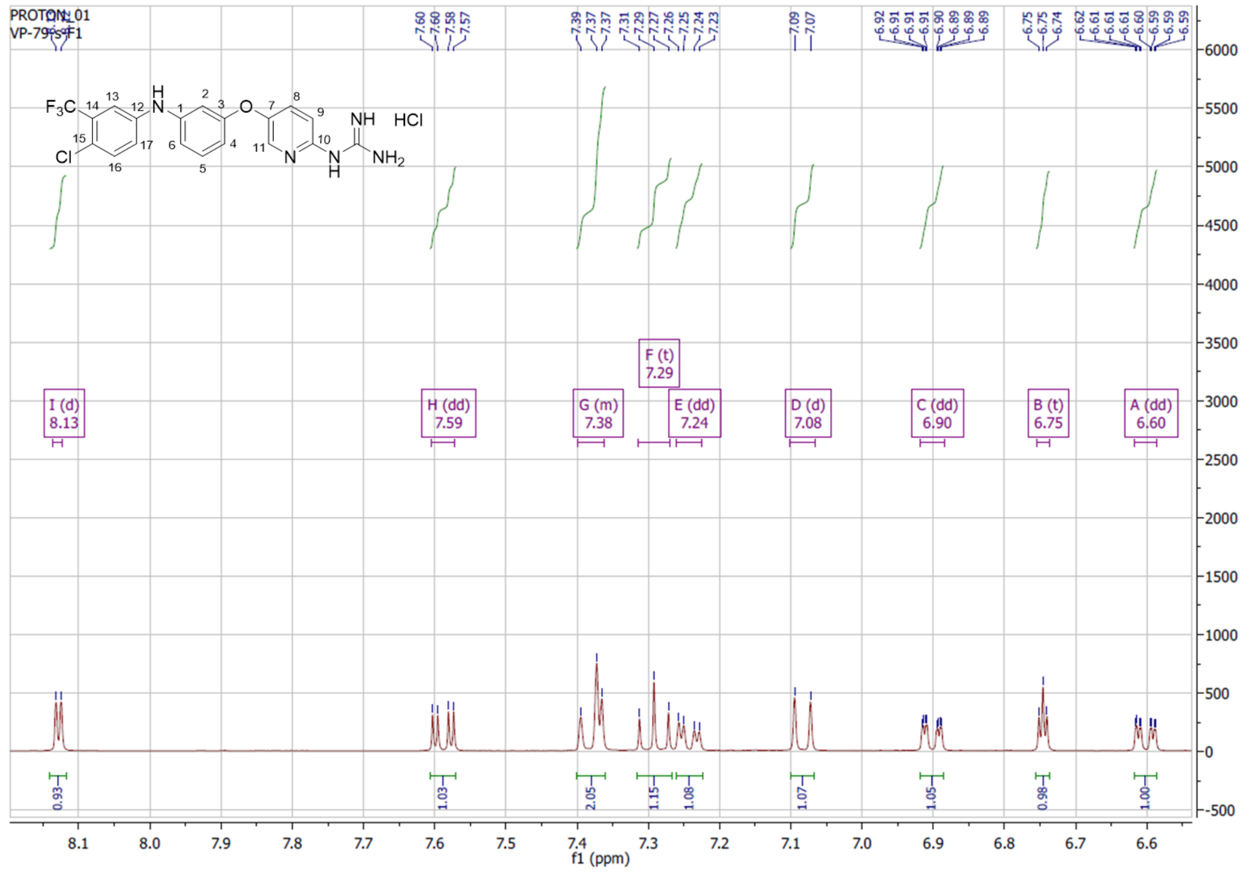

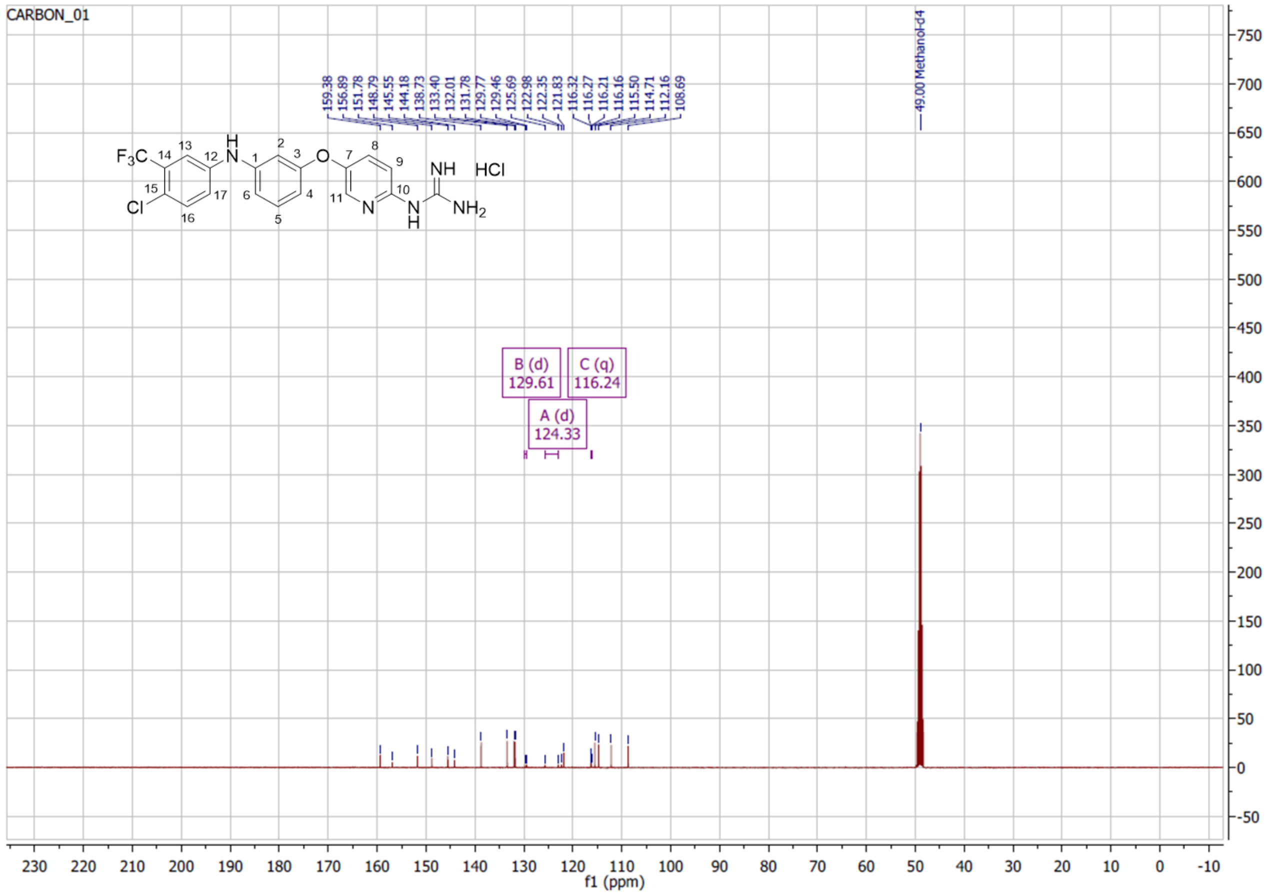
CARBON_01
159.38
156.89
151.78
148.79
145.55
144.18
138.73
133.40
132.01
131.78
129.77
129.46
125.69
122.98
122.35
121.83
116.32
116.27
116.21
116.16
115.50
114.71
112.16
108.69
49.00 Methanol-d4
HCl
B (d)
129.61
C (q)
116.24
A (d)
124.33
f1 (ppm)

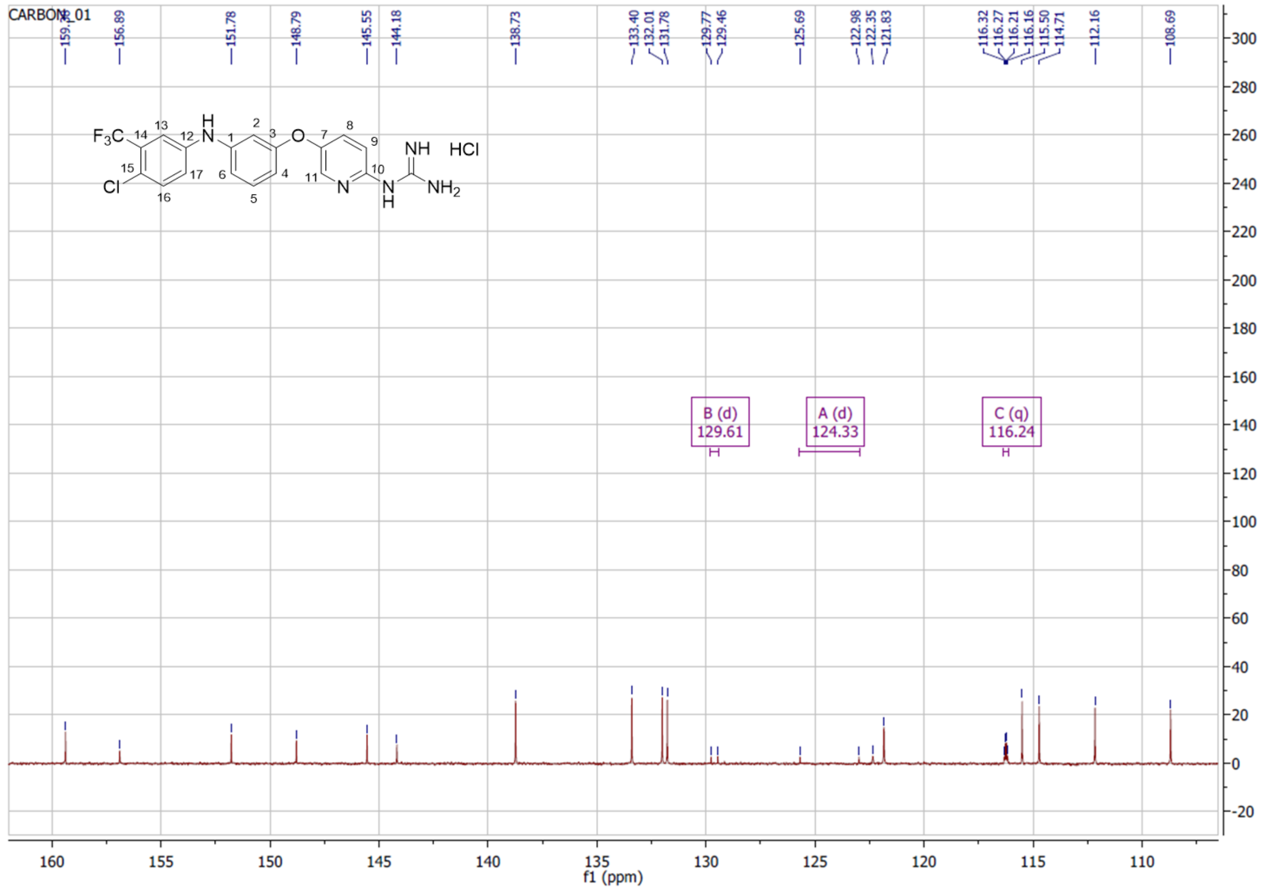
CARBON_01
159.38
156.89
151.78
148.79
145.55
144.18
138.73
133.40
132.01
131.78
129.77
129.46
125.69
122.98
122.35
121.83
116.32
116.27
116.21
116.16
115.50
114.71
112.16
108.69
HCl
B (d)
129.61
A (d)
124.33
C (q)
116.24
f1 (ppm)

FLUORINE_01
VP-79-s-F1

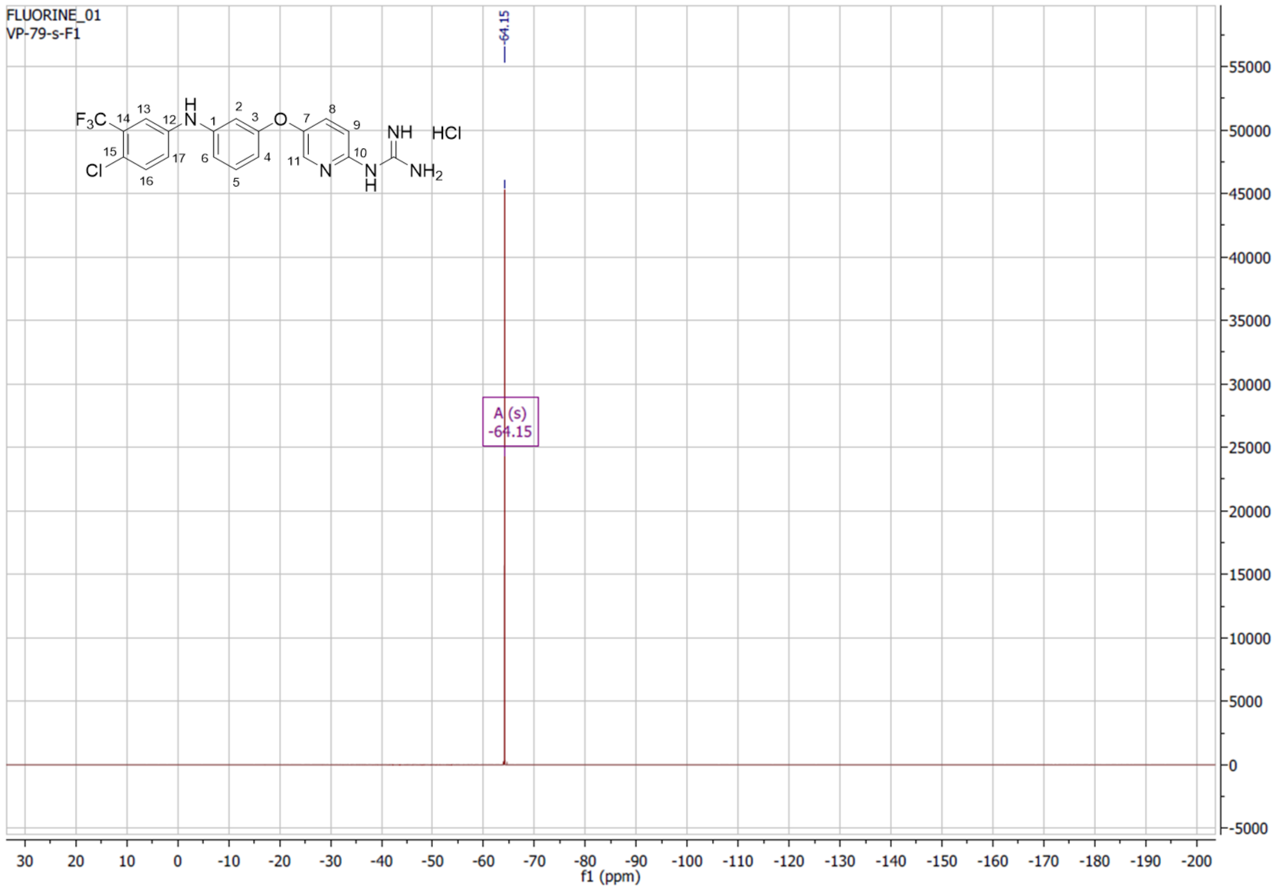

^{1}H-NMR, ^{13}C-NMR, and ^{19}F-NMR for compound **5**

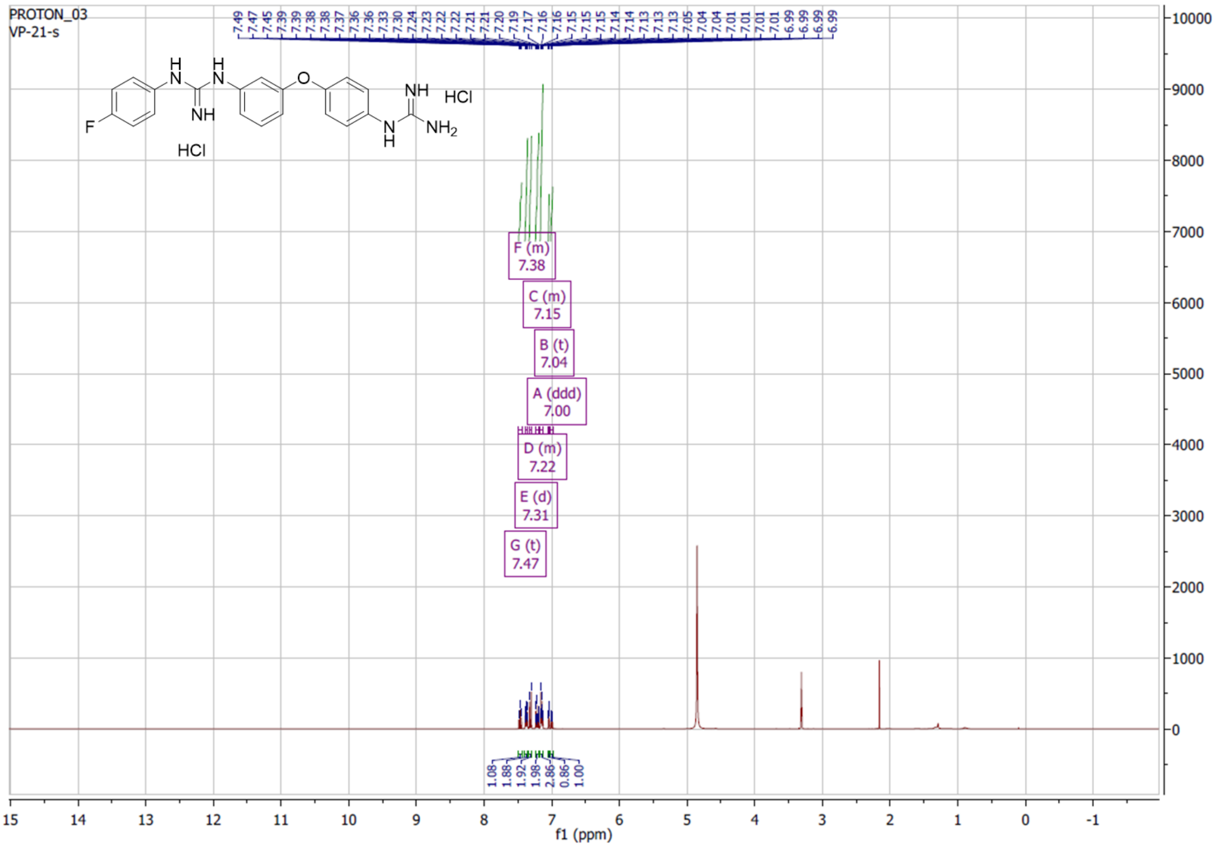

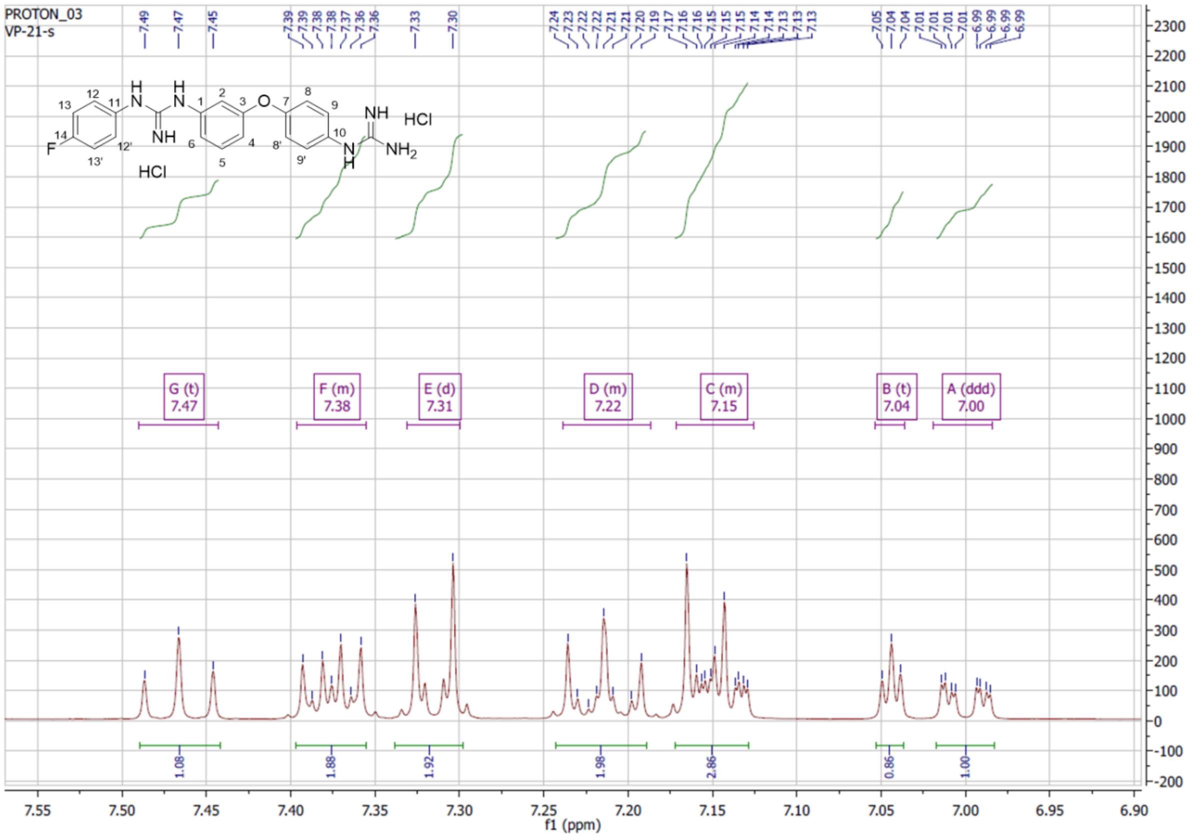

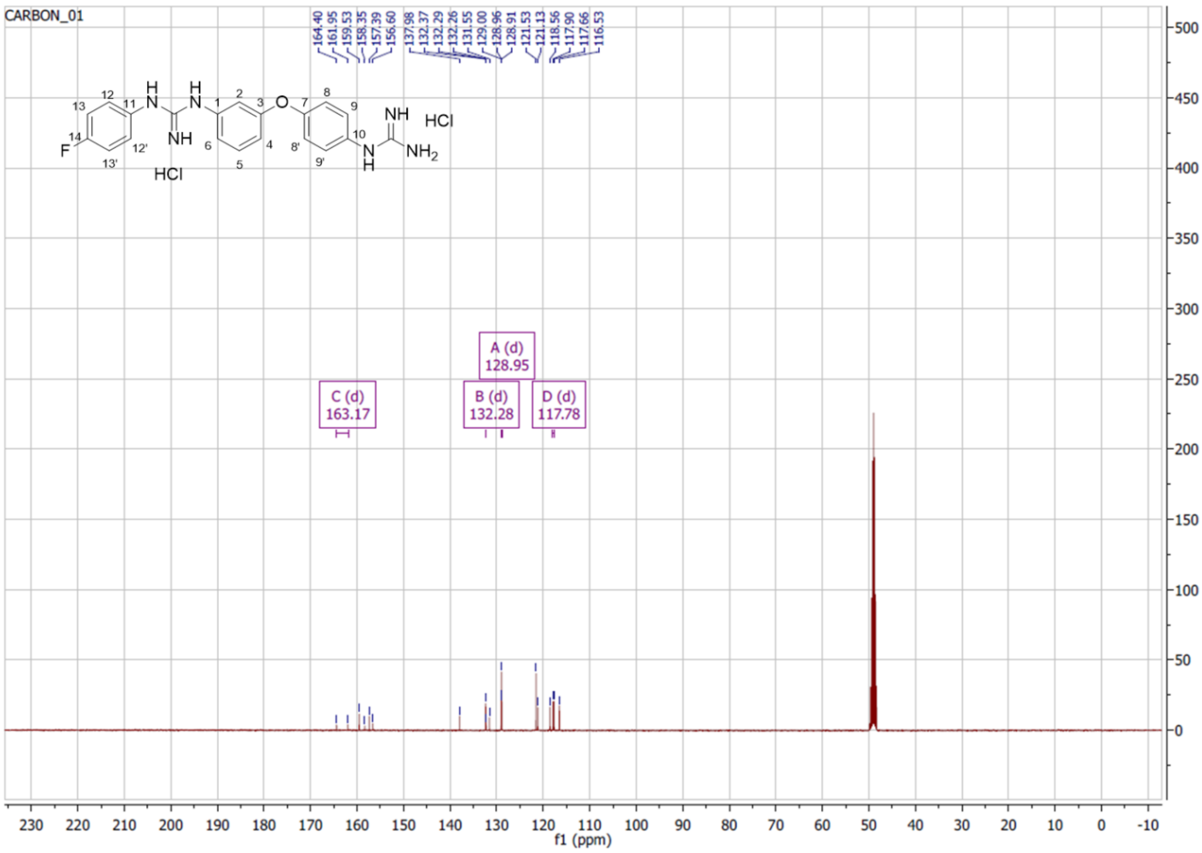
CARBON_01
164.40
161.95
159.53
158.35
157.39
156.60
137.98
132.37
132.29
132.26
131.55
129.00
128.96
128.91
121.53
121.13
118.56
117.90
117.66
116.53
HCl
HCl
A (d)
128.95
B (d)
132.28
C (d)
163.17
D (d)
117.78
f1 (ppm)

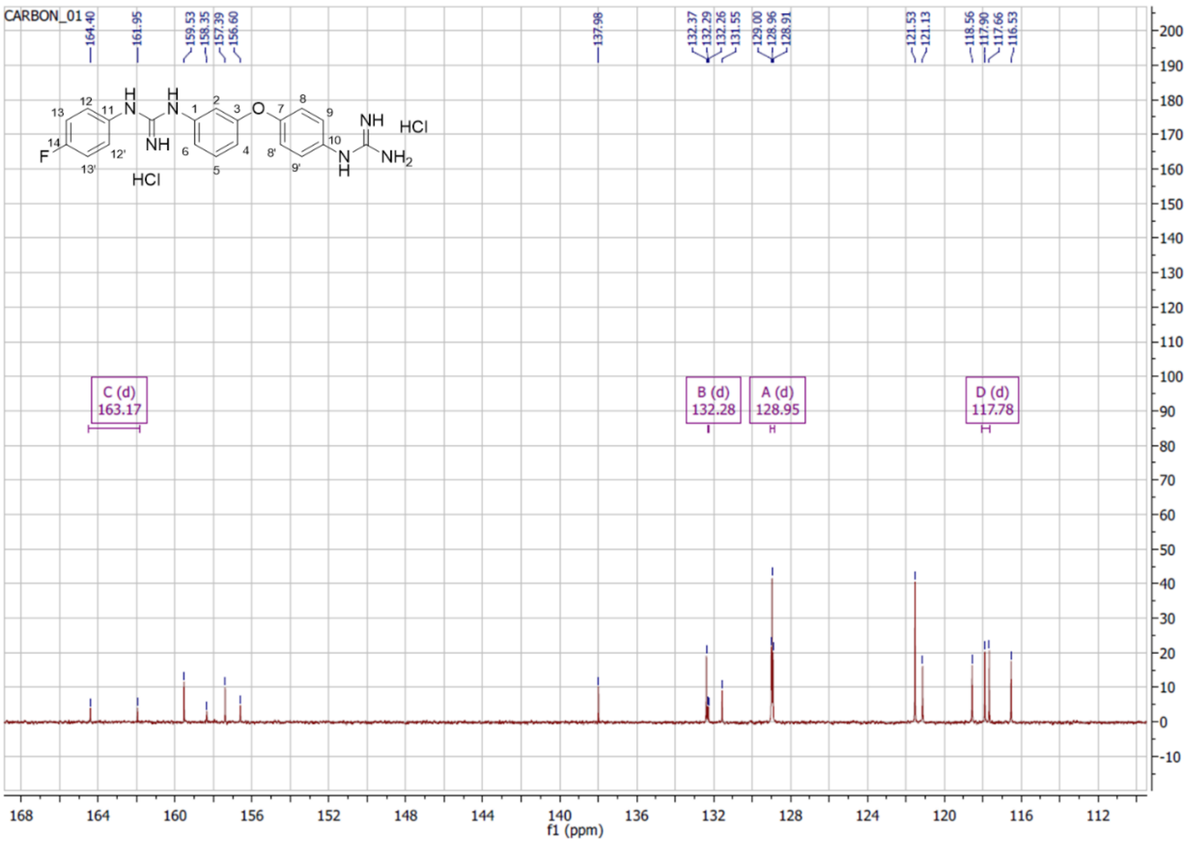
CARBON_01
164.40
161.95
159.53
158.35
157.39
156.60
137.98
132.37
132.29
132.26
131.55
129.00
128.96
128.91
121.53
121.13
118.56
117.90
117.66
116.53
HCl
HCl
C (d)
163.17
B (d)
132.28
A (d)
128.95
D (d)
117.78
f1 (ppm)

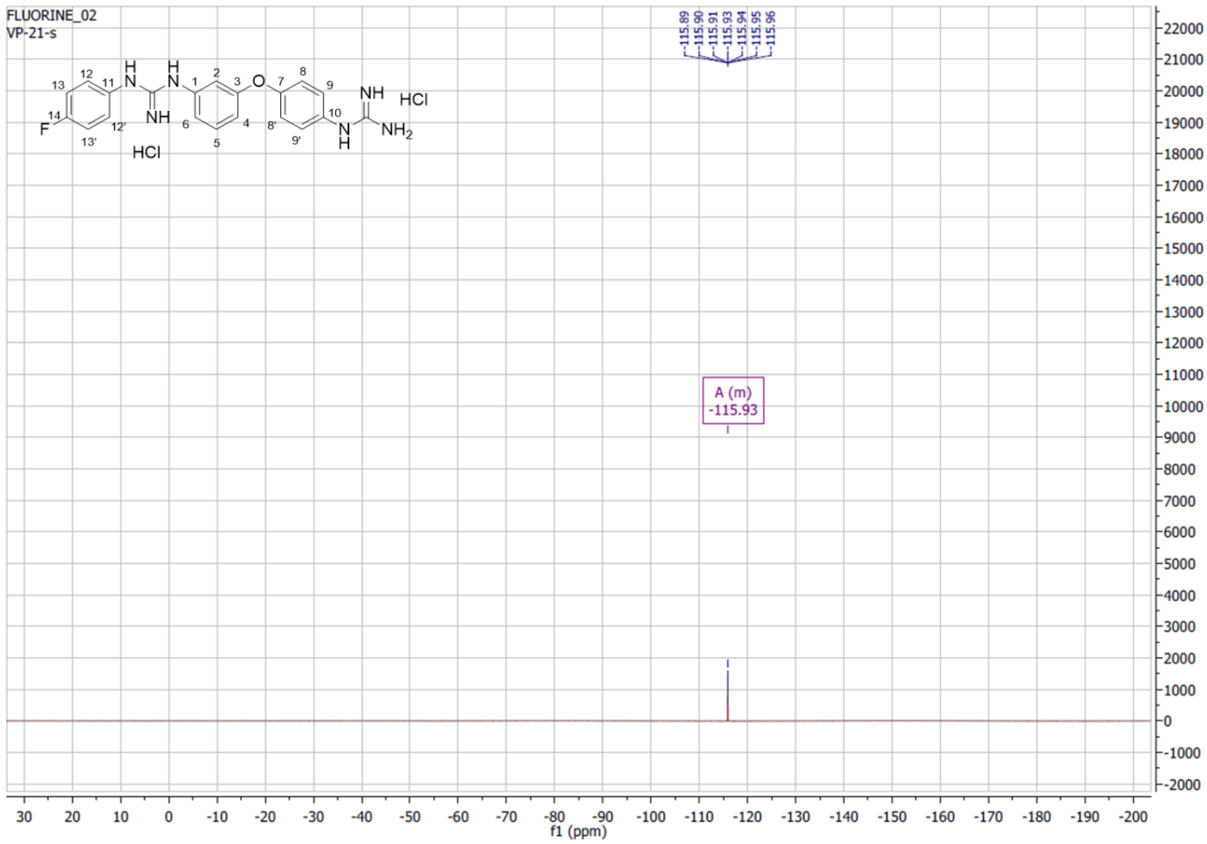
FLUORINE_02
VP-21-s
HCl
HCl
A (m)
-115.93
-115.89
-115.90
-115.91
-115.93
-115.94
-115.95
-115.96
f1 (ppm)

^{1}H-NMR, ^{13}C-NMR, and ^{19}F-NMR for compound **6**

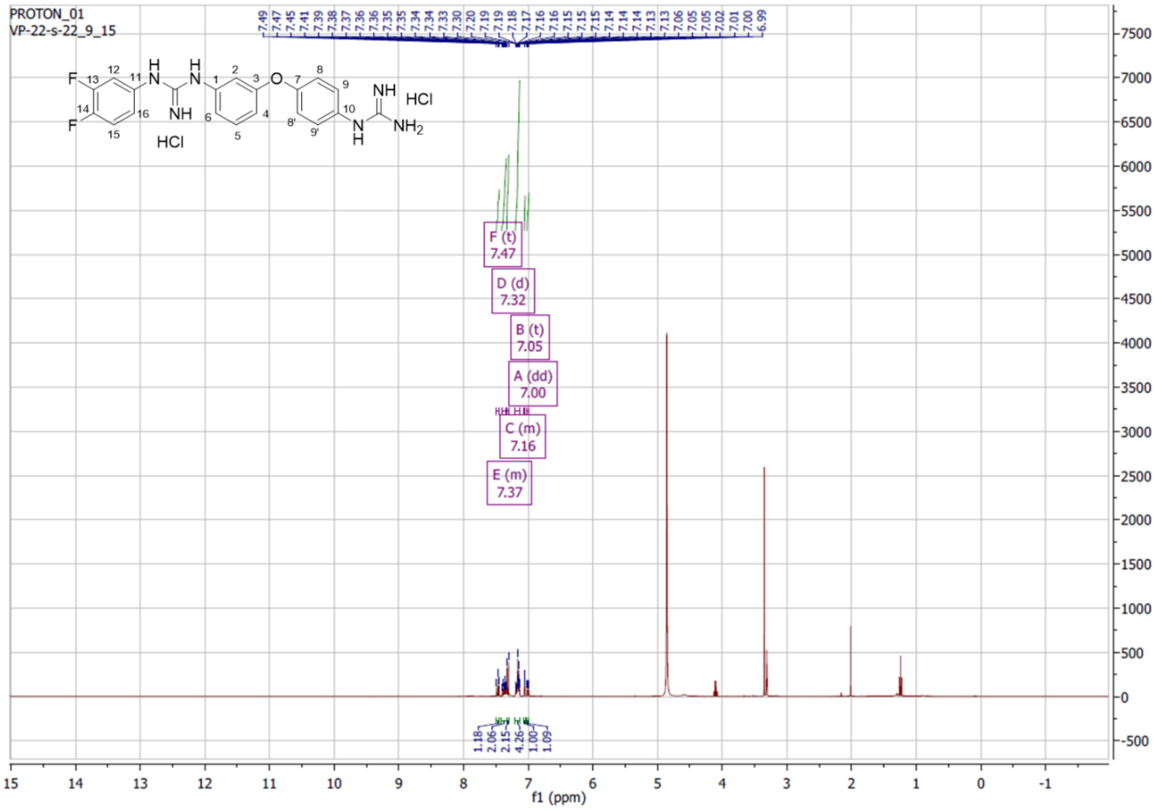

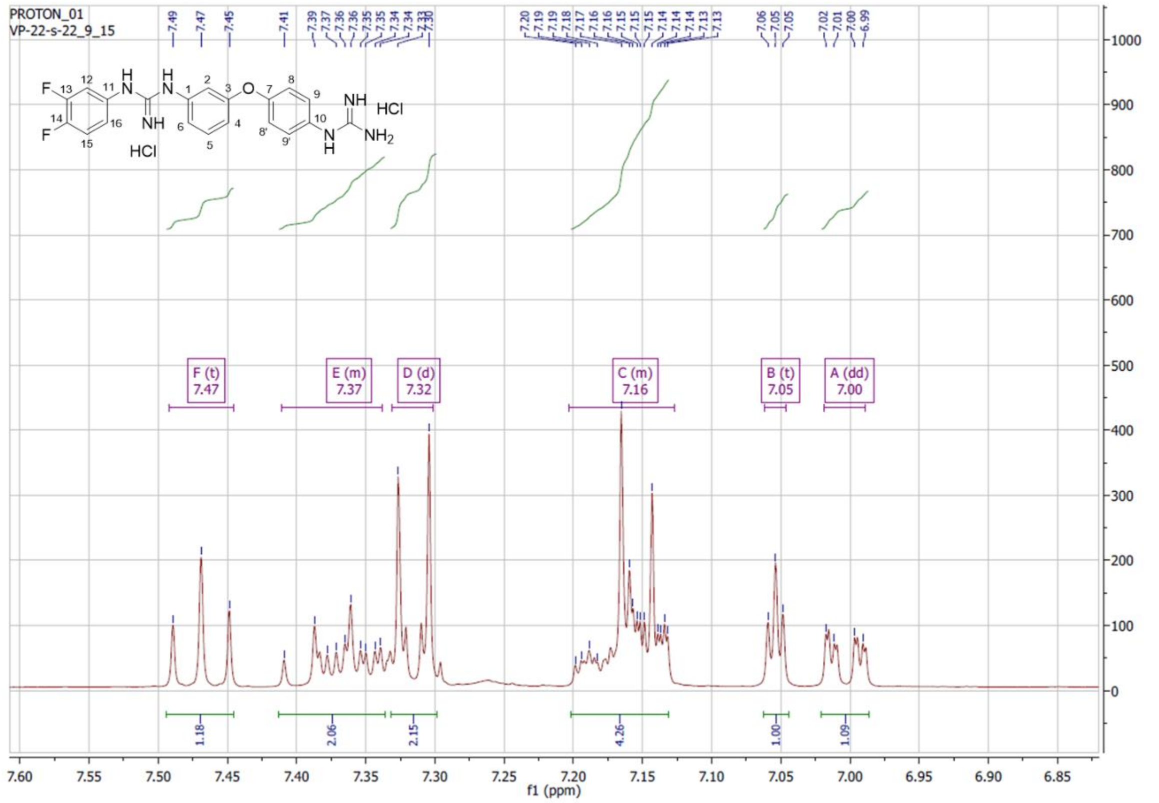

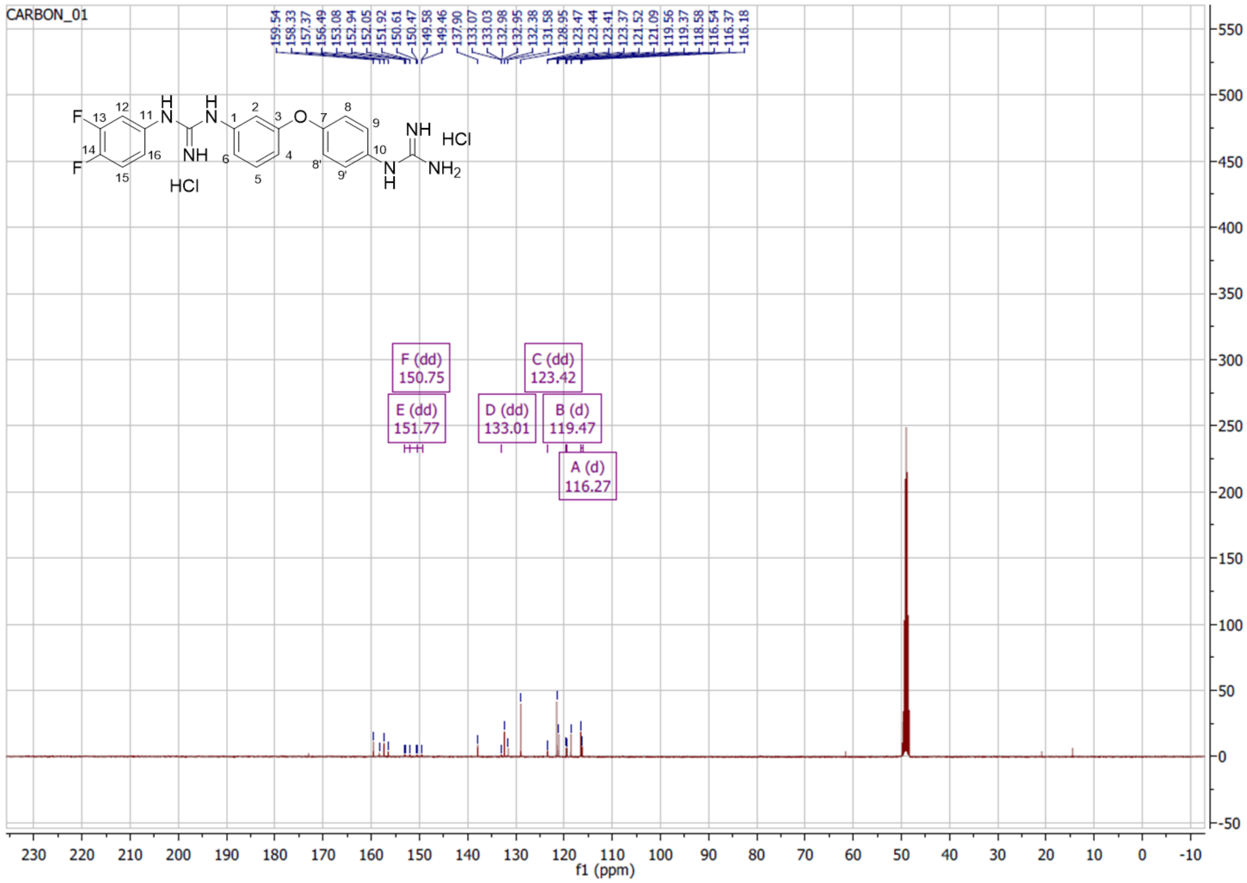

CARBON_01
159.54
158.33
157.37
156.49
153.08
152.94
152.05
151.92
150.61
150.47
149.58
149.46
137.90
133.07
133.03
132.98
132.95
132.38
131.58
128.95
123.47
123.44
123.41
123.37
121.52
121.09
119.56
119.37
118.58
116.54
116.37
116.18
HCl
HCl
F (dd)
150.75
E (dd)
151.77
D (dd)
133.01
C (dd)
123.42
B (d)
119.47
A (d)
116.27
f1 (ppm)

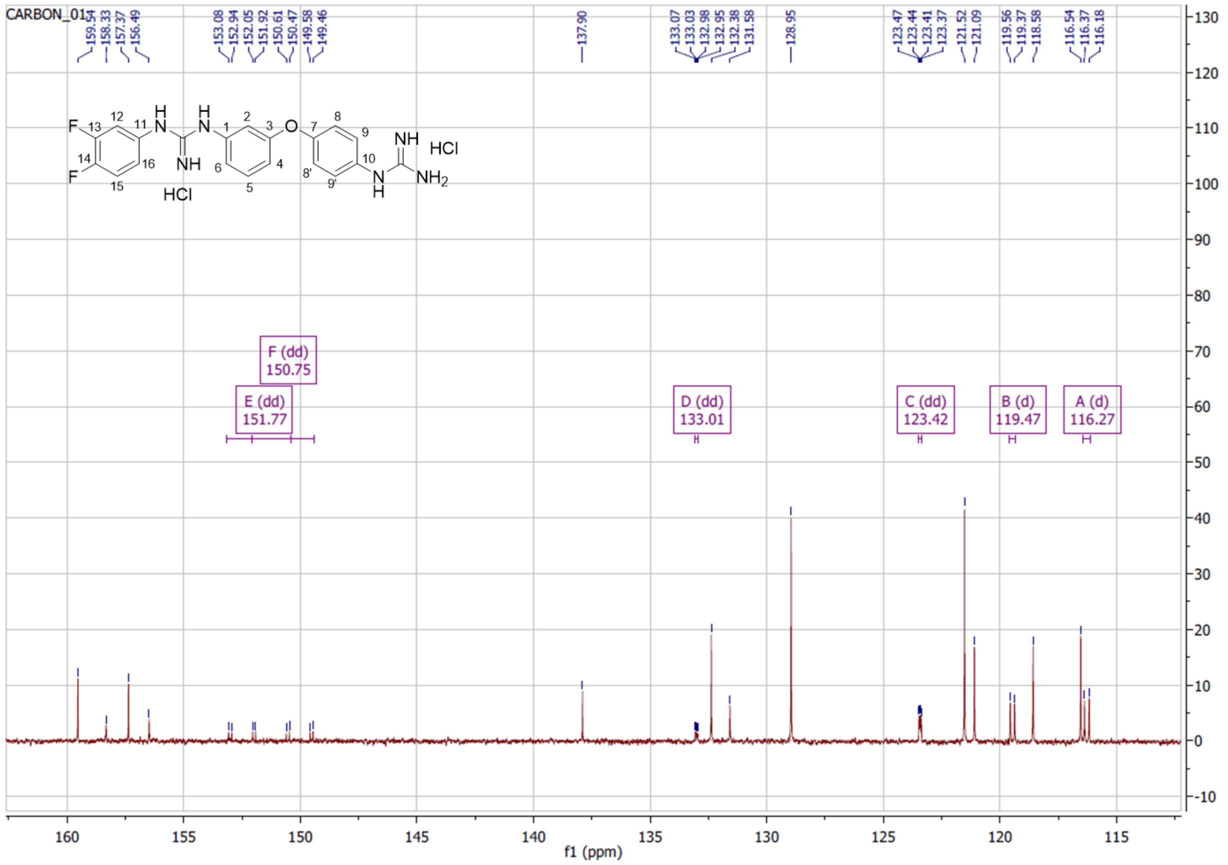

CARBON_01
159.54
158.33
157.37
156.49
153.08
152.94
152.05
151.92
150.61
150.47
149.58
149.46
137.90
133.07
133.03
132.98
132.95
132.38
131.58
128.95
123.47
123.44
123.41
123.37
121.52
121.09
119.56
119.37
118.58
116.54
116.37
116.18
HCl
HCl
F (dd)
150.75
E (dd)
151.77
D (dd)
133.01
C (dd)
123.42
B (d)
119.47
A (d)
116.27
f1 (ppm)

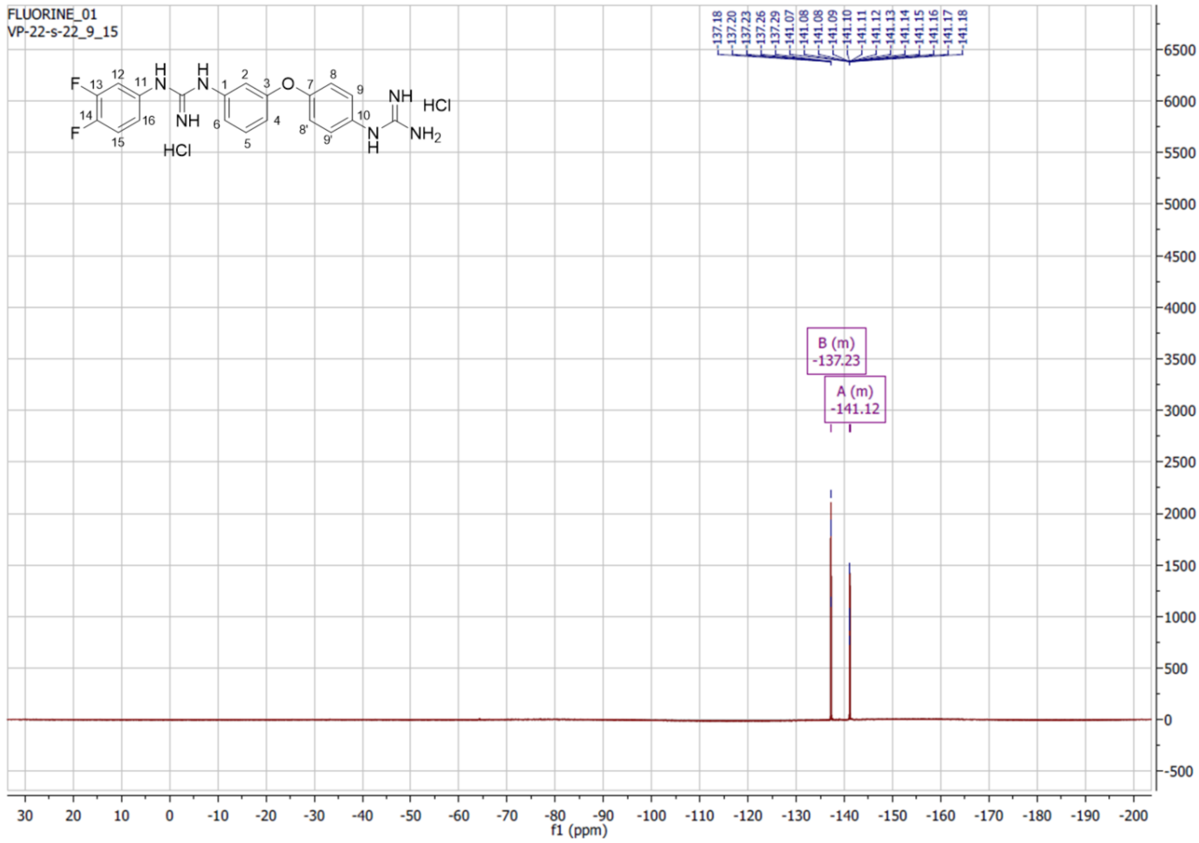
FLUORINE_01
VP-22-s-22_9_15
B (m)
-137.23
A (m)
-141.12
f1 (ppm)

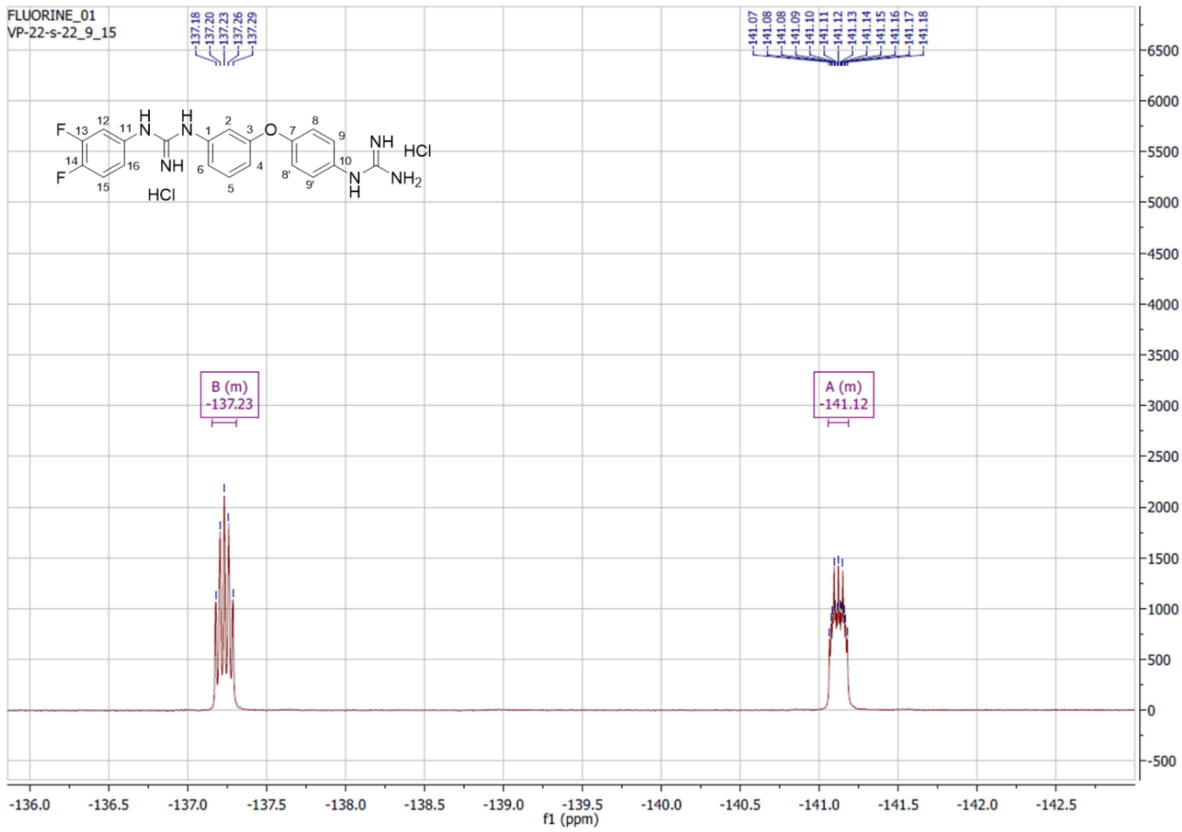
FLUORINE_01
VP-22-s-22_9_15
B (m)
-137.23
A (m)
-141.12
f1 (ppm)

^{1}H-NMR, ^{13}C-NMR, and ^{19}F-NMR for compound **7**

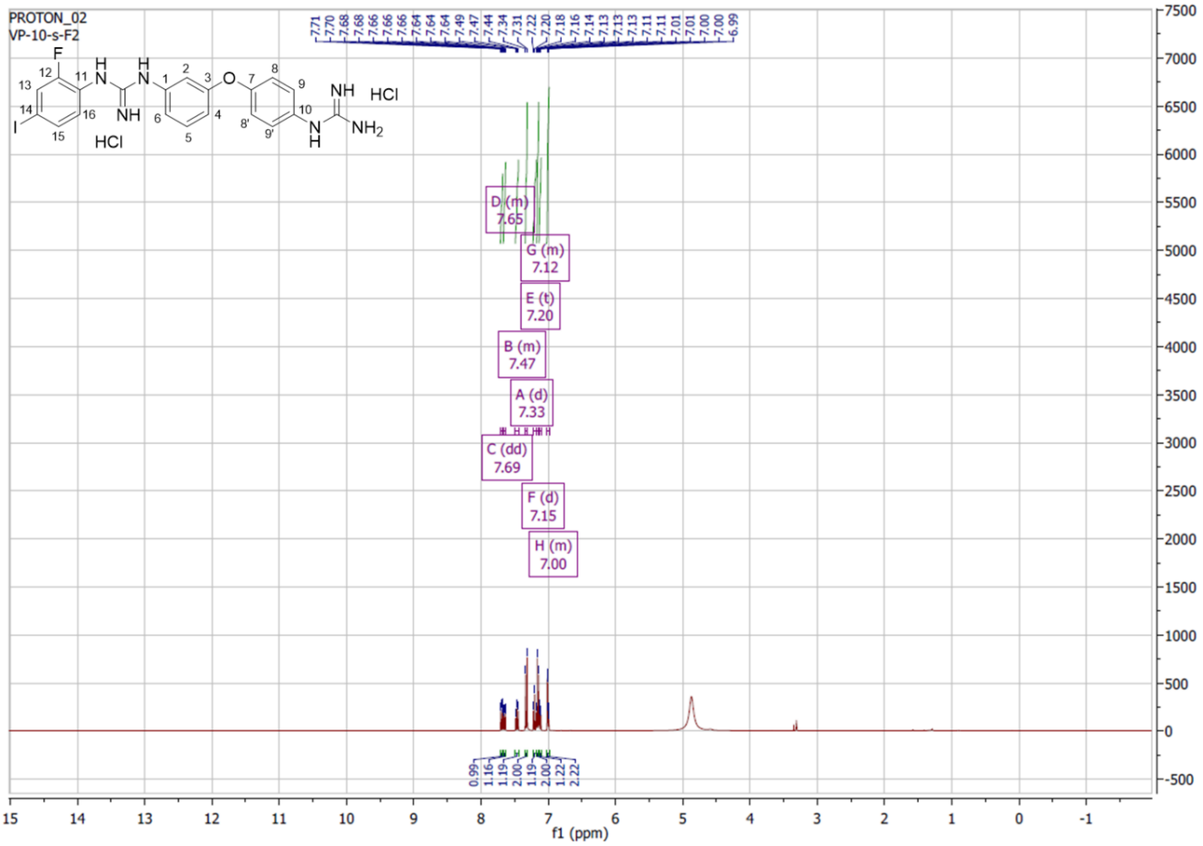

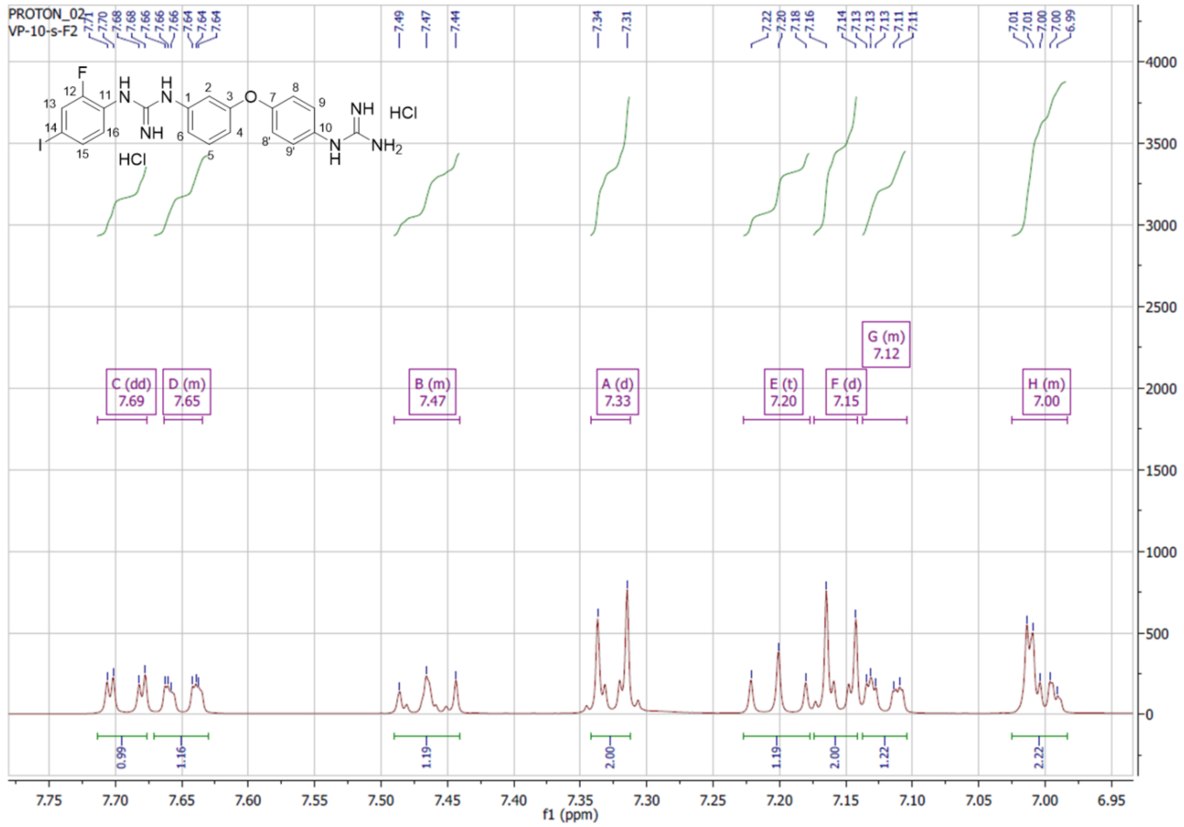

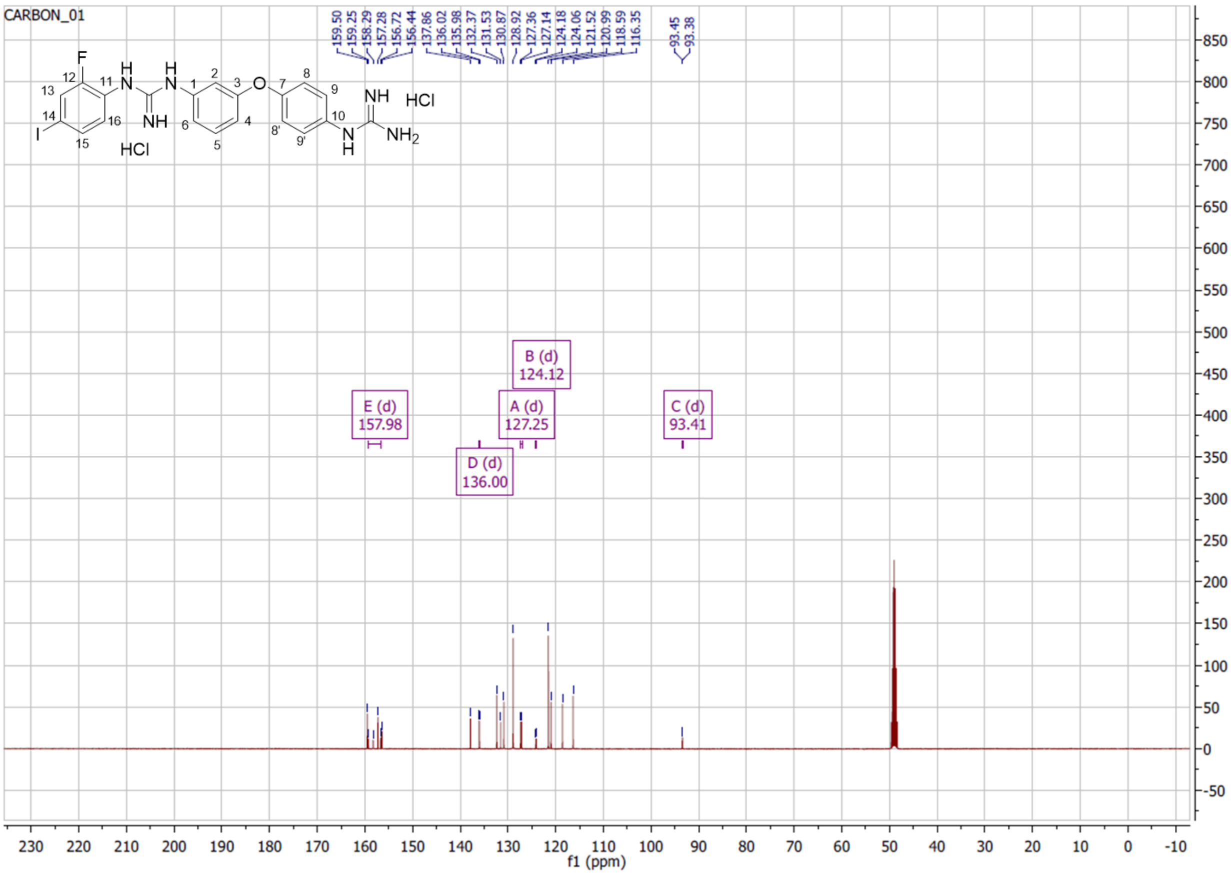
CARBON_01
159.50
159.25
158.29
157.28
156.72
156.44
137.86
136.02
135.98
132.37
131.53
130.87
128.92
127.36
127.14
124.18
124.06
121.52
120.99
118.59
116.35
93.45
93.38
E (d)
157.98
B (d)
124.12
A (d)
127.25
D (d)
136.00
C (d)
93.41
f1 (ppm)

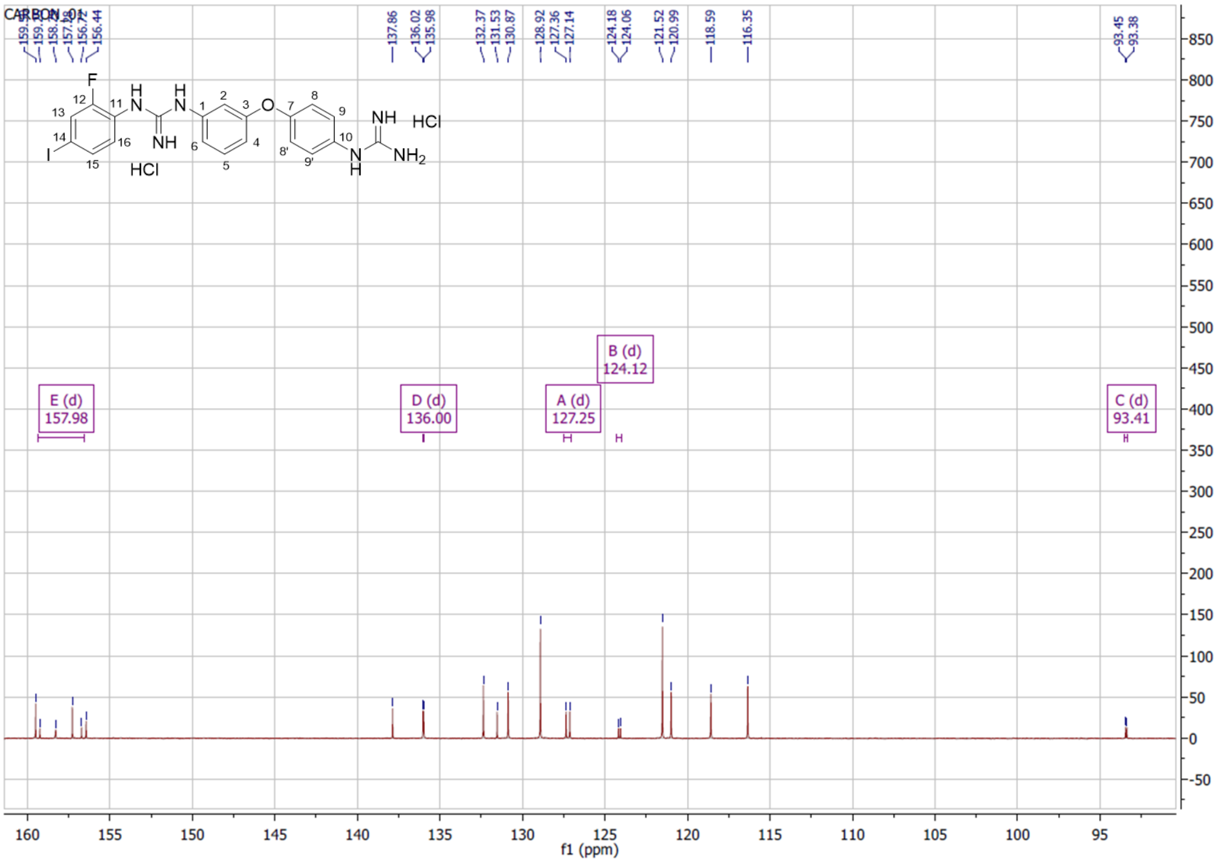
CARBON_01
137.86
136.02
135.98
132.37
131.53
130.87
128.92
127.36
127.14
124.18
124.06
121.52
120.99
118.59
116.35
93.45
93.38
E (d)
157.98
D (d)
136.00
A (d)
127.25
B (d)
124.12
C (d)
93.41
f1 (ppm)

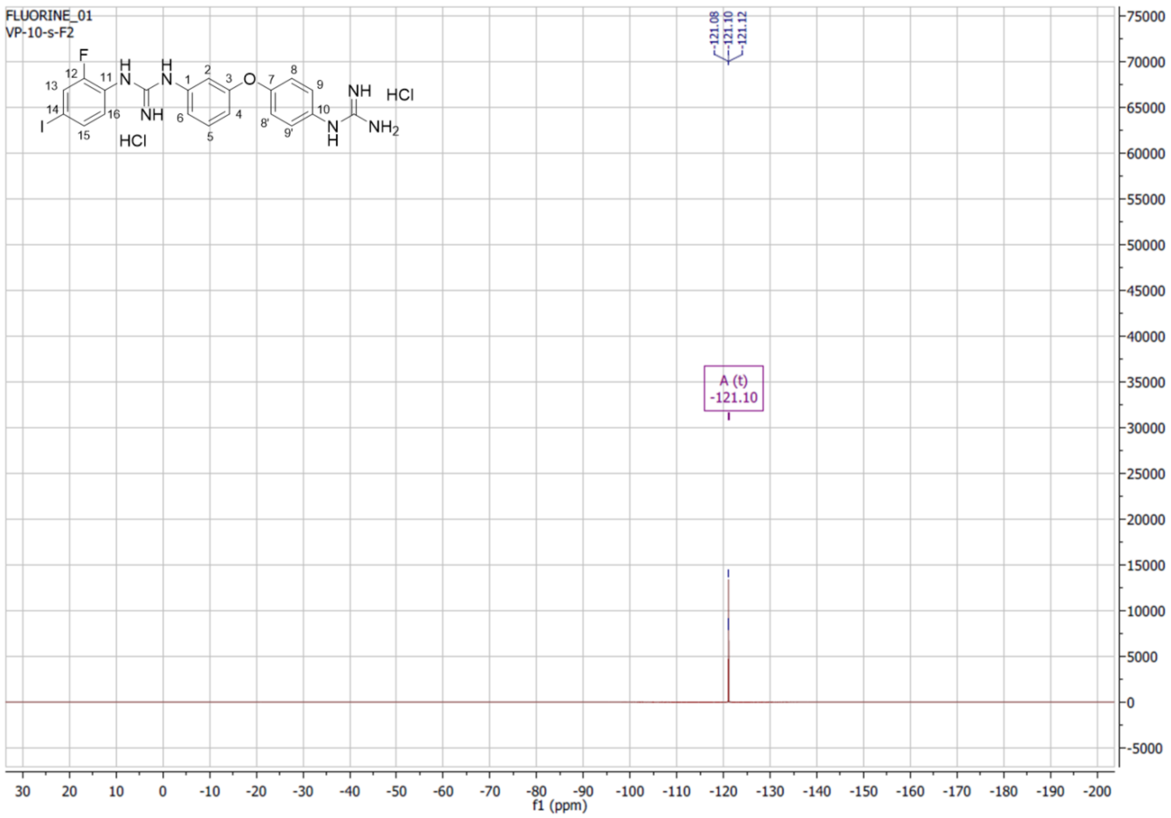
FLUORINE_01
VP-10-s-F2
HCl
HCl
-121.08
-121.10
-121.12
A (t)
-121.10
f1 (ppm)

^{1}H-NMR and ^{13}C-NMR for compound **8**

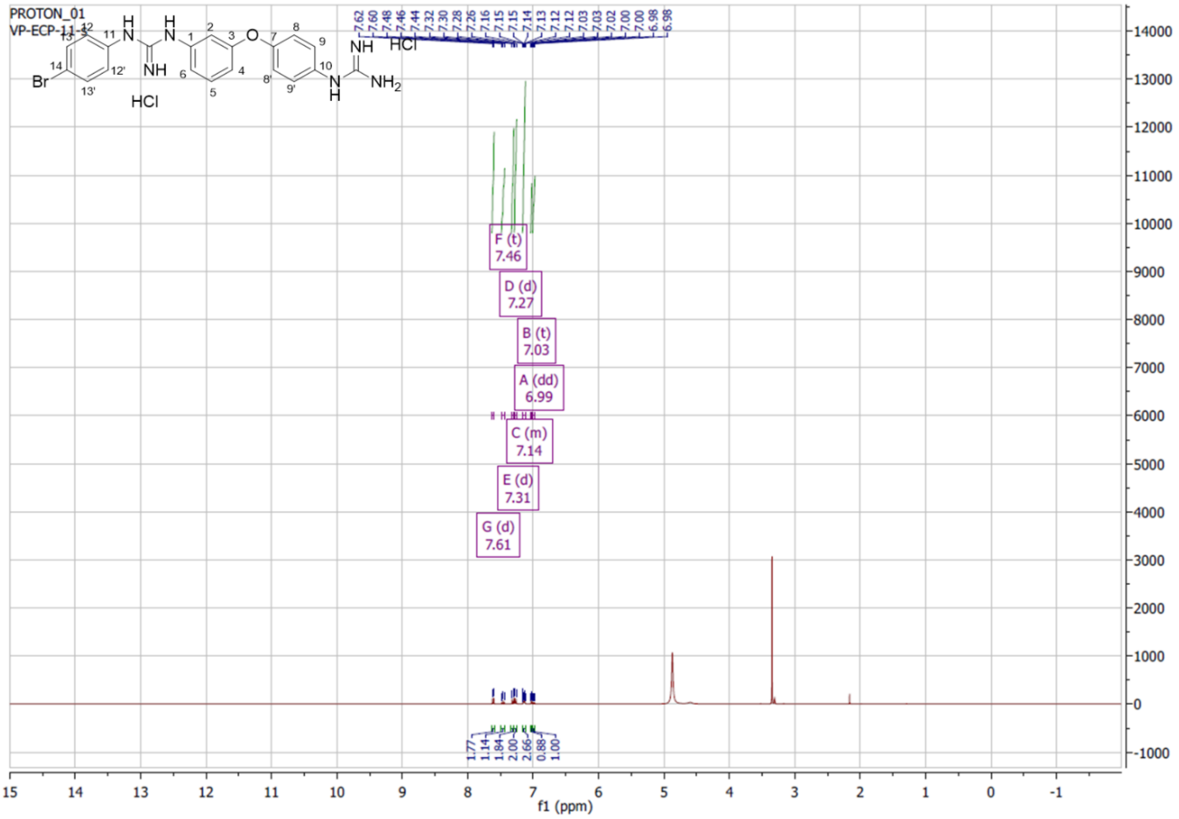

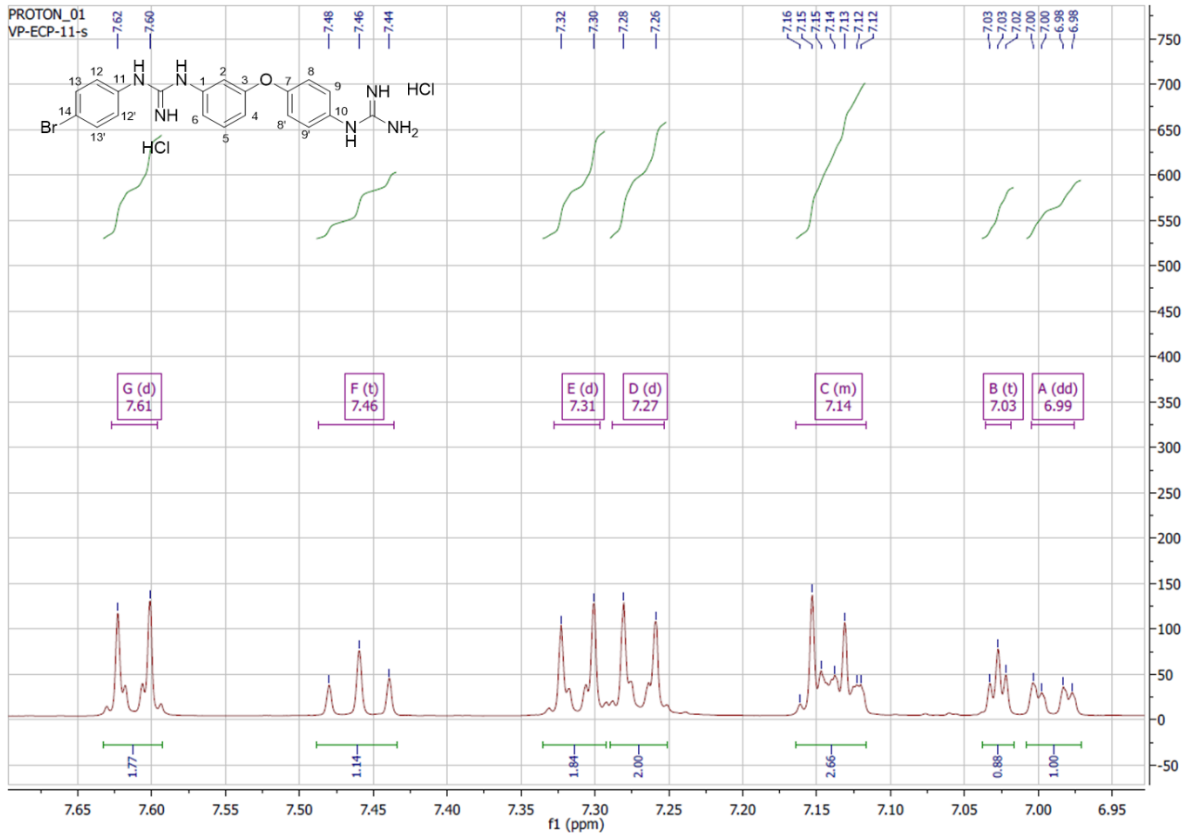

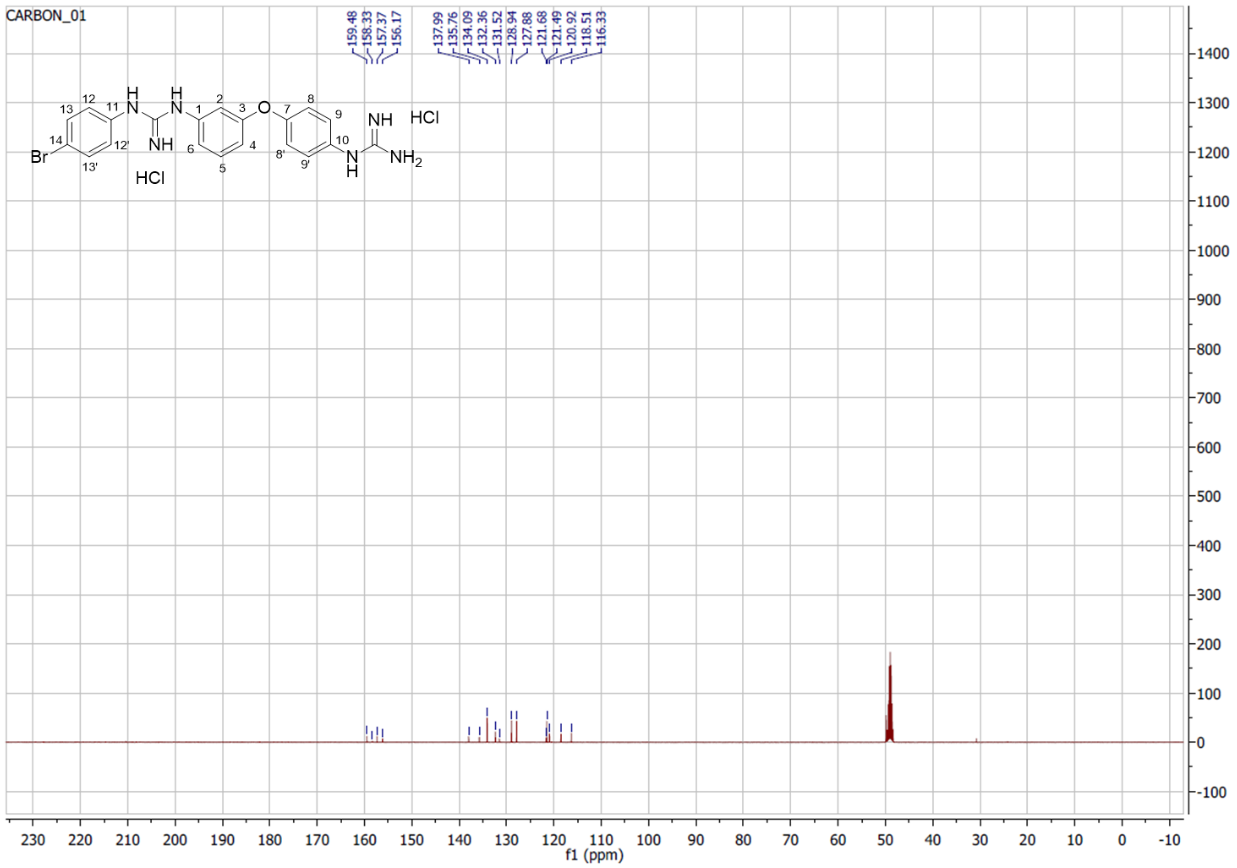
CARBON_01
159.48
158.33
157.37
156.17
137.99
135.76
134.09
132.36
131.52
128.94
127.88
121.68
121.49
120.92
118.51
116.33
HCl
HCl
f1 (ppm)

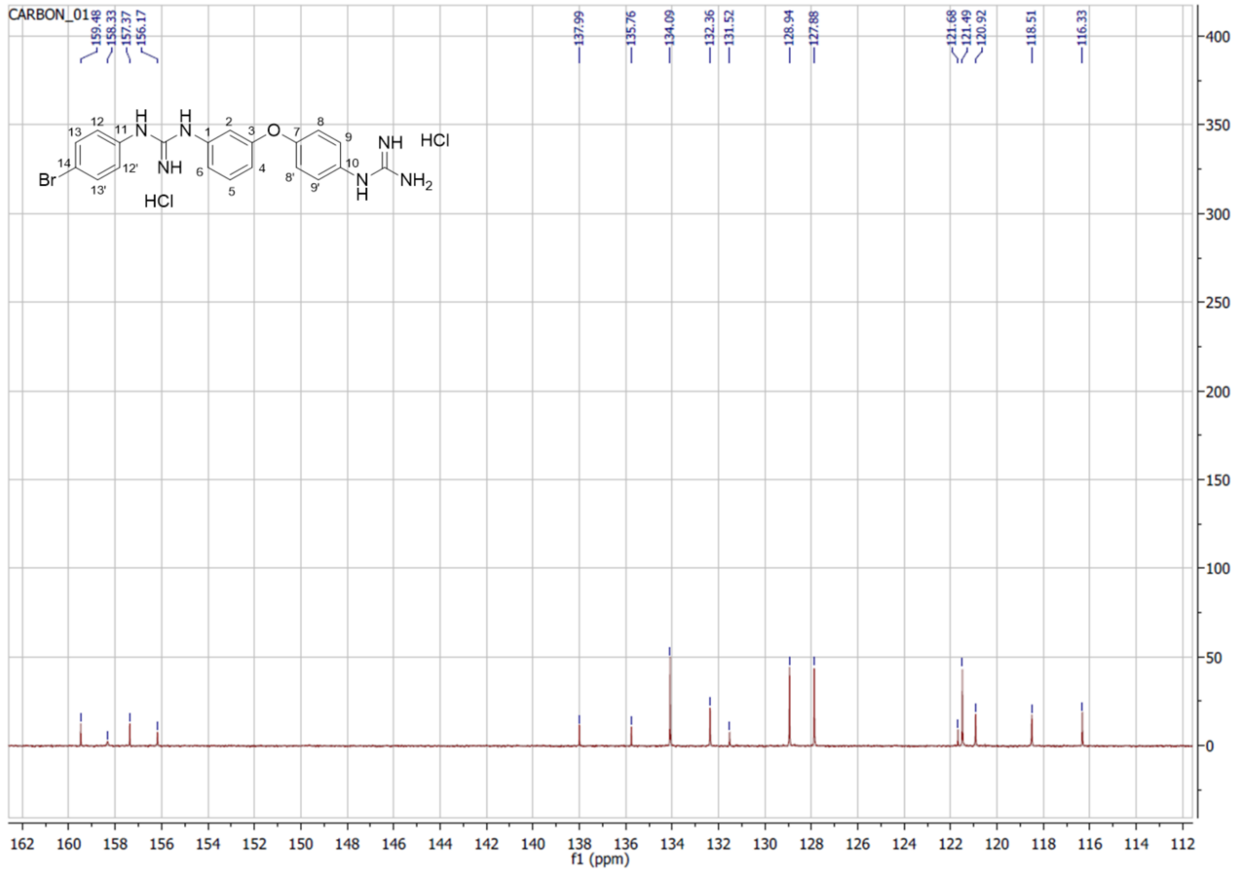
CARBON_01
159.48
158.33
157.37
156.17
137.99
135.76
134.09
132.36
131.52
128.94
127.88
121.68
121.49
120.92
118.51
116.33
HCl
HCl
f1 (ppm)

^{1}H-NMR, ^{13}C-NMR, and ^{19}F-NMR for compound **9**

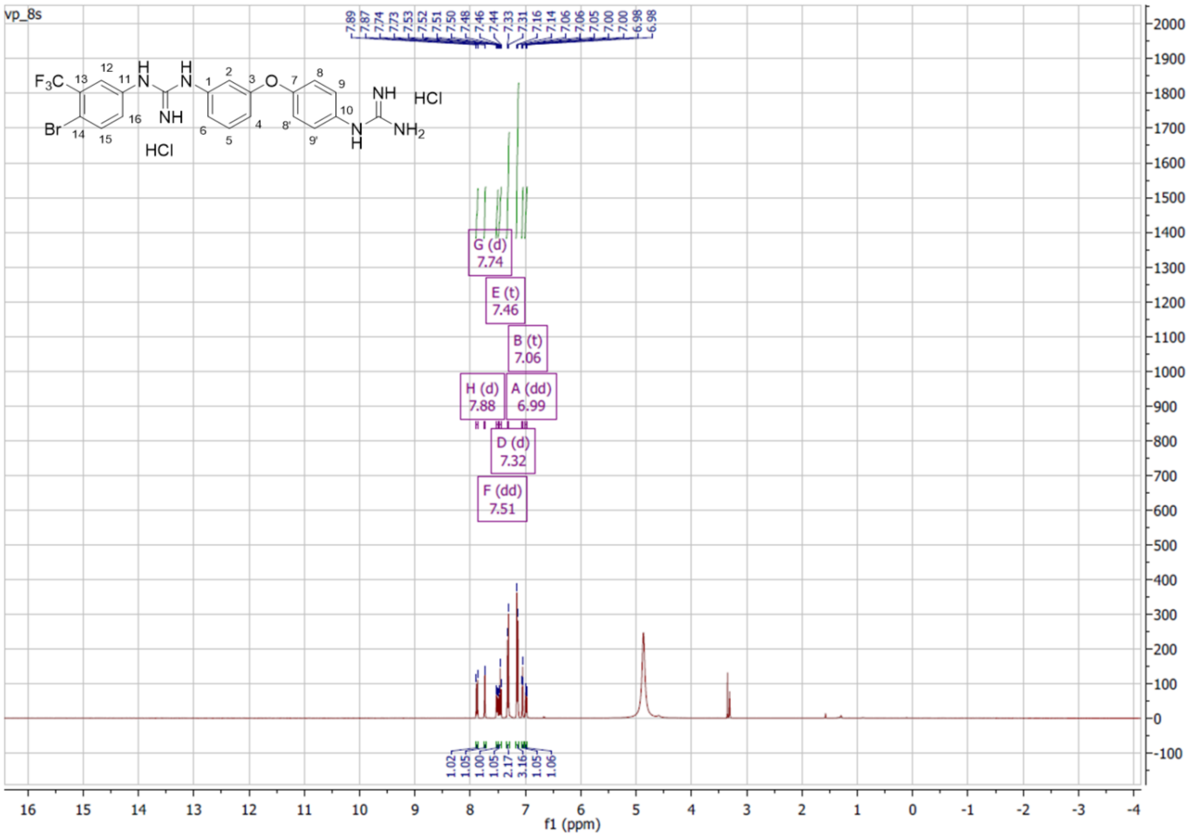

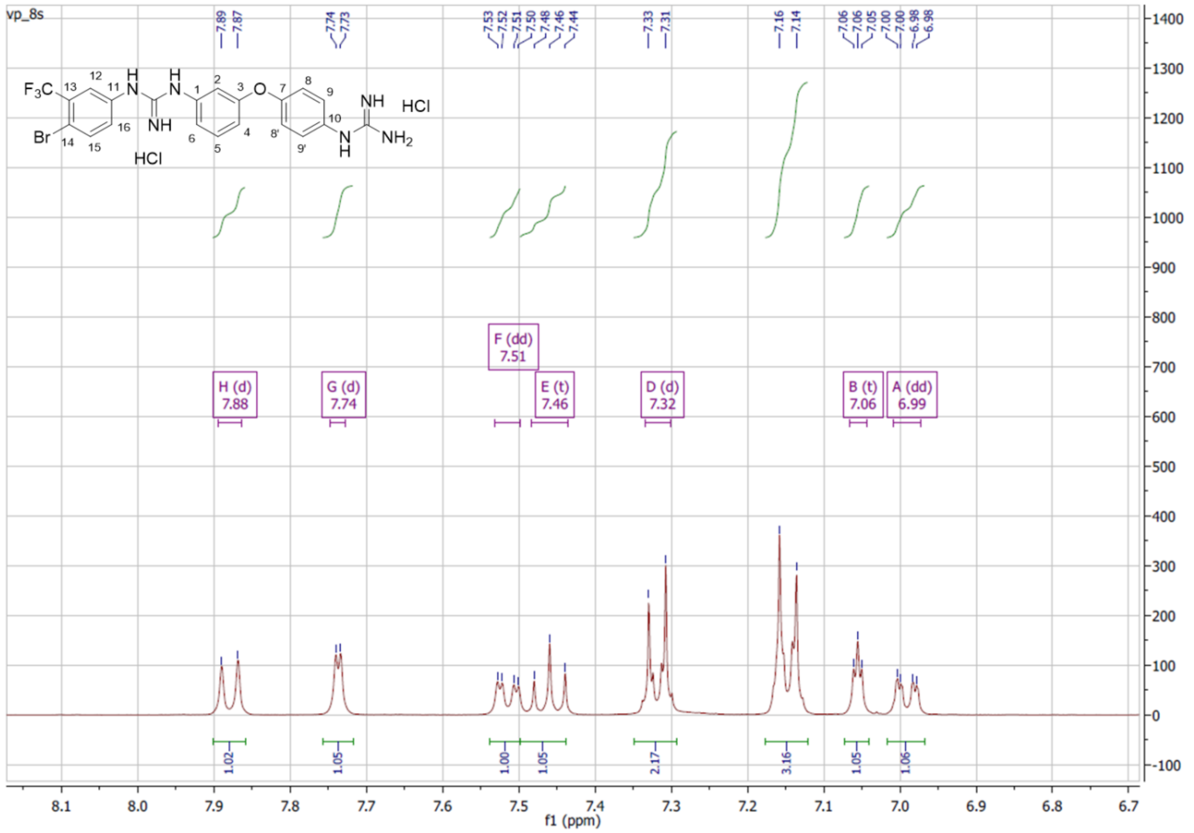

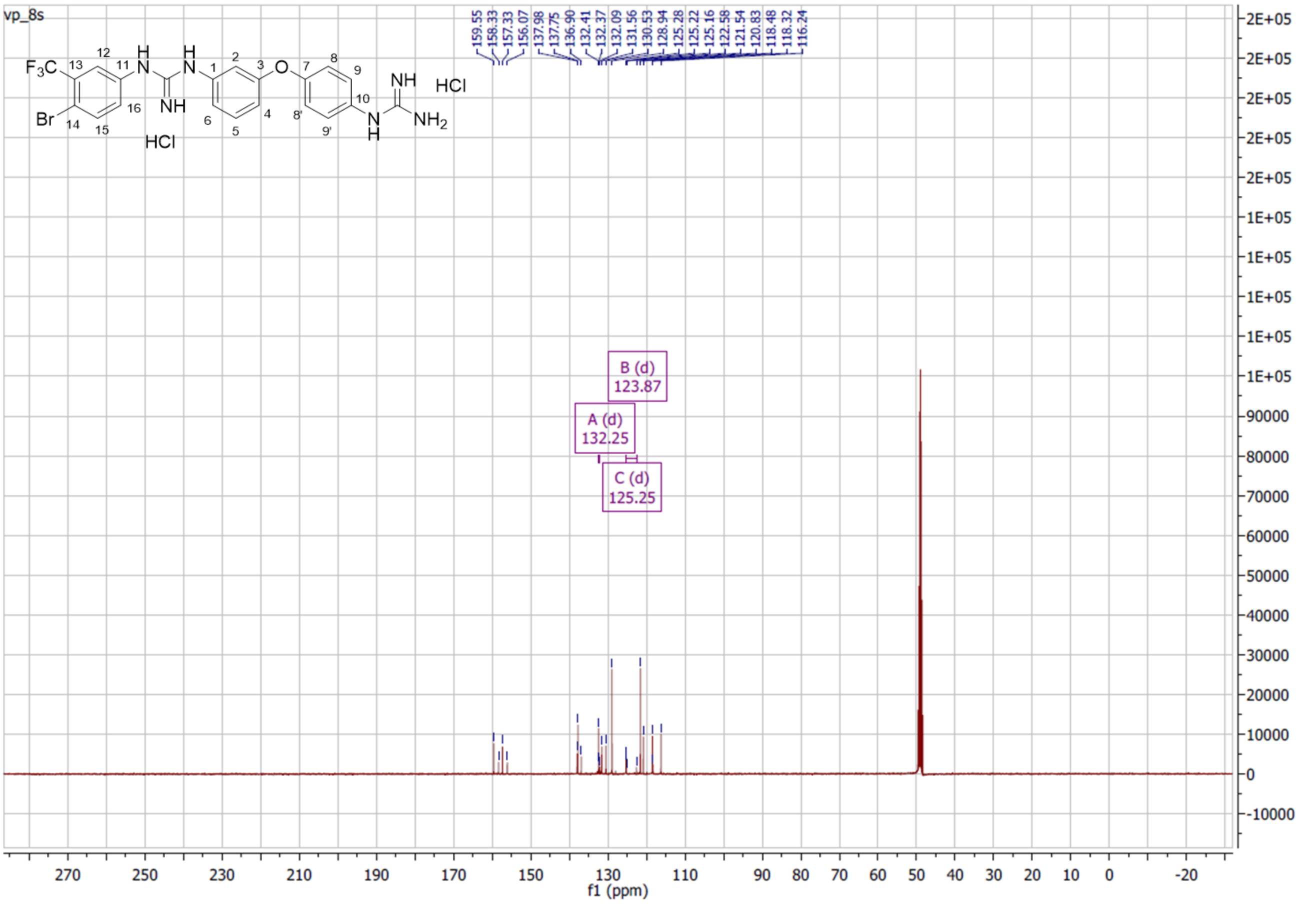
vp_8s
159.55
158.33
157.33
156.07
137.98
137.75
136.90
132.41
132.37
132.09
131.56
130.53
128.94
125.28
125.22
125.16
122.58
121.54
120.83
118.48
118.32
116.24
B (d)
123.87
A (d)
132.25
C (d)
125.25
f1 (ppm)

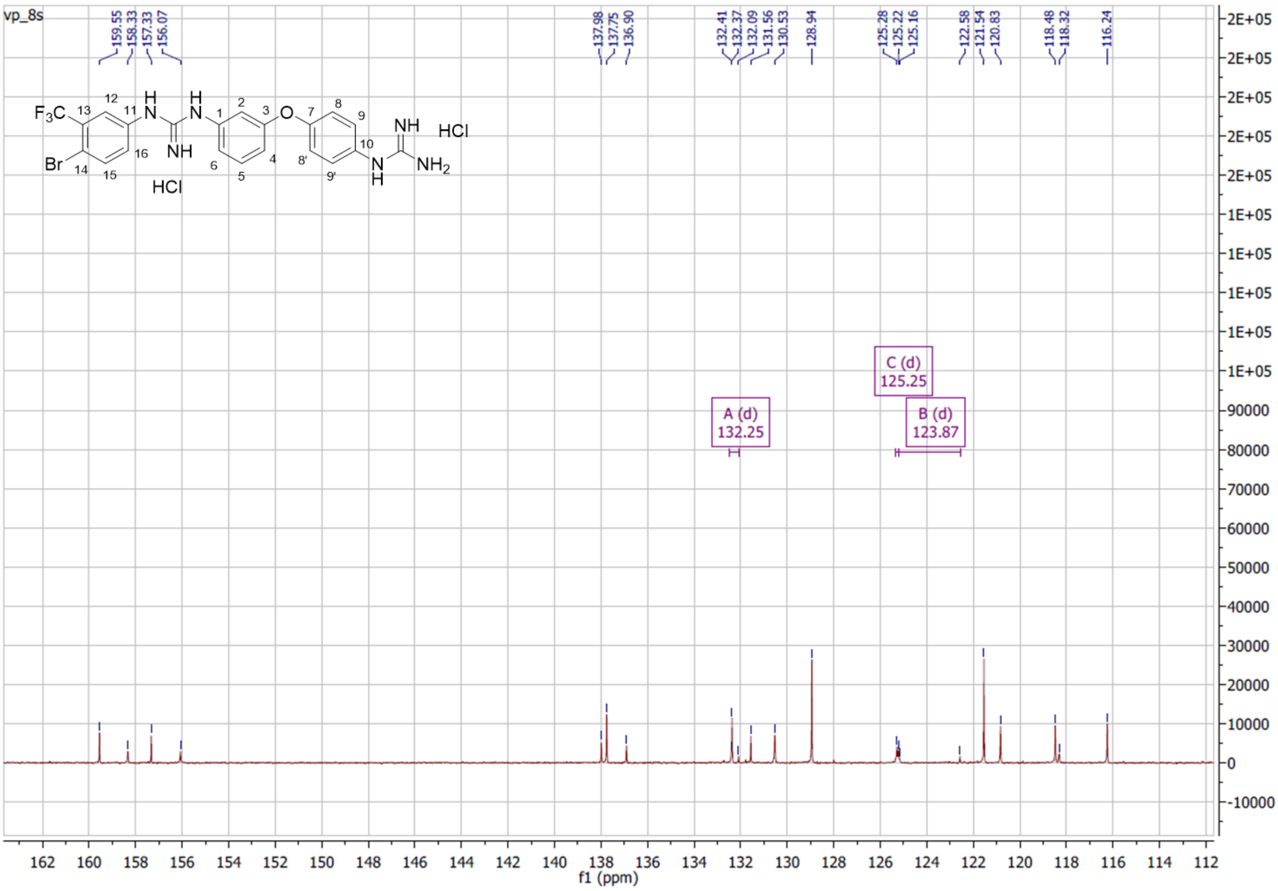
vp_8s
159.55
158.33
157.33
156.07
137.98
137.75
136.90
132.41
132.37
132.09
131.56
130.53
128.94
125.28
125.22
125.16
122.58
121.54
120.83
118.48
118.32
116.24
C (d)
125.25
A (d)
132.25
B (d)
123.87
f1 (ppm)

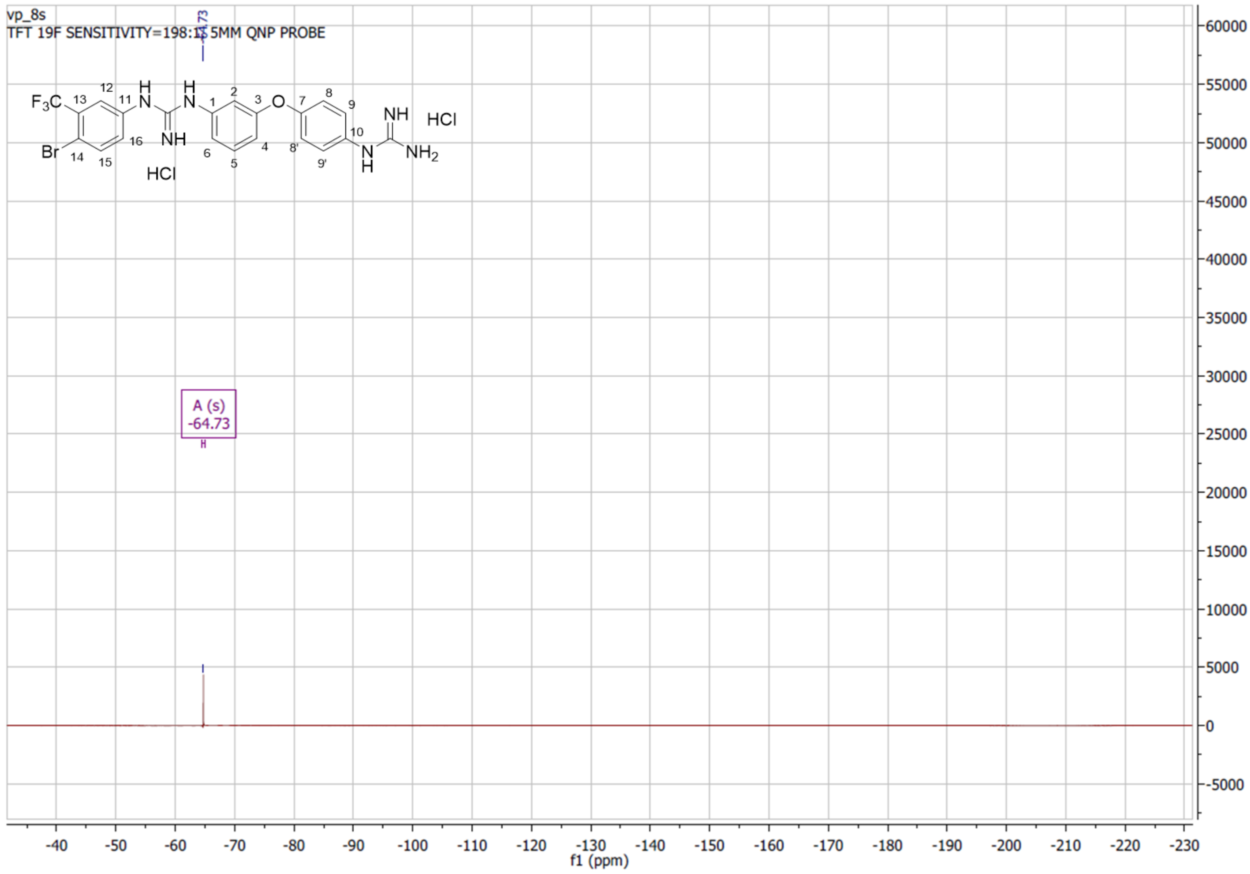
vp_8s
TFT 19F SENSITIVITY=198:1 5MM QNP PROBE
-64.73
A (s)
-64.73
HCl
NH
NH$_2$
F$_3$C
Br
f1 (ppm)

^{1}H-NMR, ^{13}C-NMR, and ^{19}F-NMR for compound **10**

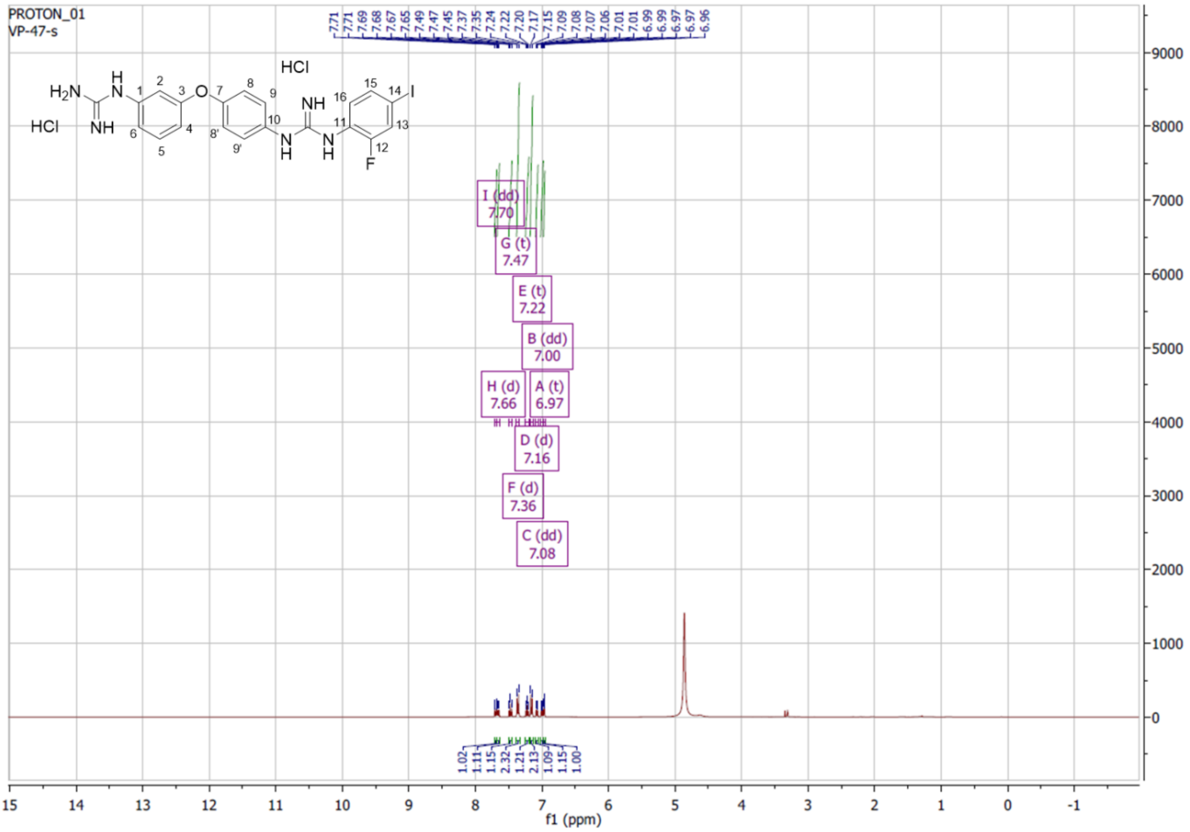

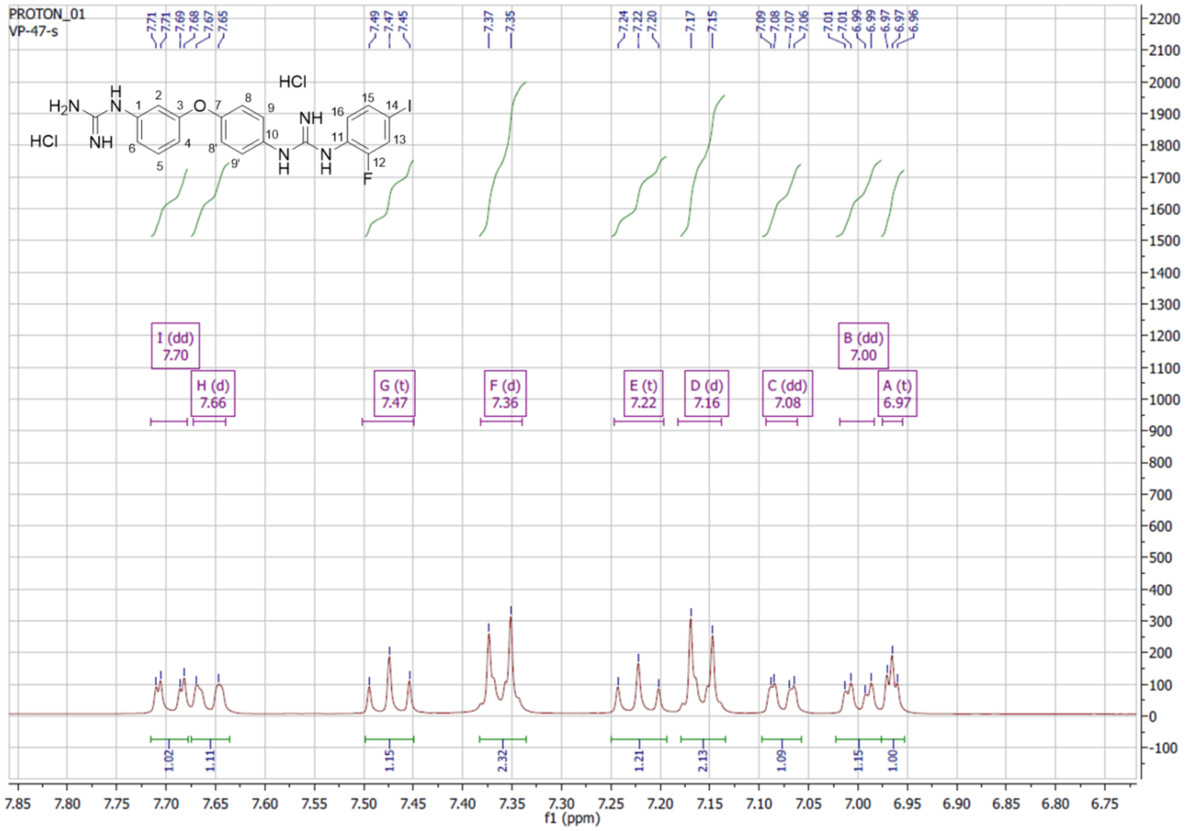

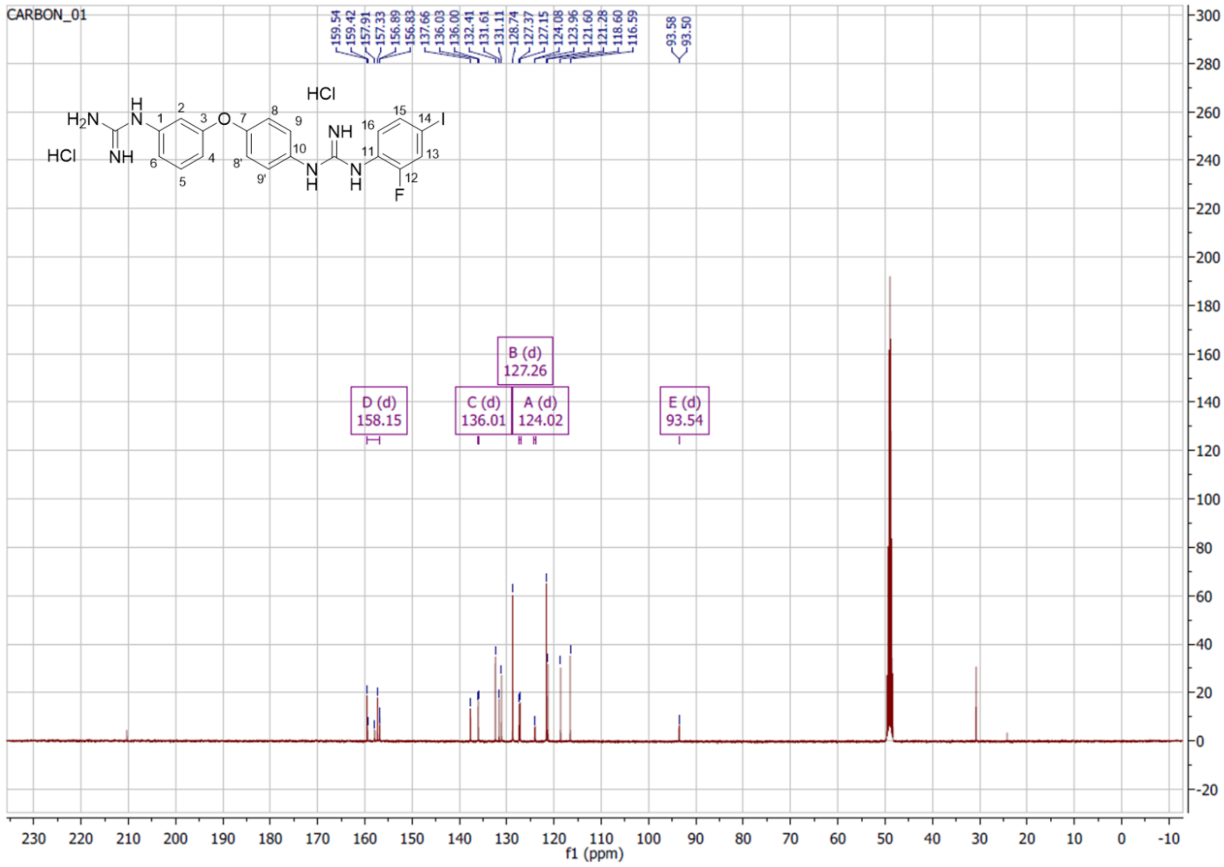
CARBON_01
159.54 159.42 157.91 157.33 156.89 156.83 137.66 136.03 136.00 132.41 131.61 131.11 128.74 127.37 127.15 124.08 123.96 121.60 121.28 118.60 116.59 93.58 93.50
HCl
HCl
D (d) 158.15
C (d) 136.01
B (d) 127.26
A (d) 124.02
E (d) 93.54
f1 (ppm)

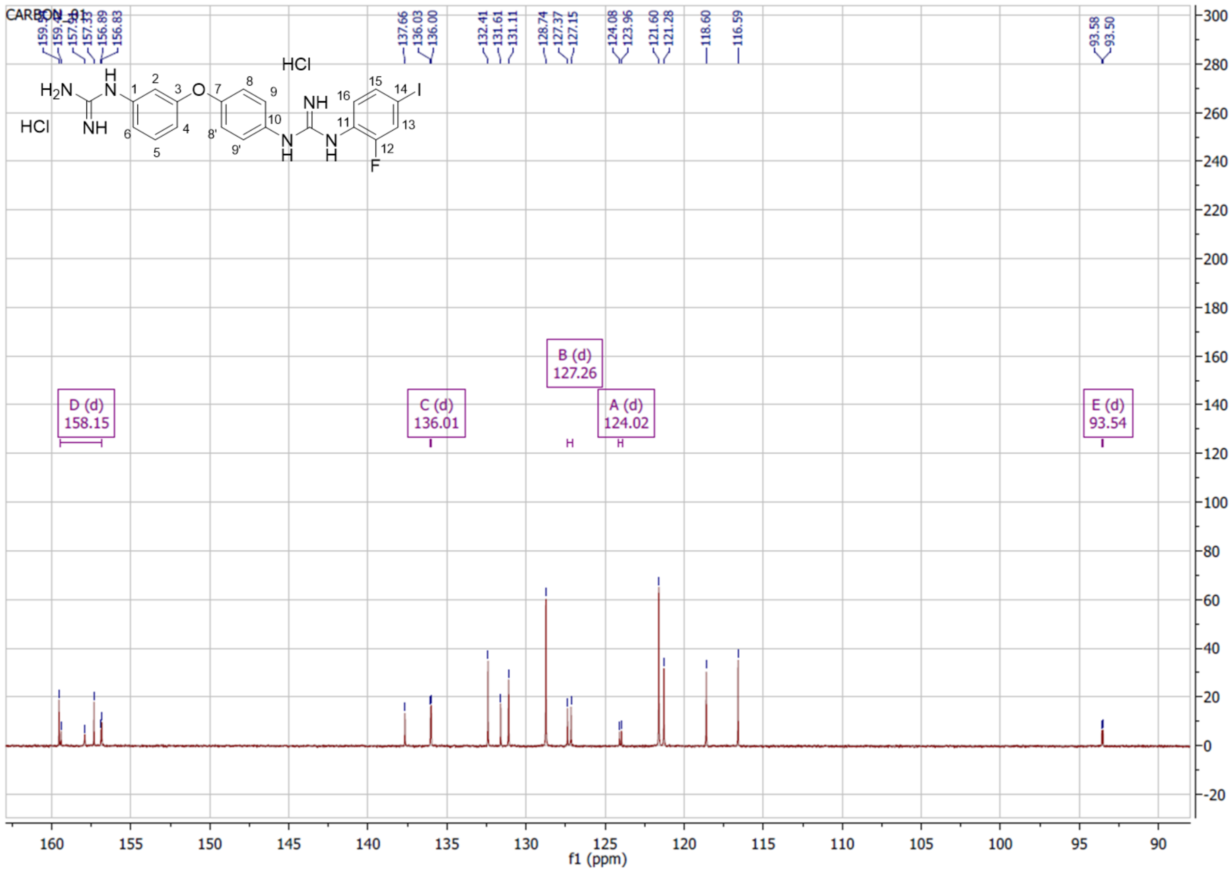
CARBON_01
159.54 159.42 157.91 157.33 156.89 156.83 137.66 136.03 136.00 132.41 131.61 131.11 128.74 127.37 127.15 124.08 123.96 121.60 121.28 118.60 116.59 93.58 93.50
HCl
HCl
D (d) 158.15
C (d) 136.01
B (d) 127.26
A (d) 124.02
E (d) 93.54
f1 (ppm)

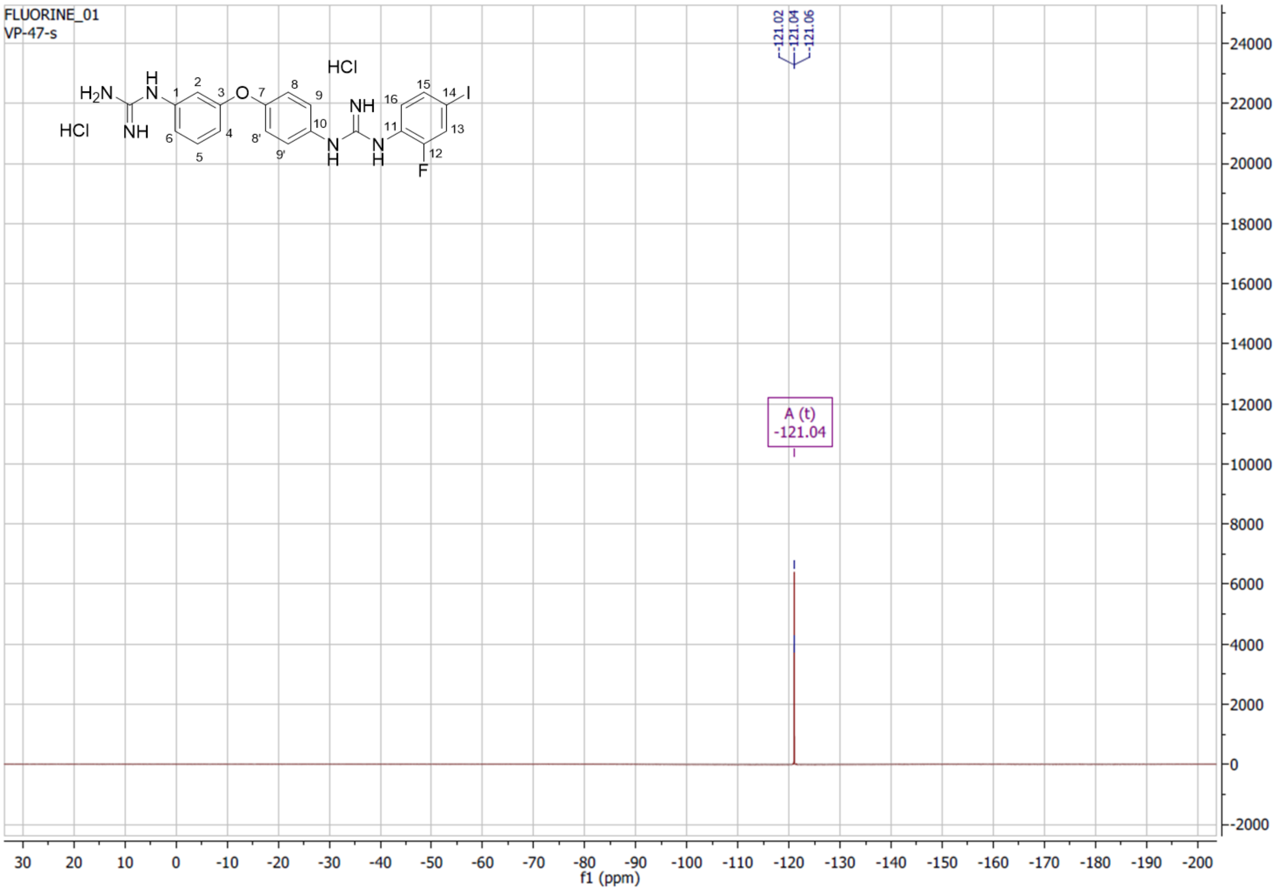
FLUORINE_01
VP-47-s
HCl
HCl
A (t)
-121.04
-121.02
-121.04
-121.06
f1 (ppm)

^{1}H-NMR, ^{13}C-NMR, and ^{19}F-NMR for compound **11**

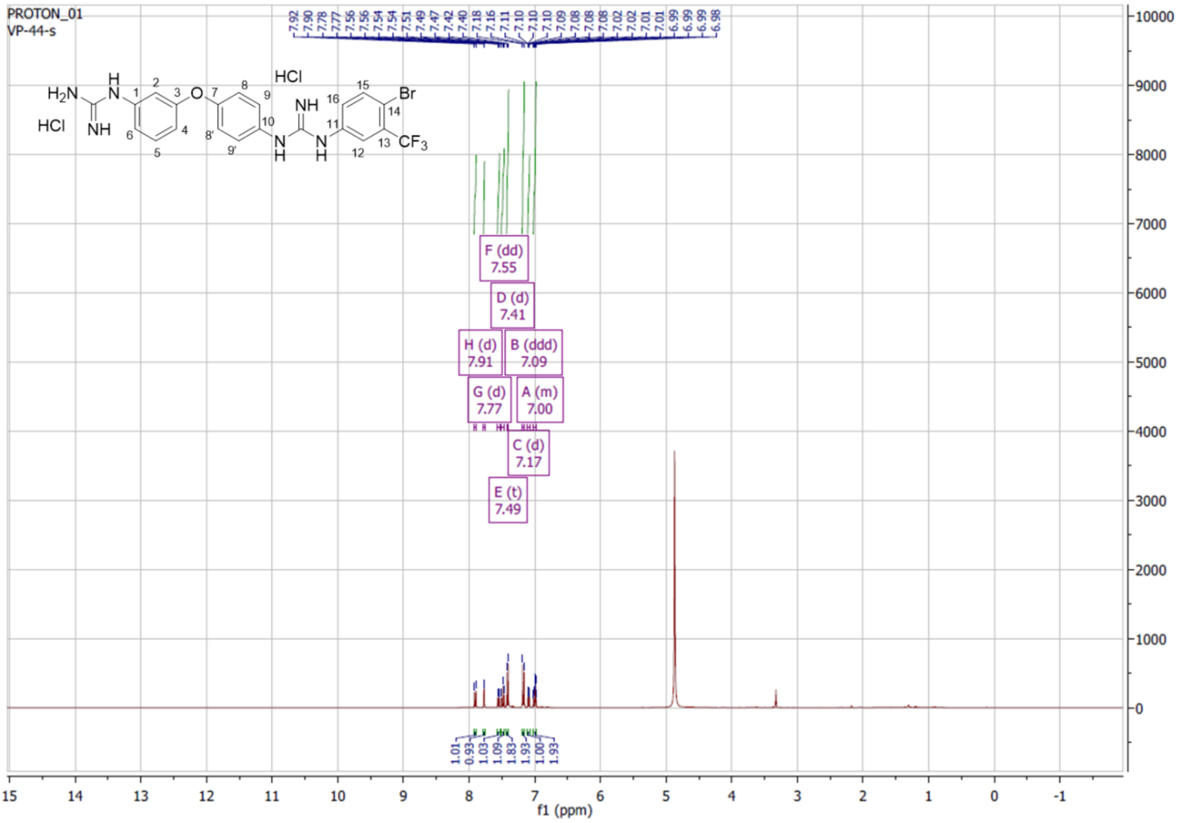

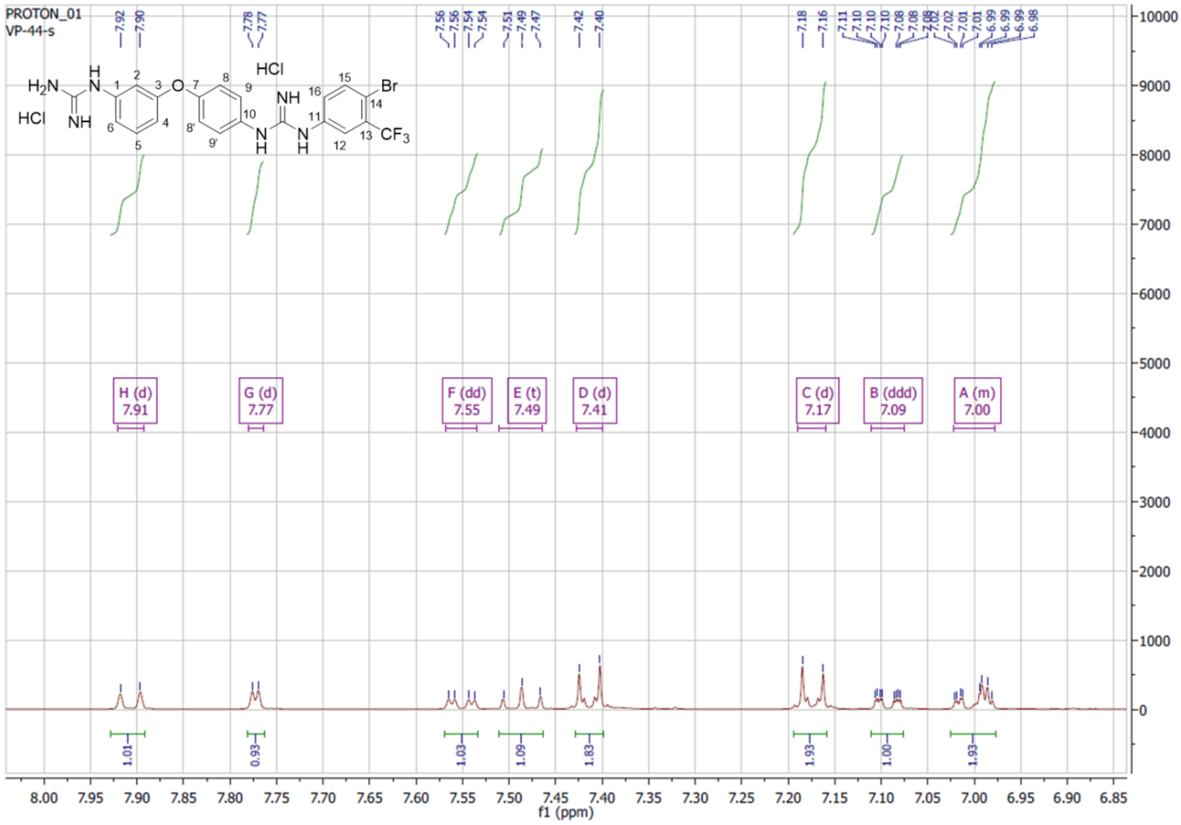

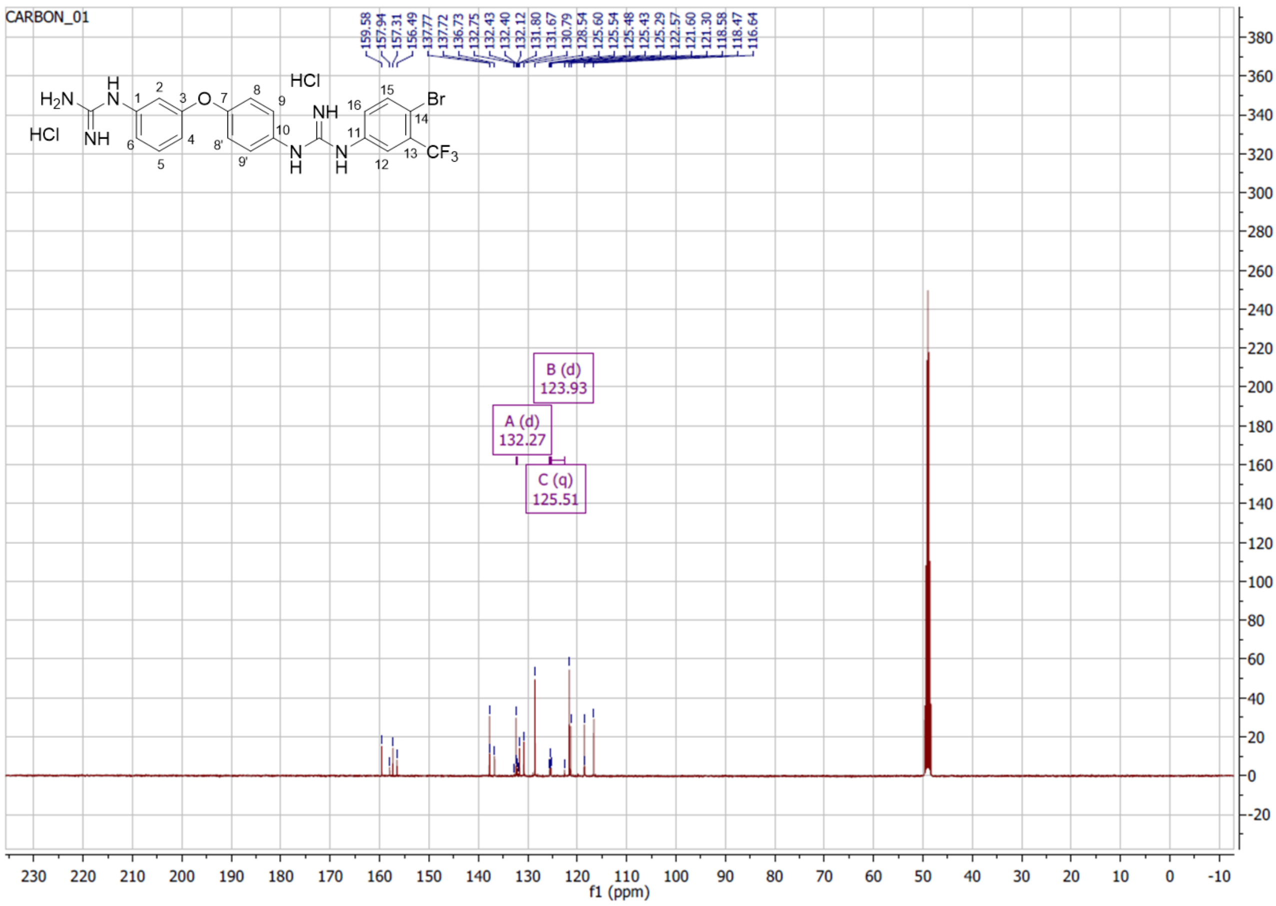
CARBON_01
159.58
157.94
157.31
156.49
137.77
137.72
136.73
132.75
132.43
132.40
132.12
131.80
131.67
130.79
128.54
125.60
125.54
125.48
125.43
125.29
122.57
121.60
121.30
118.58
118.47
116.64
HCl
HCl
H_2N
Br
CF_3
B (d) 123.93
A (d) 132.27
C (q) 125.51
f1 (ppm)

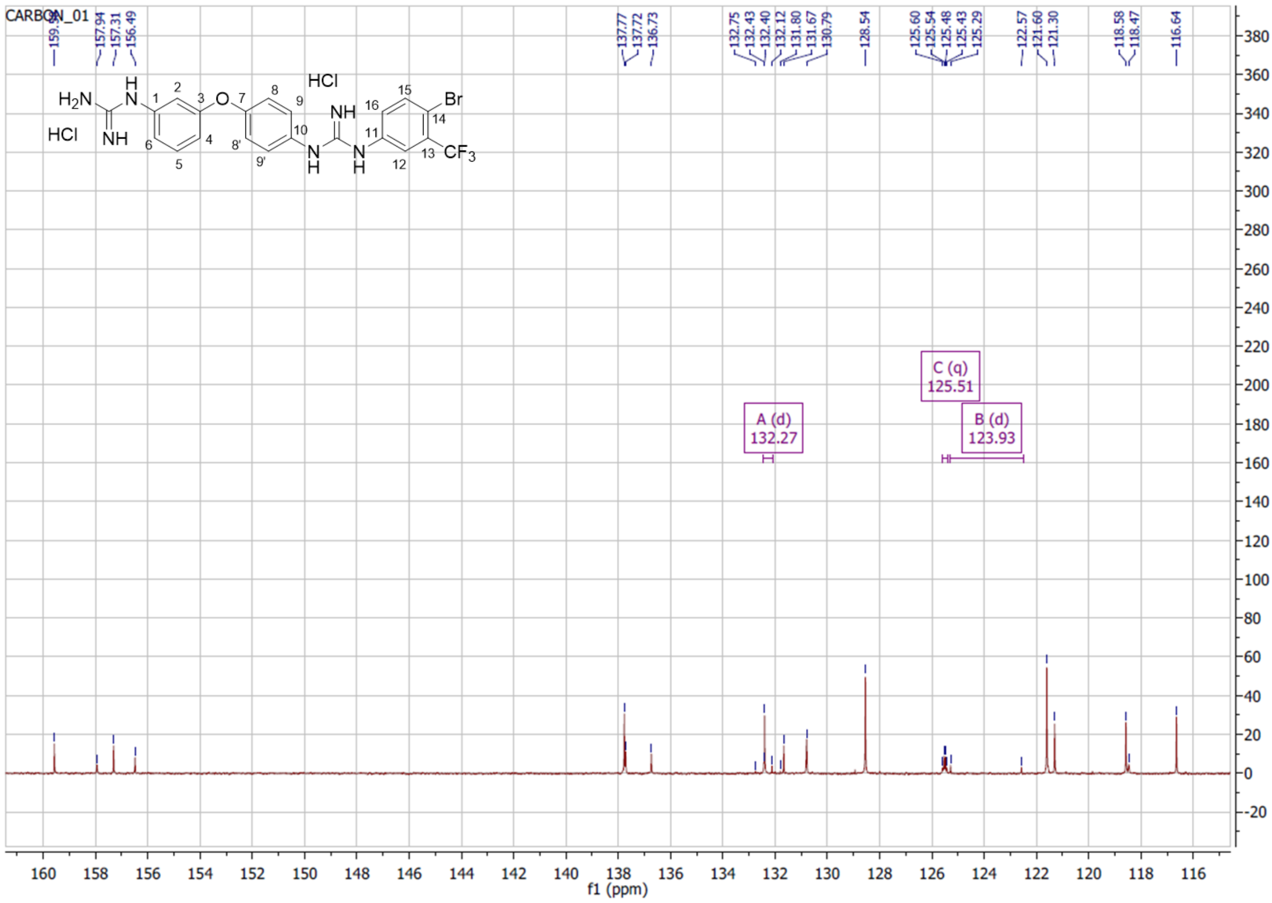
CARBON_01
159.58
157.94
157.31
156.49
137.77
137.72
136.73
132.75
132.43
132.40
132.12
131.80
131.67
130.79
128.54
125.60
125.54
125.48
125.43
125.29
122.57
121.60
121.30
118.58
118.47
116.64
HCl
HCl
H_2N
Br
CF_3
C (q) 125.51
A (d) 132.27
B (d) 123.93
f1 (ppm)

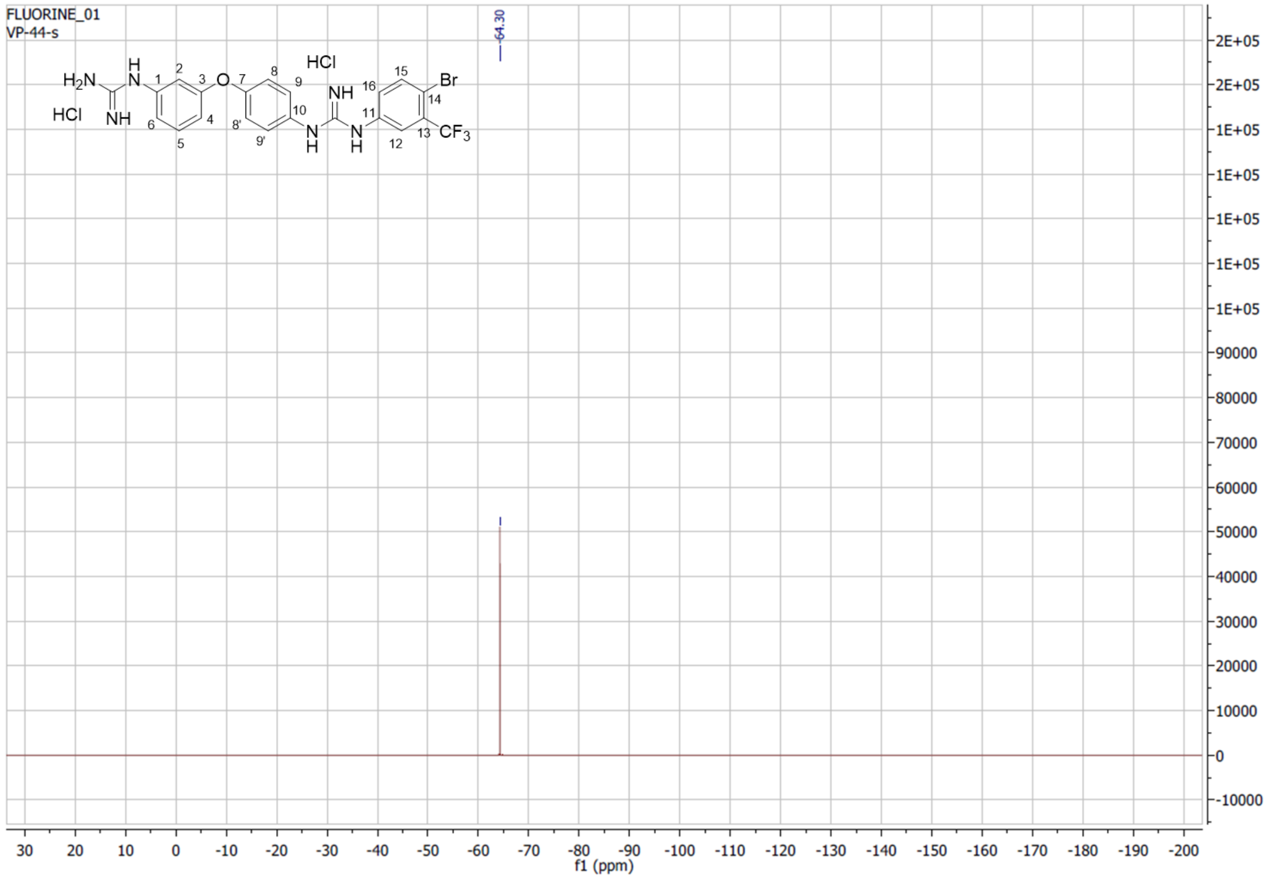

FLUORINE_01
VP-44-s
-64.30
H2N
HCl
NH
HCl
Br
CF3
f1 (ppm)

^{1}H-NMR and ^{13}C-NMR for compound **28**

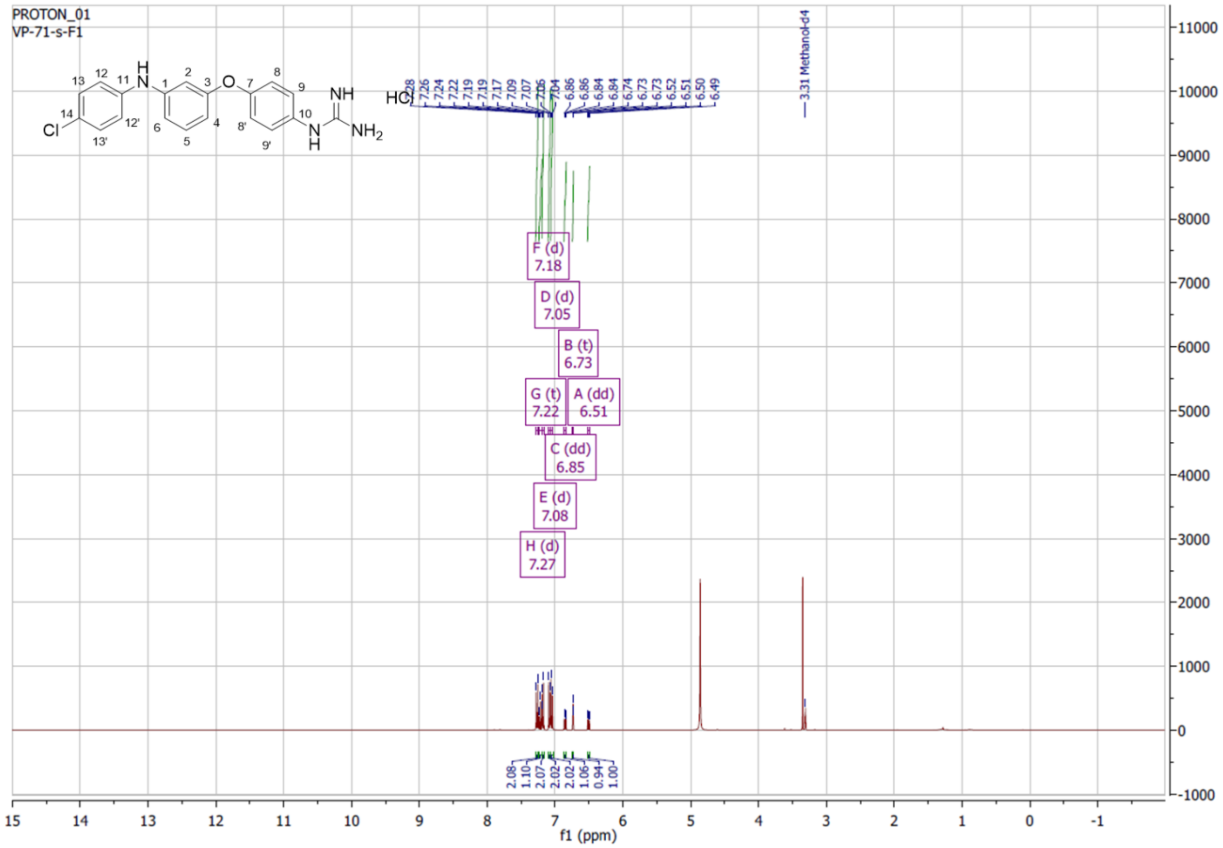

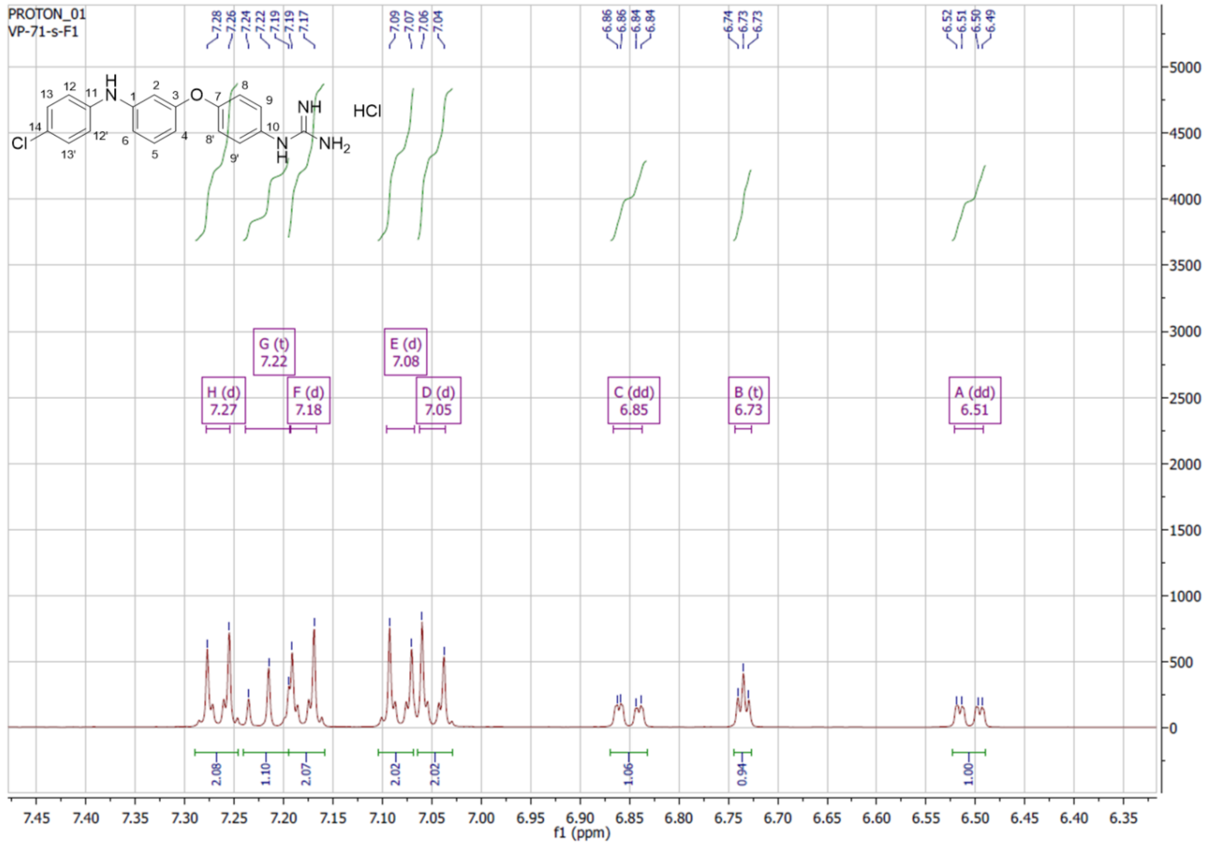

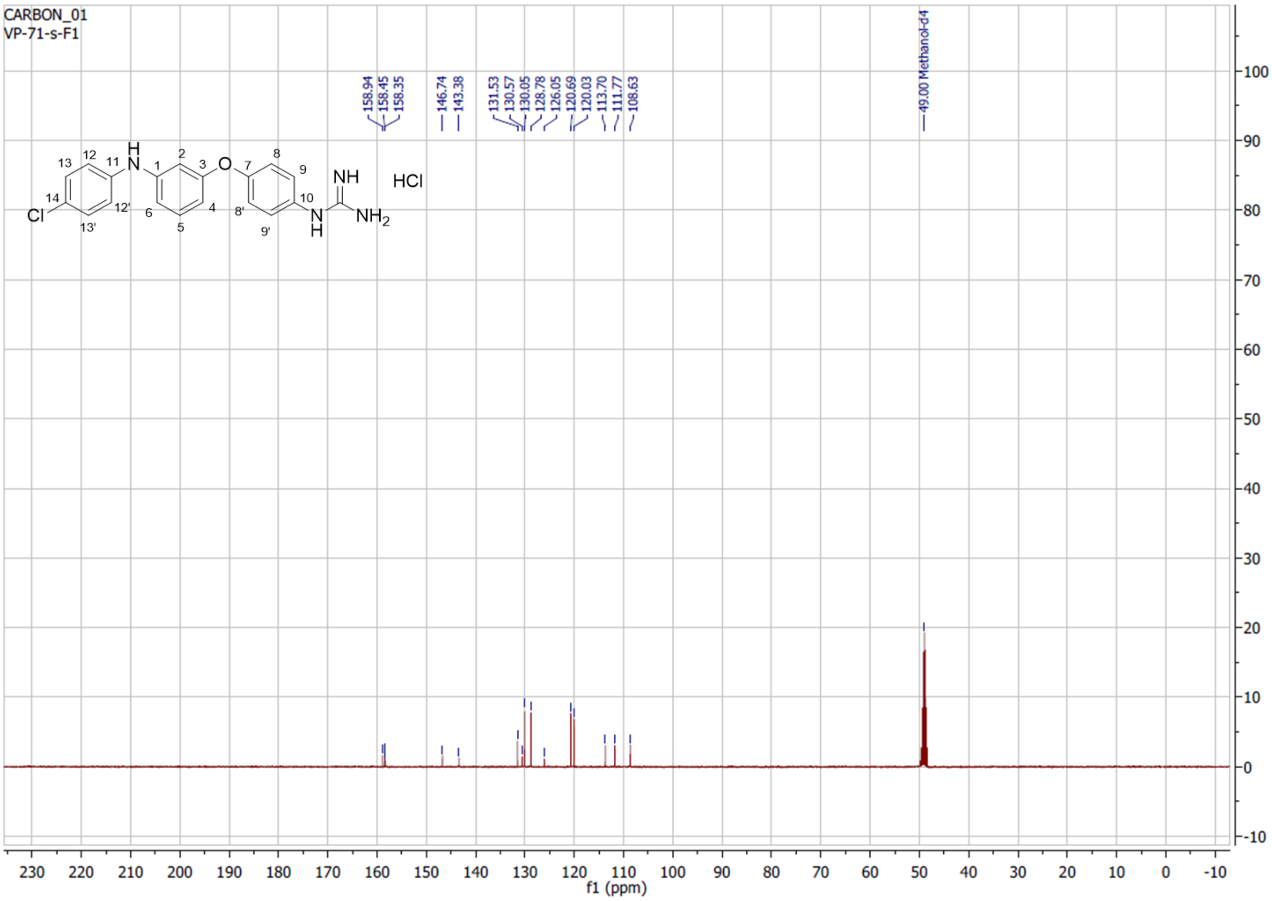
CARBON_01
VP-71-s-F1
158.94
158.45
158.35
146.74
143.38
131.53
130.57
130.05
128.78
126.05
120.69
120.03
113.70
111.77
108.63
49.00 Methanol-d4
HCl
f1 (ppm)

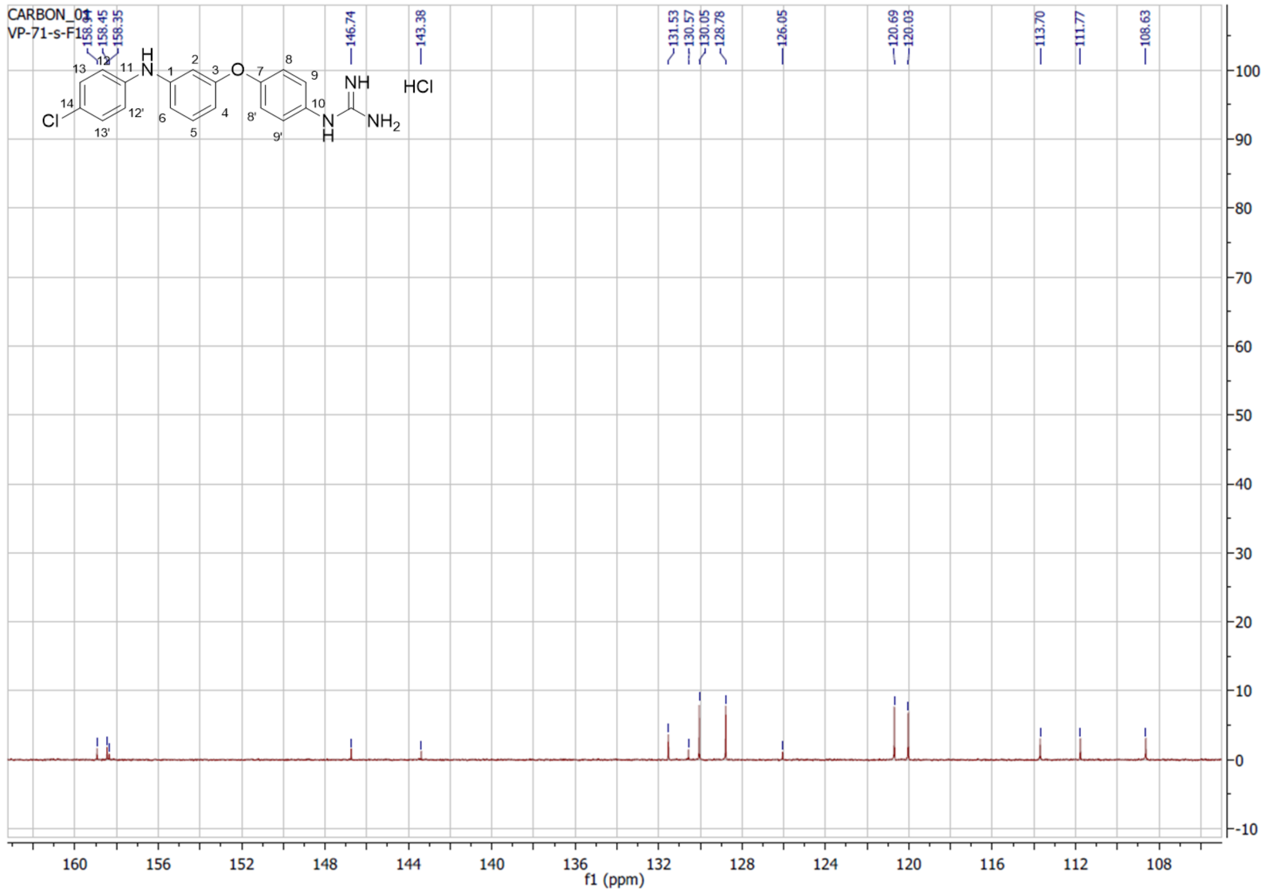
CARBON_01
VP-71-s-F1
158.94
158.45
158.35
146.74
143.38
131.53
130.57
130.05
128.78
126.05
120.69
120.03
113.70
111.77
108.63
HCl
f1 (ppm)

^{1}H-NMR, ^{13}C-NMR, and ^{19}F-NMR for compound **29**

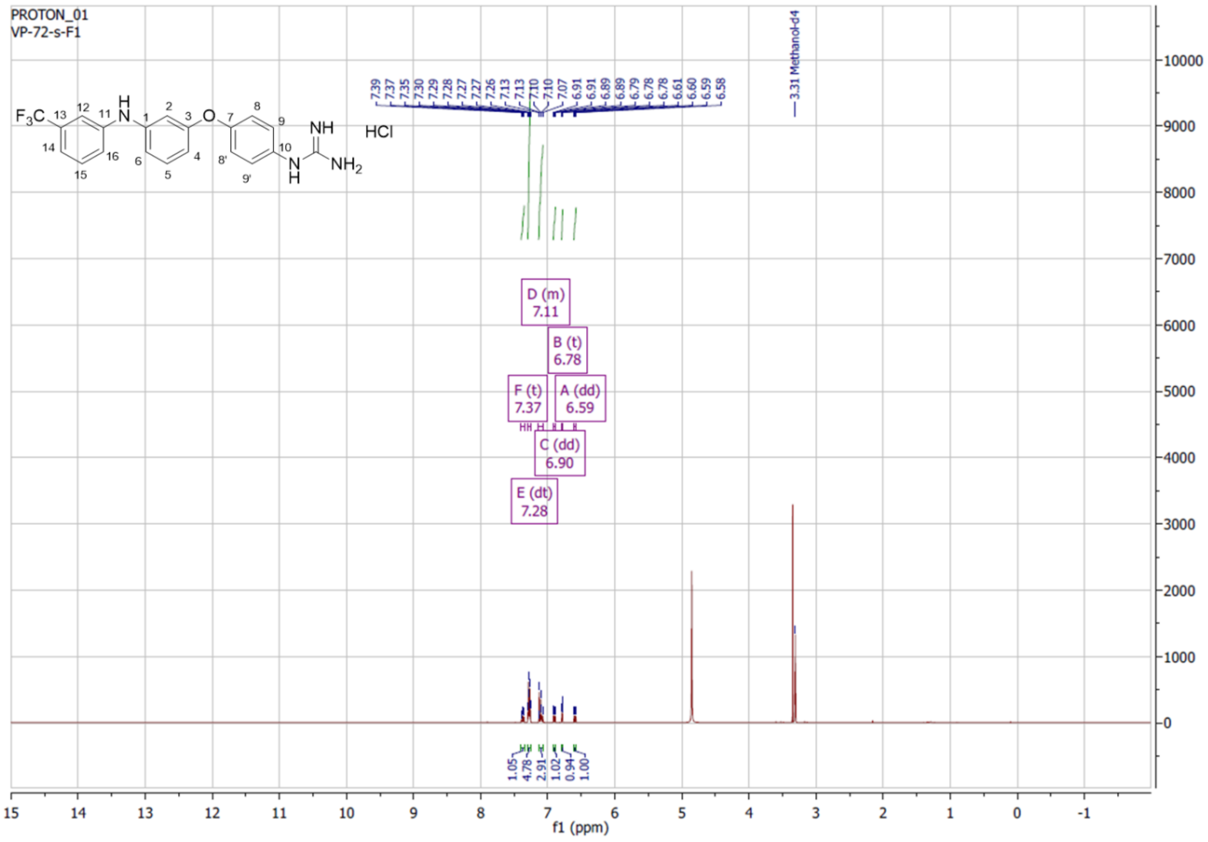

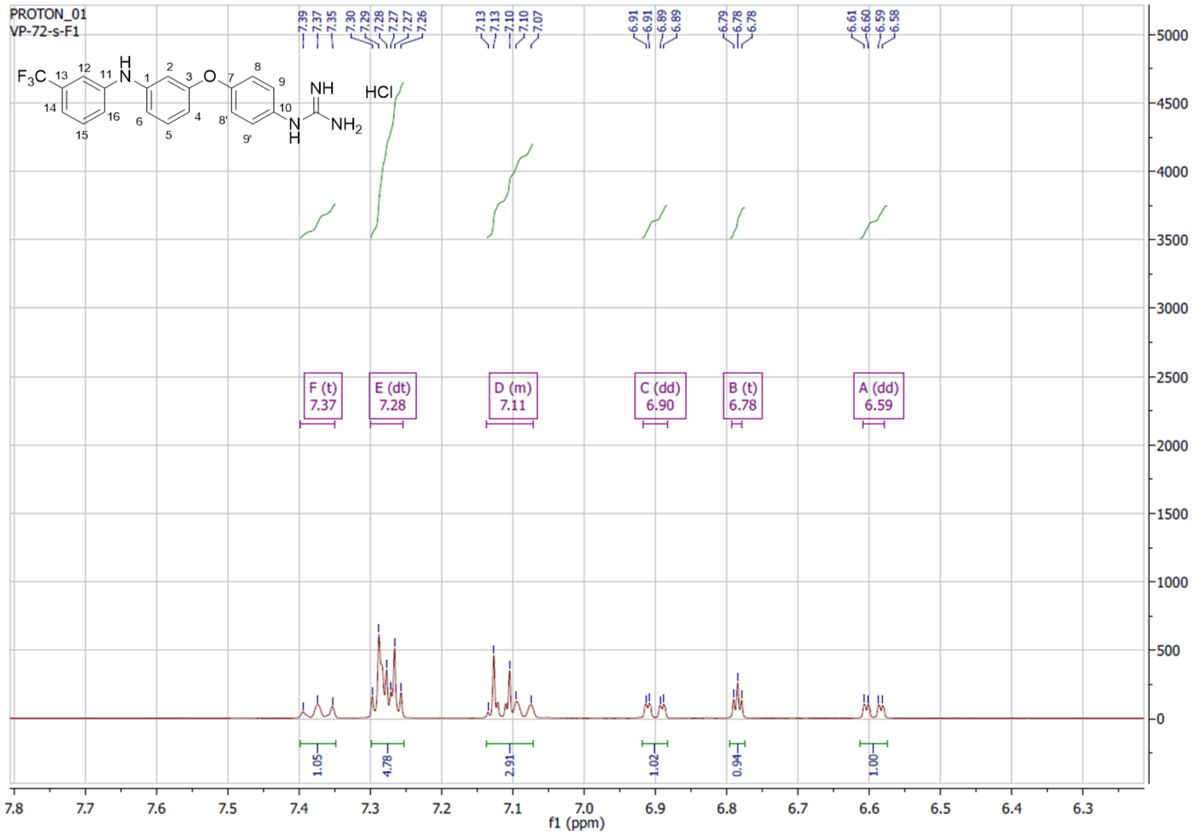

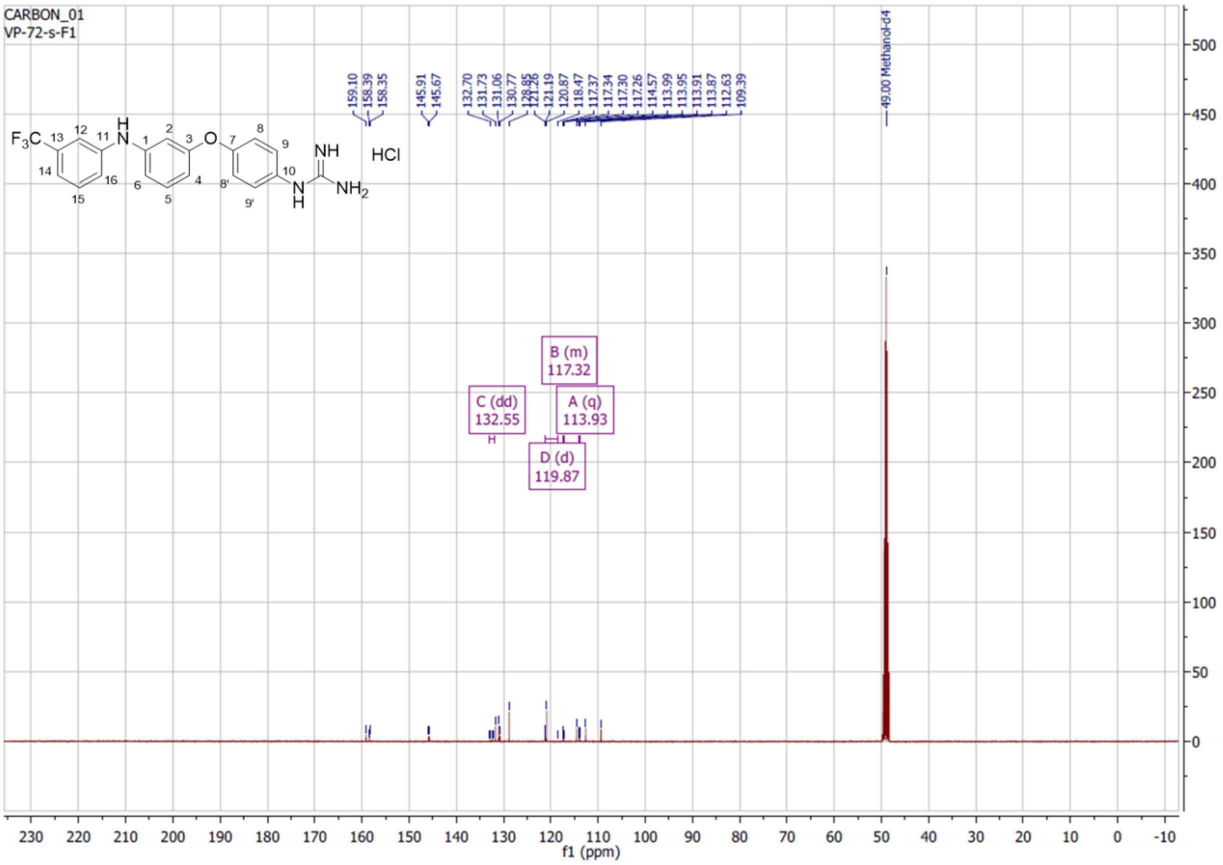
CARBON_01
VP-72-s-F1
HCl
159.10 158.39 158.35 145.91 145.67 132.70 131.73 131.06 130.77 128.85 121.26 121.19 120.87 118.47 117.37 117.34 117.30 117.26 114.57 113.99 113.95 113.91 113.87 112.63 109.39
49.00 Methanol-d4
B (m) 117.32
C (dd) 132.55
A (q) 113.93
D (d) 119.87
f1 (ppm)

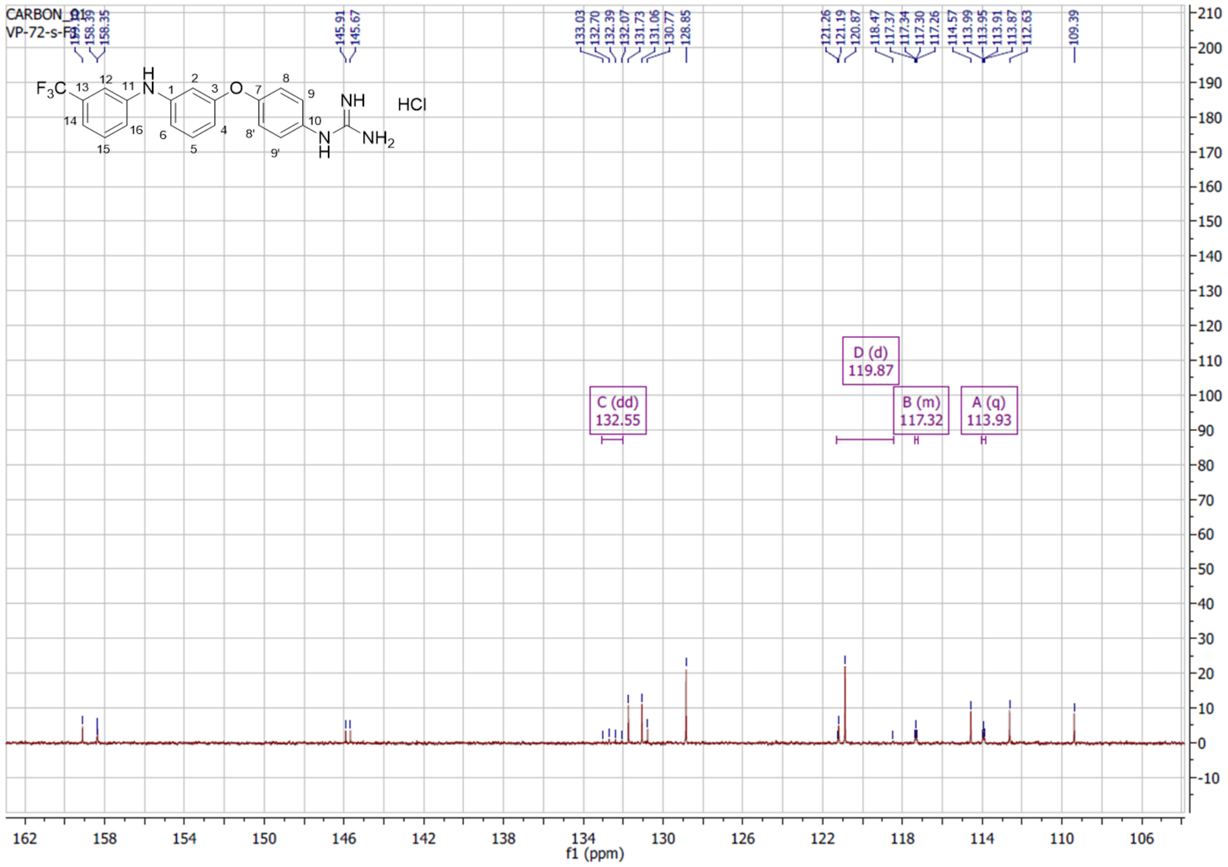
CARBON_01
VP-72-s-F1
HCl
133.03 132.70 132.39 132.07 131.73 131.06 130.77 128.85
121.26 121.19 120.87 118.47 117.37 117.34 117.30 117.26 114.57 113.99 113.95 113.91 113.87 112.63 109.39
D (d) 119.87
C (dd) 132.55
B (m) 117.32
A (q) 113.93
f1 (ppm)

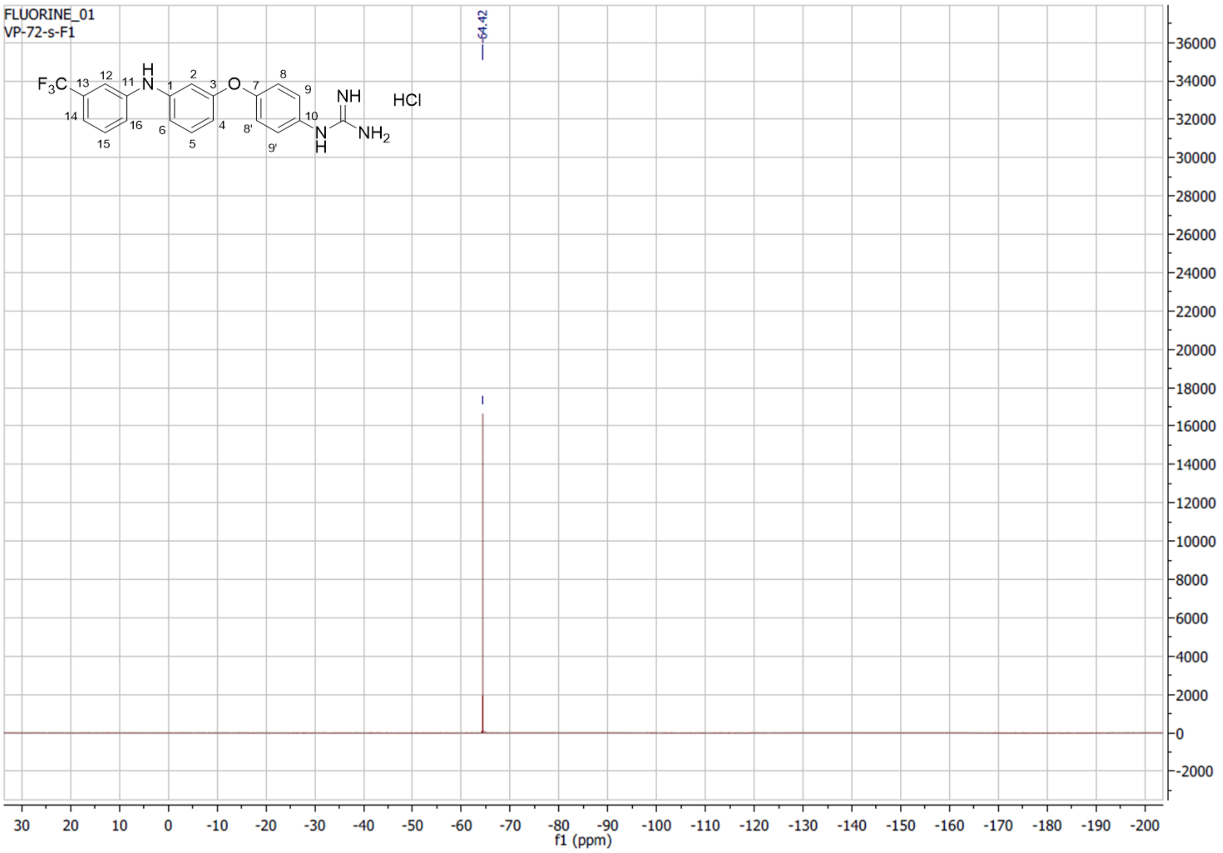
FLUORINE_01
VP-72-s-F1
HCl
-64.42
f1 (ppm)

^{1}H-NMR, ^{13}C-NMR, and ^{19}F-NMR for compound **30**

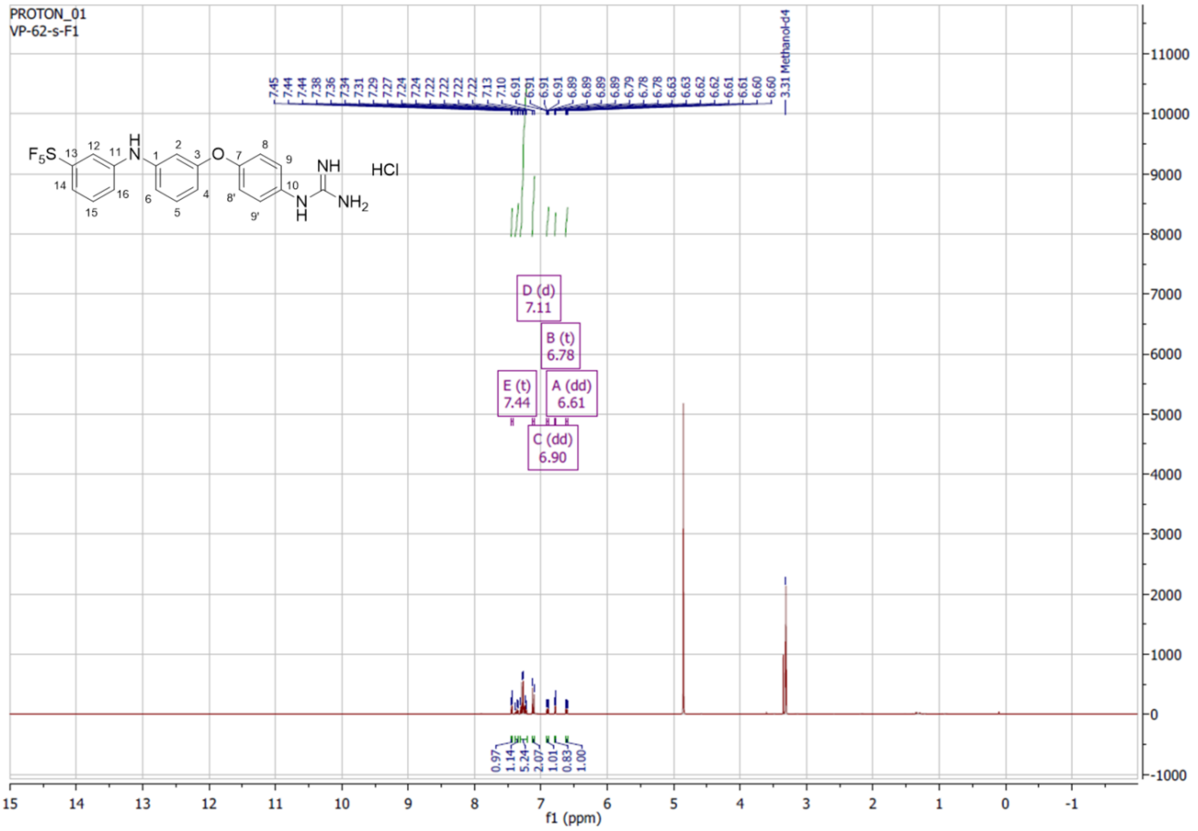

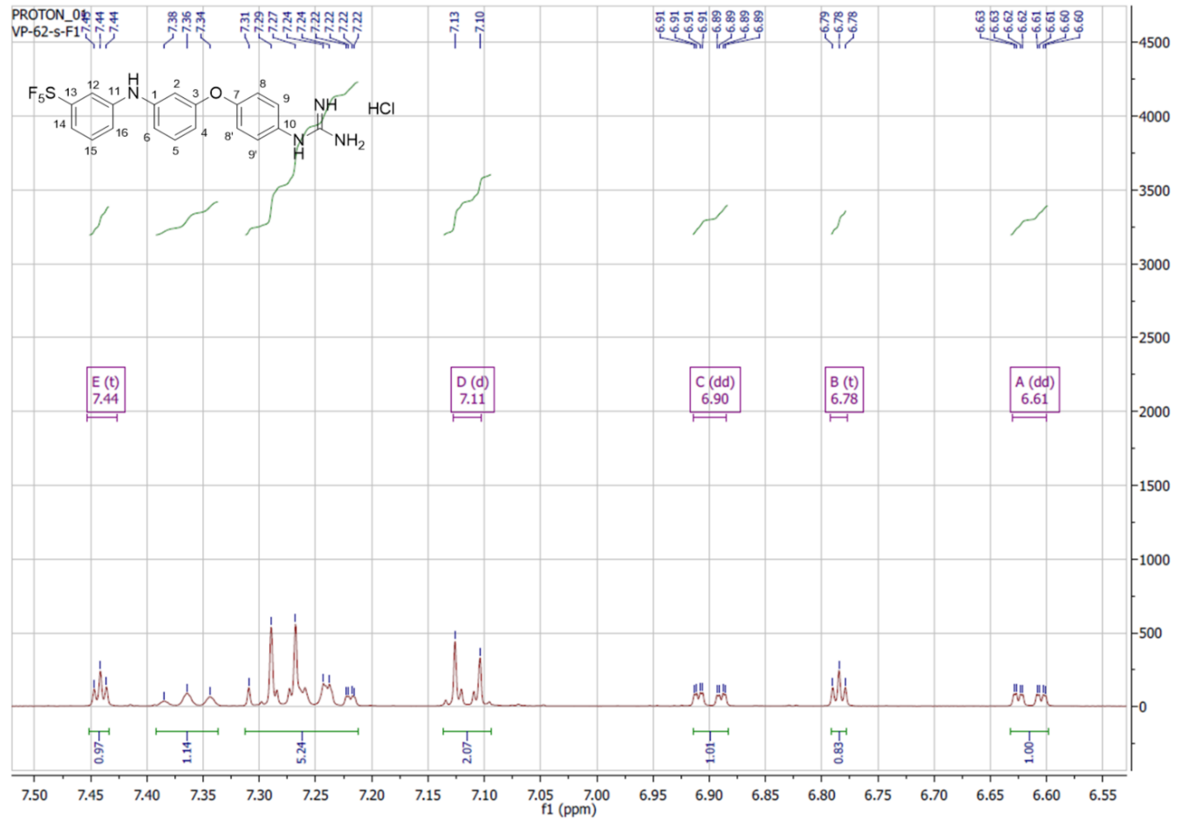

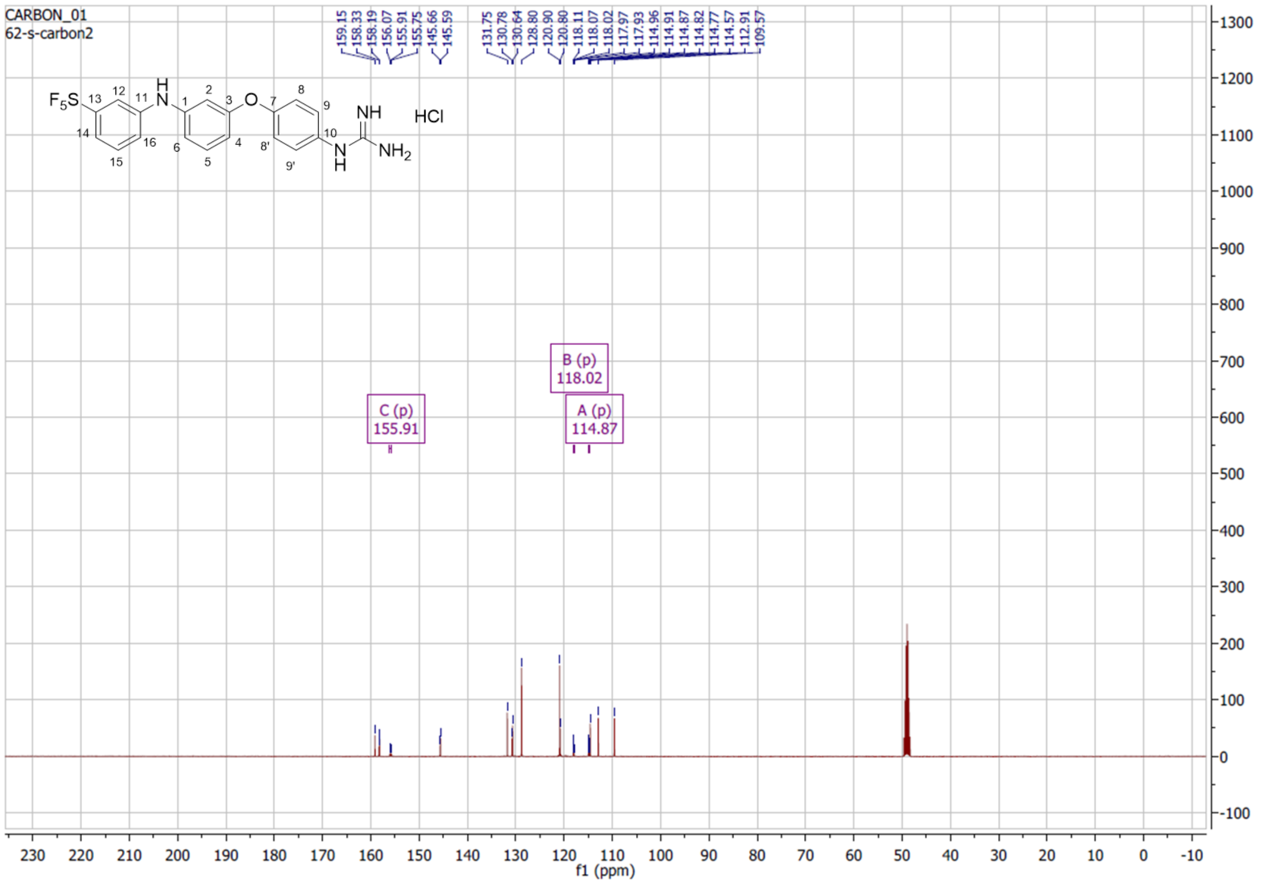
CARBON_01
62-s-carbon2
159.15
158.33
158.19
156.07
155.91
155.75
145.66
145.59
131.75
130.78
130.64
128.80
120.90
120.80
118.11
118.07
118.02
117.97
117.93
114.96
114.91
114.87
114.82
114.77
114.57
112.91
109.57
HCl
C (p)
155.91
B (p)
118.02
A (p)
114.87
f1 (ppm)

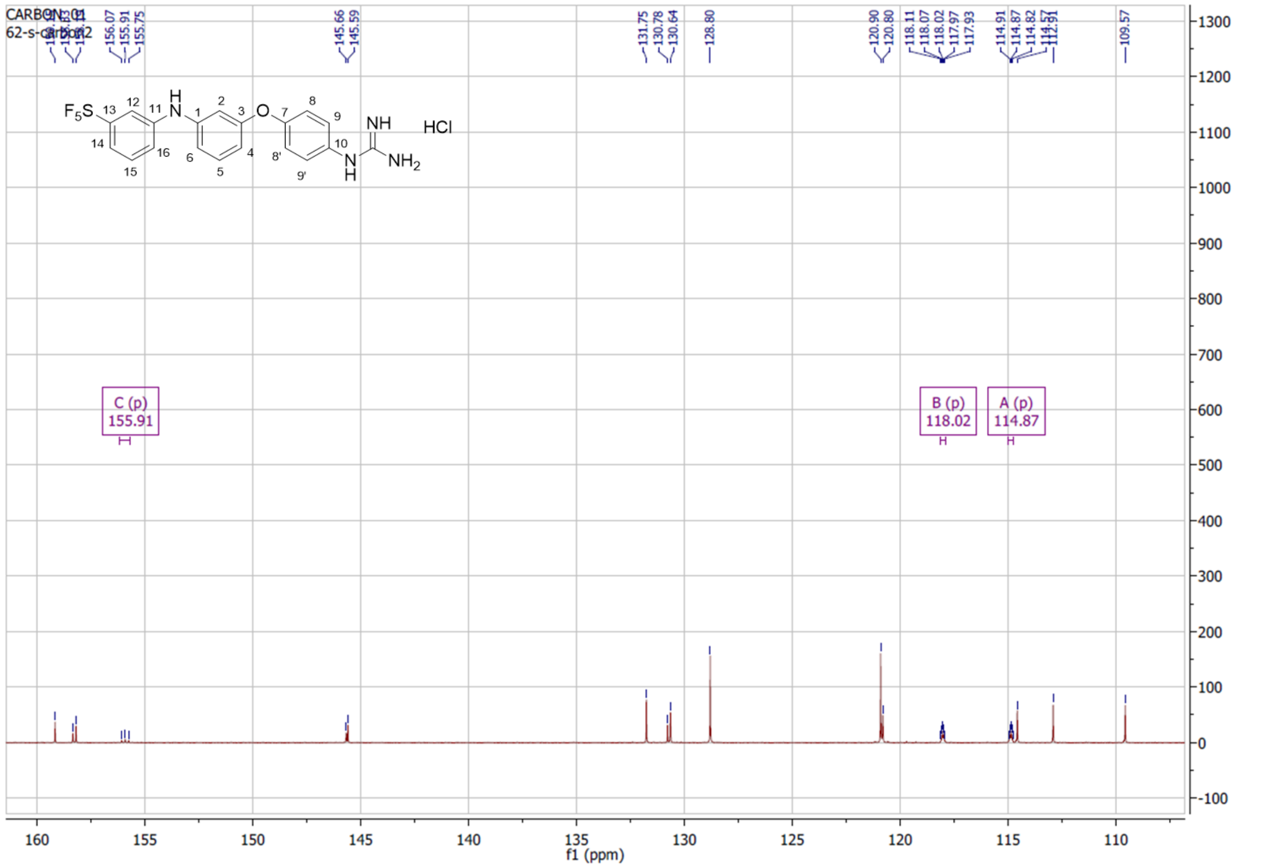
CARBON_01
62-s-carbon2
156.07
155.91
155.75
145.66
145.59
131.75
130.78
130.64
128.80
120.90
120.80
118.11
118.07
118.02
117.97
117.93
114.91
114.87
114.82
109.57
HCl
C (p)
155.91
B (p)
118.02
A (p)
114.87
f1 (ppm)

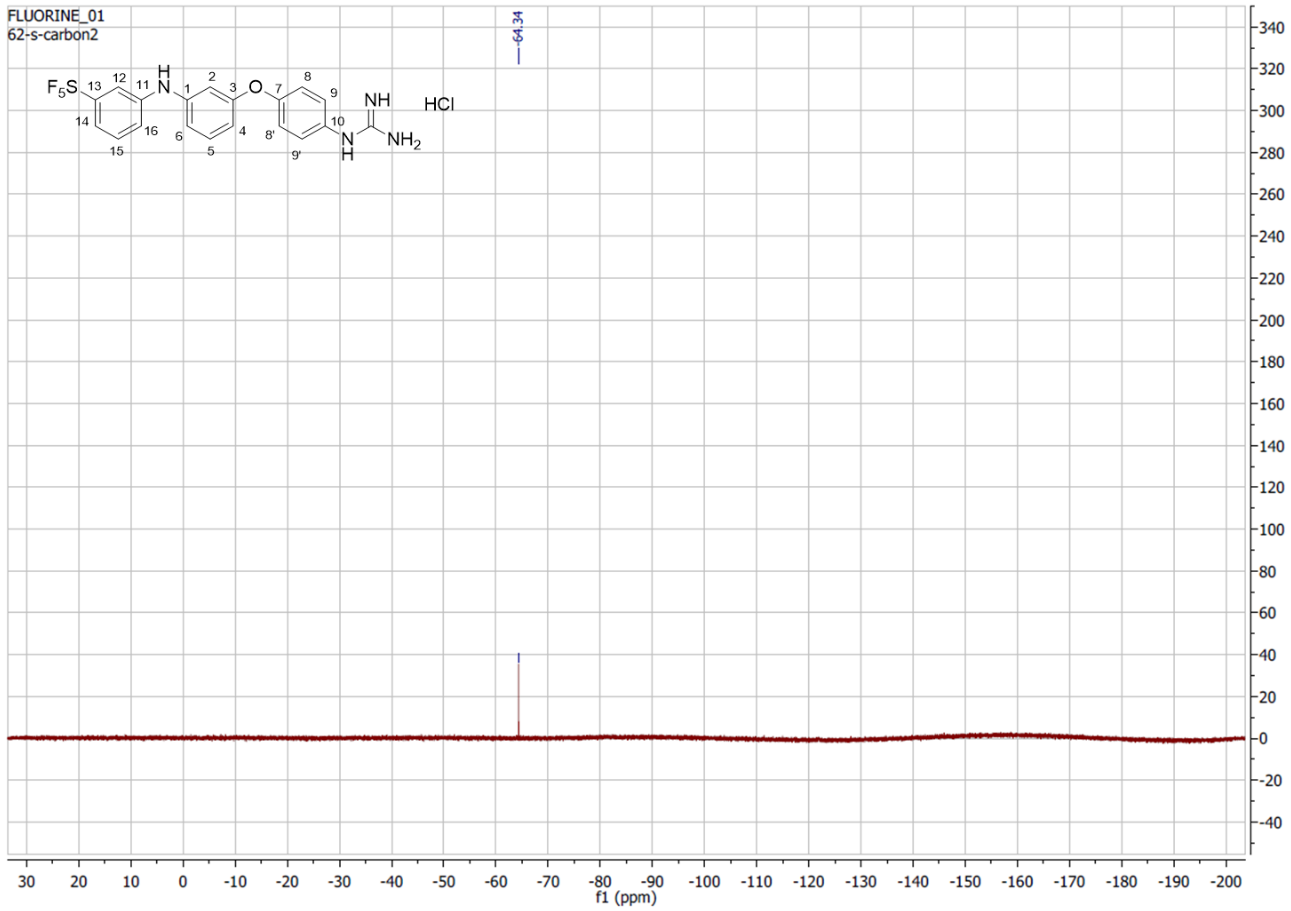
FLUORINE_01
62-s-carbon2
HCl
-64.34
f1 (ppm)

^{1}H-NMR, ^{13}C-NMR, and ^{19}F-NMR for compound **36**

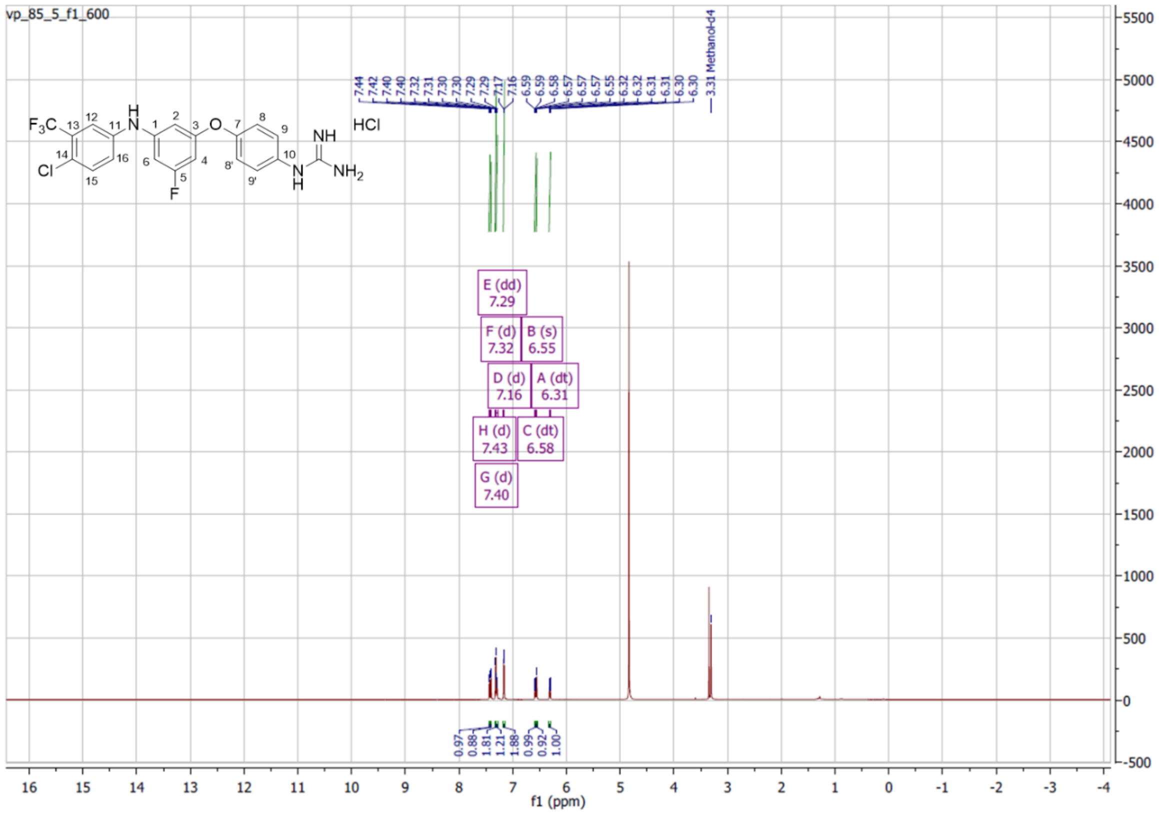

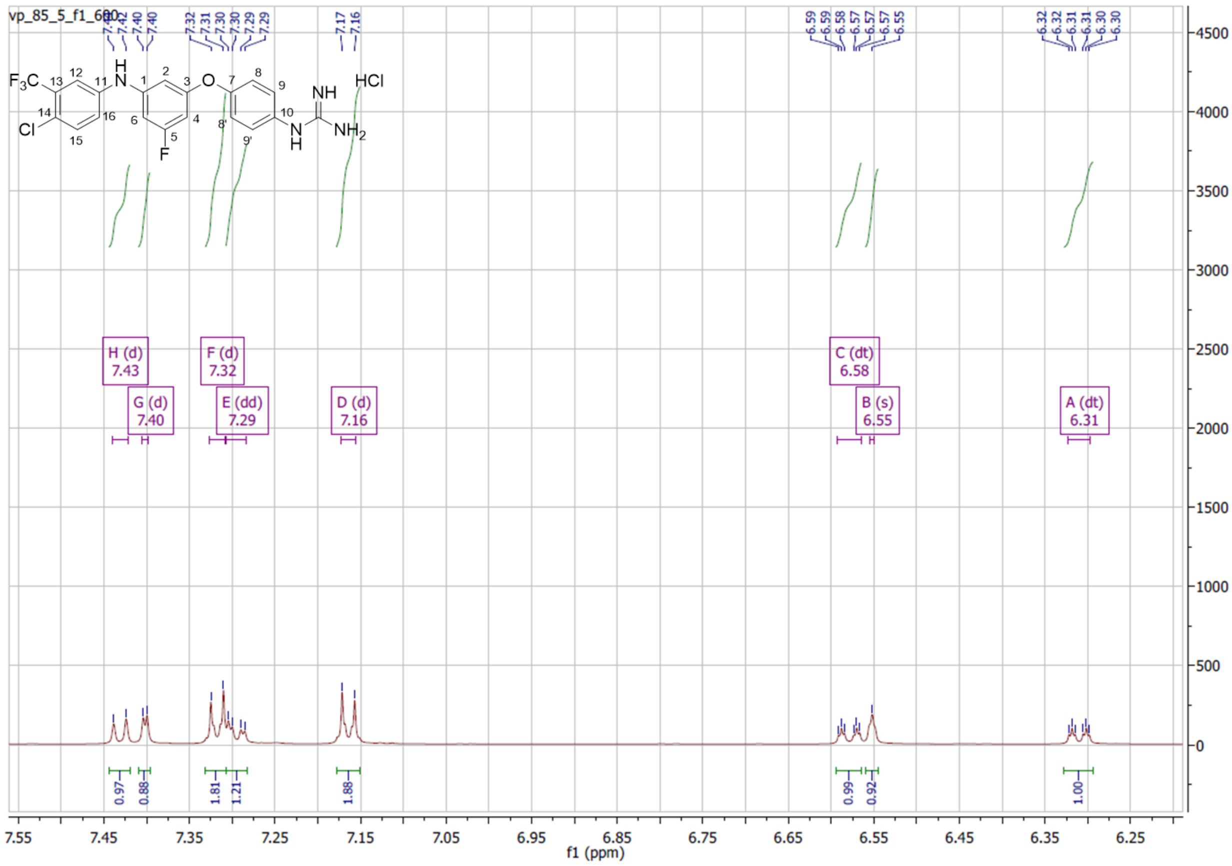

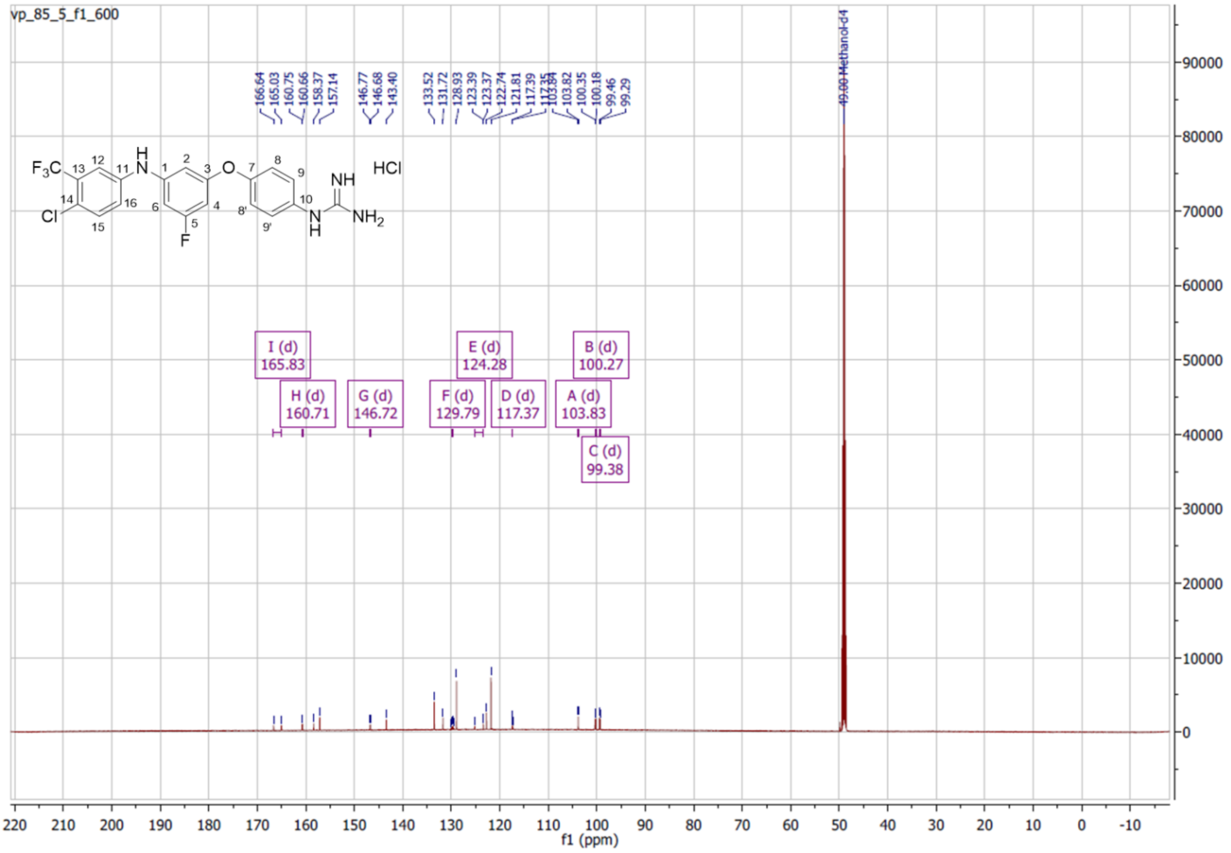
vp_85_5_f1_600
HCl
I (d) 165.83
H (d) 160.71
G (d) 146.72
F (d) 129.79
E (d) 124.28
D (d) 117.37
A (d) 103.83
B (d) 100.27
C (d) 99.38
49.00 Methanol-d4
f1 (ppm)

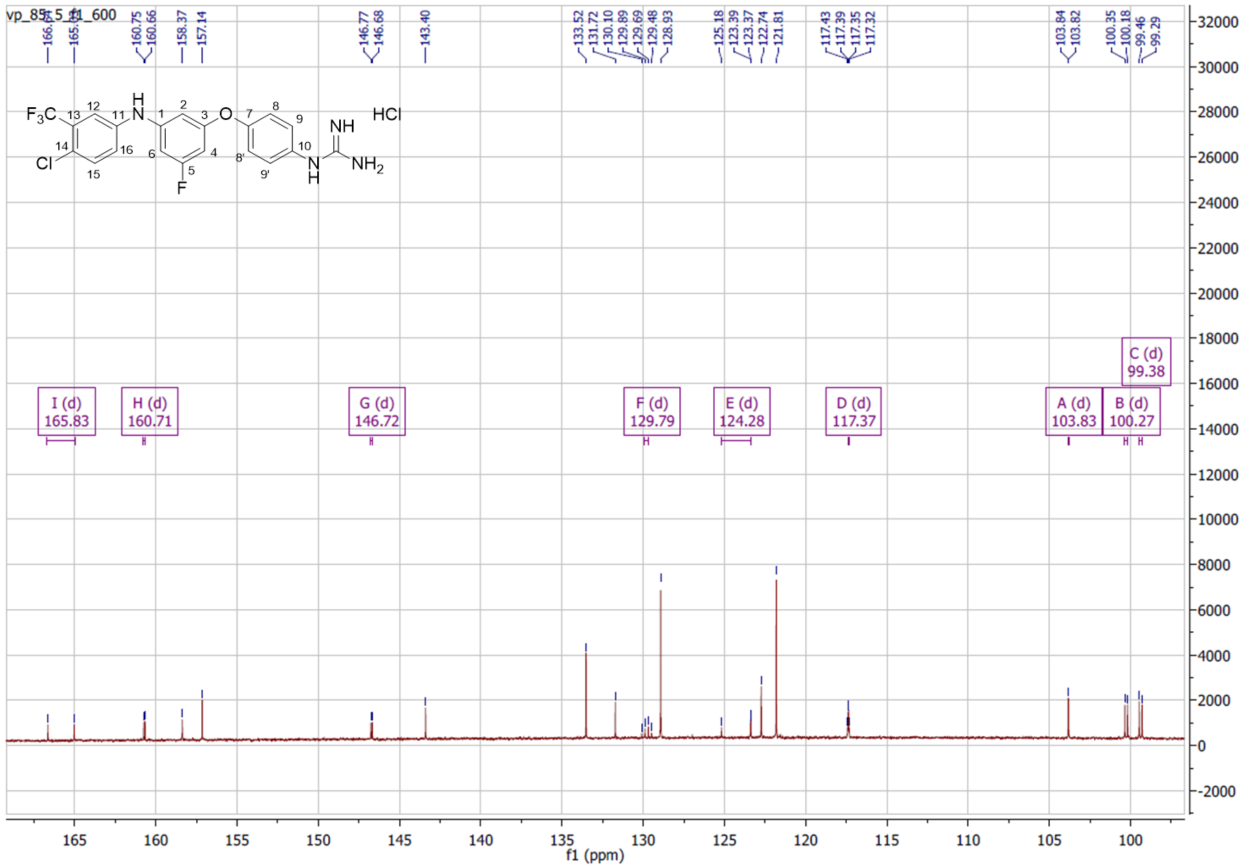
vp_85_5_f1_600
HCl
I (d) 165.83
H (d) 160.71
G (d) 146.72
F (d) 129.79
E (d) 124.28
D (d) 117.37
A (d) 103.83
B (d) 100.27
C (d) 99.38
f1 (ppm)

vp_85_5_f1_600
TFT 19F SENSITIVITY=198:1 5MM QNP PROBE

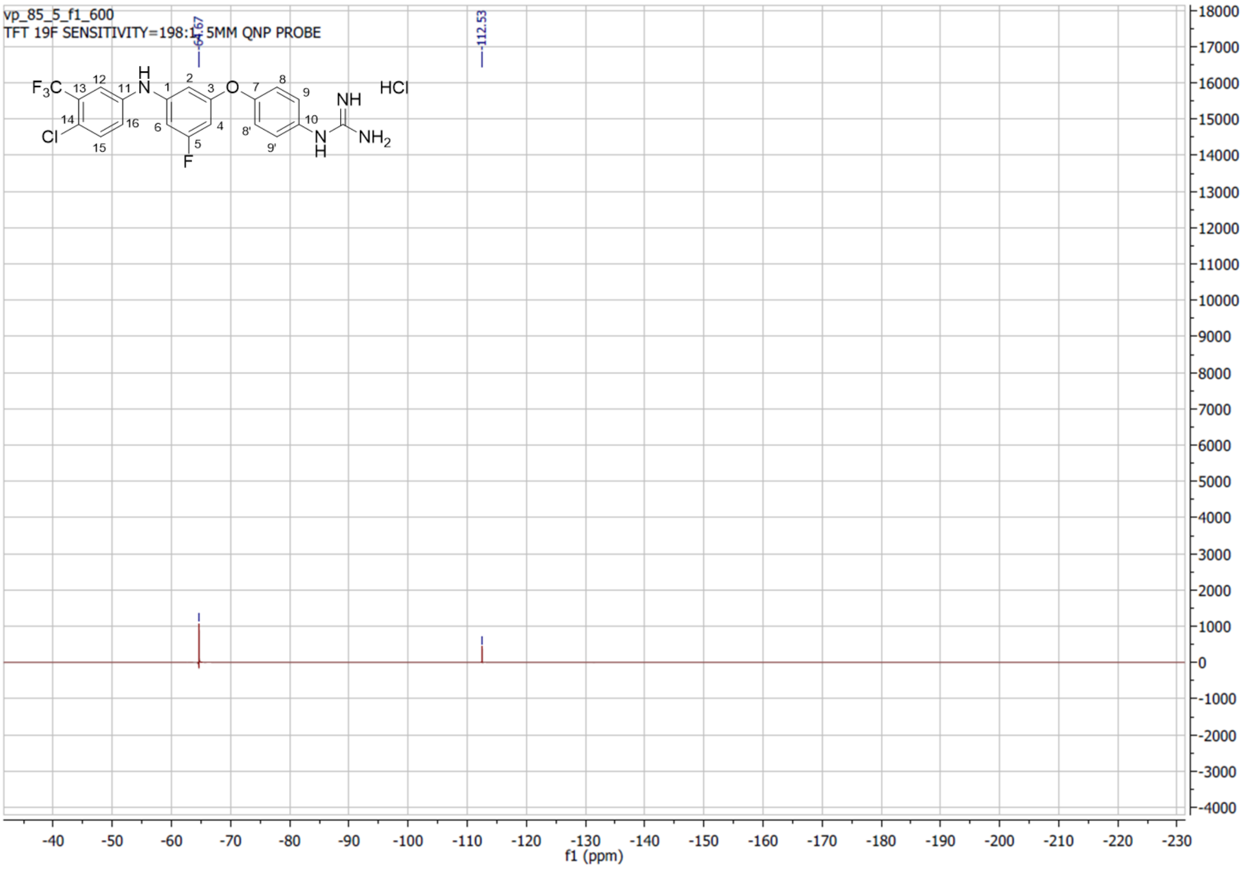

^{1}H-NMR, ^{13}C-NMR, and ^{19}F-NMR for compound **49**

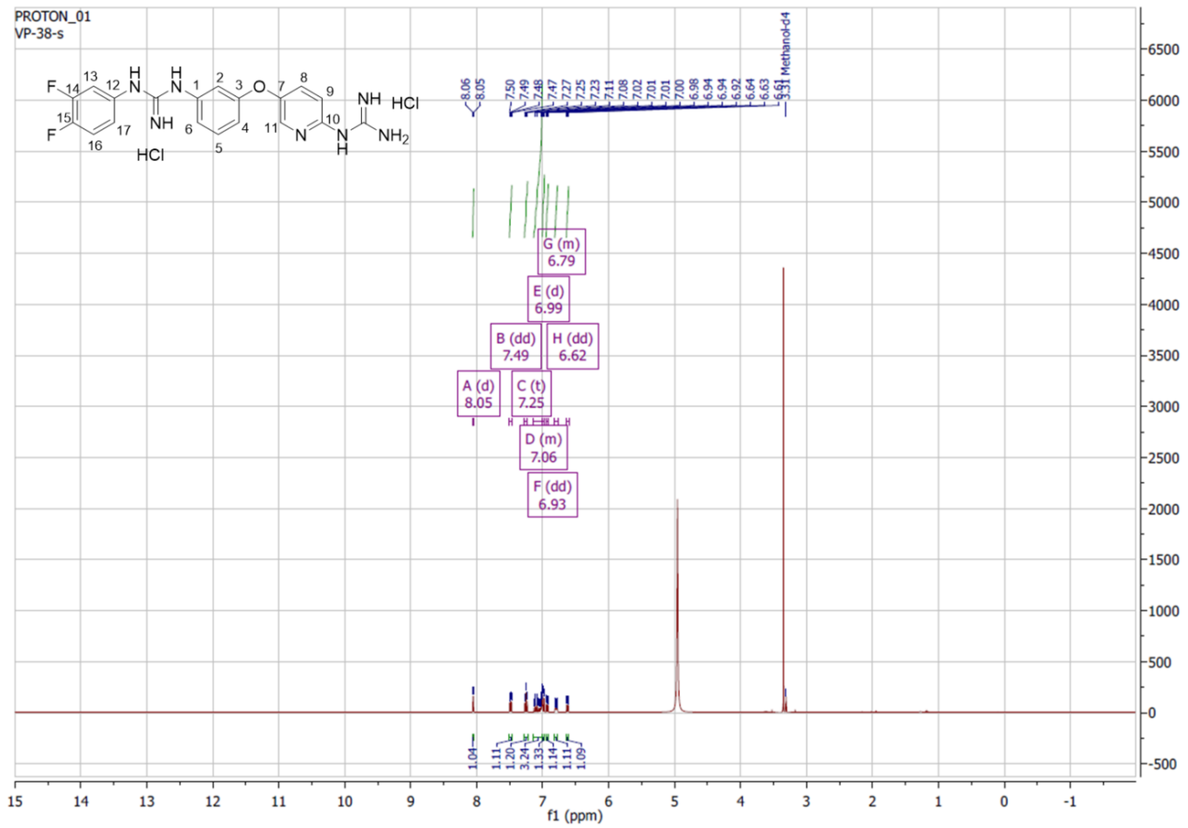

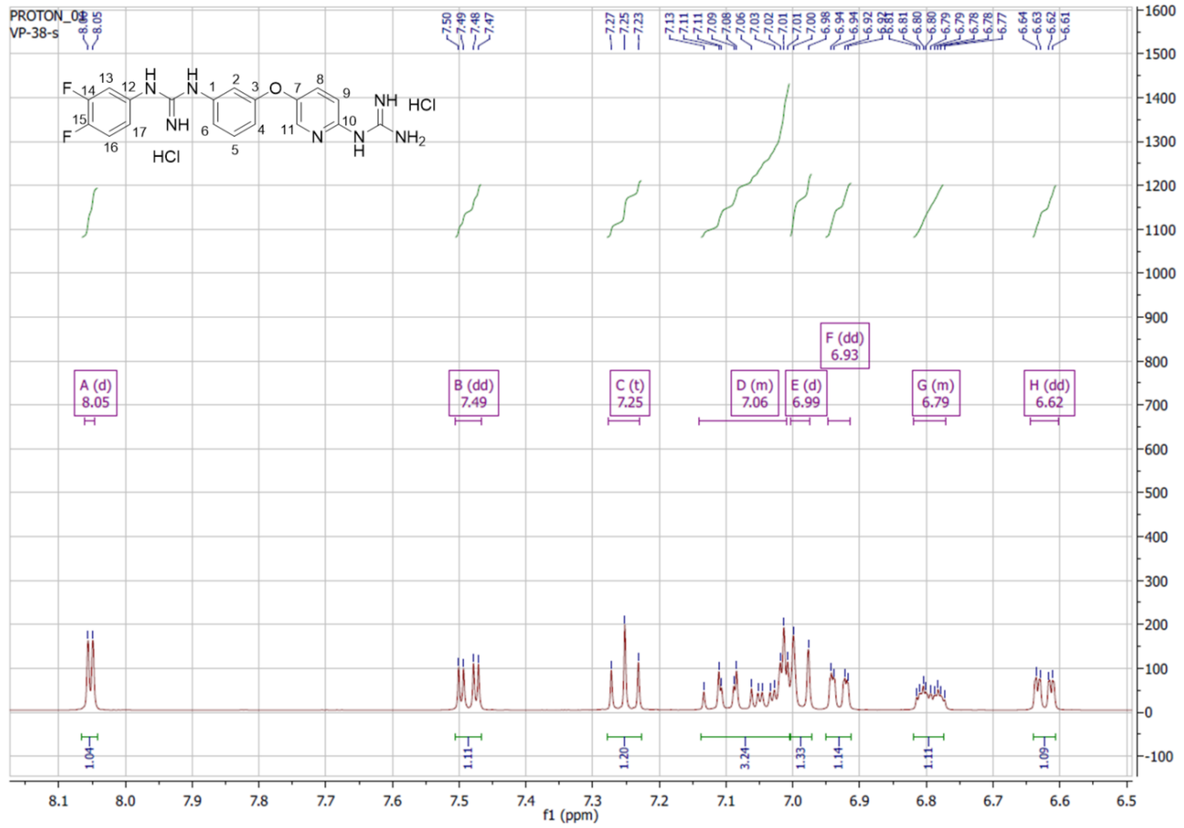

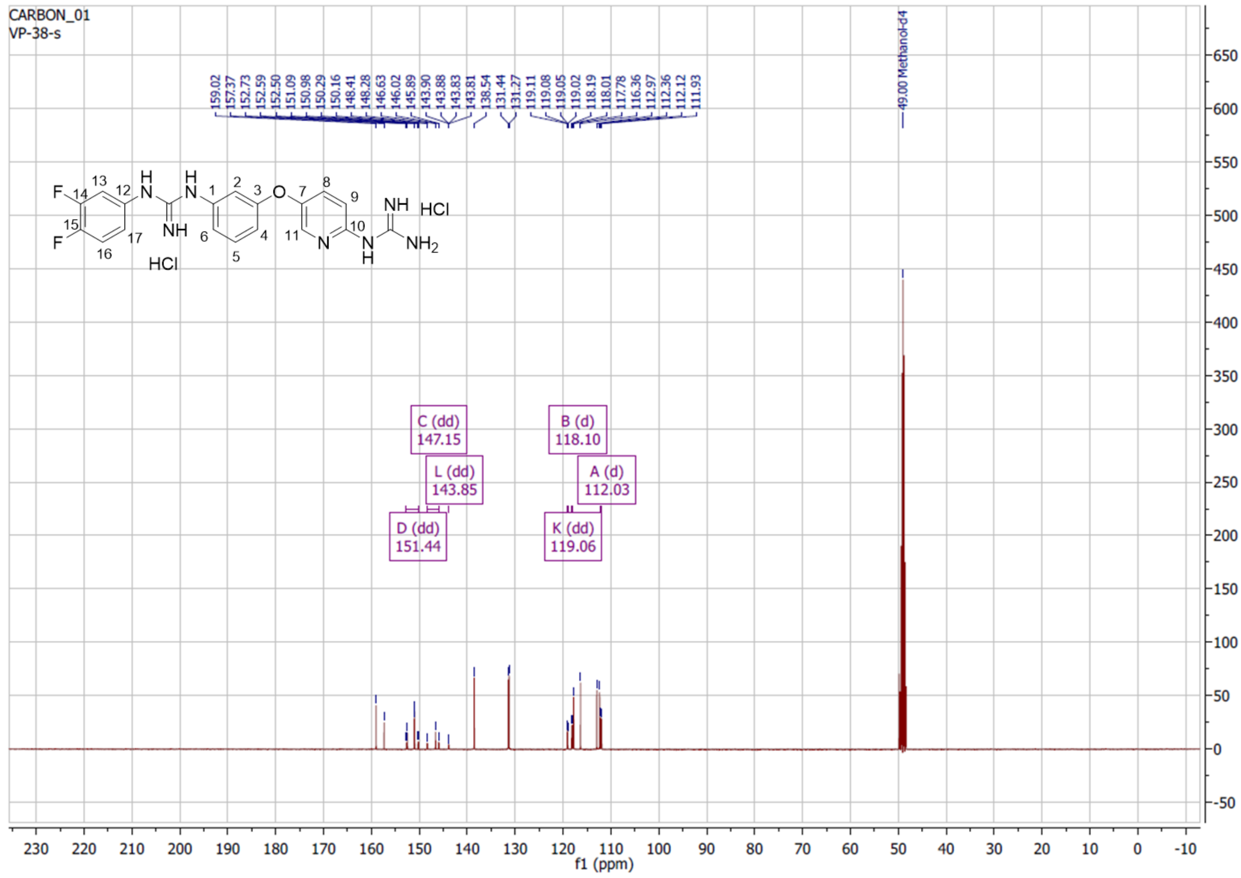
CARBON_01
VP-38-s
159.02
157.37
152.73
152.59
152.50
151.09
150.98
150.29
150.16
148.41
148.28
146.63
146.02
145.89
143.90
143.88
143.83
143.81
138.54
131.44
131.27
119.11
119.08
119.05
119.02
118.19
118.01
117.78
116.36
112.97
112.36
112.12
111.93
49.00 Methanol-d4
C (dd)
147.15
B (d)
118.10
L (dd)
143.85
A (d)
112.03
D (dd)
151.44
K (dd)
119.06
HCl
f1 (ppm)

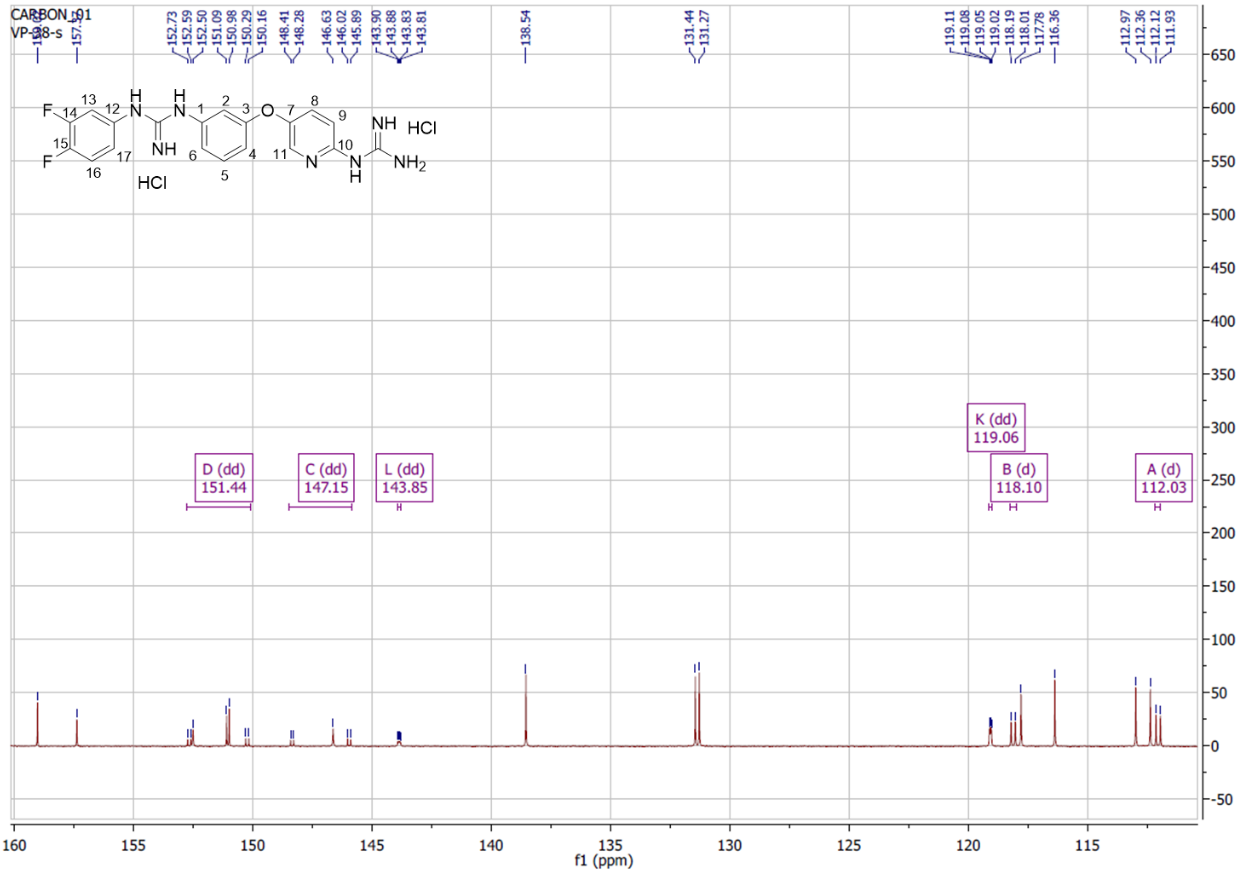
CARBON_01
VP-38-s
157.37
152.73
152.59
152.50
151.09
150.98
150.29
150.16
148.41
148.28
146.63
146.02
145.89
143.90
143.88
143.83
143.81
138.54
131.44
131.27
119.11
119.08
119.05
119.02
118.19
118.01
117.78
116.36
112.97
112.36
112.12
111.93
D (dd)
151.44
C (dd)
147.15
L (dd)
143.85
K (dd)
119.06
B (d)
118.10
A (d)
112.03
HCl
f1 (ppm)

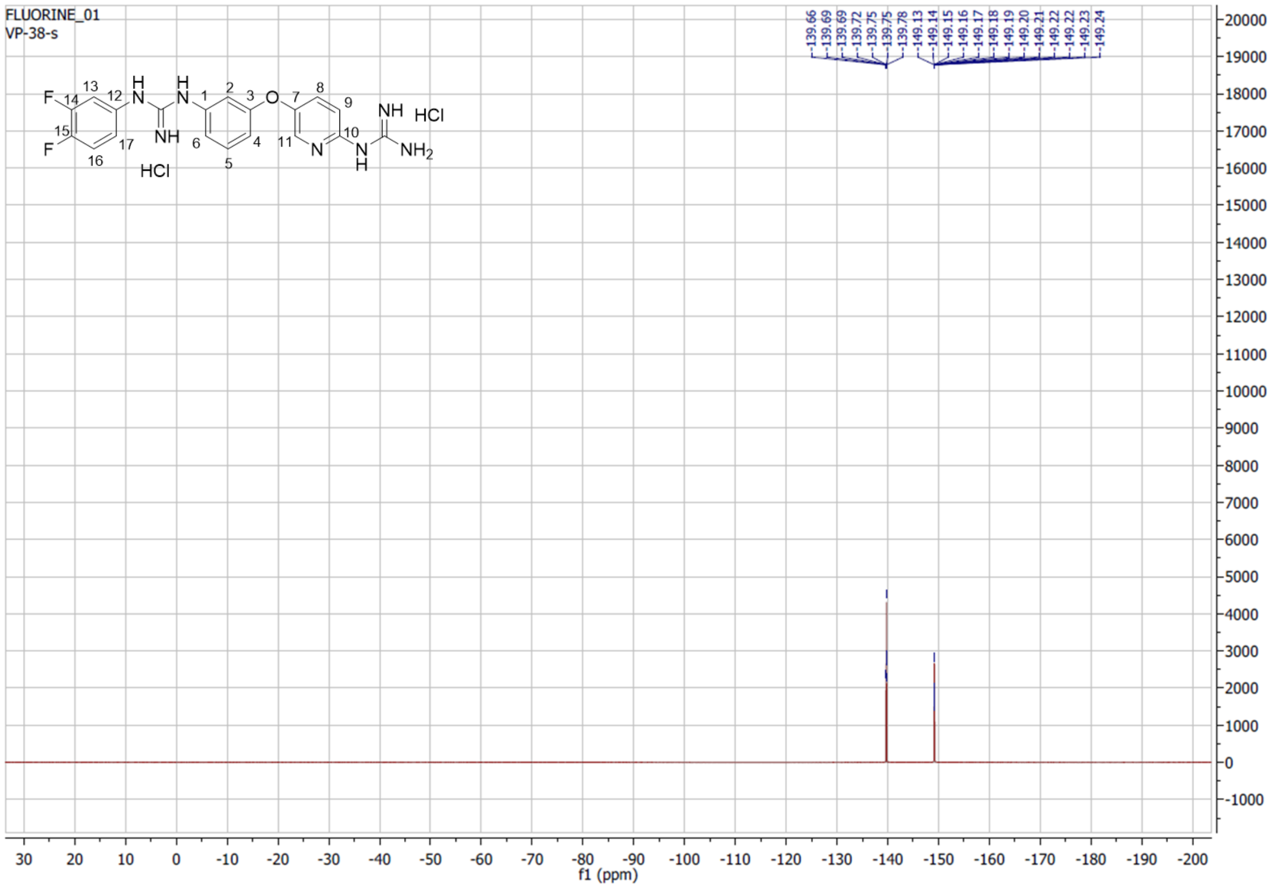
FLUORINE_01
VP-38-s
HCl
HCl
f1 (ppm)

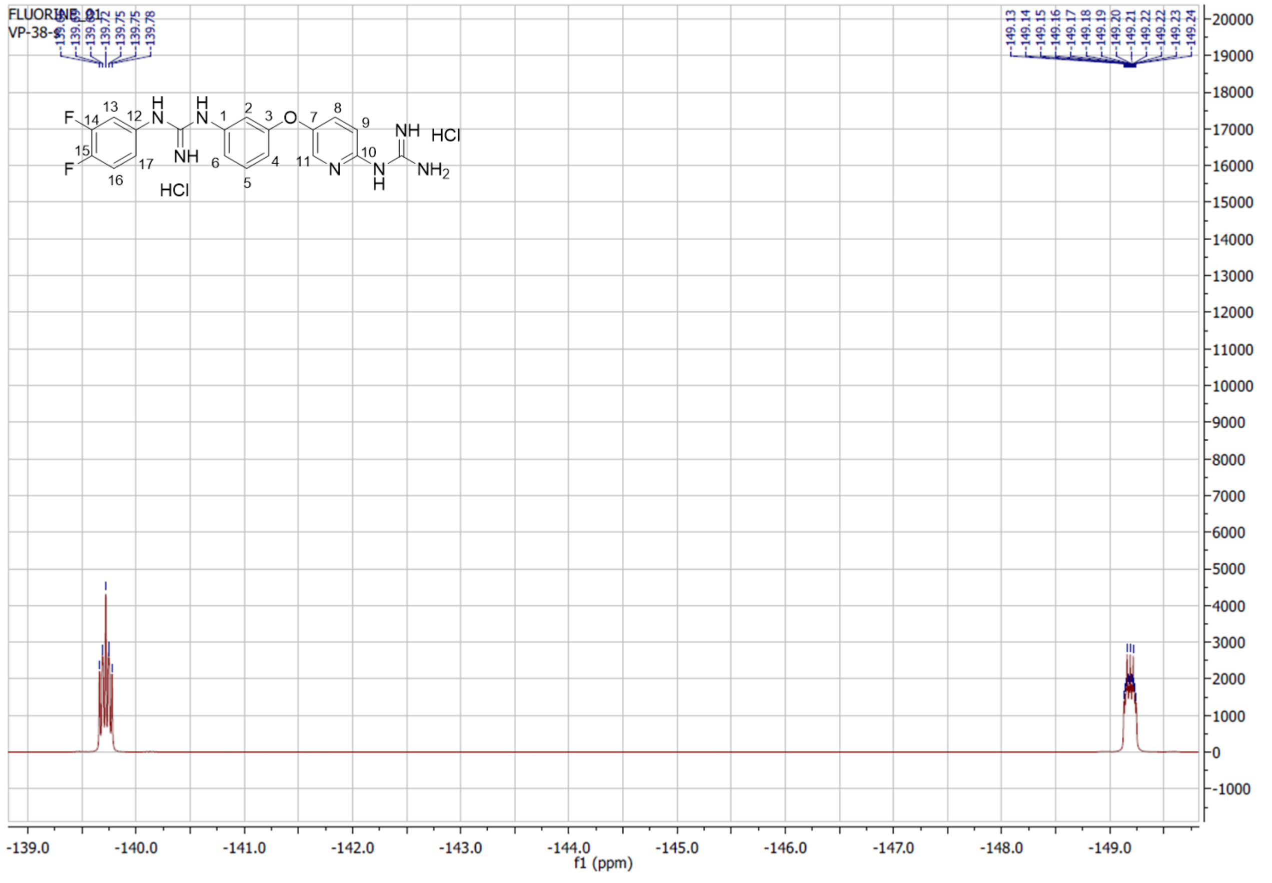
FLUORINE_01
VP-38-s
HCl
HCl
f1 (ppm)

^{1}H-NMR, ^{13}C-NMR, and ^{19}F-NMR for compound **50**

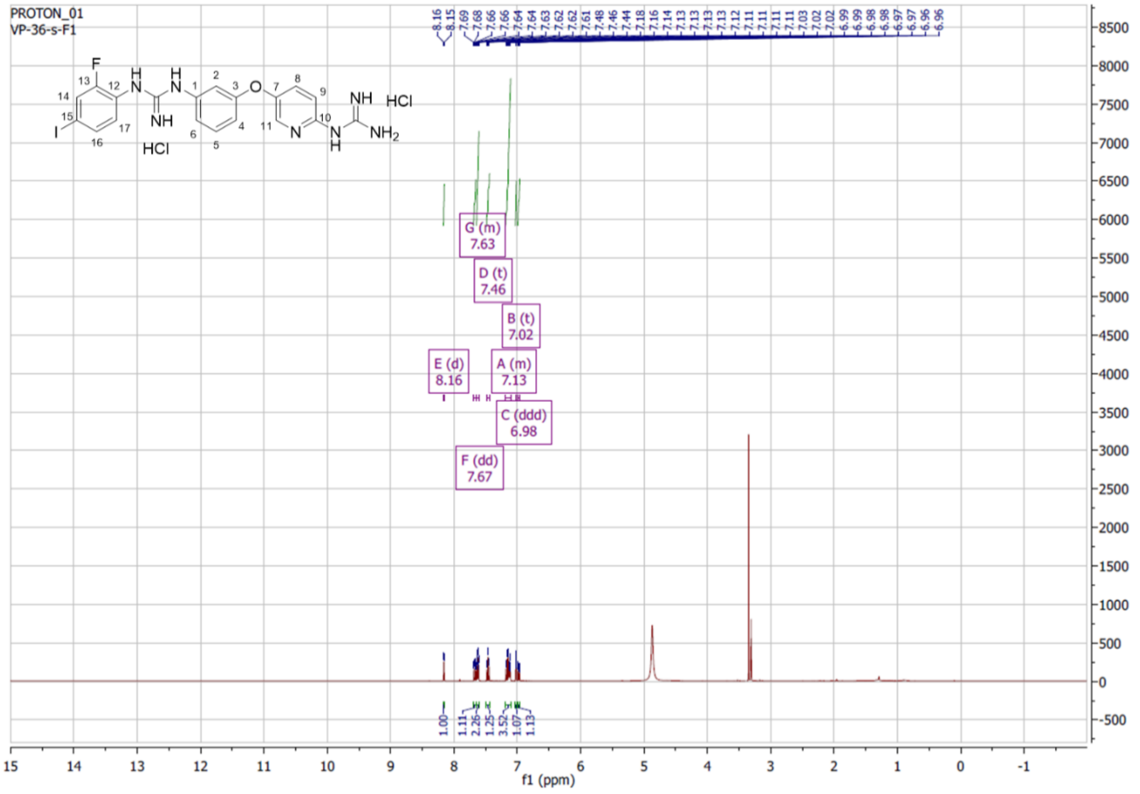

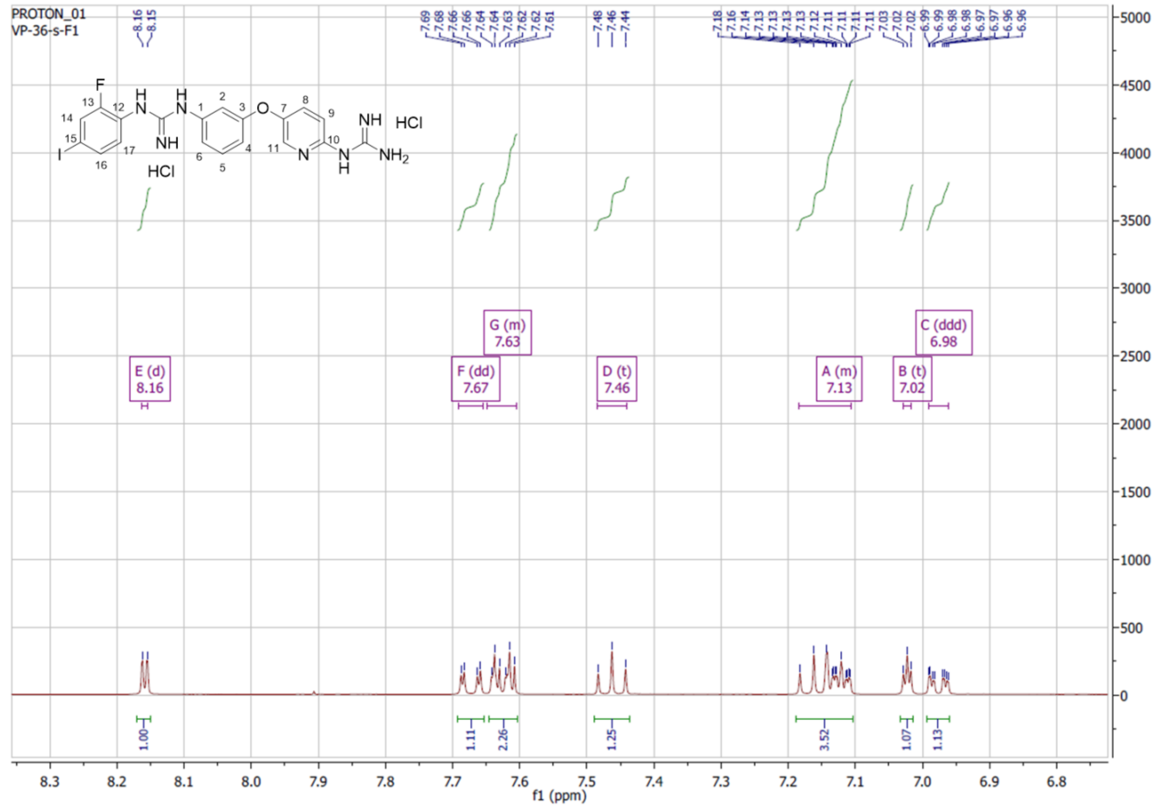

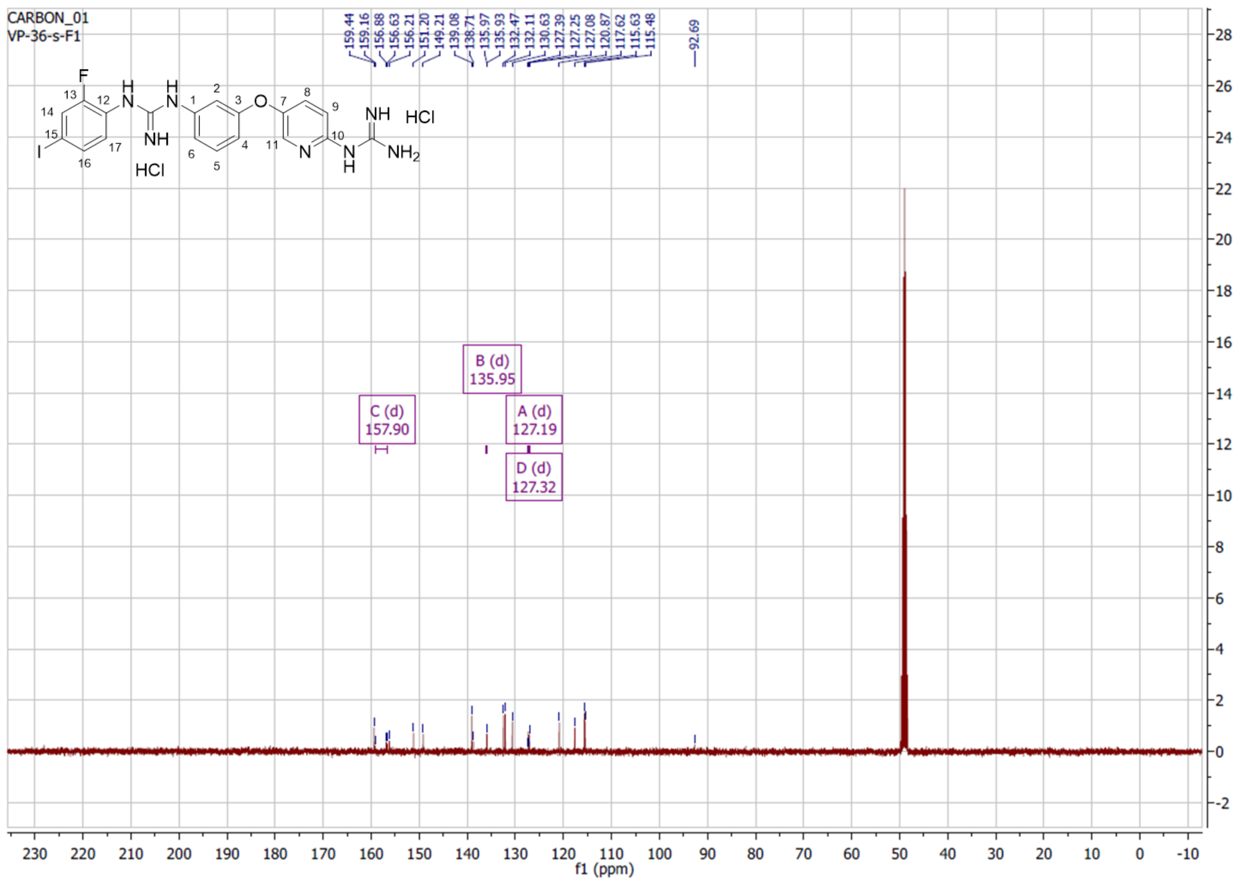
CARBON_01
VP-36-s-F1
159.44
159.16
156.88
156.63
156.21
151.20
149.21
139.08
138.71
135.97
135.93
132.47
132.11
130.63
127.39
127.25
127.08
120.87
117.62
115.63
115.48
92.69
HCl
HCl
C (d)
157.90
B (d)
135.95
A (d)
127.19
D (d)
127.32
f1 (ppm)

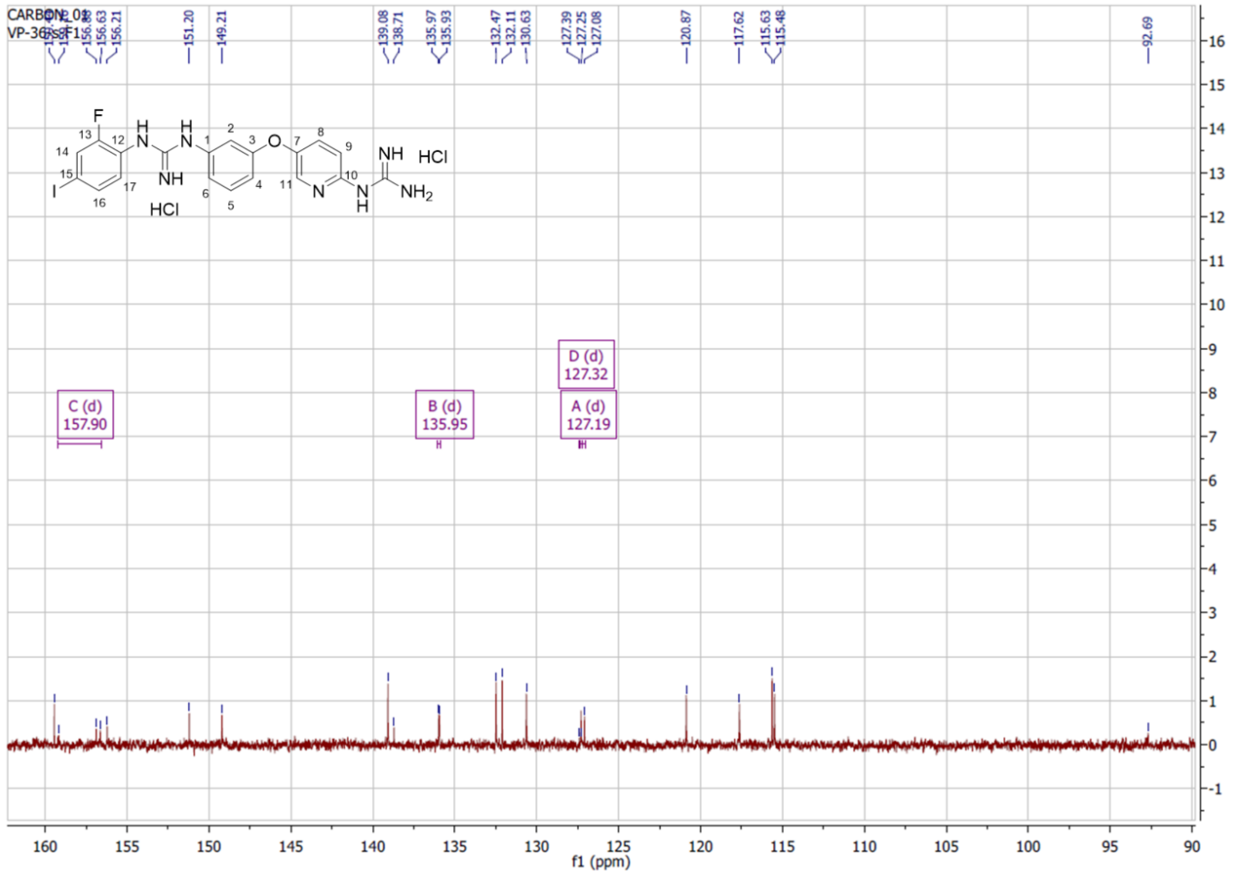
151.20
149.21
139.08
138.71
135.97
135.93
132.47
132.11
130.63
127.39
127.25
127.08
120.87
117.62
115.63
115.48
92.69
HCl
HCl
C (d)
157.90
B (d)
135.95
D (d)
127.32
A (d)
127.19
f1 (ppm)

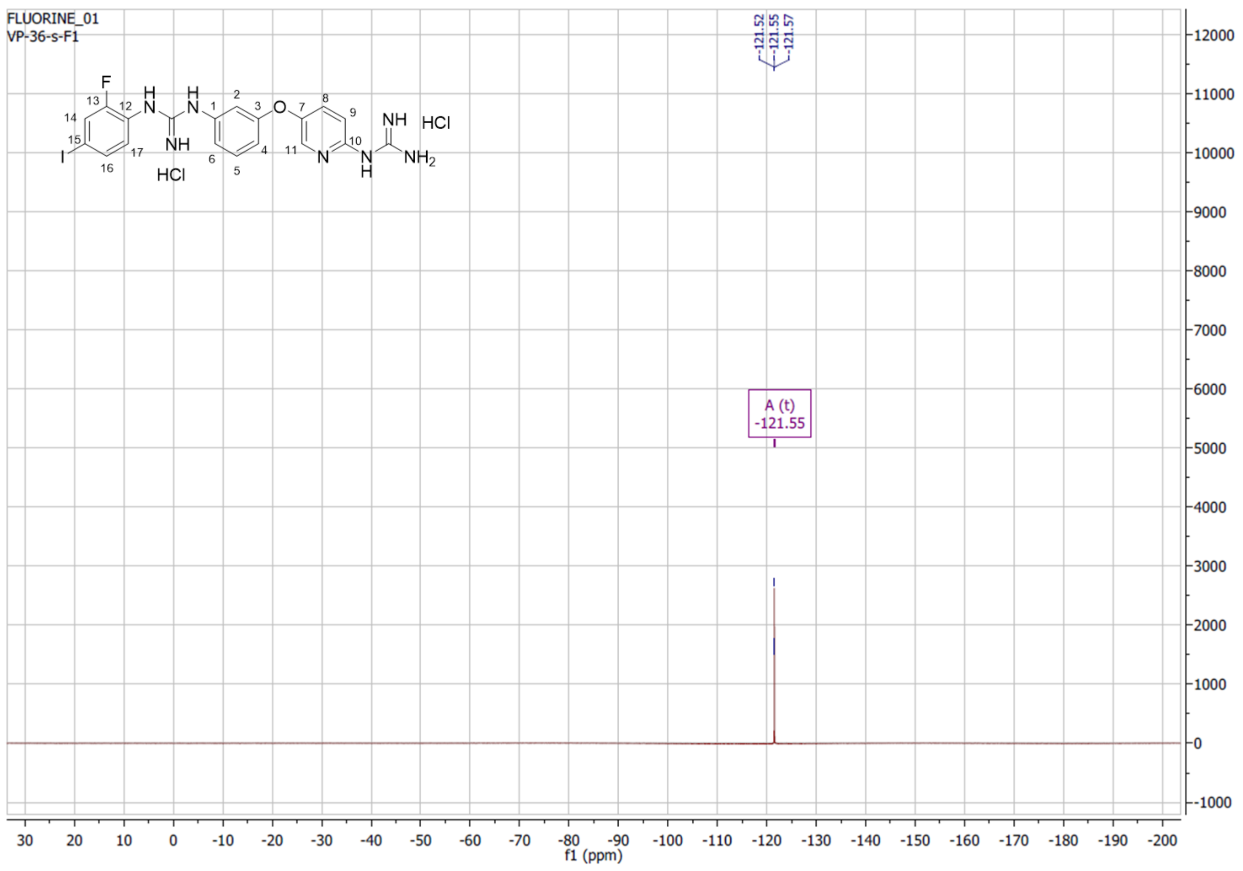

FLUORINE_01
VP-36-s-F1
-121.52
-121.55
-121.57
A (t)
-121.55
F
HCl
HCl
NH
NH
NH2
f1 (ppm)

^{1}H-NMR and ^{13}C-NMR for compound **51**

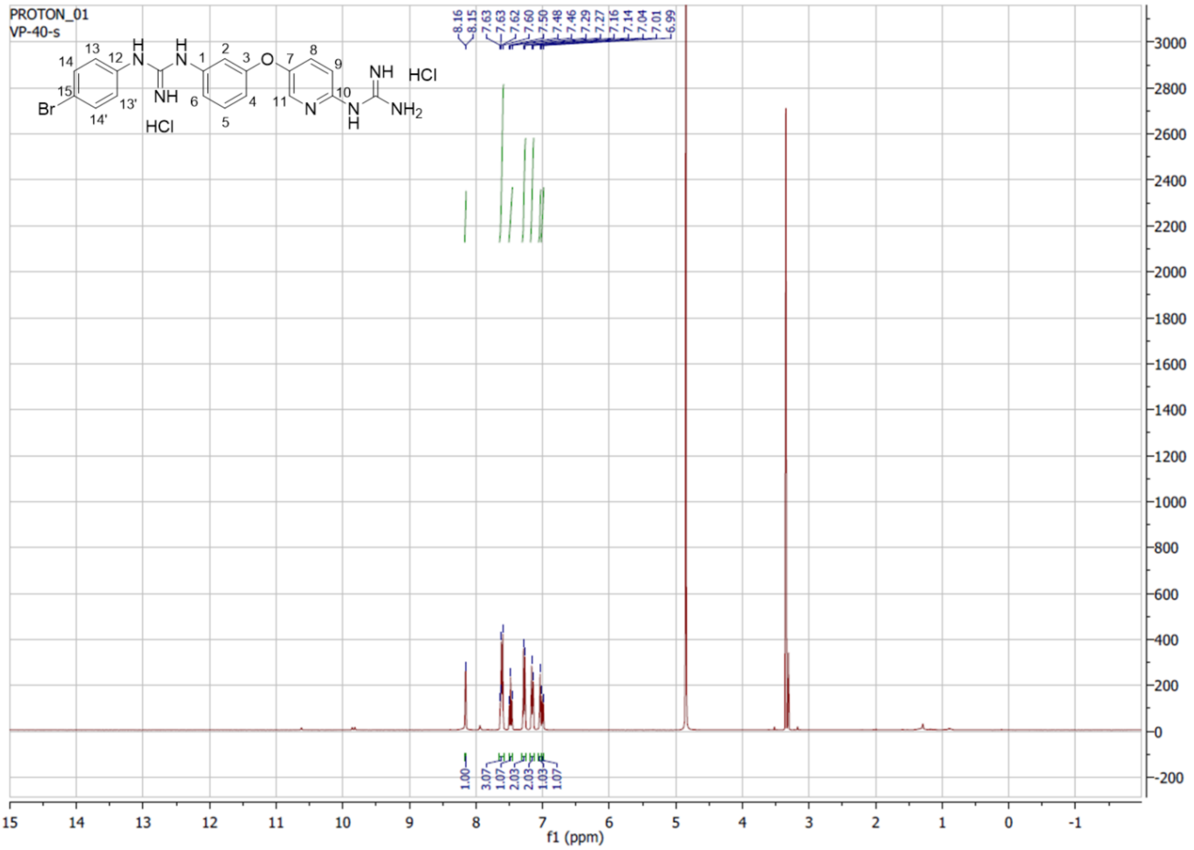

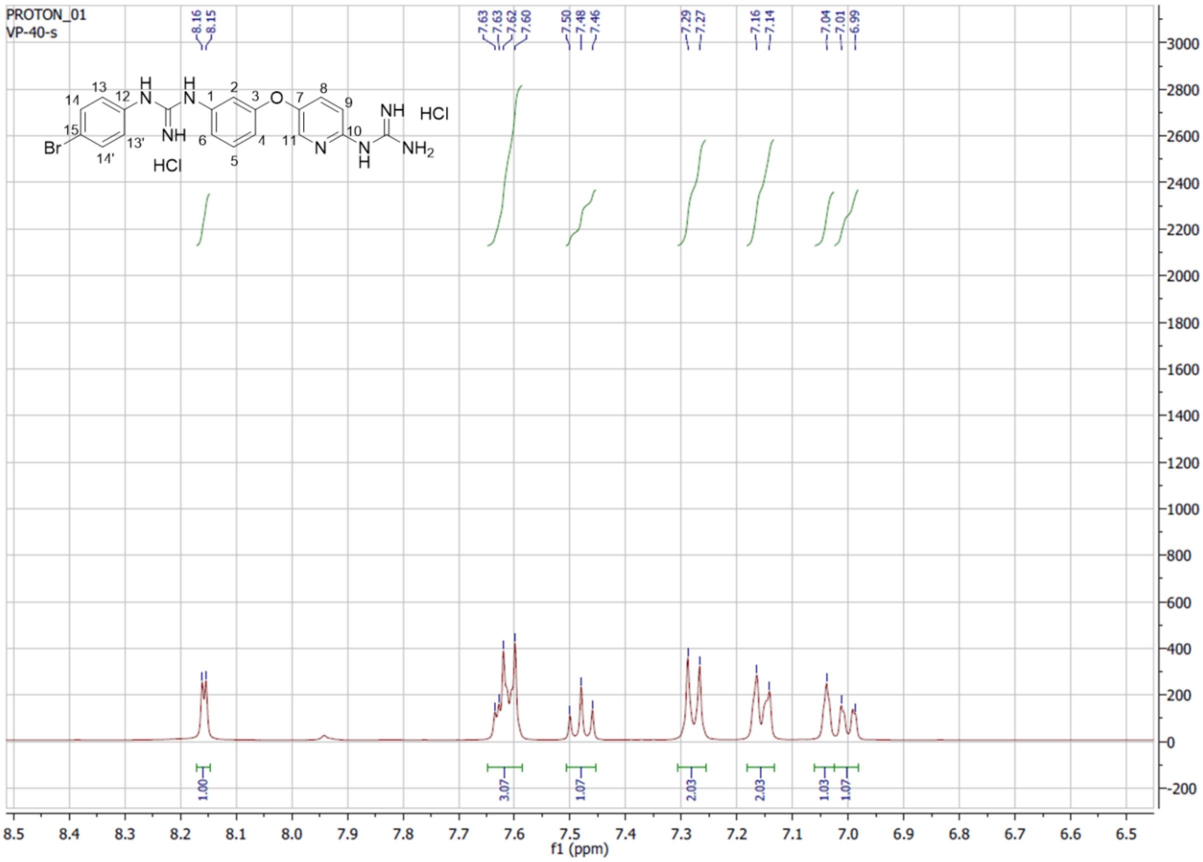

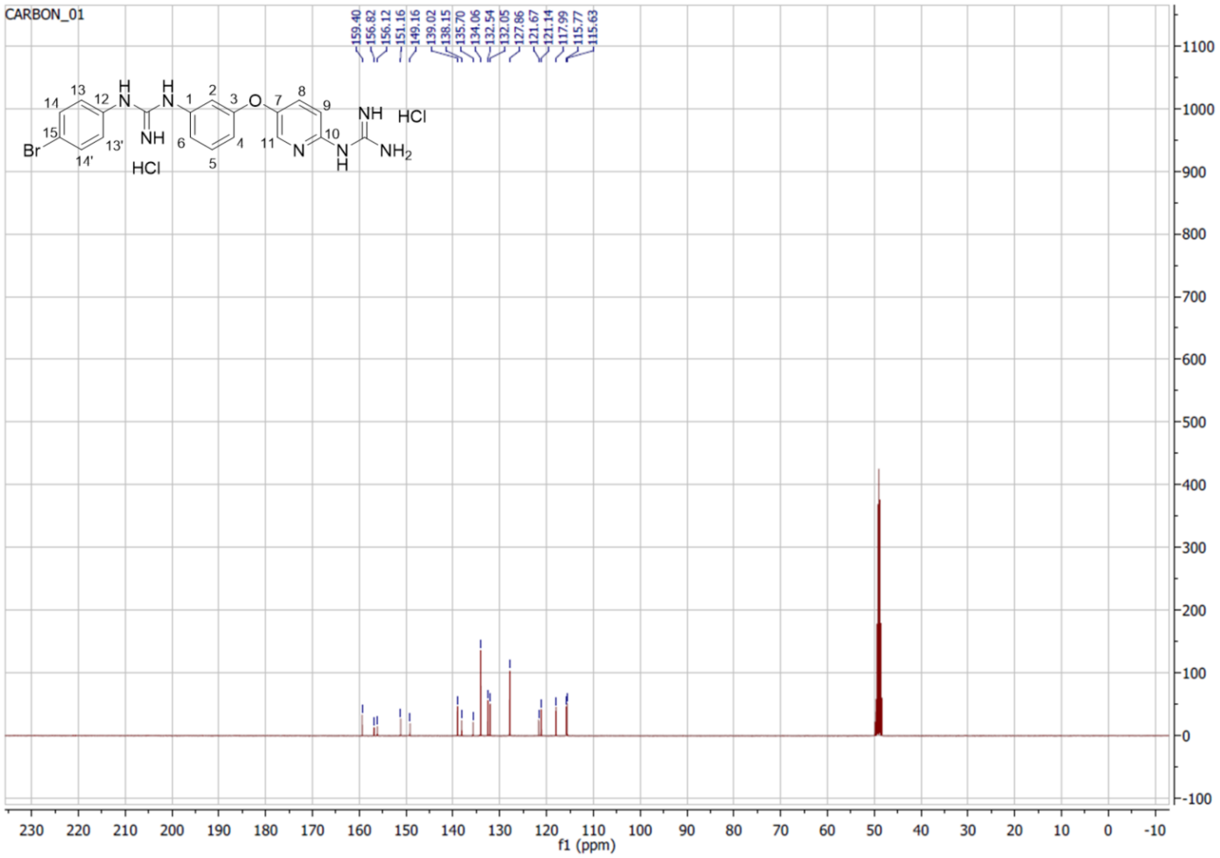
CARBON_01
159.40
156.82
156.12
151.16
149.16
139.02
138.15
135.70
134.06
132.54
132.05
127.86
121.67
121.14
117.99
115.77
115.63
HCl
HCl
f1 (ppm)

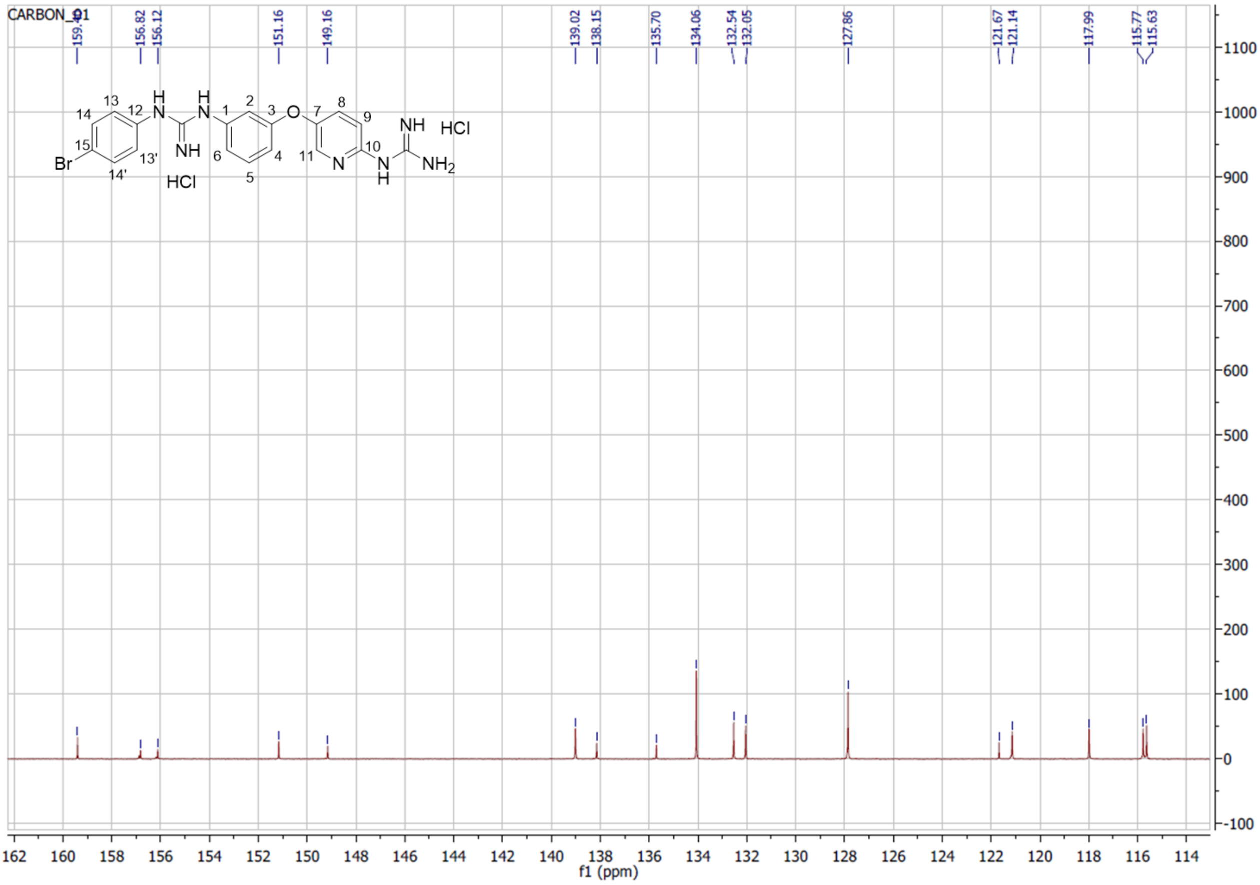
CARBON_01
159.40
156.82
156.12
151.16
149.16
139.02
138.15
135.70
134.06
132.54
132.05
127.86
121.67
121.14
117.99
115.77
115.63
HCl
HCl
f1 (ppm)

^{1}H-NMR, ^{13}C-NMR, and ^{19}F-NMR for compound **52**

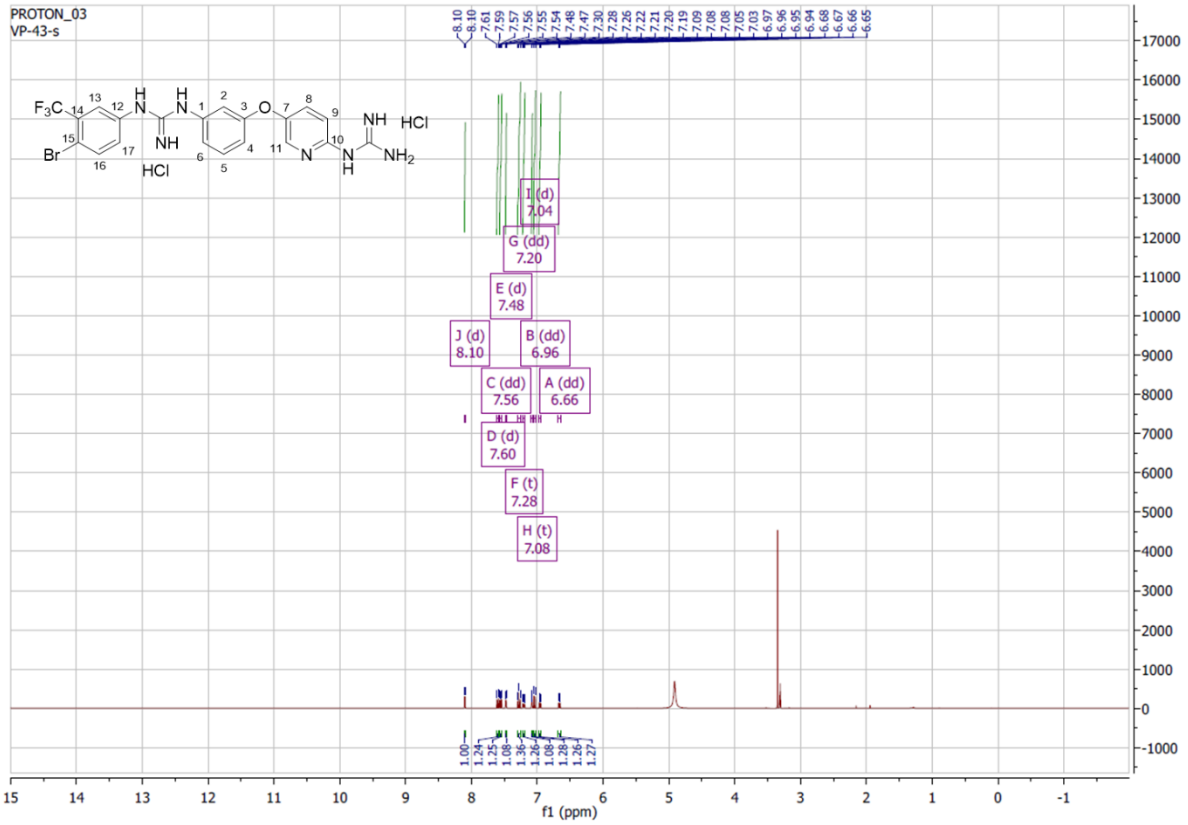

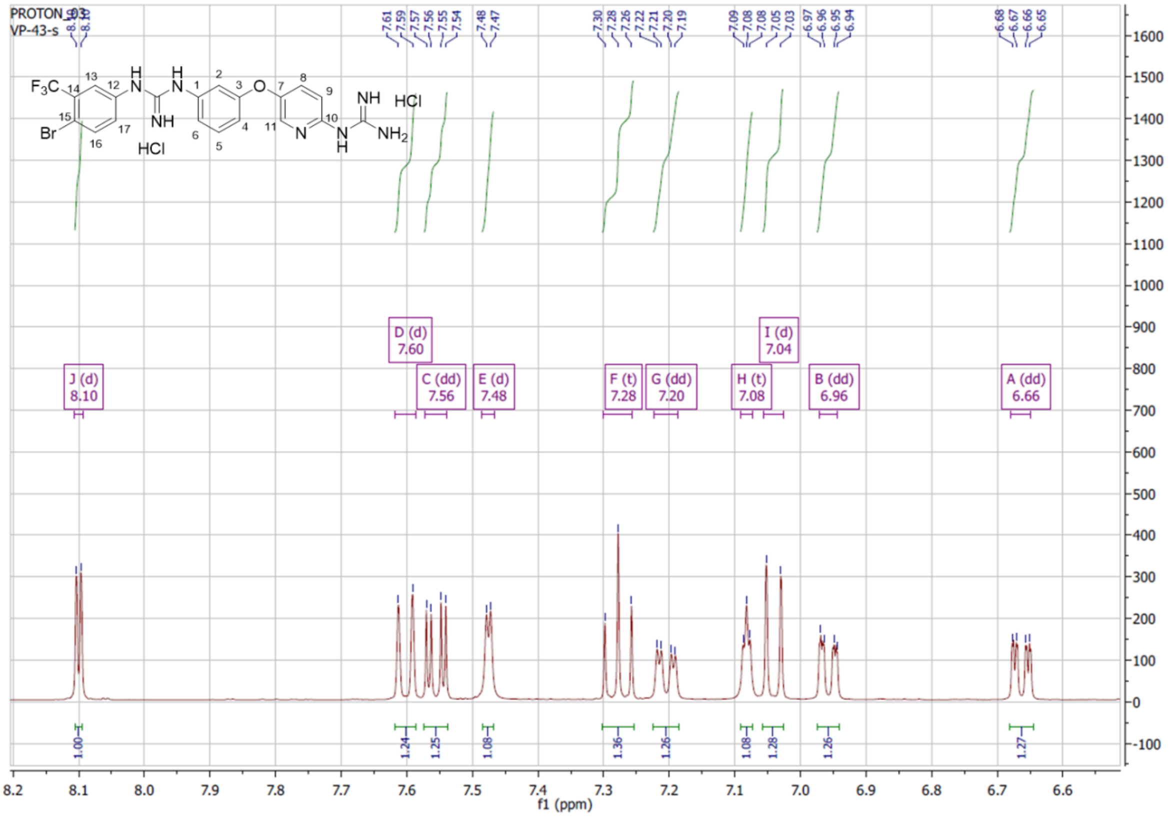

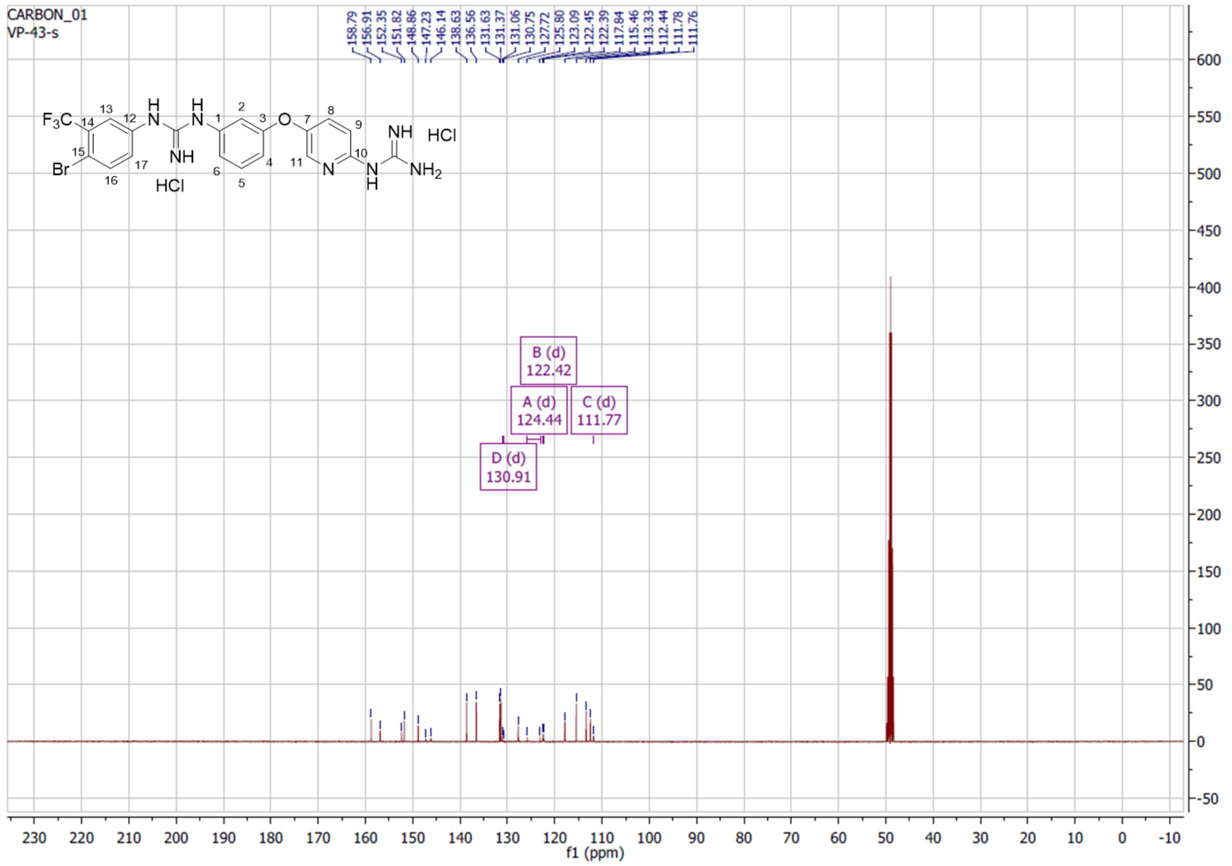

CARBON_01
VP-43-s
158.79
156.91
152.35
151.82
148.86
147.23
146.14
138.63
136.56
131.63
131.37
131.06
130.75
127.72
125.80
123.09
122.45
122.39
117.84
115.46
113.33
112.44
111.78
111.76
HCl
HCl
B (d)
122.42
A (d)
124.44
C (d)
111.77
D (d)
130.91
f1 (ppm)

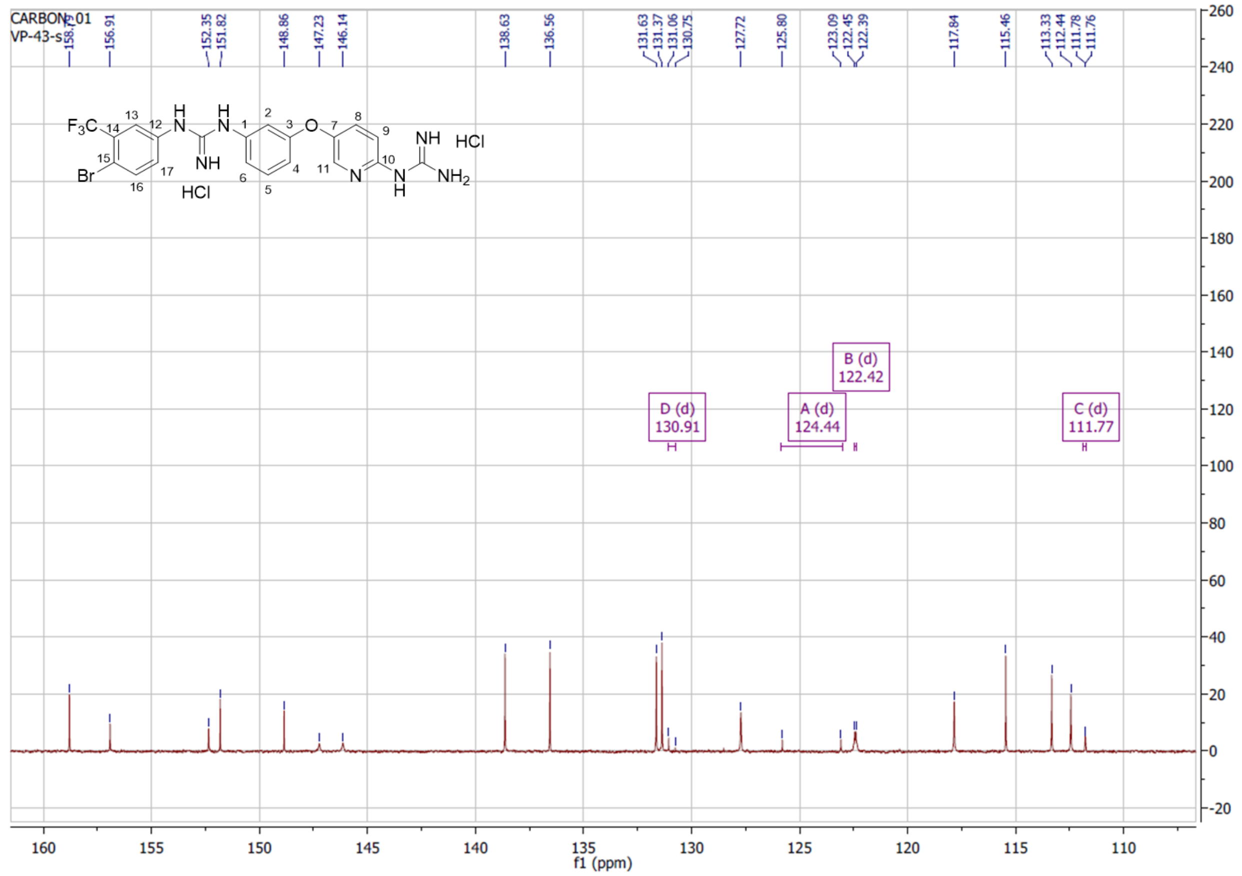

CARBON_01
VP-43-s
156.91
152.35
151.82
148.86
147.23
146.14
138.63
136.56
131.63
131.37
131.06
130.75
127.72
125.80
123.09
122.45
122.39
117.84
115.46
113.33
112.44
111.78
111.76
HCl
HCl
B (d)
122.42
D (d)
130.91
A (d)
124.44
C (d)
111.77
f1 (ppm)

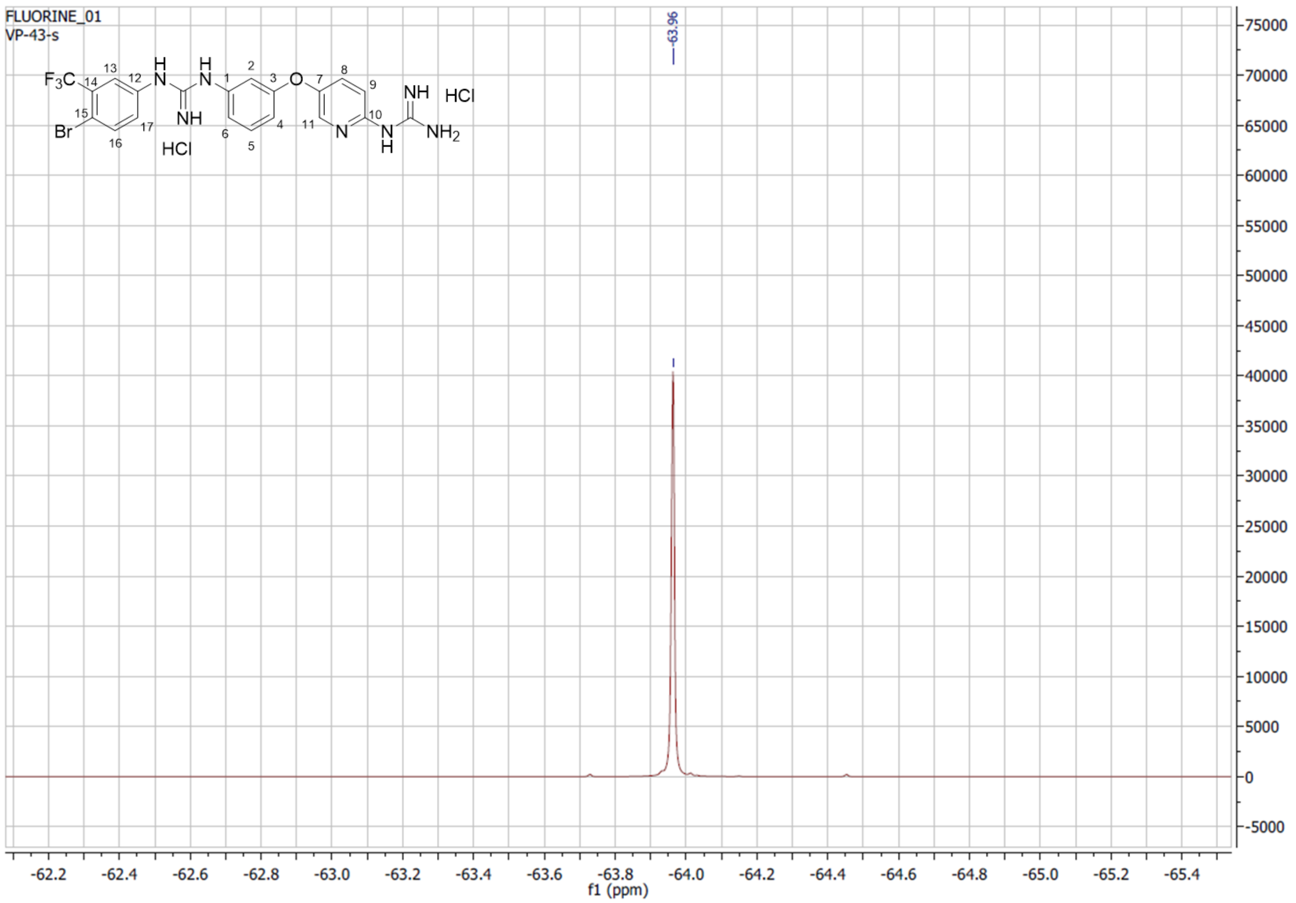
FLUORINE_01
VP-43-s
-63.96
F3C
Br
HCl
NH
NH2
N
O
f1 (ppm)

^{1}H-NMR, ^{13}C-NMR, and ^{19}F-NMR for compound **70**

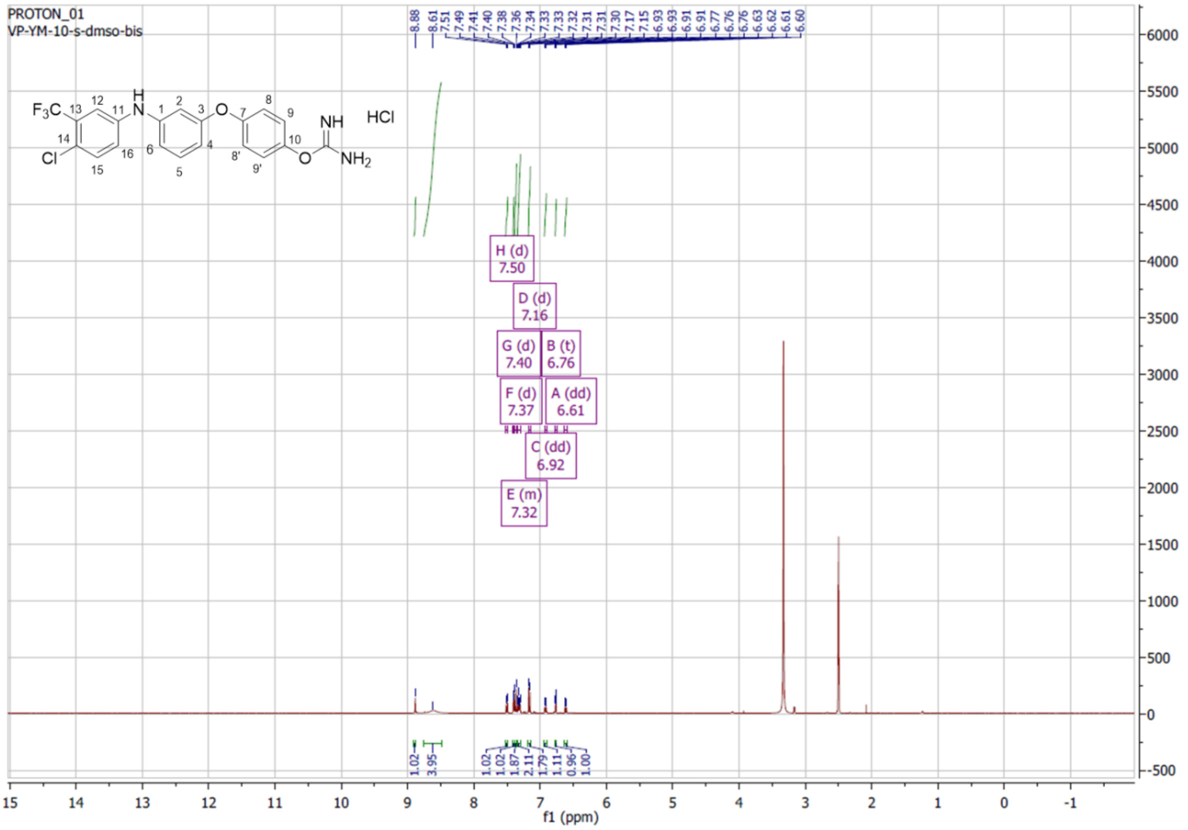

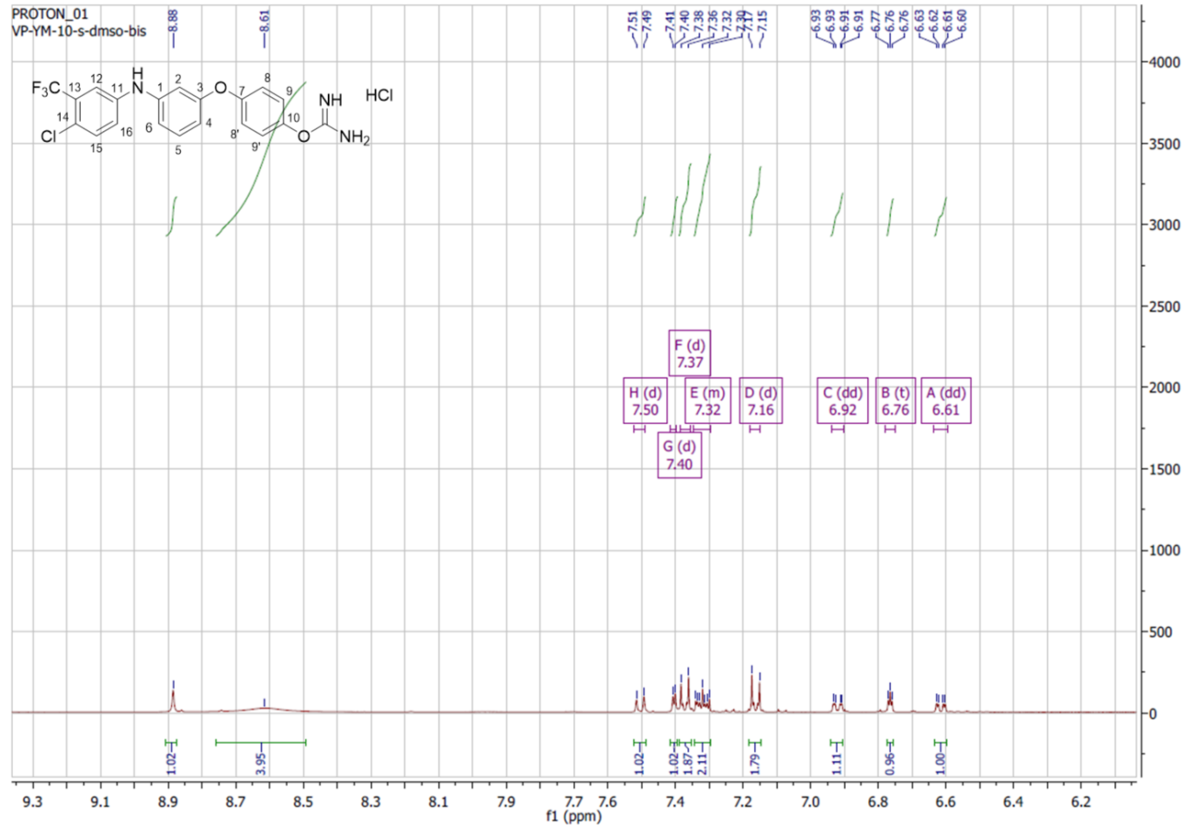

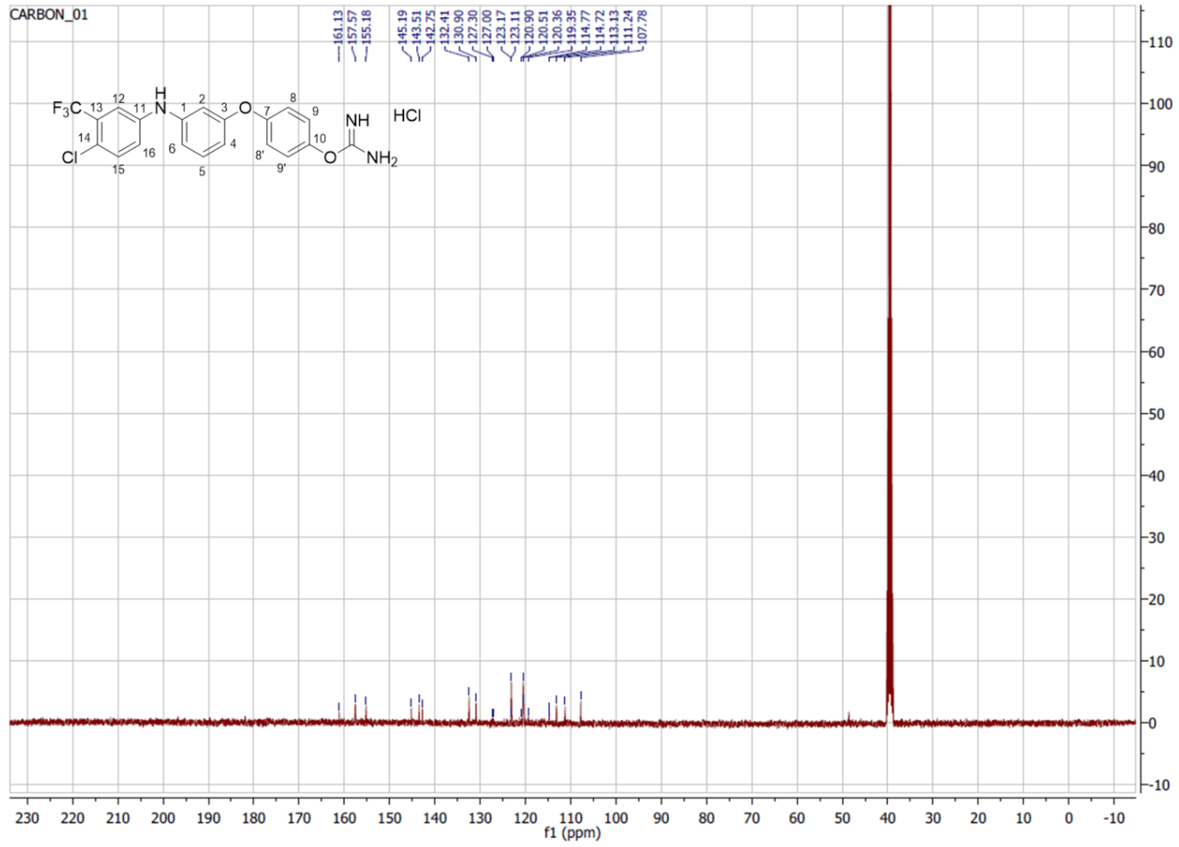
CARBON_01
161.13
157.57
155.18
145.19
143.51
142.75
132.41
130.90
127.30
127.00
123.17
123.11
120.90
120.51
120.36
119.35
114.77
114.72
113.13
111.24
107.78
HCl
f1 (ppm)

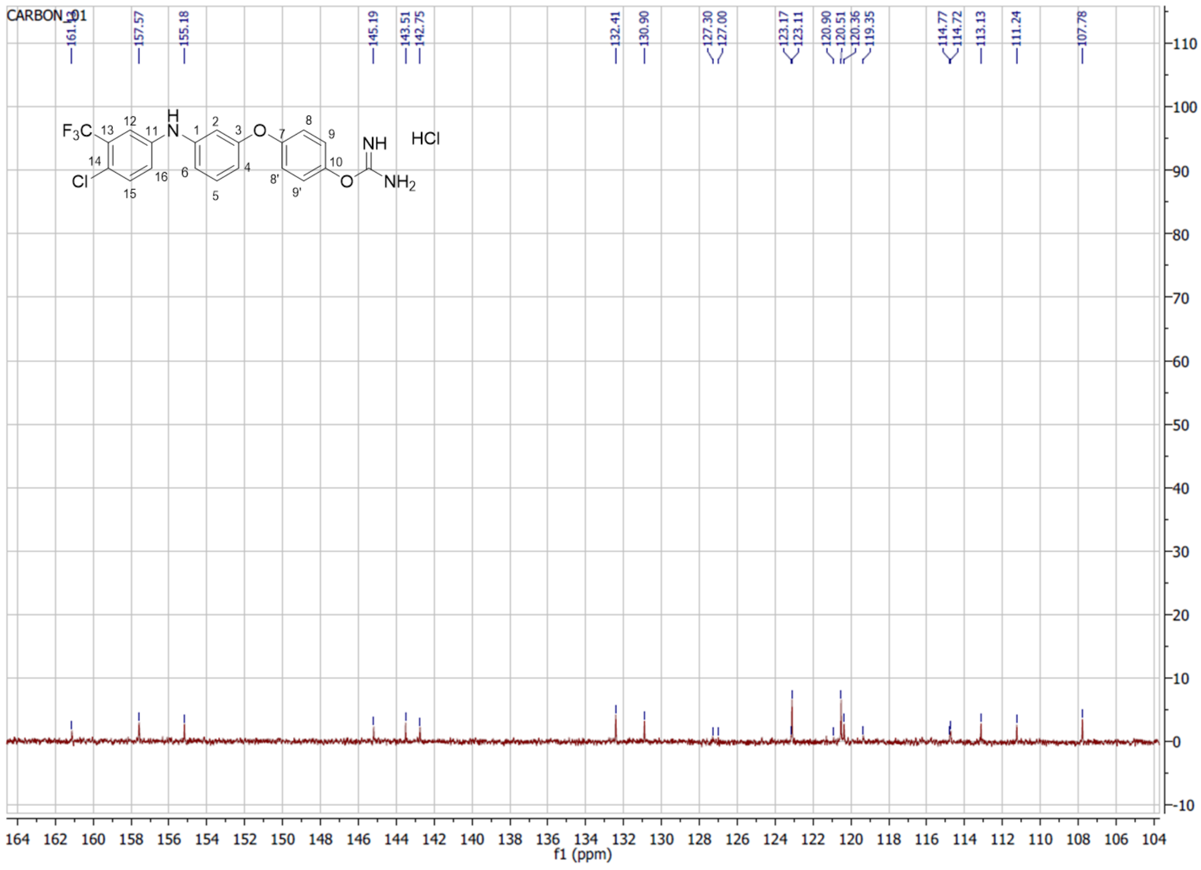
CARBON_01
161.13
157.57
155.18
145.19
143.51
142.75
132.41
130.90
127.30
127.00
123.17
123.11
120.90
120.51
120.36
119.35
114.77
114.72
113.13
111.24
107.78
HCl
f1 (ppm)

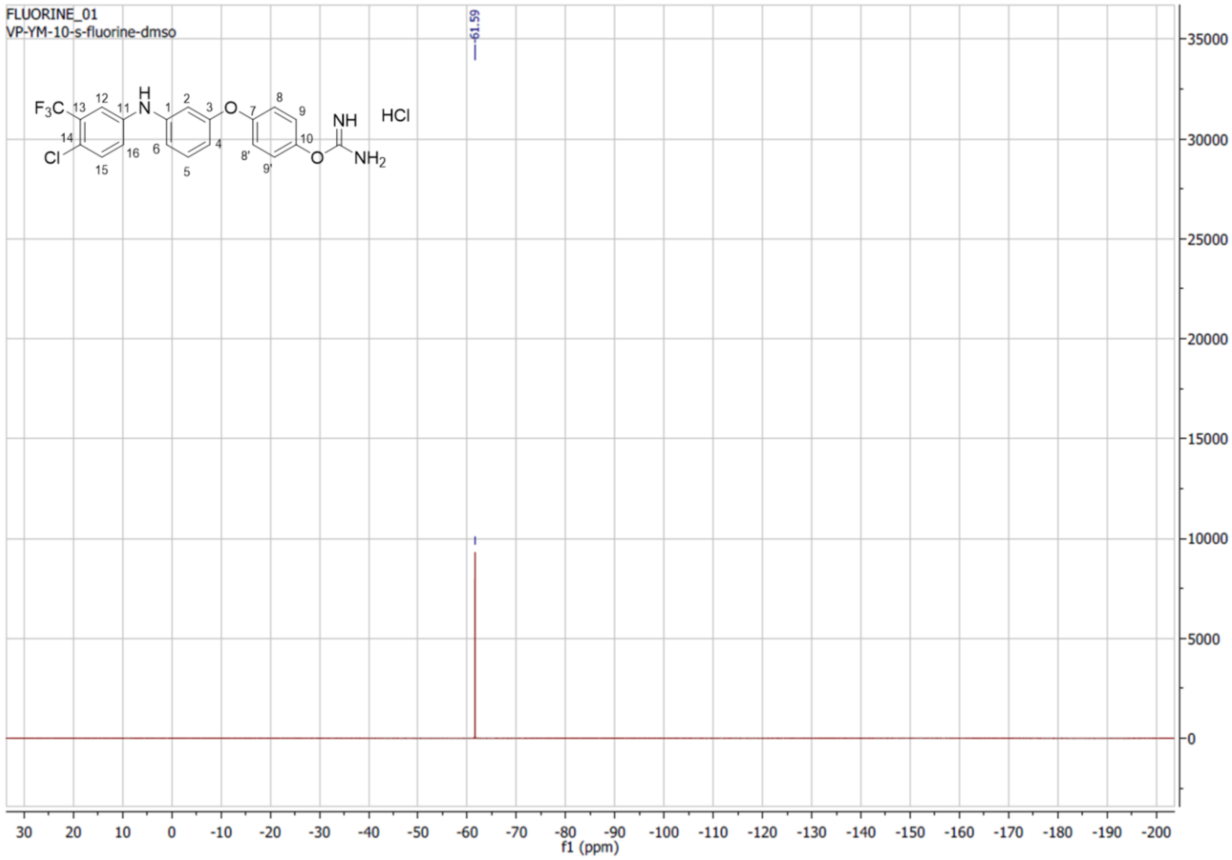
FLUORINE_01
VP-YM-10-s-fluorine-dmso
-61.59
HCl
f1 (ppm)

^{1}H-NMR, ^{13}C-NMR, and ^{19}F-NMR for compound **75**

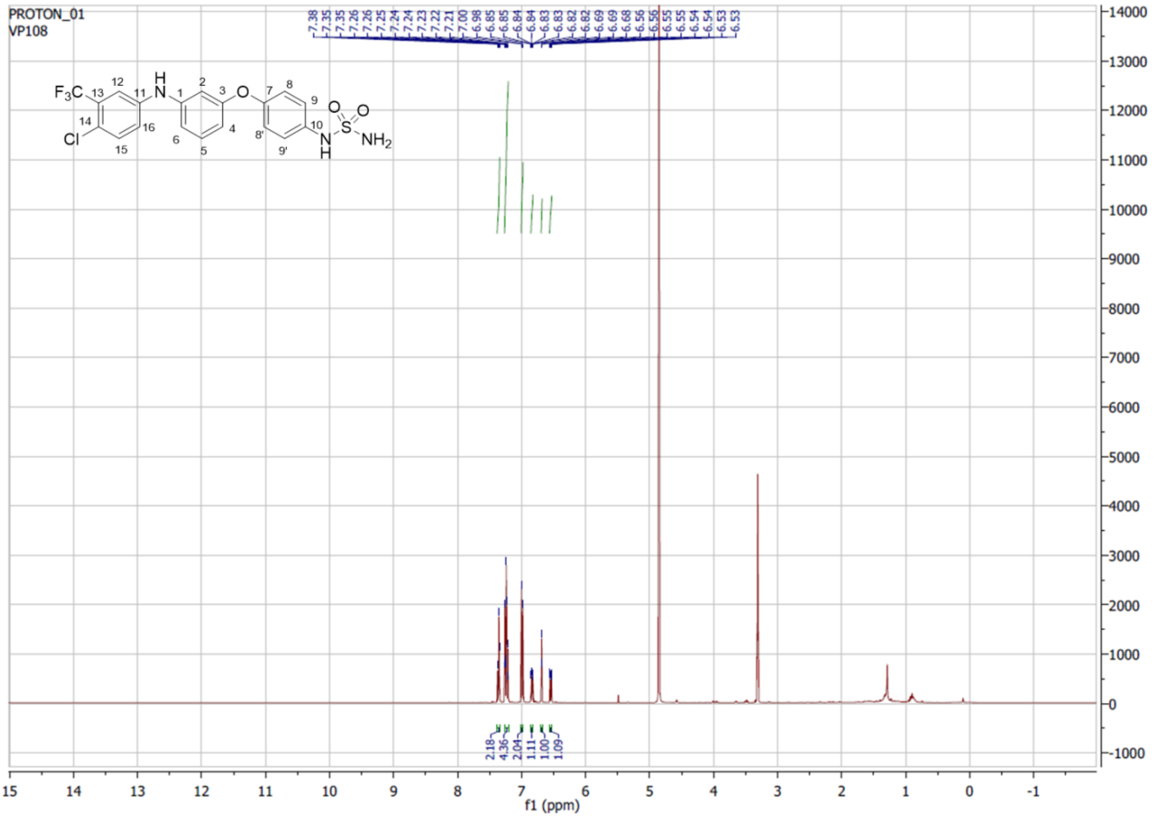

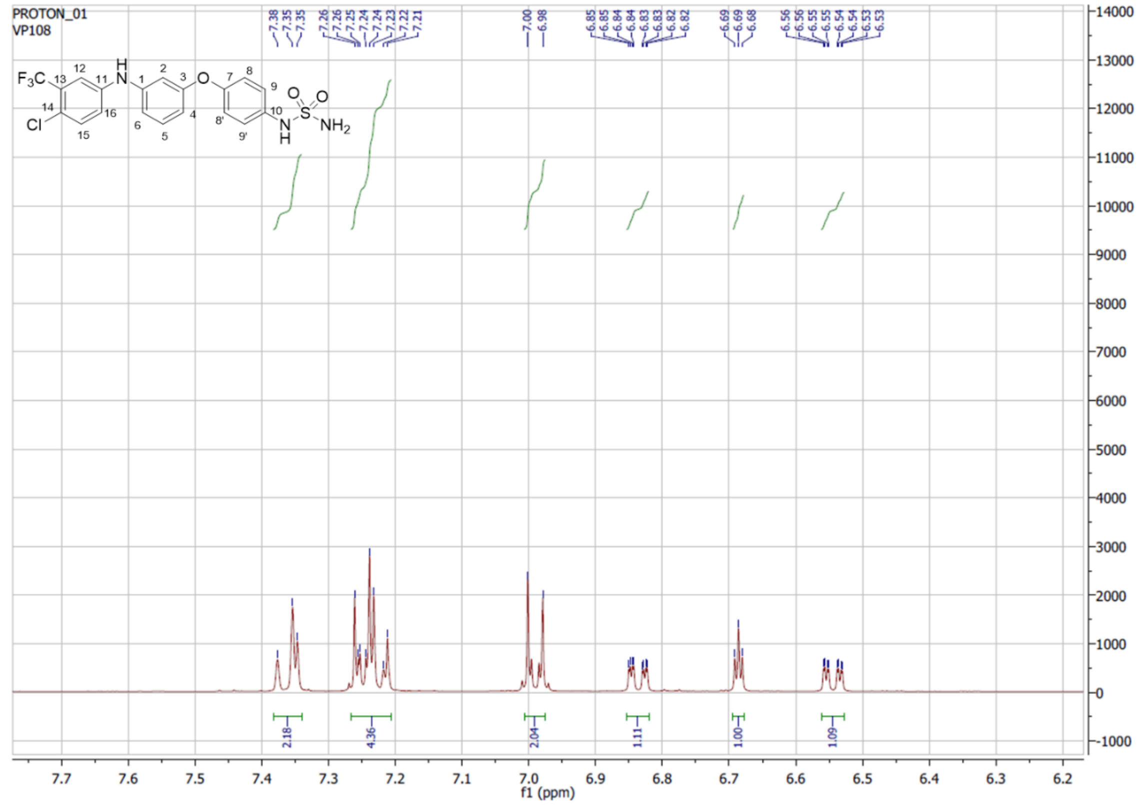

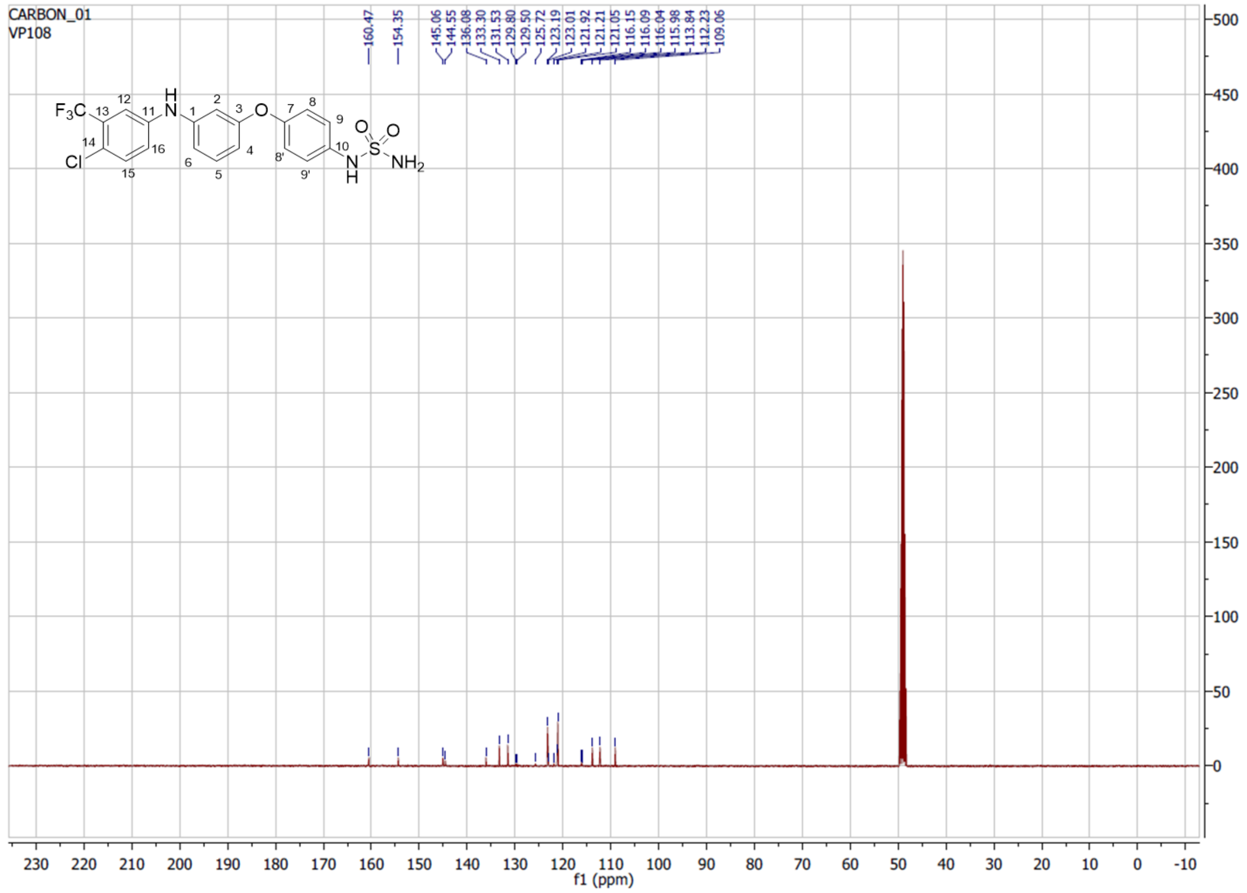
CARBON_01
VP108
160.47
154.35
145.06
144.55
136.08
133.30
131.53
129.80
129.50
125.72
123.19
123.01
121.92
121.21
121.05
116.15
116.09
116.04
115.98
113.84
112.23
109.06
f1 (ppm)

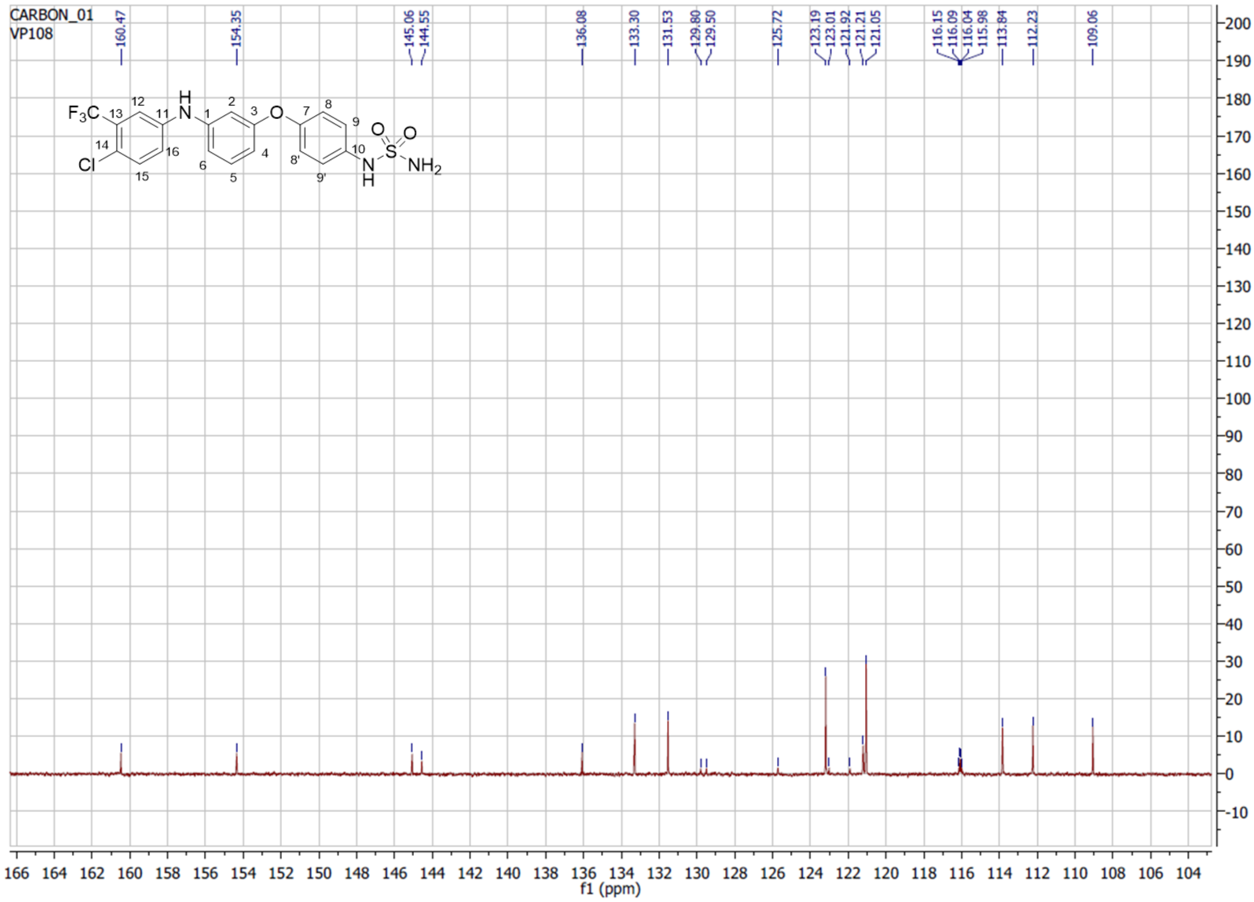
CARBON_01
VP108
160.47
154.35
145.06
144.55
136.08
133.30
131.53
129.80
129.50
125.72
123.19
123.01
121.92
121.21
121.05
116.15
116.09
116.04
115.98
113.84
112.23
109.06
f1 (ppm)

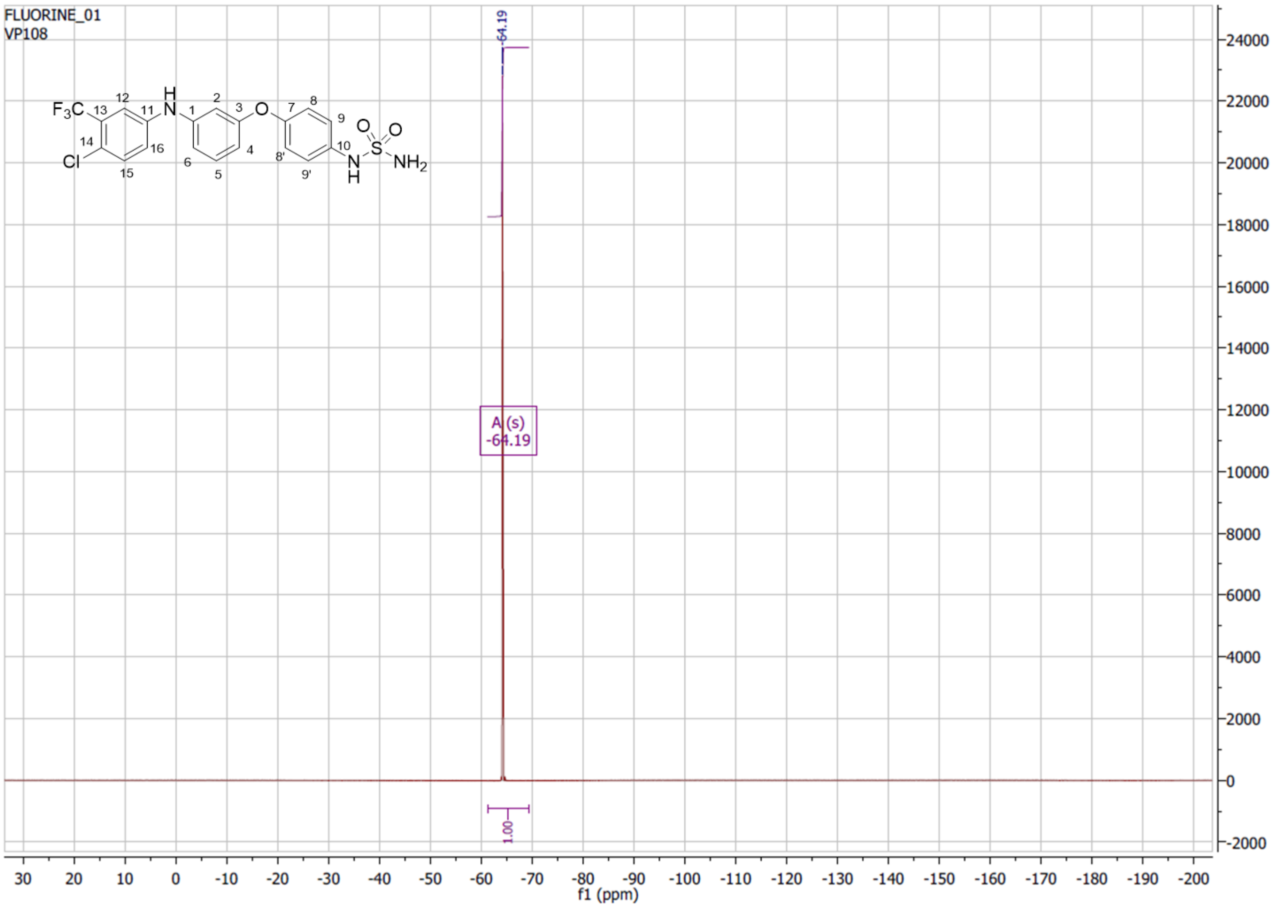
FLUORINE_01
VP108
F3C
Cl
NH2
-64.19
A (s)
-64.19
1.00
f1 (ppm)

6. HPLC Chromatograms of Final Salts

Compound **2**

System : HPLC-PDA
Method : Gradient1
User : Daniel

Acquired : 15/07/2015 12:59:28
Processed : 15/07/2015 13:46:44
Printed : 27/08/2015 08:13:30

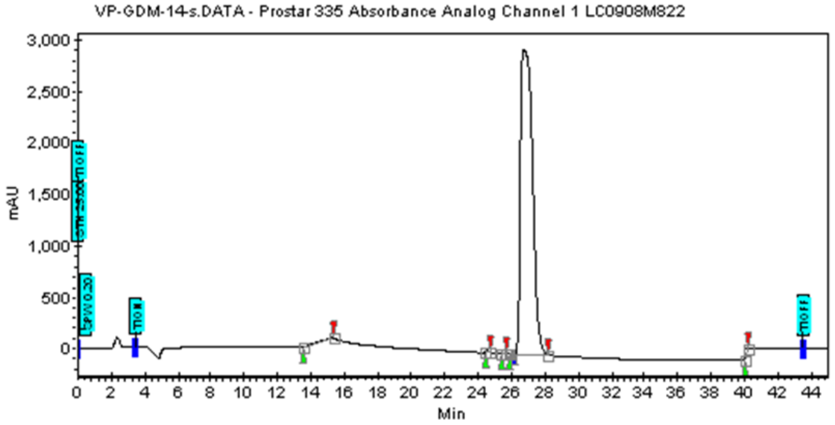

Peak results :

Index	Name	Time [Min]	Quantity [% Area]	Height [mAU]	Area [mAU.Min]	Area % [%]
1	UNKNOWN	14.41	0.37	8.5	9.2	0.366
2	UNKNOWN	24.57	0.12	20.4	2.9	0.116
3	UNKNOWN	25.56	0.03	6.9	0.8	0.031
4	UNKNOWN	26.08	0.08	15.5	2.0	0.081
5	UNKNOWN	26.77	98.88	2969.4	2479.3	98.878
6	UNKNOWN	40.00	0.53	106.3	13.2	0.528
Total			100.00	3127.1	2507.4	100.000

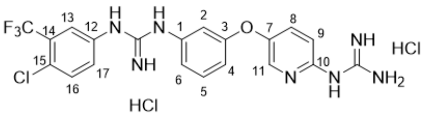

Compound **3**

System : HPLC-PDA
Method : Gradient1
User : Daniel

Acquired : 18/11/2014 13:52:34
Processed : 18/11/2014 14:40:04
Printed : 18/11/2014 17:32:12

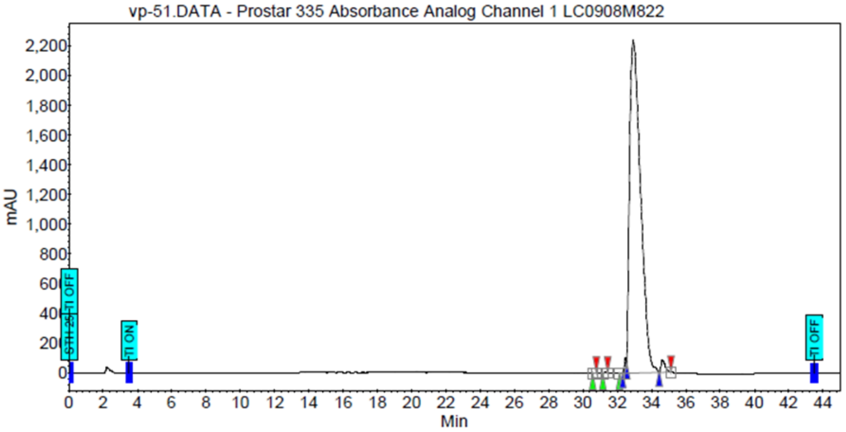

Peak results :

Index	Name	Time [Min]	Quantity [% Area]	Height [mAU]	Area [mAU.Min]	Area % [%]
1	UNKNOWN	30.67	0.02	3.0	0.3	0.019
2	UNKNOWN	31.28	0.06	8.8	0.9	0.055
3	UNKNOWN	32.21	0.02	3.0	0.3	0.019
4	UNKNOWN	32.45	0.80	104.6	13.6	0.796
5	UNKNOWN	32.93	97.80	2238.5	1665.2	97.799
6	UNKNOWN	34.63	1.31	83.5	22.3	1.312
Total			100.00	2441.4	1702.7	100.000

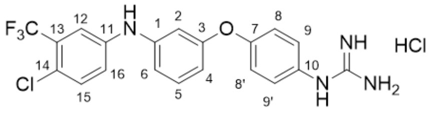

Compound **4**

System : HPLC-PDA
Method : Gradient
User : Daniel

Acquired : 26/05/2015 17:27:57
Processed : 26/05/2015 18:15:26
Printed : 27/08/2015 08:12:37

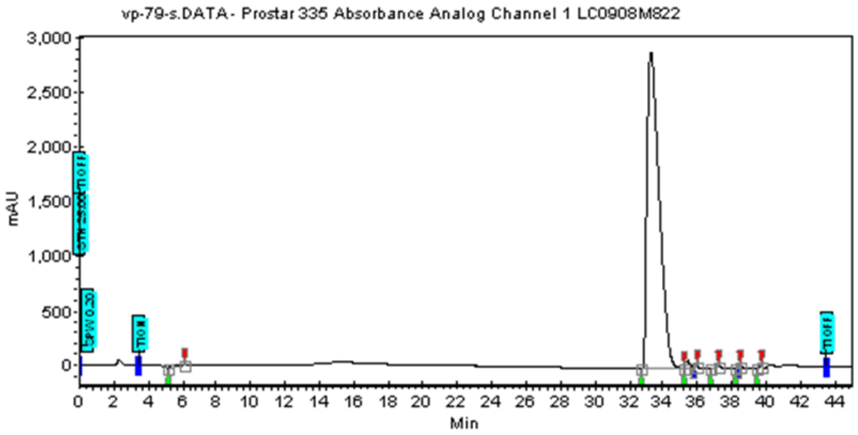

Peak results :

Index	Name	Time [Min]	Quantity [% Area]	Height [mAU]	Area [mAU.Min]	Area % [%]
1	UNKNOWN	5.44	0.40	16.7	9.6	0.403
2	UNKNOWN	33.32	98.60	2899.1	2361.7	98.605
3	UNKNOWN	35.44	0.75	60.7	17.9	0.746
4	UNKNOWN	35.84	0.04	7.0	1.0	0.041
5	UNKNOWN	37.08	0.07	9.8	1.7	0.069
6	UNKNOWN	38.31	0.07	12.8	1.6	0.067
7	UNKNOWN	38.35	0.03	10.7	0.8	0.033
8	UNKNOWN	39.57	0.04	5.7	0.9	0.036
Total			100.00	3022.4	2395.1	100.000

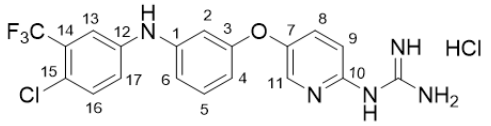

Compound **5**

System : HPLC-PDA
Method : Gradient
User : Daniel

Acquired : 20/07/2015 16:20:49
Processed : 20/07/2015 17:08:18
Printed : 27/08/2015 08:19:48

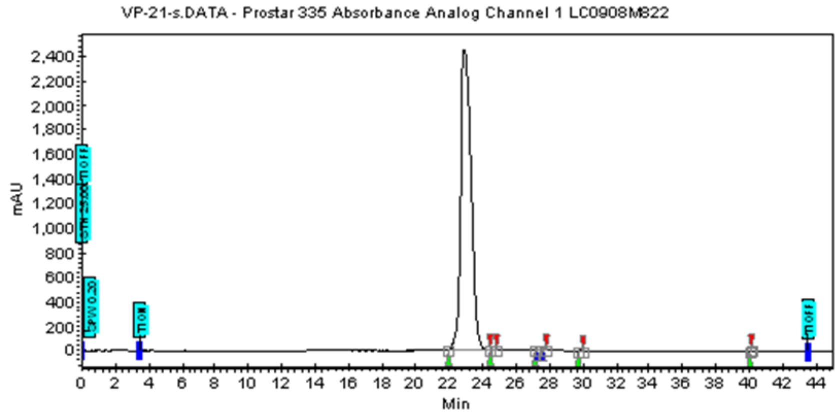

Peak results :

Index	Name	Time [Min]	Quantity [% Area]	Height [mAU]	Area [mAU.Min]	Area % [%]
1	UNKNOWN	22.88	99.74	2455.8	1774.8	99.744
2	UNKNOWN	24.67	0.04	5.3	0.7	0.039
3	UNKNOWN	27.20	0.02	3.3	0.3	0.018
4	UNKNOWN	27.43	0.11	10.2	2.0	0.115
5	UNKNOWN	27.73	0.02	3.2	0.3	0.020
6	UNKNOWN	29.87	0.05	7.0	0.8	0.046
7	UNKNOWN	40.00	0.02	8.6	0.3	0.019
Total			100.00	2493.4	1779.4	100.000

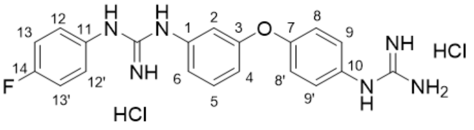

Compound **6**

System : HPLC-PDA
Method : Gradient1
User : Daniel

Acquired : 20/07/2015 15:22:27
Processed : 20/07/2015 16:09:57
Printed : 27/08/2015 08:18:54

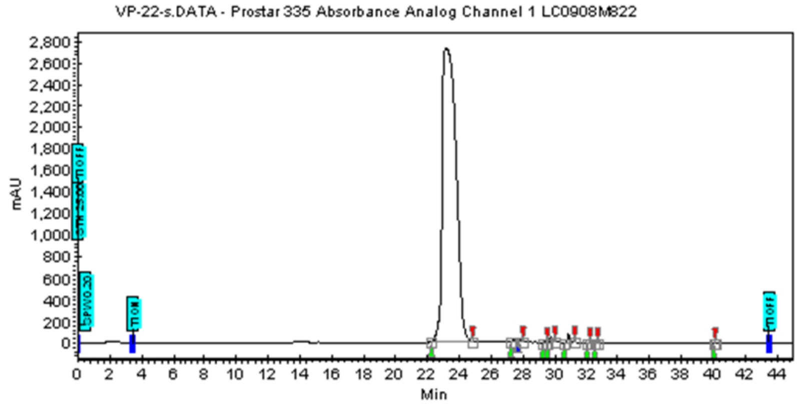

Peak results :

Index	Name	Time [Min]	Quantity [% Area]	Height [mAU]	Area [mAU.Min]	Area % [%]
1	UNKNOWN	23.15	98.75	2743.8	2745.1	98.755
2	UNKNOWN	27.44	0.14	26.7	3.9	0.142
3	UNKNOWN	27.69	0.04	10.4	1.2	0.045
4	UNKNOWN	27.84	0.12	22.3	3.4	0.123
5	UNKNOWN	29.40	0.02	5.7	0.6	0.022
6	UNKNOWN	29.75	0.29	55.0	8.1	0.292
7	UNKNOWN	30.83	0.58	89.5	16.2	0.581
8	UNKNOWN	32.11	0.01	3.2	0.4	0.013
9	UNKNOWN	32.59	0.01	2.9	0.3	0.011
10	UNKNOWN	40.00	0.02	8.5	0.5	0.017
Total			100.00	2967.9	2779.8	100.000

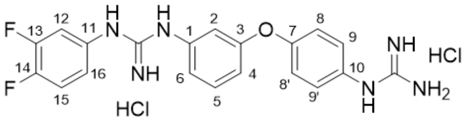

Compound **7**

System : HPLC-PDA
Method : Gradient1
User : Daniel

Acquired : 28/07/2015 15:13:36
Processed : 28/07/2015 16:01:05
Printed : 28/07/2015 16:15:01

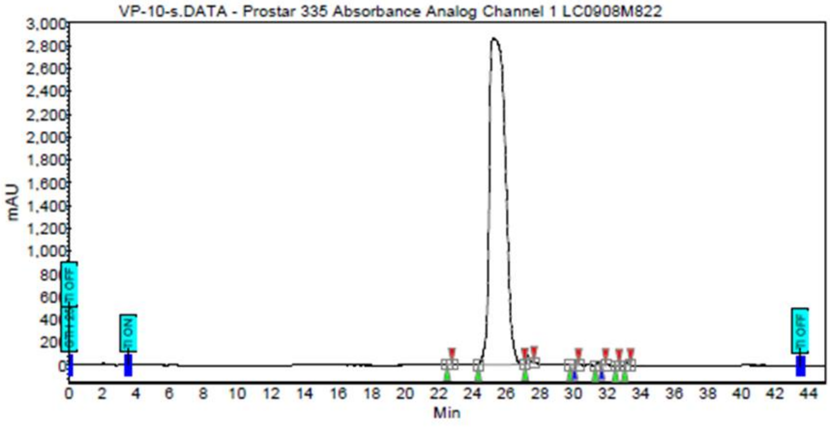

Peak results :

Index	Name	Time [Min]	Quantity [% Area]	Height [mAU]	Area [mAU.Min]	Area % [%]
1	UNKNOWN	22.61	0.02	5.6	0.7	0.023
2	UNKNOWN	25.25	99.18	2857.1	3100.9	99.182
3	UNKNOWN	27.29	0.49	73.5	15.2	0.486
4	UNKNOWN	29.95	0.02	5.7	0.7	0.023
5	UNKNOWN	30.20	0.01	3.4	0.3	0.011
6	UNKNOWN	31.44	0.10	21.5	3.2	0.104
7	UNKNOWN	31.75	0.03	6.3	0.9	0.029
8	UNKNOWN	32.61	0.01	3.0	0.4	0.012
9	UNKNOWN	33.17	0.13	26.2	4.1	0.130
Total			100.00	3002.3	3126.4	100.000

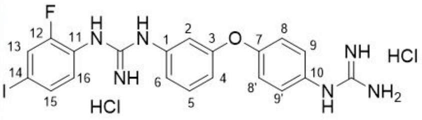

Compound **8**

System : HPLC-PDA
Method : Gradient1
User : Daniel

Acquired : 28/07/2015 14:19:22
Processed : 28/07/2015 15:06:51
Printed : 27/08/2015 08:18:08

VP-ECP-11-s-2.DATA - Prostar 335 Absorbance Analog Channel 1 LC0908M822

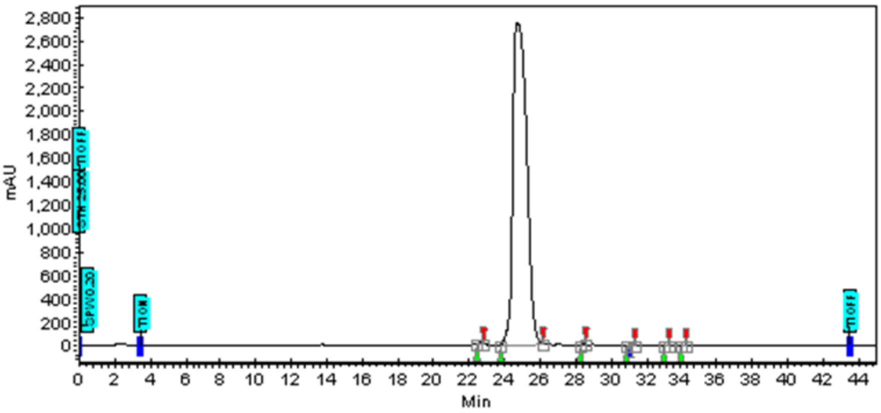

Peak results :

Index	Name	Time [Min]	Quantity [% Area]	Height [mAU]	Area [mAU.Min]	Area % [%]
1	UNKNOWN	22.63	0.10	16.2	2.4	0.096
2	UNKNOWN	24.75	99.75	2758.4	2471.0	99.748
3	UNKNOWN	28.43	0.03	5.1	0.6	0.025
4	UNKNOWN	30.99	0.02	2.9	0.4	0.016
5	UNKNOWN	31.23	0.04	5.7	0.9	0.038
6	UNKNOWN	33.17	0.03	5.9	0.8	0.031
7	UNKNOWN	34.07	0.05	7.4	1.1	0.046
Total			100.00	2801.6	2477.3	100.000

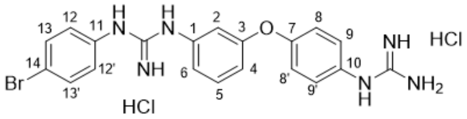

Compound **9**

System : HPLC-PDA
Method : Gradient1
User : Daniel

Acquired : 28/07/2015 16:08:06
Processed : 28/07/2015 16:55:23
Printed : 27/08/2015 08:16:53

VP-8-s.DATA - Prostar 335 Absorbance Analog Channel 1 LC0908M822

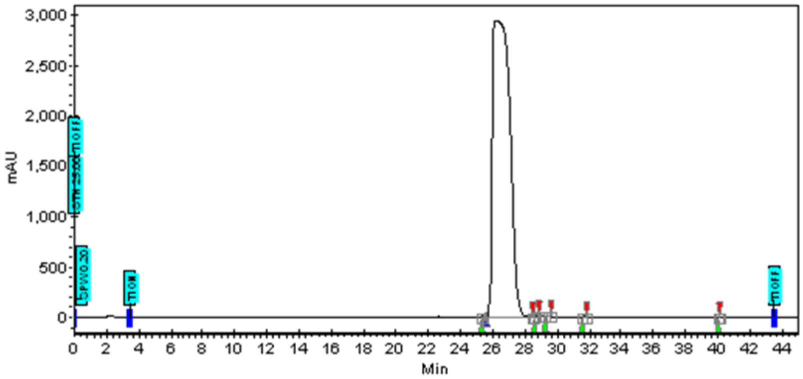

Peak results :

Index	Name	Time [Min]	Quantity [% Area]	Height [mAU]	Area [mAU.Min]	Area % [%]
1	UNKNOWN	25.44	0.02	5.0	0.6	0.016
2	UNKNOWN	25.60	0.02	7.7	0.7	0.020
3	UNKNOWN	26.27	99.87	2939.1	3640.8	99.874
4	UNKNOWN	28.69	0.02	5.0	0.7	0.020
5	UNKNOWN	29.44	0.04	8.6	1.3	0.035
6	UNKNOWN	31.72	0.02	6.2	0.8	0.022
7	UNKNOWN	40.00	0.01	7.1	0.4	0.012
Total			100.00	2978.7	3645.4	100.000

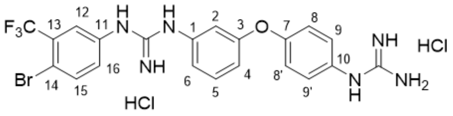

Compound **10**

System : HPLC-PDA
Method : Gradient1
User : Daniel

Acquired : 27/08/2015 14:26:25
Processed : 27/08/2015 15:13:53
Printed : 27/08/2015 15:35:32

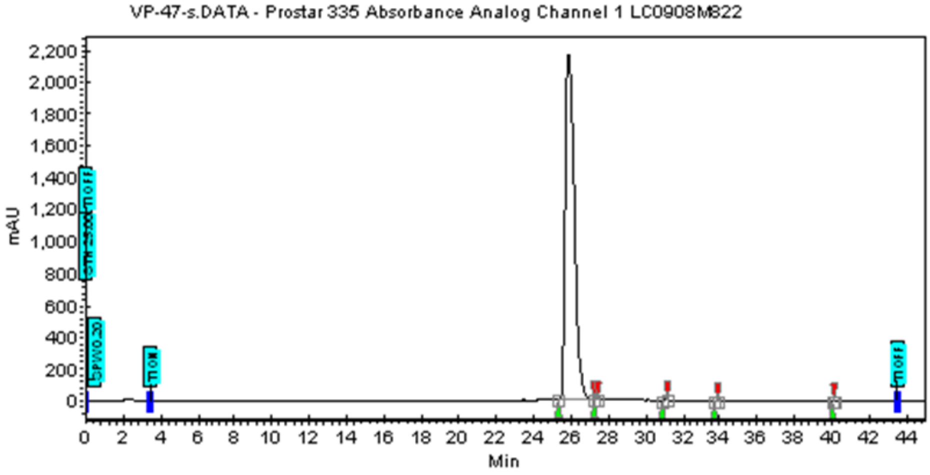

Peak results :

Index	Name	Time [Min]	Quantity [% Area]	Height [mAU]	Area [mAU.Min]	Area % [%]
1	UNKNOWN	25.87	99.89	2172.5	1239.1	99.893
2	UNKNOWN	27.37	0.02	2.6	0.3	0.022
3	UNKNOWN	31.05	0.06	6.6	0.8	0.064
4	UNKNOWN	33.79	0.02	2.8	0.2	0.019
5	UNKNOWN	40.07	0.00	0.5	0.0	0.002
Total			100.00	2185.1	1240.4	100.000

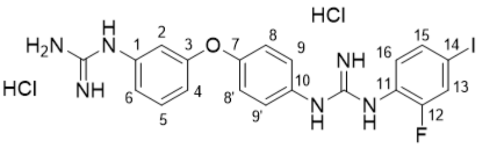

Compound **11**

System : HPLC-PDA
Method : Gradient1
User : Daniel

Acquired : 27/08/2015 13:29:11
Processed : 27/08/2015 14:16:41
Printed : 27/08/2015 14:39:20

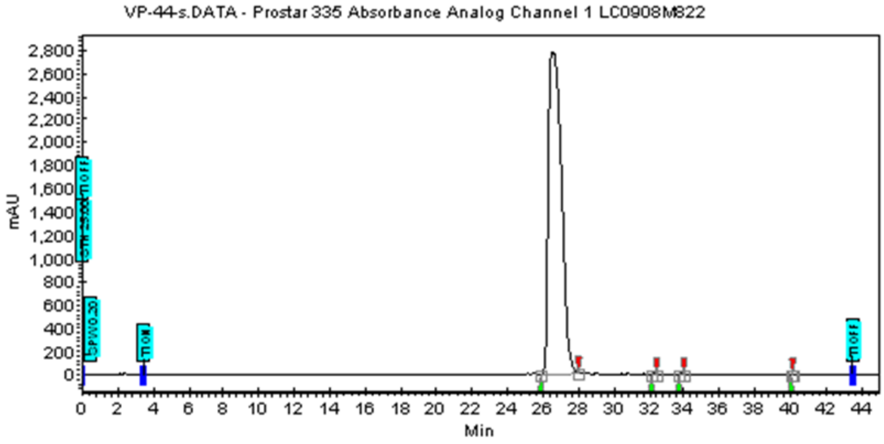

Peak results :

Index	Name	Time [Min]	Quantity [% Area]	Height [mAU]	Area [mAU.Min]	Area % [%]
1	UNKNOWN	26.57	99.86	2782.6	2370.4	99.856
2	UNKNOWN	32.24	0.05	10.2	1.2	0.050
3	UNKNOWN	33.76	0.08	14.9	1.8	0.078
4	UNKNOWN	40.00	0.02	5.7	0.4	0.016
Total			100.00	2813.3	2373.8	100.000

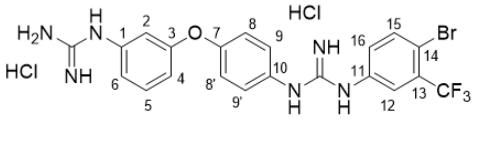

Compound **28**

System : HPLC-PDA
Method : Gradienti
User : Daniel

Acquired : 26/05/2015 12:29:56
Processed : 26/05/2015 13:17:24
Printed : 27/08/2015 08:09:02

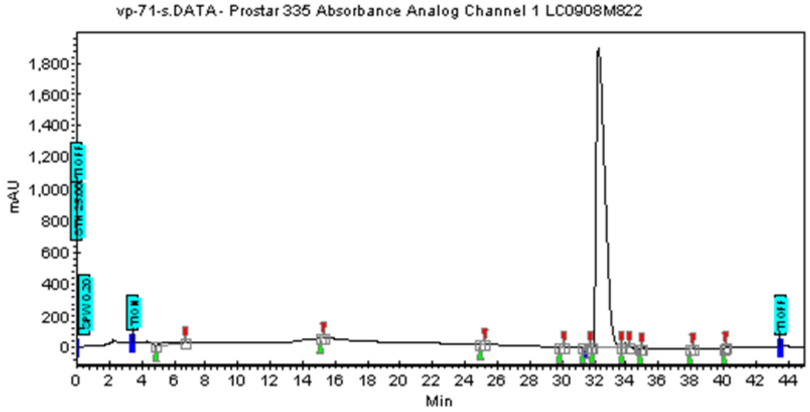

Peak results :

Index	Name	Time [Min]	Quantity [% Area]	Height [mAU]	Area [mAU.Min]	Area % [%]
1	UNKNOWN	5.25	1.26	17.2	16.8	1.258
2	UNKNOWN	15.09	0.01	0.7	0.1	0.006
3	UNKNOWN	25.05	0.03	4.3	0.5	0.034
4	UNKNOWN	29.99	0.02	3.1	0.3	0.024
5	UNKNOWN	31.39	0.04	4.6	0.5	0.035
6	UNKNOWN	31.57	0.03	3.1	0.4	0.031
7	UNKNOWN	32.31	98.02	1902.8	1311.7	98.015
8	UNKNOWN	33.91	0.51	33.8	6.8	0.511
9	UNKNOWN	34.89	0.01	1.4	0.1	0.007
10	UNKNOWN	38.04	0.03	2.7	0.3	0.026
11	UNKNOWN	40.00	0.05	11.5	0.7	0.053
Total			100.00	1985.3	1338.3	100.000

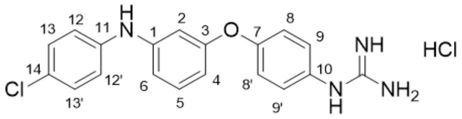

Compound **29**

System : HPLC-PDA
Method : Gradienti
User : Daniel

Acquired : 26/05/2015 15:01:20
Processed : 26/05/2015 15:48:49
Printed : 27/08/2015 08:09:51

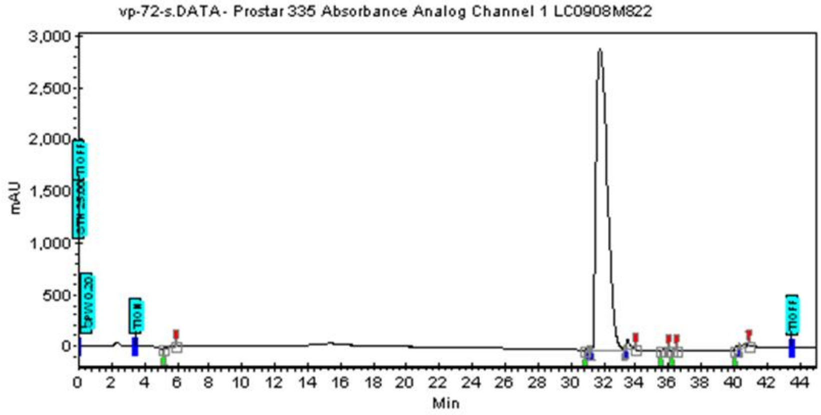

Peak results :

Index	Name	Time [Min]	Quantity [% Area]	Height [mAU]	Area [mAU.Min]	Area % [%]
1	UNKNOWN	5.39	0.29	15.1	7.2	0.287
2	UNKNOWN	31.01	0.04	7.3	1.0	0.038
3	UNKNOWN	31.20	0.02	4.9	0.5	0.020
4	UNKNOWN	31.76	97.52	2908.0	2432.7	97.517
5	UNKNOWN	33.36	0.05	12.8	1.3	0.051
6	UNKNOWN	33.51	0.91	106.8	22.7	0.908
7	UNKNOWN	35.71	0.10	13.9	2.6	0.102
8	UNKNOWN	36.31	0.08	11.3	2.1	0.083
9	UNKNOWN	40.00	0.42	42.1	10.4	0.416
10	UNKNOWN	40.53	0.58	34.8	14.4	0.577
Total			100.00	3157.1	2494.6	100.000

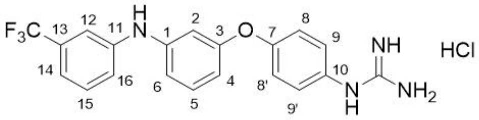

Compound **30**

System : HPLC-PDA
Method : Gradient1
User : Daniel

Acquired : 28/07/2015 13:24:02
Processed : 28/07/2015 14:11:31
Printed : 27/08/2015 08:11:47

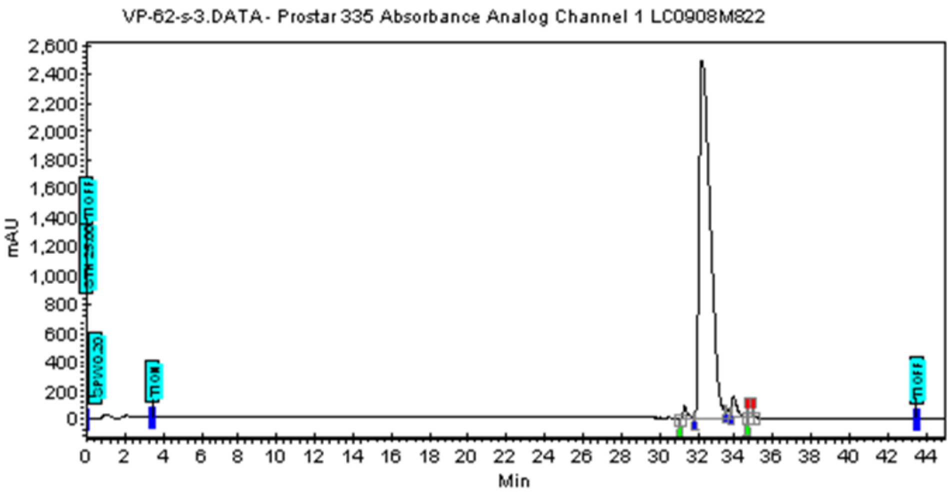

Peak results :

Index	Name	Time [Min]	Quantity [% Area]	Height [mAU]	Area [mAU.Min]	Area % [%]
1	UNKNOWN	31.32	1.07	81.8	20.7	1.067
2	UNKNOWN	32.29	95.37	2490.6	1853.2	95.372
3	UNKNOWN	33.56	0.89	65.4	17.4	0.893
4	UNKNOWN	33.93	2.64	163.6	51.3	2.641
5	UNKNOWN	34.79	0.03	4.5	0.5	0.027
Total			100.00	2805.9	1943.1	100.000

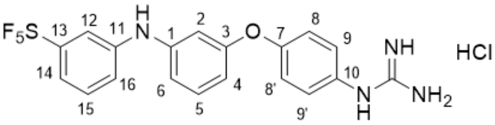

Compound **36**

System : HPLC-PDA
Method : Gradient1
User : Daniel

Acquired : 26/05/2015 16:14:54
Processed : 26/05/2015 17:02:25
Printed : 27/08/2015 08:07:26

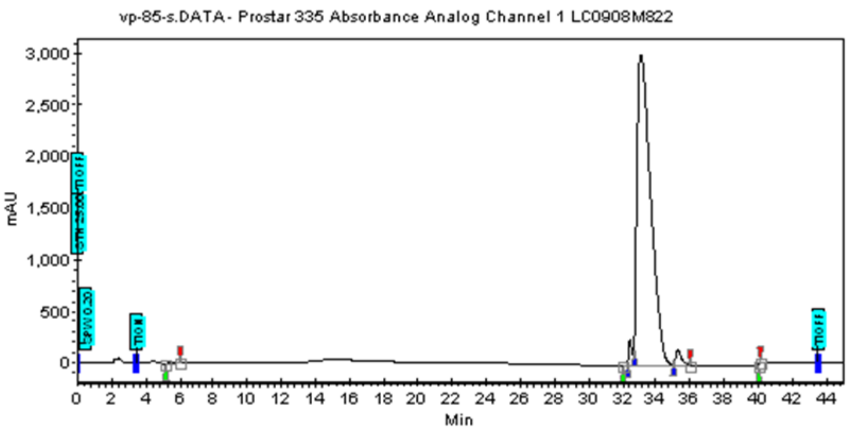

Peak results :

Index	Name	Time [Min]	Quantity [% Area]	Height [mAU]	Area [mAU.Min]	Area % [%]
1	UNKNOWN	5.45	0.25	14.8	7.4	0.252
2	UNKNOWN	32.17	0.03	4.9	0.8	0.028
3	UNKNOWN	32.47	1.98	247.4	58.4	1.985
4	UNKNOWN	33.13	95.67	3027.7	2814.3	95.674
5	UNKNOWN	35.27	1.96	146.9	57.6	1.960
6	UNKNOWN	40.00	0.10	36.1	3.0	0.101
Total			100.00	3477.8	2941.5	100.000

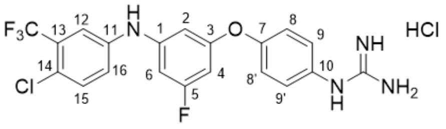

Compound **49**

System : HPLC-PDA
Method : Gradient1
User : Daniel

Acquired : 20/07/2015 10:32:44
Processed : 20/07/2015 11:20:13
Printed : 27/08/2015 08:16:04

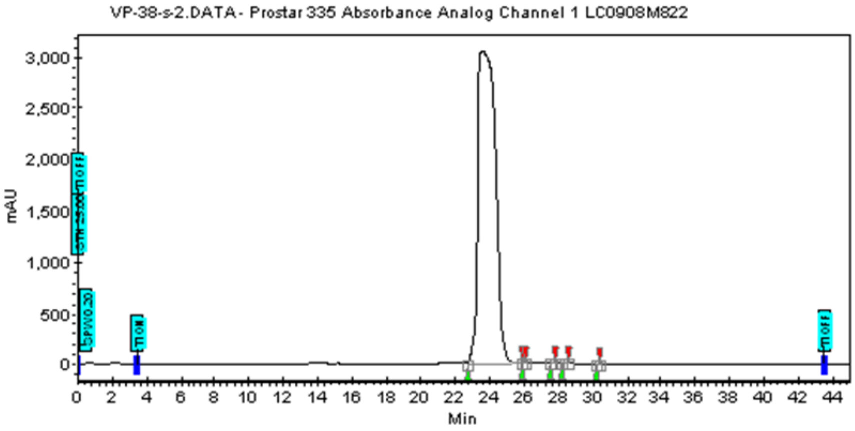

Peak results :

Index	Name	Time [Min]	Quantity [% Area]	Height [mAU]	Area [mAU.Min]	Area % [%]
1	UNKNOWN	23.56	99.84	3050.8	3585.9	99.841
2	UNKNOWN	26.00	0.02	8.0	0.9	0.024
3	UNKNOWN	27.71	0.02	6.6	0.8	0.021
4	UNKNOWN	28.31	0.10	21.9	3.7	0.103
5	UNKNOWN	30.31	0.01	4.3	0.4	0.011
Total			100.00	3091.7	3591.7	100.000

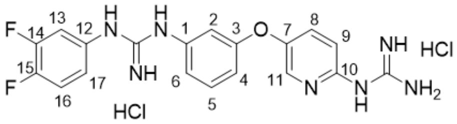

Compound **50**

System : HPLC-PDA
Method : Gradient1
User : Daniel

Acquired : 15/07/2015 12:01:08
Processed : 15/07/2015 12:48:36
Printed : 15/07/2015 13:14:28

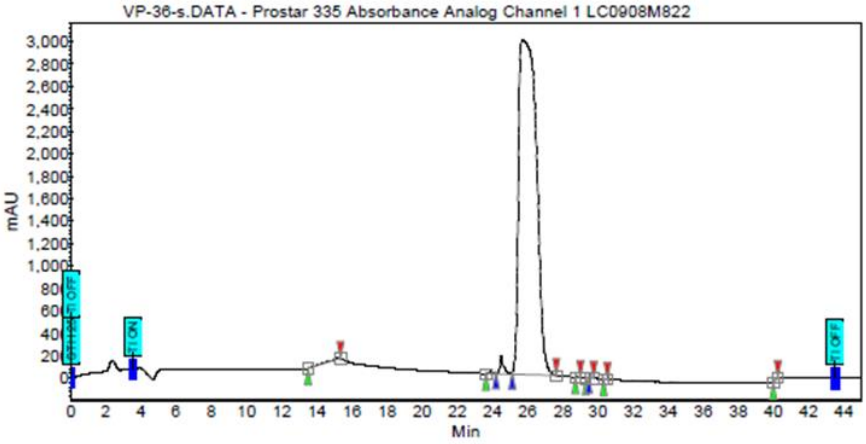

Peak results :

Index	Name	Time [Min]	Quantity [% Area]	Height [mAU]	Area [mAU.Min]	Area % [%]
1	UNKNOWN	14.41	0.33	9.4	11.7	0.330
2	UNKNOWN	23.91	0.20	34.8	7.2	0.204
3	UNKNOWN	24.48	1.15	163.0	40.7	1.151
4	UNKNOWN	25.69	98.06	2980.9	3466.1	98.064
5	UNKNOWN	28.81	0.02	4.8	0.7	0.020
6	UNKNOWN	29.43	0.01	5.0	0.5	0.015
7	UNKNOWN	29.59	0.05	13.5	1.8	0.052
8	UNKNOWN	30.41	0.02	5.8	0.6	0.018
9	UNKNOWN	40.00	0.15	38.9	5.2	0.148
Total			100.00	3256.1	3534.6	100.000

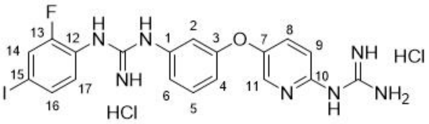

Compound **51**

System : HPLC-PDA
Method : Gradient1
User : Daniel

Acquired : 15/07/2015 15:03:39
Processed : 15/07/2015 15:50:56
Printed : 15/07/2015 16:06:45

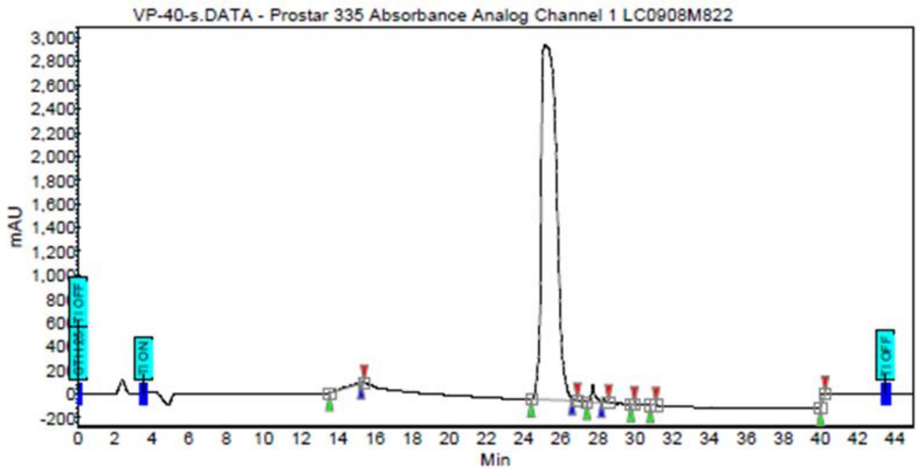

Peak results :

Index	Name	Time [Min]	Quantity [% Area]	Height [mAU]	Area [mAU.Min]	Area % [%]
1	UNKNOWN	14.47	0.43	10.6	12.3	0.431
2	UNKNOWN	15.25	0.03	8.9	0.8	0.027
3	UNKNOWN	25.20	97.52	2984.7	2792.6	97.521
4	UNKNOWN	26.64	0.09	18.1	2.5	0.087
5	UNKNOWN	27.71	1.23	149.2	35.2	1.231
6	UNKNOWN	28.33	0.14	24.2	3.9	0.137
7	UNKNOWN	29.85	0.01	3.7	0.4	0.013
8	UNKNOWN	30.95	0.05	10.7	1.4	0.047
9	UNKNOWN	40.00	0.51	112.5	14.5	0.506
Total			100.00	3322.6	2863.5	100.000

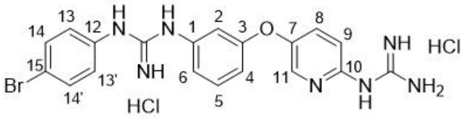

Compound **52**

System : HPLC-PDA
Method : Gradient1
User : Daniel

Acquired : 20/07/2015 11:35:15
Processed : 20/07/2015 12:22:43
Printed : 27/08/2015 08:15:21

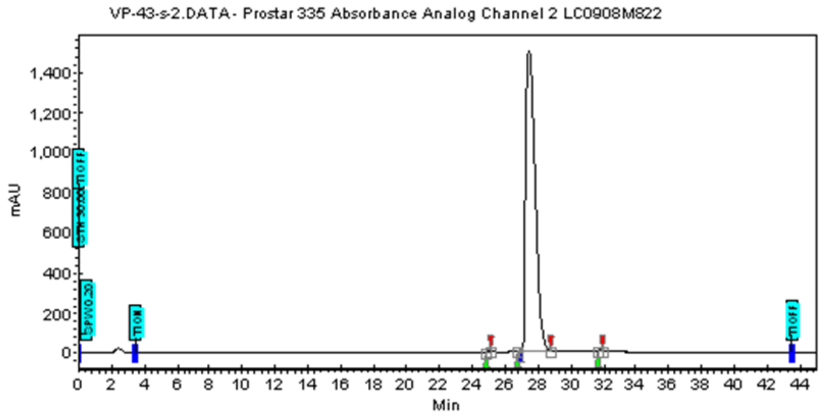

Peak results :

Index	Name	Time [Min]	Quantity [% Area]	Height [mAU]	Area [mAU.Min]	Area % [%]
1	UNKNOWN	24.97	0.05	4.5	0.5	0.046
2	UNKNOWN	26.89	0.02	1.9	0.2	0.021
3	UNKNOWN	27.45	99.80	1505.1	1002.6	99.804
4	UNKNOWN	31.80	0.13	9.0	1.3	0.129
Total			100.00	1520.6	1004.6	100.000

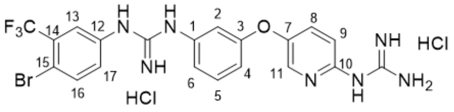

Compound **70**

System : HPLC-PDA
Method : Gradient
User : Daniel

Acquired : 10/09/2015 10:15:12
Processed : 10/09/2015 11:02:41
Printed : 10/09/2015 11:10:06

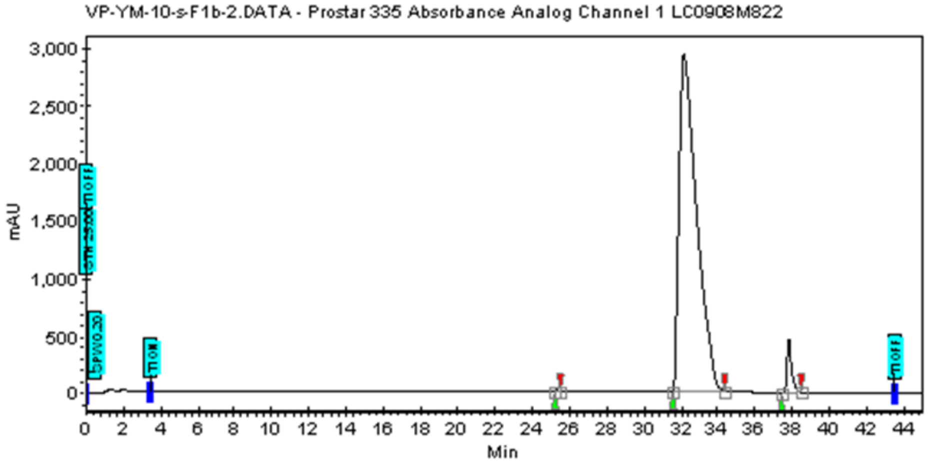

Peak results :

Index	Name	Time [Min]	Quantity [% Area]	Height [mAU]	Area [mAU.Min]	Area % [%]
1	UNKNOWN	25.37	0.02	5.0	0.8	0.021
2	UNKNOWN	32.19	96.24	2946.0	3440.2	96.239
3	UNKNOWN	37.83	3.74	471.3	133.7	3.739
Total			100.00	3422.3	3574.6	100.000

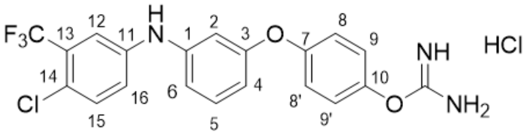

Compound **75**

System : HPLC-PDA
Method : Gradient
User : Daniel

Acquired : 09/02/2016 11:37:18
Processed : 09/02/2016 12:24:48
Printed : 09/02/2016 14:53:26

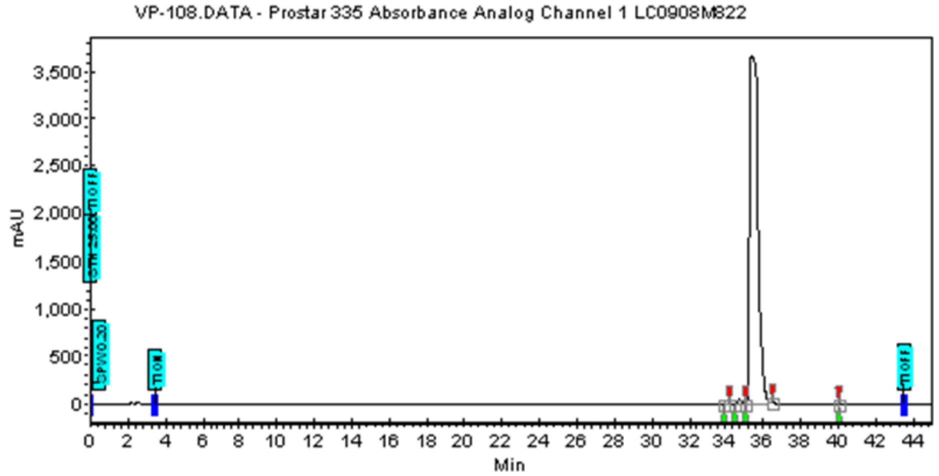

Peak results :

Index	Name	Time [Min]	Quantity [% Area]	Height [mAU]	Area [mAU.Min]	Area % [%]
1	UNKNOWN	33.97	0.07	10.4	1.3	0.067
2	UNKNOWN	34.69	0.63	66.2	12.2	0.627
3	UNKNOWN	35.39	99.30	3668.4	1928.7	99.296
4	UNKNOWN	40.00	0.01	6.4	0.2	0.011
Total			100.00	3751.4	1942.4	100.000

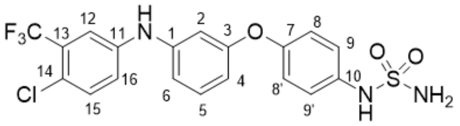

6. References

1. Diez-Cecilia, E.; Kelly, B.; Perez, C.; Zisterer, D. M.; Nevin, D. K.; Lloyd, D. G.; Rozas, I., Eur. J. Med. Chem. 2014, 81, 427.

2. Surry, D. S.; Buchwald, S. L., Chem. Sci. 2011, 2, 27.

3. Bellamy, F. D.; Ou, K., Tetrahedron Lett. 1984, 25, 839

4. Kelly, B.; McMullan, M.; Muguruza, C.; Ortega, J. E.; Meana, J. J.; Callado, L. F.; Rozas, I., J. Med. Chem. 2015, 58, 963.

5. Kurti, L.; Czakó, B., Strategic applications of named reactions in organic synthesis. Elsevier, 2005.

6. Okaniwa, M.; Hirose, M.; Imada, T.; Ohashi, T.; Hayashi, Y.; Miyazaki, T.; Arita, T.; Yabuki, M.; Kakoi, K.; Kato, J.; Takagi, T.; Kawamoto, T.; Yao, S.; Sumita, A.; Tsutsumi, S.; Tottori, T.; Oki, H.; Sang, B.-C.; Yano, J.; Aertgeerts, K.; Yoshida, S.; Ishikawa, T., J. Med. Chem. 2012, 55, 3452.

7. Oguro, Y.; Cary, D. R.; Miyamoto, N.; Tawada, M.; Iwata, H.; Miki, H.; Hori, A.; Imamura, S., Biorg. Med. Chem. 2013, 21, 4714.

8. Li, Y. N.; Urbas, A.; Li, Q., J. Org. Chem. 2011, 76, 7148.

9. Maiti, D.; Buchwald, S. L., J. Am. Chem. Soc. 2009, 131, 17423.

10. Gaussian 16, Revision C.01, M. J. Frisch, G. W. Trucks, H. B. Schlegel, G. E. Scuseria, M. A. Robb, J. R. Cheeseman, G. Scalmani, V. Barone, G. A. Petersson, H. Nakatsuji, X. Li, M. Caricato, A. V. Marenich, J. Bloino, B. G. Janesko, R. Gomperts, B. Mennucci, H. P. Hratchian, J. V. Ortiz, A. F. Izmaylov, J. L. Sonnenberg, D. Williams-Young, F. Ding, F. Lipparini, F. Egidi, J. Goings, B. Peng, A. Petrone, T. Henderson, D. Ranasinghe, V. G. Zakrzewski, J. Gao, N. Rega, G. Zheng, W. Liang, M. Hada, M. Ehara, K. Toyota, R. Fukuda, J. Hasegawa, M. Ishida, T. Nakajima, Y. Honda, O. Kitao, H. Nakai, T. Vreven, K. Throssell, J. A. Montgomery, Jr., J. E. Peralta, F. Ogliaro, M. J. Bearpark, J. J. Heyd, E. N. Brothers, K. N. Kudin, V. N. Staroverov, T. A. Keith, R. Kobayashi, J. Normand, K. Raghavachari, A. P. Rendell, J. C. Burant, S. S. Iyengar, J. Tomasi, M. Cossi, J. M. Millam, M. Klene, C. Adamo, R. Cammi, J. W. Ochterski, R. L. Martin, K. Morokuma, O. Farkas, J. B. Foresman, and D. J. Fox, Gaussian, Inc., Wallingford CT, 2016.

11. Previtali, V.; Trujillo, C.; Boisson, J.-C.; Khartabil, H.; Hénon, E.; Rozas, I. Development of the first model of a phosphorylated, ATP/Mg2+-containing B-Raf monomer by molecular dynamics simulations: a tool for structure-based design. Phys. Chem. Chem. Phys. 2017, 19, 31177-31185.

12. O. Trott, A. J. Olson, AutoDock Vina: improving the speed and accuracy of docking with a new scoring function, efficient optimization and multithreading, J. Comp. Chem. 2010, 31, 455-461.

13. Humphrey, W., Dalke, A. and Schulten, K. VMD - Visual Molecular Dynamics. J. Molec. Graphics, 1996, 14, 33-38.

14. SwissADME: a free web tool to evaluate pharmacokinetics, drug-likeness and medicinal chemistry friendliness of small molecules. Sci. Rep. 2017, 7, 42717. DOI: 10.1038/srep42717.

15. Marvin was used for calculating basicity (pKaH) of chemical structures, Marvin 17.21.0, ChemAxon (https://www.chemaxon.com).
